# Pharmacognostic Evaluation, Chemical Characterization, and Antibacterial Activity of *Bassia indica* (Wight) A.J. Scott

**DOI:** 10.3390/plants13131753

**Published:** 2024-06-25

**Authors:** Fayyaz Anjum, Saad Touqeer, Muhammad Younus Khan, QurratUlAin Jamil, Ayesha Rida, Jafir Hussain Shirazi, Syeda Abida Ejaz, Hafiz Muhammad Attaullah, Ghulam Sarwar, Zaeem Hayat Khan, Muhammad Asif Wazir, Barizah Malik, Mohammed Aufy, Shahid Muhammad Iqbal

**Affiliations:** 1 Department of Pharmacology, Faculty of Pharmacy, The Islamia University of Bahawalpur, Bahawalpur 63100, Pakistan; fayyaz.anjum@iub.edu.pk (F.A.);; 2 College of Pharmacy, Al Ain University, Abu Dhabi 112612, United Arab Emirates; 3 AAU Health and Biomedical Research Center, Al Ain University, Abu Dhabi 112612, United Arab Emirates; 4 Department of Pharmacognosy, Faculty of Pharmacy, The Islamia University of Bahawalpur, Bahawalpur 63100, Pakistan; 5 Department of Pharmacy Practice, Faculty of Pharmacy, The Islamia University of Bahawalpur, Bahawalpur 63100, Pakistan; 6 Department of Pharmaceutics, Faculty of Pharmacy, The Islamia University of Bahawalpur, Bahawalpur 63100, Pakistan; 7 Department of Pharmaceutical Chemistry, Faculty of Pharmacy, The Islamia University of Bahawalpur, Bahawalpur 63100, Pakistan; 8 Department of Botany, Faculty of Chemical and Biological Sciences, The Islamia University of Bahawalpur, Bahawalpur 63100, Pakistan; 9 Department of Pharmacy, Faculty of Pharmacy, The Islamia University of Bahawalpur, Bahawalpur 63100, Pakistan; 10 School of Biochemistry and Biotechnology, Quaid-e-Azam Campus, University of the Punjab, Lahore 54590, Pakistan; 11 Division of Pharmacology and Toxicology, University of Vienna, UZA II, Josef-Holaubek-Platz 2, A-1090 Vienna, Austria

**Keywords:** *Bassia indica*, microscopic, macroscopic, antibacterial, GC-MS

## Abstract

*Bassia indica* (Wight) A.J. Scott is an Indian origin plant with documented medicinal and nutritional value, but has not been fully characterized yet. The present study was designed to establish pharmacognostic standards for the proper identification of the *B. indica* plant and its chemical characterization. The plant was standardized with World Health Organization (WHO) standardization tools and chemically characterized by Fourier transform infrared spectroscopy (FTIR) and gas chromatography-mass spectroscopy (GC-MS) analysis. Antibacterial potential was assessed by the zone of inhibition and minimum inhibitory concentration (MIC), and molecular docking studies were also performed. Pharmacognostic evaluation established the macroscopic and microscopic parameters for the identification of whole plant and its powder. Physicochemical parameters were also set forth while quantitative phytochemical analysis showed that the ethyl acetate fraction had the highest quantity of phenols, flavonoids, and tannins. FTIR analysis showed several functional groups such as phenols, alkanes, and alcohols while 55 phytochemicals were identified in the GC-MS analysis of the crude fraction. The crude extract and other fractions showed marked antibacterial activity, while the ethyl acetate fraction showed the least MIC (1.95–31.25 mg/mL). Phytochemicals identified in the GC-MS showed good molecular docking interactions against the DNA gyrase subunit B of bacteria with binding energies ranging from −4.2 to −9.4 kcal/mol. The current study describes the pharmacognostic characterization and phytochemical profiling of *B. indica* and provides scientific evidence to support its use in infections.

## 1. Introduction

Plants have been a major source of agents used for the management of various diseases across the world for millennia and are still of contemporary importance as 80% of people worldwide depend on herbal remedies in their primary healthcare system, and over 25% of prescribed medicines in developed countries are of plant origin [1,2]. Over the last few decades, an upsurge has been witnessed in the public acceptance of natural therapies, leading to the increased usage of herbal medicines and phytonutrients. In Europe, over 1300 medicinals are being used for various ailments, while 118 out of the top 150 prescription drugs in the USA are based on natural resources [3]. Due to the increased demand for medicinal plants and the continuous loss of natural habitat, medicinal plants are facing a serious challenge to their existence. An upsurge in the usage of herbal medicines, a decrease in natural sources, and less availability of genuine crude drugs are resulting in adulteration and substitution practices [4]. Furthermore, crude drugs are collected, stored, distributed, and sold in raw form without any special packaging, which may also lead to adulteration and substitution [5].

As the use of herbal products continues to grow globally, concerns regarding their safety and public health issues are also increasingly recognized. One survey showed that 27% of herbal products commercialized worldwide are adulterated. The growing evidence of poor quality and a lack of authenticity is causing deep concerns [6]. Therefore, the proper identification of plants, their standardization, and documentation is very important for the quality of drugs. Identification includes the physical, biological, chemical, and biochemical features of plant species [7]. The WHO has set various qualitative and quantitative parameters for the standardization and quality control of herbal medicines. In pharmacognostic standardization, crude drugs are properly identified, their quality and purity is confirmed, and possible adulteration is observed. Qualitative methods of standardization such as organoleptic evaluation, toxicity testing, and bioassays contribute to the correct identification of crude drugs, while physicochemical analysis is carried out for their quantification [8].

*Bassia indica* (Wight) A.J. Scott [Syn.: *Kochia indica* Wight] belongs to the family Amaranthaceae [9]. This family includes a large number of halophytes that are well-known for their therapeutic applications in traditional medical systems and are used for infected skin wounds, internal worms, fungal infections, renal and rheumatic disorders [10] as a cardiotonic [11], anti-inflammatory, and antioxidant [12]. *B. indica* is an annual herb found in Egypt, India, Pakistan, Northern Africa, Saudi Arabia, and other countries [13]. The plant extracts are reported to have therapeutically important metabolites including polyphenols, sterols, alkaloids, glycosides, tannins, coumarins, terpenes, and saponins [14]. Moreover, it has been reported that this plant exhibits anti-inflammatory, anticancer [15], antioxidant [16], antimicrobial [17], antifungal [18], antiparasitic [14], anti-ulcer [12], and neuroprotective activities [19].

Despite the medicinal potential of *B. indica*, its pharmacognostic evaluation has not been performed yet. Therefore, the present study was designed to establish a pharmacognostic standard for the proper identification and purity of the plant to avoid adulteration. Along with this, comprehensive chemical characterization was carried out and the antibacterial activity was tested. Further in silico molecular docking was performed to identify the compounds responsible for the antibacterial effects. In vitro cytotoxicity and acute toxicity studies in mice were also conducted to establish its safety profile.

## 2. Results

### 2.1. Extract Preparation and Fractionation

A total of 264 g *B. indica* extract was obtained from 3 kg powder of the aerial parts with an 8.8% yield. During fractionation, the highest yield (77%) was observed in the aqueous fraction while the least yield (1%) was observed in the *n*-hexane fraction.

### 2.2. Pharmacognostic Evaluation

#### 2.2.1. Macroscopic Analysis 

**Figure 1:**
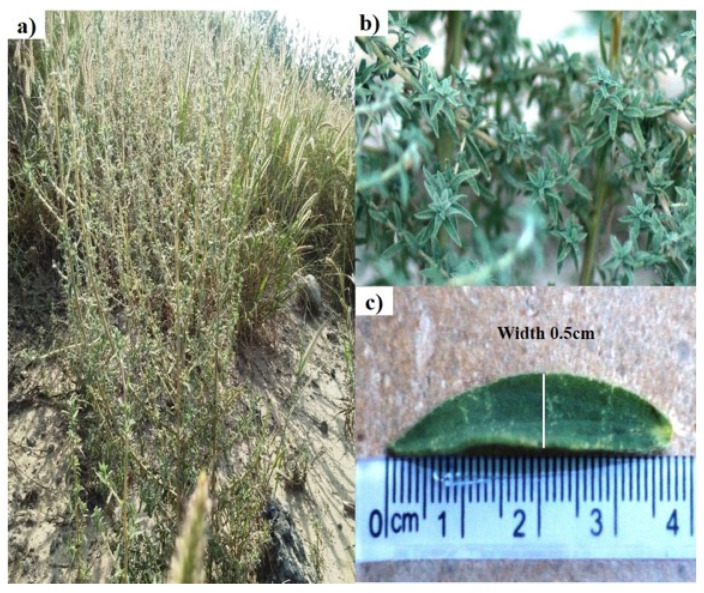
Different parts of the *B. indica* plant: (**a**) whole plant with area of growing; (**b**) plant stem and leaves showing color and summitry; (**c**) leaf indicating length and width.

The fresh leaves of the plant were hairy, dark green in color, and decussate in arrangement with lanceolate tips (Figure 1b), which turned into light brown on drying. The leaves were 3.7 cm long and had a 0.5 cm width (Figure 1c). The stem was yellowish green and flowers were inconspicuous. The plant has a characteristic odor and bitter taste.

#### 2.2.2. Microscopic Analysis

**Figure 2:**
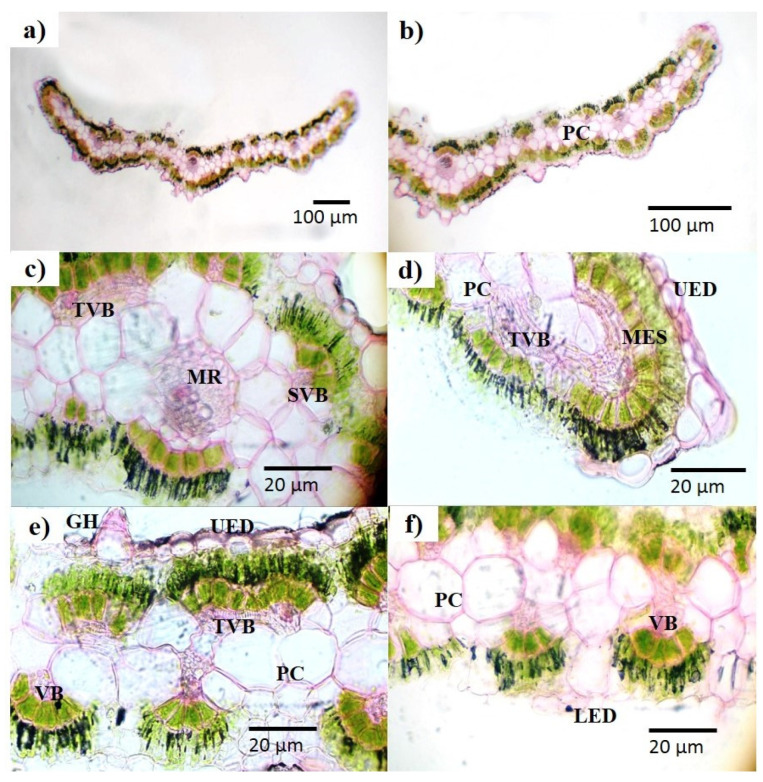
Different portions showing various tissues: (**a**) overall outline of leaf cross section (4×); (**b**) portion of same under 10×; (**c**) midrib portion; (**d**) leaf tip; (**e**) part with upper epidermis; (**f**) part with lower epidermis (40×); GH: glandular hair; LED: lower epidermis; MR: midrib; MES: mesophyll; PC: parenchyma; SVB: small vascular bundle; TVB: transverse vascular bundle; UED: upper epidermis; VB: vascular bundle.

**Figure 3:**
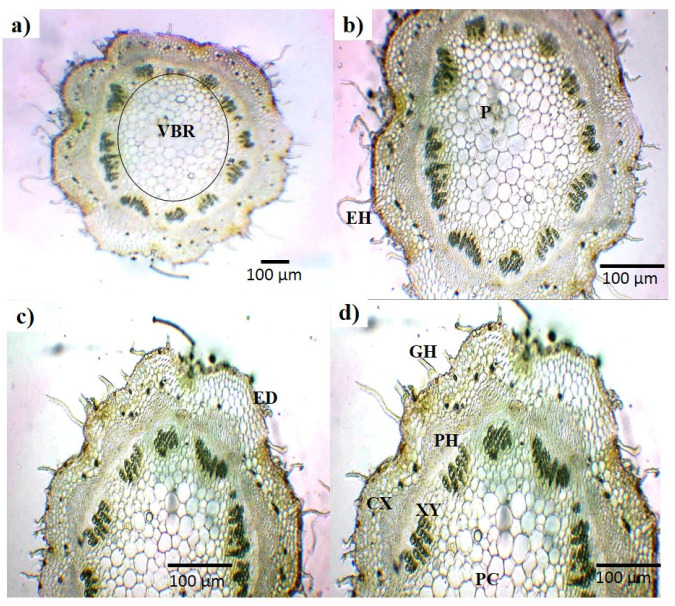
Different portions showing various tissues: (**a**) overall outline of stem cross section (4×); (**b**) portion of same under 10×; (**c**) overall showing epidermis; (**d**) different parts including glandular hair, cortex, phloem, xylem and parenchyma cells; CX: cortex, ED: epidermis, GH: e glandular hairs, P: pith, PC: parenchyma cells, PH: phloem, VBR: vascular bundle ring, XY: xylem.

**Figure 4:**
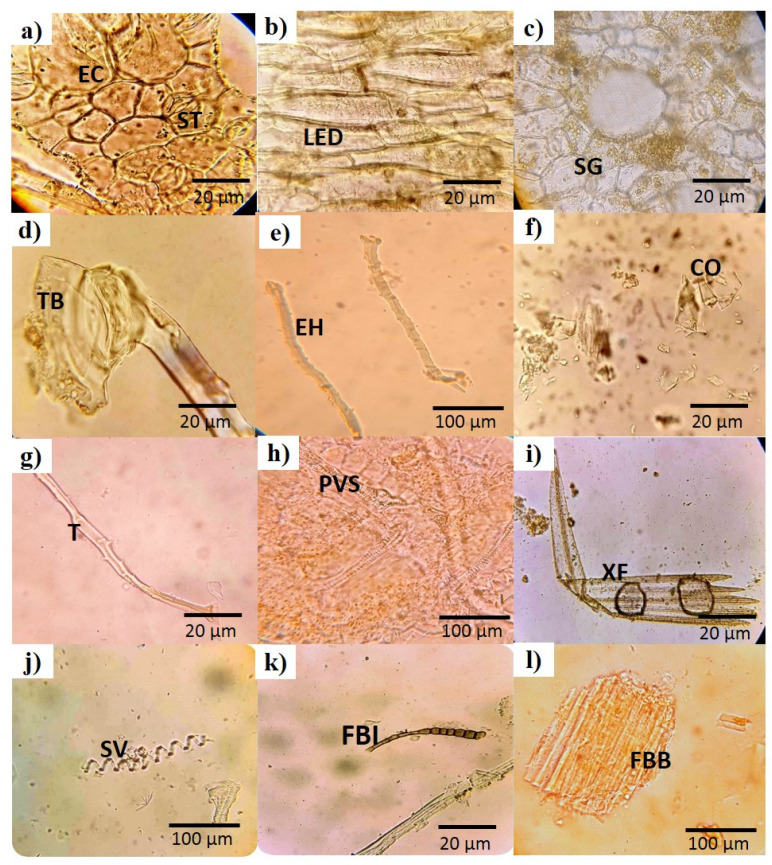
Powder microscopy showing different structures at 10× (**e**–**h**,**j**–**l**) and 40× (**a**–**d**,**i**). (**a**) epidermal cells (upper) with stomata; (**b**) lower epidermis cells; (**c**) cortical cells with starch grains; (**d**) trichome with base cell; (**e**) epidermal hair; (**f**) calcium oxalate crystals of different shape; (**g**) trichome; (**h**) pitted vascular system; (**i**) group of xylem fibers; (**j**) spiral thickening of vessels; (**k**,**l**): fiber bundle; CO: calcium oxalate crystals, FBI: intact fiber bundle, FBB: broken fiber bundle EH: epidermal hair, LED: lower epidermis, XF: xylem fibers, EC: epidermal cells, ST: stomata, SG: starch grain, SV: spiral thickening (lignin) of broken vessel, TB: trichome base, T: trichome, PVS: pitted vessel system.

In the microscopic analysis of fresh leaves, complete transverse sections (TSs) of a leaf are presented (Figure 2a). In the center of the TS, a closely packed cell area (midrib) was seen, along with a number of whitish parenchyma cells in the adjacent areas (Figure 2b,c). Between the parenchyma cells and mesophyll, vascular bundles (cylindrical shaped) could be seen (Figure 2c,d). The TS of the leaf showed that an elongated cell layer covered both sides of the leaf called the upper and lower epidermal cells (Figure 2d,f). Glandular hair and mesophyll was also seen in the TS (Figure 2e). Similarly, the complete TS of the *B. indica* stem with a vascular bundle ring in the middle is presented (Figure 3a). A small and closely packed area, called the pith, and green colored xylem could also be visualized (Figure 3b,c). These different parts and cells of the leaf and stem were also observed in the dried powder of the aerial parts of *B. indica* (Figure 4). In addition to these structures, a number of stomata cells were seen in the epidermal cells along with abundant starch grains (Figure 4a,c). Microscopic analysis also showed that the powder had pitted as well as spiral vessels (Figure 4h,j).

#### 2.2.3. Scanning Electron Microscopy

**Figure 5:**
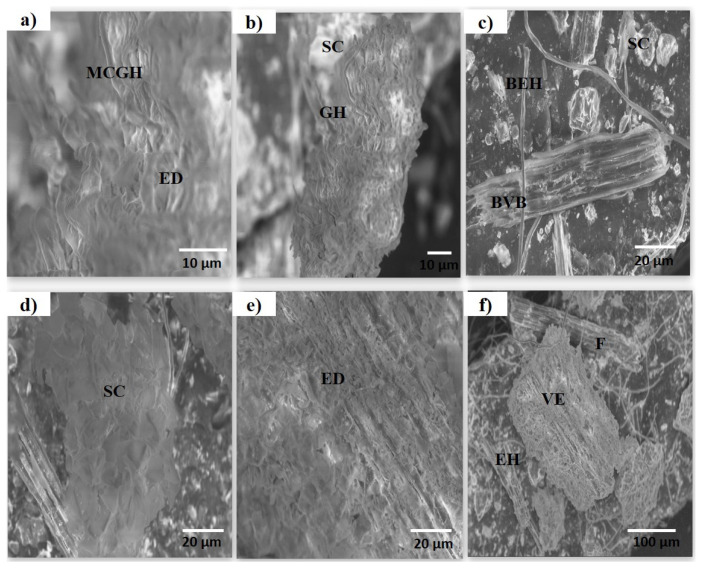
Different fields of view of scanning electron microscopy: (**a**) segment of epidermis with multicellular glandular hair and epidermal cells (600×); (**b**) portion of same as (**a**) under 250×; (**c**) powder showing broken vascular bundle, salt crystals and broken epidermal hairs; (**d**) powder showing salt crystals and broken fiber cells; (**e**) epidermis showing tightly packed cells; (**f**) segments of vessel elements, fibers and hairs (60×) BEH: broken epidermal hairs, BVB: broken vascular bundle, EC: epidermal cells, ED: epidermis, EH: epidermal hair, F: fibers, GH: glandular hair, MCGH: multicellular glandular hairs, SC: salt crystals, VE: vessel elements.

*B. indica* powder was observed under a scanning electron microscope for detailed anatomy. SEM analysis of the aerial parts (powder) showed the presence of epidermal and stomata cells embedded in fibers like a vascular bundle (Figure 5a). Many salt crystals and broken bundles of vessels were also seen (Figure 5b–d). Under the scanning electron microscope, epidermal hairs and fibers were seen, which can be an important parameter for plant identification (Figure 5f).

#### 2.2.4. Extractive Value and Swelling Index

Percent extractive values of the *B. indica* powder was carried out in solvents with different polarities. The maximum extractive value was observed in the aqueous solvent (10.5%) and the minimum for *n*-hexane (Table 1). The swelling index of the plant powder was 0.6 cm.

#### 2.2.5. Nutritional and Elemental Analysis

The nutritional analysis of the powder showed the presence of the highest amount of crude fibers (24.21%), followed by carbohydrates and proteins (Table 2). The total caloric value was 111.43 kcal/100 g of dry powder. The atomic absorption spectroscopy of the *B. indica* extract showed the presence of different elements (Table 2), where the analysis showed the highest amount of calcium (470 ppm), followed by iron (161 ppm) and magnesium (55.6 ppm). The content of heavy metals (i.e., lead, cadmium, chromium, copper, and zinc) was less than the WHO maximum permissible element values. 

#### 2.2.6. Fluorescence Analysis

Fluorescence analysis of the powdered aerial parts of *B. indica* was carried out with various reagents that showed different shades of colors such as light and dark brown, light greenish, black greenish, and purple (Table 3). Fluorescence analysis is an important parameter adopted to confirm the quality of the crude drug as different secondary metabolites present in the crude material exhibit different fluorescence under visible and ultraviolet (UV) light when treated with different reagents.

#### 2.2.7. Thin Layer Chromatography

In the TLC profile, different fluorescent compounds were separated depending upon their affinity toward the mobile phase polarity. Brown spots were observed when TLC plates were seen under UV light at 254 nm. The maximum number (5) of spots were seen when the chloroform and methanol (4:1) mobile phase was used. The appearance of multiple spots on the TLC plates suggests the presence of secondary metabolites like flavonoids, phenolic compounds, glycosides, and saponins. The Rf values of different spots are listed in Table 4.

### 2.3. Phytochemical Analysis

#### 2.3.1. Qualitative Analysis

The presence of different important bioactive secondary metabolites was detected by phytochemical analysis. The appearance of different colors and precipitates confirmed the presence of several secondary metabolites including alkaloids, carbohydrates, flavonoids, glycosides, phenols, saponins, and tannins.

#### 2.3.2. Quantitative Analysis of Total Phenols, Tannins, Flavonoids, and Saponins

Therapeutically important phytoconstituents like phenols, flavonoids, and tannins present in the extract were further quantified (Table 5). The quantitative analysis showed that the ethyl acetate fraction contained the highest amount of total phenols (419.10 ± 11.76), tannins (249.60 ± 10.50) mg tannic acid equivalent per gram of dried extract (mg TAEg^−1^), and flavonoids 161.790 ± 1.375 mg quercetin equivalent per gram of dried extract (mg QEg^−1^). The saponin contents were greater in the aqueous fraction (40.5 ± 0.9%) compared to the crude extract (30.9 ± 2.2%). Significant amounts of these secondary metabolites present in the plant suggest that *B. indica* may have therapeutic potential. 

### 2.4. FTIR Spectroscopy

**Figure 6:**
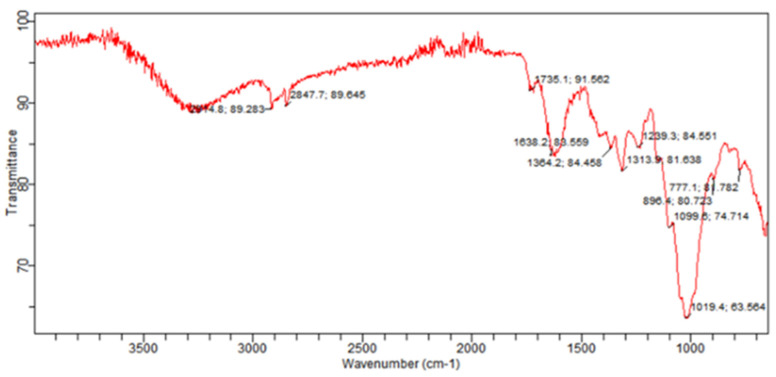
Fourier transform infrared spectrogram of *B. indica* showing different peaks.

The *B. indica* extract was also characterized with FTIR (Figure 6). The FTIR of the crude extract showed 11 characteristic peaks ranging from 777.1 to 2914.8 cm^−1^ in the frequency range and important medicinal functional groups (Table 6).

### 2.5. GC-MS Analysis

**Figure 7:**
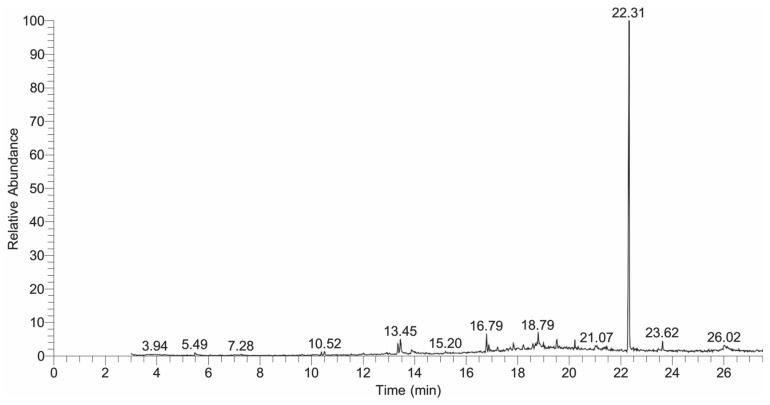
GC-MS chromatogram of the *n*-butanol fraction of the *B. indica* extract.

**Figure 8:**
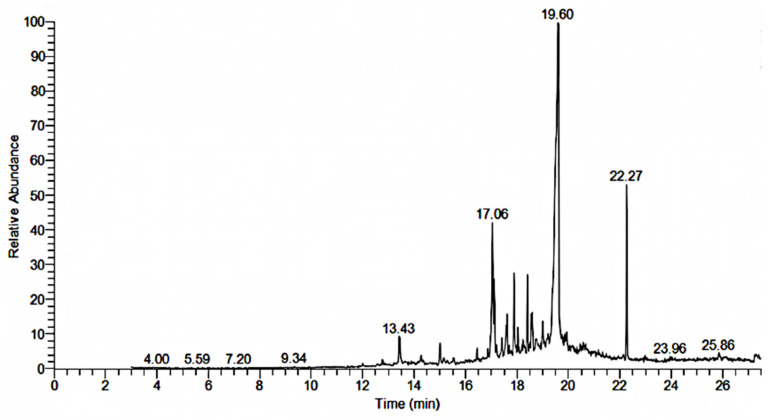
GC-MS chromatogram of the ethyl acetate fraction of the *B. indica* extract.

**Figure 9:**
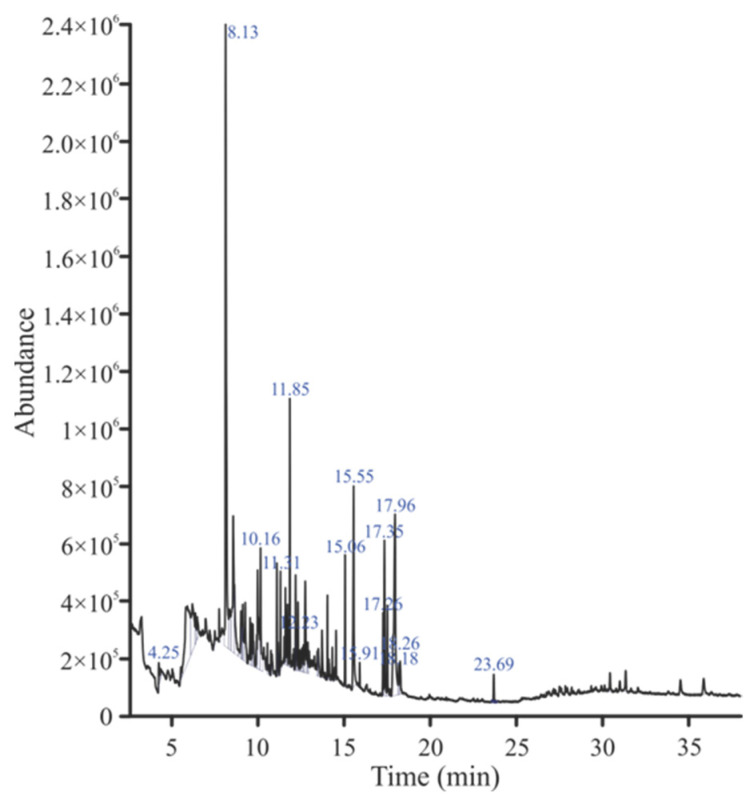
GC-MS chromatogram of the *n*-hexane fraction of the *B. indica* extract.

GC-MS analysis of the *n*-butanol, ethyl acetate, and *n*-hexane fractions were performed and their chromatograms are shown in Figure 7, Figure 8 and Figure 9, respectively. A total of 30 compounds were detected in the *n*-butanol fraction while 25 compounds were found in the ethyl acetate fraction (Table 7 and Table 8). Six compounds were common among the *n*-butanol and ethyl acetate fractions including dodecane, 5,8-diethyl-17-pentatriacontene, 1,2-benzenedicarboxylic acid, mono(2-ethylhexyl) ester, 1,2-benzenedicarboxylic acid, diisooctyl ester, 24,25-dihydroxycholecalciferol, and 9,12,15-octadecatrienoic acid, 2,3-bis[(trimethylsilyl)oxy]propyl ester, (Z,Z,Z)-. The total number of compounds detected in the *n*-hexane fraction was 62, out of which 14 compounds had a qual factor more than 80 (Table 9). One compound 10,13-octadecadiynoic acid, methyl ester was found to be common among the *n*-butanol and *n*-hexane fractions. Similarly, the ethyl acetate and *n*-hexane fractions also contained isoaromadendrene epoxide as a common compound.

### 2.6. Acute Toxicity and Cytotoxicity

Oral acute toxicity was performed on mice and no mortality was observed. Furthermore, no sign of any toxicity like behavioral changes and any apparent injury or deformity was seen in animals throughout the study. Therefore, the extract was found to be safe up to a dose of 2000 mg/kg. The MTT cytotoxicity assay, carried out in mouse fibroblast cells, showed that *B. indica* is safe and did not cause any toxicity.

### 2.7. Antimicrobial Activity

#### 2.7.1. Disc Diffusion Method

The antimicrobial assay of the crude extract and its *n*-hexane, dichloromethane, ethyl acetate, *n*-butanol, and aqueous fractions showed good antibacterial activity against Gram-positive and Gram-negative bacteria. The zone of inhibition at 10, 30, and 100 mg/mL of all fractions was measured (Table 10). Results were dose dependent and the largest zone of inhibition (27 mm) was seen in the ethyl acetate fraction against *E. coli.* The aqueous extract was more effective at a lower dose than a higher dose; no growth inhibition of *B. subtilis* was seen at 100 mg/mL. The effects were comparable to the standard drug ceftriaxone (1 mg/mL); the fractions were more effective against *E. coli*, and the least effective against *P. aeruginosa*.

#### 2.7.2. Minimum Inhibitory Concentration

The MIC was also observed and the results indicated that the ethyl acetate fraction had the lowest minimum inhibitory concentration against all of the tested bacterial strains ranging from 1.95 to 31.25 mg/mL. The MIC was the lowest (1.95 mg/mL) against *B. subtilis* and highest (31.25 mg/mL) against *S. typhi*. The *n*-butanol fraction showed overall promising results, with the MIC ranging from 3.91 to 7.81 mg/mL. The *n*-hexane fraction was found to be the least effective (MIC > 100 mg/mL) against the tested strains, with zero response against *E. coli* (Table 11).

### 2.8. Molecular Docking

**Figure 10:**
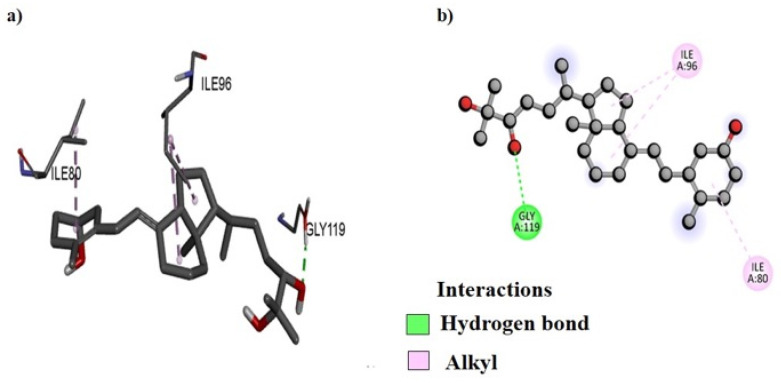
The predicted 3D (**a**) and 2D (**b**) binding modes of compound NB23 against the DNA gyrase subunit B of *P. aeruginosa.*

**Figure 11:**
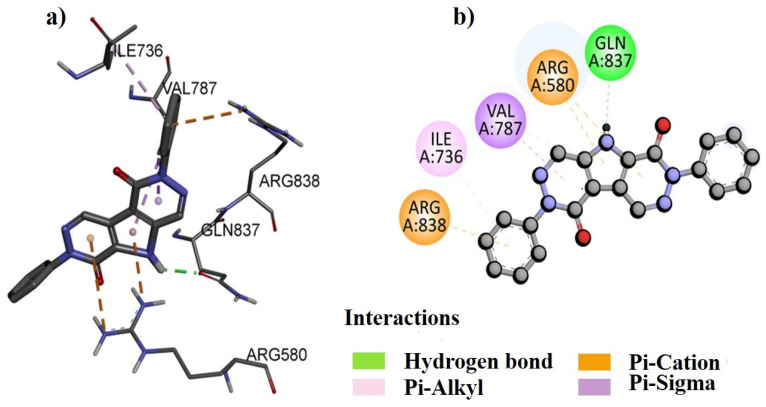
The predicted 3D (**a**) and 2D (**b**) binding modes of compound EA19 against the DNA gyrase subunit B *of* S. *typhi.*

**Figure 12:**
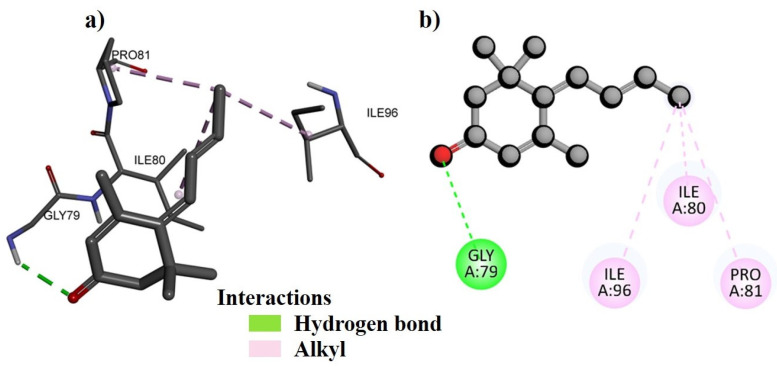
The predicted 3D (**a**) and 2D (**b**) binding modes of compound NH4 against the DNA gyrase subunit B of *P. aeruginosa.*

DNA gyrase was chosen as the molecular docking target for the *B. indica* extracts for several reasons. Firstly, DNA gyrase plays a crucial role in bacterial DNA replication and transcription, making it a potential target for antibacterial agents. Secondly, it is a well-validated target for existing antibacterial drugs, enhancing the likelihood of finding effective compounds in the extracts. Additionally, numerous studies have shown that plant-derived compounds can inhibit DNA gyrase [20], suggesting that *B. indica* may contain phytochemicals that target the DNA gyrase. Additionally, targeting DNA gyrase also offers the advantage of broad-spectrum antibacterial activity and can potentially complement other antibacterial mechanisms, leading to synergistic effects. These factors collectively justify the focus on DNA gyrase in identifying antibacterial compounds in *B. indica* [21]. Our analysis revealed that several amino acids were involved in forming both bonding and non-bonding interactions with the ligand. The docking protocol was validated by first separating the co-crystal ligand from the active pocket of the complex, and then re-docking was performed to validate its accuracy. The co-crystal ligand docking score and figures are given in the Supplementary Materials (Table S1; Figures S1–S9). The molecular docking scores of selected compounds of different fractions show that the best docked compound, NB23, from the *n*-butanol fraction bound with a −8.4 kcal/mol binding energy (Table 12). The 2D and 3D binding interactions between NB23 and the active pocket of DNA gyrase subunit B of *P. aeruginosa* were also visualized (Figure 10). Amino acid residue GLY119 interacts via forming conventional hydrogen bonds while lLE96 and ILE80 interact by alkyl linkage. Similarly, the best docked compound, EA19, from the ethyl acetate fraction bound with a −9.4 kcal/mol energy within the active pocket of DNA gyrase subunit B of *S. typhi* and its 2D and 3D binding interactions are presented in Figure 11. The docking analysis shows that amino acid residue GLN837 was found to have conventional hydrogen bond interactions with EA19 while ARG580 and ARG838 formed pi-cation, and the VAL787 and ILE736 amino acid residues interacted with EA19 by pi-sigma and pi-alkyl linkages (Figure 11). The binding interactions of NH4 with the active site of DNA gyrase from *P. aeruginosa*, both in two-dimensional (2D) and three-dimensional (3D) visuals are also presented in Figure 12. The amino acid GLY79 in the DNA gyrase established conventional hydrogen bonds with NH4. Furthermore, three other amino acids, ILE96, ILE80, and PRO81, formed alkyl interactions with NH4.

## 3. Discussion

The standardization of medicinal plants is of great importance as it plays a legitimate role in understanding its botanical value, chemical structure, and clinical suitability; moreover, it may be helpful in finding morphologically similar and adulterated species [7,22]. The World Health Organization has laid down many standardization parameters that ensure the safety, efficacy, and quality of crude drugs [23]. The first and quickest method of drug identification and adulteration or purity is macroscopic evaluation. Macroscopic evaluation of the plant showed that it has green colored lanceolate leaves, a hairy stem, fibrous texture, and other characteristic parameters (Figure 1). These results allow for the easy identification of *B. indica* when harvesting from its natural habitat and preventing unintentional adulteration. These organoleptic characters are consistent with the previously reported description of the *B. indica* plant [24,25]. Microscopic evaluation parameters have an important role in the identification and validation of the plant. Anatomical histology and powder microscopy act as excellent tools for the authentication, adulteration, or purity of crude drugs [26]. Anatomical structures observed in the present microscopic study of the leaf, stem, and powder provide necessary information about the morphological characteristics of *B. indica*, which can aid in the identification and authentication of the plant material [27]. Similar structures have also been reported in other plants of the same genus [28,29]. Stomata observed in the epidermis prevent a cellular imbalance in plants; this adaptive strategy helps the plant to survive in drought conditions. Trichomes maintain still air on the leaves and are involved in secretory functions [30]. Scanning electron microscopy (SEM) was used to measure and describe the surface morphology of the plant material. Such morphological characteristics are important parameters in the identification of the sample [31]. Xylem observed in the SEM images had a narrow opening, which allows for the capillary movement of water and decreases its loss; the phloem ensures the storage of food. Therefore, the water conservation and food storage ability of different parts of plants help the plant survive in drought conditions. Similar anatomical features have also been observed in other plants growing in drought conditions [30,32,33] Moreover, these observations provide information about the physical characteristics of the plant that may affect its solubility [28]. Extraction with different solvents showed the maximum yield in the aqueous solution. These results indicate that the plant material contains more polar compound (i.e., alkaloids, flavonoids, phenolics, saponins, and polysaccharides). It is reported that polar compounds often exhibit different biological activities such as antioxidant, anti-inflammatory, antimicrobial, and anticancer activities. A high aqueous extractive value indicates the presence of polar compounds, which imparts medicinal importance to plants [15,34]. Swelling of the plant material, when soaked in a solvent, indicates the presence of mucilage found in the crude drug. The swelling index of the powder observed in the current study was in line with previously reported studies performed on the other halophytic plant *Salsola kali*. Mucilages are mainly polysaccharides, and the swelling index properties are specific to their pharmaceutical utility [28]. The nutrients present in plants are vital for health and growth promotion as well as preventing many diseases. [35]. The nutritional analysis of *B. indica* showed the presence of fibers in a high amount, followed by carbohydrates and proteins, just like other nutritional plants [26,36]. The high ash value depicts a good amount of minerals in plants [8]. Minerals are very important for living organisms to maintain their physiological and biochemical functions. Plants are considered a good source of minerals but may also contain toxic heavy metals that may lead to dangerous consequences to human health when ingested [26,37]. The *B. indica* extract contained a significant amount of important minerals such as calcium, iron, and magnesium. These results were in line with other reported studies [38]. Heavy metals like lead, copper, chromium, and cadmium quantified in the extract did not exceed the safety threshold prescribed by the WHO [39]. Forage, having a considerable amount of elements and nutrients, improve the growth, reproduction, and performance of animals [24,40]. It is reported that *B. indica* is used as forage, which increases the performance, growth, and milk yield of cattle [24,41].

Crude drugs may also be assessed qualitatively by their fluorescence pattern [42]. The fluorescence analysis of the extract showed different fluorescent colors when treated with different organic and inorganic solvents and observed under daylight and UV light. Different fluorescent colors indicated the presence of multiple compounds in the plant, as claimed in previously reported studies [43]. Phytochemical analysis of the crude extract showed the presence of important secondary metabolites such as phenolics, flavonoids, tannins, and saponins, which were further quantified. The results of the current study were similar to the reported phytochemical profile of other plants of the genus *Bassia* like *B. scoporia*, and *B. muricata*, etc. [10,17,44]. The presence of phenols and flavonoids makes *B. indica* a plant of therapeutic potential. It is a well-documented fact that polyphenols are involved in various human health benefits and strongly linked with antioxidant, antimicrobial, anti-inflammatory, cardioprotective, and anticancer activities [45,46]. In a previous study, phenolic compounds exhibited significant antimicrobial activity [47], so the antimicrobial effects of *B. indica* observed in the current study may be attributed to phenols. Tannins are also polyphenols, which precipitate the proteins and form complexes with enzymes, thereby inhibiting their activity. Tannins are known for their antimicrobial activities and are used in wound healing and anti-inflammatory activities [48]. Saponins are glycosides and have many therapeutic activities including diuretic, ant-inflammatory, antimicrobial, cardioprotective, and many other biological activities [49]. The functional groups observed in the FTIR analysis of *B. indica* further confirmed the phytochemical results, and this test can be used to control the quality and standardization of the plant material, as conducted in previously reported studies [50]. 1,2-Benzenedicarboxylic acid, diisooctyl ester, and 1,2-benzenedicarboxylic acid, mono(2-ethylhexyl) ester were the most abundant compounds observed in the GC-MS analysis of the *n*-butanol fraction, and their presence has also been reported in many other medicinal plants with antimicrobial, anticancer, antifungal, antioxidant and anti-inflammatory properties [51,52,53]. Squalene observed in the current study has also been found in the phytochemical screening of many other plants, is considered as a natural antioxidant, and possesses cholesterol lowering, anticancer, immune enhancing, and chemopreventive activities [54]. Other identified compounds like 24,25 dihydroxycholcalciferol, oleic acid, 3-(octadecyloxy)propyl ester, azafrin, and rhodopin have also been identified in other plants and is reported to have calcium regulation, antifungal, cardioprotective, and antineoplastic activities, respectively [55,56,57]. In the ethyl acetate fraction, 4,4-dimethyladamantan-2-ol was the most abundant compound, and is also found in the essential oil of plants with antimalarial and antioxidant activities [58,59]. Other compounds like agathic acid, gitoxigenin, betulin, and stigmasterol have previously been isolated from other plants and shown to have antileishmanial and abortifacient [60], cardioprotective [61], antioxidant, anti-inflammatory, anticancer [62] anti-osteoarthritis, anti-inflammatory, and antibacterial [63] properties, respectively. Similarly, 5-methylfurfural, 2-methoxy-4-vinylphenol, Ar-turmerone, and hexadecanoic acid, methyl esters found in the *n*-hexane fraction have also been found in different plant extracts and reported to have acaricidal, anti-inflammatory, anticancer [64], anti-inflammatory, anti-nociceptive, anti-proliferative [65], and antibacterial activities [66], respectively. An oral acute toxicity study performed in mice and the MTT cytotoxicity assay carried out in mouse fibroblast cells showed that *B. indica* is safe and did not cause any toxicity.

The *B. indica* crude extract and all fractions showed good antibacterial effects against Gram-positive and Gram-negative strains. It was observed that the ethyl acetate fraction was more effective against bacterial strains, followed by the *n*-butanol fraction. The most abundant compound of the *n*-butanol fraction 1,2-benzenedicarboxylic acid, diisooctyl ester was previously isolated from the root of *Plumbago zeylanica* Linn and tested against different bacterial strains, which showed good antibacterial results [53,67]. Therefore, the present antibacterial results of the *n*-butanol fraction may be due to the presence of a large amount of the 1,2-benzenedicarboxylic acid, diisooctyl ester compound. Similarly, hexadecanoic acid, methyl ester, identified in the *n*-hexane fraction, was also isolated from clove, which showed antibacterial effects [66]. Furthermore, in the current study, it was observed that the *B. indica* extract had a considerable amount of polyphenols and tannins, and the observed antibacterial effects may also be attributed to these secondary metabolites [48]. Moreover, to provide a theoretical rectification to the antibacterial investigations, the major compounds of different fractions identified in the GC-MS analysis were subjected to in silico computational analysis against the DNA gyrase subunit B of various bacteria. Computational studies like molecular docking help to predict the least good binding energy and high affinity as well as a better framework between protein and ligand interactions [68]. The amino acid residues interact with the DNA gyrase through hydrogen bonds, pi-cation, pi-sigma, pi-alkyl, and alkyl interactions, which play an important role in the overall stability and specificity of the binding between the two molecules [69].

## 4. Materials and Methods

### 4.1. Plant Collection

Aerial parts (Stem, branches and leaves) of *Bassia indica* (Wight) A.J. Scott were collected in bulk (10 kg) from the district pf Muzaffargarh (Punjab) in Pakistan [31°10′5″ N 70°50′25″ E] in June 2021, early morning before flowering, and were identified by a botanist, Dr. Ghulam Sarwar, Department of Botany, The Islamia University of Bahawalpur. The specimen was stored and voucher no. 324/Botany was issued. Plant material was cleaned, shade dried, and crushed into a coarse powder. A small amount of powder was stored in closed amber glass bottles for microscopic analysis.

### 4.2. Extract Preparation and Fractionation

Plant extraction was performed as described previously with minor modifications [70]. Briefly, the powdered plant material (3 kg) was soaked into an aqueous methanol (30:70) solvent for 7 days with daily stirring and shaking. The soaked material was filtered and evaporated under reduced pressure and temperature until a thick semisolid crude extract was obtained. Then, 150 g of the extract was used for fractionation, and the remaining portion was stored in a freezer at −20 °C for further use. The compounds present in the extract were then separated depending upon their polarity by the separation funnel method [71]. Solvents of different polarities were selected for this purpose, and 150 g of extract was dissolved in 250 mL of distilled water and extracted successively with an equal volume of different organic solvents (i.e., *n*-hexane, dichloromethane, ethyl acetate, and *n*-butanol, respectively). All of the solvent fractions were then evaporated under reduced pressure (150 mbr) and temperature (40 °C) to obtain thick extracts. The percentage yield of the crude extract and fractions was calculated, and the extracts were stored in closed containers at −20 °C for further studies.

### 4.3. Experimental Animals

Albino mice of either sex weighing about 30–50 g were housed in the animal house of the Pharmacology Research Lab, Department of Pharmacology, Faculty of Pharmacy, The Islamia University of Bahawalpur. The animals were housed in polycarbonate cages with sawdust (renewed every 48 h) under standard lab conditions (temp: 25 ± 2 °C; humidity 60 ± 5%), a light and dark cycle of 12 h was maintained, and they were fed standard diet and water ad libitum. The study was carried out according to the guidelines of the Pharmacy Animal Ethics Committee (AEC file no PAEC/23/101).

### 4.4. Pharmacognostic Evaluation

#### 4.4.1. Macroscopic Studies

Cleaned and shade dried samples of the *B. indica* plant were observed for color, shape, size, odor, taste, and texture. Similarly, the leaves and stem were powdered and observed for color, odor, and taste [72].

#### 4.4.2. Microscopic Studies

Microscopic studies of the *B. indica* fresh leaves and stem were performed under a simple compound microscope. Fresh, cleaned, small-sized (5–10 mm) parts of the leaf and stem were placed into the potato block and sections were cut with a razor blade manually and dipped into water in a Petri dish. Uniform and small-sized sections (5–15 µm) were stained and observed under a camera-fitted microscope (4× and 10×) [72]. Similarly, dried powder of the aerial parts of *B. indica* was investigated following the previously described method with slight modifications [73]. A few particles of fine powder material were placed on cleaned glass slides and 1–2 drops of 10% chloral hydrate, 50% glycerin, and 5% iodine solution was added separately and mixed well with a needle. Glass cover slides were placed, and various structures were observed under the microscope at 10×, 50×, and 100×.

#### 4.4.3. Scanning Electron Microscopy

The surface morphology of the powder was assessed by keeping the powder in the chamber of a scanning electron microscope. Scanning was performed at various magnifications ranging from 900 to 4500 cm^−1^ at 10.0, 20.0, and 100.0 μm [74].

#### 4.4.4. Swelling Index

The swelling index was calculated by adding 2 g of plant powder in a 100 mL measuring cylinder with 50 mL of water. The cylinder was gently shaken many times, allowed to stand for 24 h, then the volume occupied by the sample was measured [75].

#### 4.4.5. Extractive Value Determination

The plant powder (2 g) was macerated in *n*-hexane, ethyl acetate, dichloromethane, *n*-butanol, and distilled water (20 mL each) separately in air tight bottles for 7 days with intermittent shaking. The extract was filtered in pre-weighted flasks, then the filtrate was evaporated and the flasks were weighed again. The extractive value (percentage) was calculated using the following formula [72]:

(1) $$Extractive\;value\left(\%\right)=\frac{weight\;of\;extract}{weight\;of\;powder}\times 100$$

#### 4.4.6. Elemental and Nutritional Analysis

The quantities of the different metals and minerals in the extract were measured with the help of an atomic absorption spectrophotometer (Hitachi Polarized Zeeman AAS, Z-8200, Tokyo, Japan) under the conditions described in the AOAC (1990). Metals assessed in the extract were calcium (Ca), chromium (Cr), copper (Cu), cadmium (Cd), iron (Fe), lead (Pb), manganese (Mn), magnesium (Mg), nickel (Ni), and zinc (Zn). Commercially available stock solution (Applichem^®^, Darmstadt, Germany) was used for the preparation of calibrated standards in purified de-ionized water (1000 ppm). The glass apparatus used during the procedure of experimental work was kept overnight in 8 N HNO_3_ and cleaned with de-ionized water many times before use [76]. Nutritional contents such as carbohydrates, fats, proteins, and fiber percentage were calculated according to the AOAC 2005 guidelines and method adopted by Zaman et al. The carbohydrate percentage and total energy was calculated by the following equations [26]:

(2) Carbohydrate content=100−(%moisture+%protein+%crude lipid+crude fiber+%ash)

(3) $$Total\;caloric\;value\left(\frac{Kcal}{100\;g}\right)=\left(2.62\times protein\right)+\left(4.2\times carbohydrate\right)+8.37\times fat$$

#### 4.4.7. Fluorescence Analysis

A small quantity of the powder was soaked in different reagents as mentioned including methanol, ethyl acetate, dichloromethane, chloroform, *n*-butanol, *n*-hexane, 50% HNO_3_, picric acid, conc. H_2_SO_4,_ 50% HCl, 1% NaOH, iodine, and 5% FeCl_3_. After 5 min, all mixtures were observed in visible light and under a UV lamp at 254 nm for fluorescence [5].

#### 4.4.8. Thin Layer Chromatography

Thin layer chromatography of the plant extract and its fractions was carried out using various mobile phases of different concentrations and silica gel 60 backed aluminum TLC plates. The mobile phase was poured into a closed tank, and saturation was achieved by placing the filter paper across the walls of the tank. The sample extracts dissolved in different solvents were placed on a TLC plate with the help of a capillary tube, 1.5 cm above the base of the plate, and allowed to dry. Then, the plates were placed in a chromatographic tank until the elevation of the mobile phase to the upper mark. Then, the plates were removed, dried, and observed under a UV lamp (λ = 254 nm) to visualize the separated compound spots, and their Rf value was calculated [77].

### 4.5. Phytochemical Analysis

#### 4.5.1. Qualitative Analysis

For the detection of various phytochemicals present in the crude extract, a standard procedure was followed with some modifications [70]. Briefly, Molisch’s test was used for the detection of carbohydrates, which turns a purple color in their presence. The formation of a yellow precipitate in Hager’s reagent confirmed the alkaloids. The extract was treated with FeCl_3_ and H_2_SO_4_ for the detection of phenol and flavonoids, respectively. A brown colored ring at the interface in the Keller–Kiliani test showed the presence of glycosides in the extract. A blackish precipitate with the ferric chloride solution and a pink color with HCl confirmed the tannins and resins in the extract, respectively. The formation of persistent froth while vigorous shaking of the extract with water exhibited the presence of saponins.

#### 4.5.2. Quantitative Analysis of Total Phenols, Tannins, Flavonoids, and Saponins

The total phenol and tannins were estimated using the Folin–Ciocalteu method. A standard calibration curve was constructed using tannic acid solution. Different volumes (0, 20, 40, 60, 80, and 100 μL) of standard tannic acid solution (10 mg/mL) were poured into a test tube and the final volume (500 μL) was made with distilled water. Solutions were mixed with 250 µL 1 N FC reagent and 1.25 mL of 20% sodium carbonate, and the absorbance was measured (λ = 725 nm) after 40 min of incubation at room temperature. Similarly, 10, 30, and 100 μL of the extract stock solution (100 mg/mL) were diluted and mixed with FC reagent and sodium carbonate, and the absorbance was measured after incubation. The total phenols were estimated as tannic acid equivalent from the calibration curve and the results were expressed as the total phenolic milligram per gram of extract on a dry matter basis. Total tannins were determined by separating and subtracting non-tannin phenols from the total phenols. For the estimation of non-tannin phenols, 1 mL of each extract stock solution (100 mg/mL) and distilled water was mixed with 100 mg polyvinyl polypyrrolidone (PVPP), vortexed, and incubated at 4 °C for 15 min. Then, the mixture was centrifuged at 3000 rpm for 10 min, and the supernatant, containing non-tannin phenol, was collected. Next, 20, 60, and 200 µL of the supernatant were diluted and mixed with FC reagent and sodium carbonate as above-mentioned, and the absorbance was taken. Values were interpolated against the tannic acid calibration curve [78]. The tannin contents were calculated with the following equation

(4) $$Tannins\%=\left(\%total\;phenolics\right)-(\%non-tannin\;phenolics)$$

For the estimation of the total flavonoid content, 50 µL of the extract sample (1 mg/mL) was mixed with 100 µL methanol in a 96-well microplate. Then, 20 µL 10% AlCl_3_ was added in the well and gently shaken. After 3 min of incubation, 20 µL 1 M CH_3_COONa was added to the well, followed by the addition of 60 µL of methanol. One well containing 150 µL methanol was used as the blank. The microplate was then further incubated for 40 min in the dark and the absorbance was measured at 430 nm using a microplate reader. A standard curve of quercetin (concentration ranging from 7.81 to 500 mg/mL, 2-fold dilution) was drawn under the same procedure. Flavonoid contents were expressed in milligram equivalent to quercetin per gram of dry extract (mg QE/g) [79].

The total saponin content present in the extract and its aqueous fraction were estimated by treating 1 g of extract with 20% acetic acid in ethanol and allowed to stand for 24 h at 50 °C. The mixture was filtered, concentrated in a water bath to one quarter of its initial volume, and then precipitated with NH_4_OH. Precipitates were separated by filtration, and the saponin content was calculated with the following equation [80].

(5) $$Saponin\;content=\frac{W_{2}-W_{1}}{Sample\;weight}\times 100$$

where W_2_ is the weight of the filter paper + residue and W_1_ is the weight of the empty filter paper.

### 4.6. FTIR Spectroscopy

Extracts can also be standardized by identifying different functional groups through FTIR analysis. The FTIR analysis of the extract was carried out at room temperature using a Bruker FTIR (Tensor 27 series, Ettlingen, Germany) scanning over the frequency range of 4000–650 cm^−1^ at a resolution of 4 cm^−1^. Attenuated total reflectance (ATR) technology along with OPUS data collection software (version 7.8) was used to collect the spectra. A sample of the extract was kept on the pike miracle ATR cell with a zinc selenide crystal surface, followed by rotation of the assembly, thus forming a compact mass. Finally, the spectrum was scanned and recorded [81].

### 4.7. GC-MS Analysis

GC-MS analysis of the *n*-butanol, ethyl acetate, and *n*-hexane fractions of the *B. indica* extract was performed by following the previously described method [82]. The samples were analyzed using a Thermo Scientific (DSQI) GC (Waltham, MA, USA) attached to the NIST11.L library. The gas was equipped with a TR-5MS capillary column with a length of 30 M, film thickness of 0.25 µm, and an internal diameter of 0.25 mm. Helium was used as the carrier gas with a 1 mL/min real flow rate. The GC-MS spectral lines were detected by the ionization energy method with an ionization energy of 70 electron volt and a 0.2 s scan time with a ranging fragment from 40 to 600 *m*/*z*. The injector was operated in split mode with a temperature of 250 °C. The 1 µL sample volume was injected with an initial oven temperature of 50 °C and held for 2 min, then increased to 150 °C with the temperature rate of 8 °C/min, and further increased to 300 °C with the temperature rate of 15 °C/min and held for 5 min. Retention time per minute, peak area, peak height, and spectral line patterns were used to identify the components present in the sample plant materials when compared with spectral lines from the database of authenticated compounds stored in the National Institute of Standards and Technology (NIST) library.

### 4.8. Cytotoxicity Study

Cytotoxic effects were assessed by the standard MTT (3-[4,5-dimethylthiazole-2-yl]-2,5-diphenyl-tetrazolium bromide) colorimetric assay on mouse fibroblast 3T3 cells. Cytotoxic effects were measured as concentration causing 50% cell growth inhibition (IC_50_) [83]. Percent inhibition was calculated by using the following formula:

(6) $$\%inhibition=100-\frac{\left(mean\;of\;O.D\;of\;test\;compound-mean\;of\;O.D\;of\;negative\;control\right)}{\left(mean\;of\;O.D\;of\;positive\;control-mean\;of\;O.D\;of\;negative\;control\right)}\times 100$$

### 4.9. Acute Toxicity Study

The acute toxicity study was performed on mice with a weight of 28 ± 4 g according to OECD guidelines test no. 425 [84]. A limit test was used for the toxicity study. Animals were housed under standard conditions and fed according to the mentioned guidelines. Initially, 2000 mg/kg of the extract was administered to a single mouse and the animal was closely monitored for the first 30 min, then for 4 h, and 24 h. After the survival of the first mouse, four additional mice were treated with the same dose and monitored accordingly. After that, the monitoring of all five mice was continued for 14 days, during which any signs of toxicity and altered behavior were observed.

### 4.10. Antibacterial Activity

The antibacterial activity of the *B. indica* crude extract, its *n*-hexane, dichloromethane, ethyl acetate, *n*-butanol, and aqueous fractions, and positive control (ceftriaxone) was assessed against Gram-positive bacteria (i.e., *Bacillus subtilis* (ATCC 6633), *Staphylococcus aureus* (ATCC 6538)) and Gram-negative bacteria including *Escherichia coli* (ATCC 10536), *Salmonella typhi* (ATCC 19430), and *Pseudomonas aeruginosa* (ATCC 9027), as described previously with minor modifications [85]. Ceftriaxone was used as a standard due to its broad spectrum antibacterial activity against both Gram-positive and Gram-negative bacteria. All of the apparatus and solutions used were sterilized, and the experiment was carried out in triplicate. Antibacterial potential was measured by the following methods.

#### 4.10.1. Zone of Inhibition by Disc Diffusion Method

Solutions of different concentrations (10, 30, and 100 mg/mL) of the *B. indica* crude extract and its *n*-hexane, dichloromethane, ethyl acetate, *n*-butanol, and aqueous fractions were prepared to assess the zone of inhibition. Discs of 5 mm were cut from filter paper and soaked in different concentrations of extracts or solvent (control) and dried at room temperature. Bacterial cultures of all bacterial strains were prepared in nutrient broth, incubated for 24 h at 37 °C, and diluted with a normal saline concentration equivalent to the 0.5 McFarland standard. Muller–Hinton agar was dissolved in distilled water, heated, and sterilized by autoclaving. Sterilized agar was poured into a Petri dish, marked into four equal parts, in a laminar airflow hood near a flame burner and was allowed to solidify. Bacterial culture (100 µL) of different strains was spread over agar, and discs soaked in different concentrations of the extract and positive control ceftriaxone (1 mg/mL) were placed. Petri dishes were incubated for 24 h at 37 °C and the zone of inhibition was measured.

#### 4.10.2. Minimum Inhibitory Concentration

The MIC of the extracts was measured using the 2-fold broth micro-dilution method in nutrient broth. Sterile 96-well plates were used for testing and dilutions ranging from 250 to 0.488 mg/mL were prepared by adding 100 µL of the extract concentration and 100 µL of nutrient broth. Negative control wells contained only 100 µL of nutrient broth. Stock solutions of the *B. indica* fractions were prepared in 10% DMSO. The effect of DMSO was ruled out by keeping one lane for the solvent without any extract. Similar dilutions of the standard drug ceftriaxone (dissolved in sterile distilled water) were prepared. Fresh bacterial inoculum, prepared from 24 h old culture, was standardized with 0.5 McFarland and 5 µL was added in each well. After 24 h of incubation at 37 °C, the clarity of the wells was observed to assess the MICs and the concentration of the wells was noted until no bacterial growth was seen [86].

### 4.11. Molecular Docking

Molecular docking was performed by AutoDock Vina (version 4.2) software to analyze the binding interactions of different compounds, obtained from GC-MS data, within the active pockets of the DNA gyrases of the targeted bacteria. The study was carried out using the 3D crystal structure of the DNA gyrase subunit B of various bacteria, retrieved from the Protein Data Bank (www.rcsb.com) with PDB ID: 4DDQ for *B. subtilis*, 1KZN for *E. coli*, 7PTF for *P. aeruginosa*, 6FM4 for *S. aureus*, and 5ZTJ for *S. typhi* [87]. Before the docking analysis, the targeted DNA gyrase structure was prepared by using MGL tools, where the heteroatoms and water molecules were removed, afterward, polar hydrogen and Kollman charges were added and the structure was rendered for missing amino acid residues [88]. The energy minimized 3D structures of all compounds were drawn using ChemDraw (Ultra 12.0) 3D [89]. The docking protocol was validated by first separating the co-crystal ligand from the active pocket of the complex, and then re-docking was performed to validate its accuracy [90]. The force field MMFF94x with an RMSD gradient of >0.01 kcal·mol^−1^Å^−1^ was used to minimize the energy of the selected compounds [91]. All compounds were docked within the active pocket of the DNA gyrase subunit B using AutoDock’s default genetic algorithm as the scoring function. The dimensions of the grid box were set as (x; −41.857501 y; −12.318299; z; −8.245484 for 4DDQ, x; 19.537493, y; 19.165566, z; 43.283299 for 1KZN, x; −2.468462 y; −2.933290 z; 38.392610 for 7PTF, x; −42.133240 y; −3.919944 z; 22.992590 for 6FM4 and x; 26.379112, y; 22.904972, z; 22.022154 for 5ZTJ). About 100 different configurations of the docking complexes were generated for the compounds with the active pocket of DNA gyrase subunit B. Finally, the most stable configuration possessing high affinity was selected for the further analysis and development of 2D and 3D models to understand the binding interactions of the compound within the active site of proteins [92]. For a more comprehensive understanding of the binding affinity, we also calculated and presented the binding energies of various other compounds. These binding energies can provide insights into the strength and stability of the binding interactions between these compounds and the DNA gyrase. Moreover, the results were compared with the docking of the positive control and the procedure was validated via the re-docking procedure.

### 4.12. Statistical Analysis

The results were calculated by linear regression using GraphPad Prism 6 and the results were presented as the mean ± SEM.

## 5. Conclusions

Our work has set forth pharmacognostic standards for the correct identification and authentication required for the quality control of *Bassia indica* (Wight) A.J. Scott. The results showed that the plant is rich in minerals and nutrients. Chemical characterization of the extract showed the presence of important bioactive phytochemicals like carbohydrates, glycosides, phenols, saponins, and sterols. GC-MS analysis identified several phytochemicals of pharmacological importance. *B. indica* showed good antibacterial activity against both Gram-positive and Gram-negative bacteria. Molecular docking studies described several phytochemicals with good binding interaction with DNA gyrase, which may suggest its antibacterial mechanism.

## Figures and Tables

**Table 1:** Extractive value of *B. indica.*

Solvent	Value (%)
Distilled water	10.5
*n*-Butanol	3
Ethyl acetate	2
Dichloromethane	1.5
*n*-Hexane	1

**Table 2:** Nutritional and elemental analysis of *B. indica.*

Parameter	Value
Nutritional analysis	(%)
Crude protein	12.39
Crude fat	1.10
Crude fiber	24.21
Carbohydrate	16.61
Ash value	14.52
Moisture content	31.17
Caloric value	111.43 (kcal/100 g)
Elemental content	(mg/kg)
Calcium	470
Cadmium	0.1
Chromium	0.05
Copper	34.5
Iron	161
Magnesium	55.6
Manganese	5.5

**Table 3:** Fluorescence analysis of the dried powder of the *B. indica* aerial parts.

Reagents	Daylight	UV 254 nm
Powder as such	Greenish brown	Brown
Methanol	Brown	Dark brown
Ethyl acetate	Light green	Dark green
Dichloromethane	Dark green	Blue
Chloroform	Brown	Dark brown
*n*-Butanol	Dark brown	Blue
*n*-Hexane	Dark green	Blackish green
50% HNO_3_	Green	Pink
Picric acids	Yellowish	Brown
50% HCl	Green	Light pink
Conc. H_2_SO_4_	Black	Dark brown
1% NaOH	Green	Dark green
Iodine	Light brown	Blue
5% FeCl_3_	Yellow	Dark brown

**Table 4:** Rf values of the *B. indica* extracts in different mobile phases.

Extract	Solvent	Ratio	Rf Value
*n*-Hexane	H:E.A	1:1	0.34, 0.55, 0.67, 0.85
	H:E.A	3:1	0.15
Dichloromethane	CH:M	4:1	0.50, 0.62, 0.70, 0.90
	H:E.A	1:1	0.28, 0.52
Ethyl acetate	H:E.A	1:1	0.544, 0.68, 0.85
	CH:M	4:1	0.11, 0.26, 0.34
*n*-Butanol	CH:M	4:1	0.10, 0.16, 0.51, 0.63, 0.85
	H:E.A	1:1	0.23, 0.50, 0.59, 0.66
Aqueous	CH:M	4:1	0.88
Crude	E.A:B:AA:W	16:2:1:1	0.08, 0.94

H: *n*-hexane, E.A: ethyl acetate, CH: chloroform, M: methanol, B: *n*-butanol, AA: acetic acid, W: water.

**Table 5:** Quantitative analysis of phenols, flavonoids, and tannins in the *B. indica* extracts.

Fraction	TPC (mg TAEg^−1^)	TTC (mg TAEg^−1^)	TFC (mg QEg^−1^)
*n*-Hexane	36.00 ± 2.9	3.99 ± 0.48	24.089 ± 3.952
Dichloromethane	303.48 ± 25.28	190.95 ± 37.26	106.223 ± 3.437
Ethyl acetate	419.10 ± 11.76	249.60 ± 10.50	161.790 ± 1.375
*n*-Butanol	296.07 ± 10.14	156.97 ± 29.47	109.658 ± 9.107
Aqueous	169.86 ± 12.08	104.17 ± 26.93	47.973 ± 0.344
Crude	214.27 ± 14.98	107.50 ± 30.27	86.943 ± 5.670

TPC: total phenolic content, TTC: total tannin content, TFC: total flavonoid content.

**Table 6:** Functional groups and their wave numbers found in the FTIR of the *B. indica* powder.

Wave Number (cm^−1^)	Intensity of Estimation	Group or Functional Group Class
2914.8	S	O-H stretching (alcohol)
	M	C-H stretching (alkane)
2847.7	M	C-H stretching (alkane)
1735.1	W	C-H bending (aromatic compound)
1638.2	M	C=C stretching (conjugated alkene)
1364.2	M	O-H bending (alcohol)
1313.9	S	S=O stretching (sulfone)
	M	O-H bending (phenol)
1239.3	S	C-O stretching (alkyl aryl ether)
	M	C-N stretching (amine)
1099.6	S	C-O stretching (secondary alcohol)
1019.4	S	C=C bending (alkene)
896.4	M	C=C bend (alkenes like vanylidine)
777.1	S	C-Cl stretching (halo compound)

S: strong, M: medium, W: weak.

**Table 7:** Compounds identified in the GC-MS analysis of the *n*-butanol fraction of *B. indica.*

Sr. NO	RT	Area	Compound	Molecular Formula	Molecular Weight
**NB1**	**3.92**	**1.10**	**6-Methoxy-2-phenyl-hexahydropyrano[2,3-b][1,3]dioxine-7,8 diol**	**C_14_H_18_O_6_**	**282.29**
NB2	4.28	0.17	2,5-Dihydro-5-methoxy-2-furanone	C_5_H_6_O_3_	114.10
NB3	7.28	0.9	9-Octadecenoic acid, (2-phenyl-1,3-dioxolan-4-yl)methyl ester, cis	C_28_H_44_O_4_	444
NB4	9.62	0.15	Acetamide, N-methyl-N-[4-[2-acetoxymethyl-1-pyrrolidyl]-2-butynyl]-	C_14_H_22_N_2_O_3_	266.33
NB5	10.50	1.28	Tetradecane	C_14_H_30_	198.38
NB6	12.01	0.49	Dodecane, 5,8-diethyl-	C_16_H_34_	226.44
**NB7**	**12.31**	**0.20**	**2H-indeno[1,2-b]furan-2-one, 3,3a,4,5,6,7,8,8b-octahydro-8,8-dimethyl**	**C_13_H_18_O_2_**	**206.28**
NB8	12.92	0.74	Pentanoic acid, 2,2,4-trimethyl-3-carboxyisopropyl, isobutyl ester	C_16_H_30_O_4_	286.40
NB9	13.43	5.20	Hexadecane	C_16_H_34_	226.44
NB10	13.92	3.16	N-Acetyl-4-phenylbutylamine	C_12_H_17_NO	191
NB11	15.20	0.77	Myristyl monoethoxylate	C_16_H_34_O_2_	258.44
NB12	15.92	0.15	2(3H)-Furanone, dihydro-5-tetradecyl	C_18_H_34_O_2_	282.46
NB13	16.18	0.16	trans-1,2-diaminocyclohexane-N,N,N,N-tetraacetic acid	C_14_H_22_N_2_O_8_	346
NB14	16.51	0.14	17-Pentatriacontene	C_35_H_70_	490.93
NB15	16.81	3.39	Bacteriochlorophyll-c-stearyl	C_52_H_72_MgN_4_O_4_	841.5
NB16	17.20	0.77	2- Nonadecanone 2,4- dinitrophenylhydrazine	C_25_H_42_N_4_O_4_	462
NB17	17.83	2.38	1-Docosanol	C_22_H_46_O	326.6
NB18	18.22	0.68	7,9-Di-tert-butyl-1-oxaspiro(4,5)deca-6,9-diene-2,8-dione	C_17_H_24_O_3_	276.37
NB19	18.79	8.08	Hexadecanoic acid, 1-(hydroxymethyl)-1,2-ethanediyl ester	C_35_H_68_O_5_	568.91
NB20	20.56	0.11	3-Pyridinecarboxylic acid, 2,7,10-tris(acetyloxy)-1,1a,2,3,4,6,7,10, 11,11a-decahydro-1,1,3,6,9-pentamethyl-4-oxo-4a,7a-epoxy-5H-cyclopenta[a]cyclo propa [f]cycloundecen-11-yl ester, [1aR(1aR*,2R*,3S*,4aR*,6S*,7S*,7aS*,8E,10R*,11R*,11aS*)]-	C_32_H_39_NO_10_	597.25
NB21	21.05	1.77	Dodecanecarboxamide, N-[2-(3-indolyl)ethyl]-	C_22_H_34_N_2_O	342
NB22	21.40	1.86	Erucic acid	C_22_H_42_O_2_	338.57
NB23	21.99	0.18	18,19-Secoyohimban-19-oic acid, 16,17,20,21-tetradehydro-16-(hydroxy methyl)-, methyl ester, (15α,16E)-	C_21_H_24_N_2_O_3_	352
NB24	22.31	48.18	1,2-Benzenedicarboxylic acid, diisooctyl ester	C_24_H_38_O_4_	390
NB25	23.62	2.08	Squalene	C_30_H_50_	410
**NB26**	**23.92**	**0.22**	**24,25-Dihydroxycholecalciferol**	**C_27_H_44_O_3_**	**416**
**NB27**	**24.29**	**0.76**	**Corynan-17-ol, 18,19-didehydro-10-methoxy-, acetate (ester)**	**C_22_H_28_N_2_O_3_**	**368**
NB28	25.0	0.54	9,12,15-Octadecatrienoic acid, 2,3-bis[(trimethylsilyl)oxy]propyl ester, (Z,Z,Z)-	C_27_H_52_O_4_Si_2_	496
NB29	26.04	5.91	Azafrin	C_27_H_38_O_4_	426
NB30	27.8	0.13	Butanoic acid,1a,2,5,5a,6,9,10,10a-octahydro-5,5a-di hydroxy-4-(hydroxymethyl)-1,1,7,9-tetra methyl-11-oxo-1H-2,8a-methanocyclop enta[a]cyclopropa[e]cyclodecen-6-yl ester	C_24_H_34_O6	418

Compounds written in bold characters were used for molecular docking; NB: *n*-butanol fraction, RT: retention time, *: describe spatial configuration.

**Table 8:** Compounds identified in the GC-MS analysis of the ethyl acetate fraction of *B. indica.*

Sr. No	RT	Area	Compound	Molecular Formula	Molecular Weight
EA1	12.0	0.15	Octadecane, 3-ethyl-5-(2-ethylbutyl)-	C_26_H_54_	366.70
EA2	12.80	0.59	Illudol	C_15_H_24_O_3_	252.35
EA3	13.45	3.16	Hexadecane	C_16_H_34_	226.44
EA4	14.29	0.95	2-Naphthalenemethanol, decahydro-α,α,4a-trimethyl-8-methylene-, [2R-(2α,4aα,8aα)]-	C_15_H_26_O	222.36
EA5	15.02	1.58	Isoaromadendrene epoxide	C_15_H_24_O	220.35
EA6	15.53	0.40	Alpha-Santanol acetate	C_17_H_26_O_2_	262.38
EA7	16.10	0.12	17-Pentatriacontene	C_35_H_70_	490.93
EA8	16.44	0.46	Acetate, [6-(acetyloxy)-5,5,8a-trimethyl-2-methyleneperhydro-1-naphthalenyl]methyl ester	C_19_H_30_O_4_	322.00
EA9	17.06	12.11	Bicyclo[2.2.2]octa-2,5-diene, 1,2,3,6-tetramethyl-	C_12_H_18_	162.27
**EA10**	**17.61**	**2.97**	**5-Hydroxymethyl-1,1,4a-trimethyl-6-methylenedecahydronaphthalen-2-ol**	**C_15_H_26_O_2_**	**238.37**
EA11	17.89	3.36	Benzene, hexamethyl-	C_12_H_18_	162.27
**EA12**	**19.01**	**1.05**	**Gitoxigenin**	**C_23_H_34_O_5_**	**390.50**
EA13	19.56	55.80	4,4-Dimethyladamantan-2-ol	C_12_H_20_O	180.29
EA14	20.17	0.11	Androst-4-en-3-one, 9-fluoro-11,17-dihydroxy-17-methyl-, (11α,17α)-	C_20_H_29_FO_3_	336.40
**EA15**	**20.62**	**1.65**	**Methyl 3-(acetyloxy)-7,12-dioxocholan-24-oate**	**C_27_H_40_O_6_**	**460.00**
**EA16**	**21.01**	**0.73**	**Agathic acid**	**C_20_H_30_O_4_**	**334.40**
**EA17**	**22.27**	**5.58**	**1,2-Benzenedicarboxylic acid, diisooctyl ester**	**C_24_H_38_O_4_**	**390.00**
**EA18**	**22.98**	**0.48**	**2,7-Diphenyl-1,6-dioxopyridazino[4,5:2′,3′]pyrrolo [4′,5′-d]pyridazine**	**C_20_H_13_N_5_O_2_**	**355.30**
EA19	23.98	0.67	9-Octadecene, 1-[2-(octadecyloxy)ethoxy]-	C_38_H_76_O_2_	565.00
**EA20**	**24.43**	**0.17**	5aH-3a,12-methano-1H-cyclopropa[5′,6′]cyclodeca [1′,2′:1,5]cyclopenta[1,2-d][1,3]dioxol-13-one, 1a,2,3,9,12,12a-hexahydro-9-hydroxy-10 -(hydroxymethyl)-1,1,3,5,7,7-hexamethyl-, [1aR-(1aα,3α,3aα,5aα,8aR*,9α,12α,12aα)]-	**C_23_H_32_O_5_**	**388.50**
**EA21**	**24.43**	**0.17**	**Betulin**	**C_30_H_50_O_2_**	**442.70**
**EA22**	**24.72**	**0.13**	**24,25-Dihydroxycholecalciferol**	**C_27_H_44_O_3_**	**416.00**
**EA23**	**25.86**	**2.47**	**Stigmasterol**	**C_29_H_48_O**	**412.70**
EA24	26.63	0.63	2,2,3,5,5-Pentachloro-7,7-bis(chloromethyl)-1 (dichloromethyl)bicyclo[2.2.1]heptane	C_10_H_9_Cl_9_	448.20
EA25	27.30	0.68	9,12,15-Octadecatrienoic acid, 2,3-bis[(trimethylsilyl)oxy]propyl ester, (Z,Z,Z)-	C_27_H_52_O_4_Si_2_	496.90

Compounds written in bold characters were used for molecular docking; EA: ethyl acetate fraction, RT: retention time, *: describe spatial configuration.

**Table 9:** Compounds identified in the GC-MS analysis of the *n*-hexane fraction of *B. indica.*

Sr. No	RT	Area	Compound	Molecular Formula	Molecular Weight
**NH1**	**4.25**	**0.54**	**5-Methylfurfural**	**C_6_H_6_O_2_**	**110.11**
**NH2**	**8.13**	**16.94**	**2-Methoxy-4-vinylphenol**	**C_9_H_10_O_2_**	**150.17**
**NH3**	**10.16**	**2.59**	**4-(2,6,6-Trimethylcyclohexa-1,3-dienyl)but-3-en-2-one**	**C_13_H_18_O**	**190.28**
**NH4**	**11.31**	**2.99**	**Megastigmatrienone**	**C_13_H_18_O**	**190.28**
NH5	12.23	0.65	Ar-turmerone	C_15_H_20_O	216.31
NH6	15.06	1.58	Hexadecanoic acid, methyl ester	C_17_H_34_O_2_	270.45
NH7	15.25	4.41	*n*-Hexadeconic acid	C_16_H_32_O_2_	256.42
NH8	15.91	0.28	Hexadeconic acid, ethyl ester	C_18_H_36_O_2_	284.47
NH9	17.26	1.09	9,12-Octadecadienoic acid, methyl ester	C_19_H_34_O_2_	294.47
NH10	17.35	2.91	9,12,15-Octadecatrienoic acid	C_18_H_30_O_2_	278.40
NH11	17.96	8.58	9,12,15-Octadecatrien-1-ol, (Z,Z,Z)-	C_18_H_32_O	264.44
NH12	18.18	0.59	Linoleic acid ethyl ester	C_20_H_36_O_2_	308.49
NH13	18.26	0.89	Ethyl Oleate	C_20_H_38_O_2_	310.51
**NH14**	**23.69**	**0.49**	**1,2-Benzenedicarboxylic acid, monopentyl ester**	**C_13_H_16_O_4_**	**236.26**

Compounds written in bold characters were used for molecular docking; RT: retention time, NH: *n*-hexane fraction.

**Table 10:** Zone of inhibition of the *B. indica* extracts.

Fractions	Conc.	*B. subtilis*	*S. aureus*	*P. aeruginosa*	*S. typhi*	*E. coli*
	(mg/mL)	Mm				
*n*-Hexane	10	6	10.5	8.5	6	10.5
	30	12	9	9	8.5	15
	100	11.5	14	9.5	9	12.5
Dichloromethane	10	9	7.5	7.5	4.5	4
	30	9.5	11	8	9	13
	100	13	12	11.5	10.5	13
Ethyl acetate	10	10	9.5	8.5	8	5
	30	15.5	11.5	9	9.5	8.5
	100	13	14.5	10.5	9.5	27
*n*-Butanol	10	9	8	5	9	9.5
	30	10.5	9	7.5	4.5	11.5
	100	13	10.5	11.5	11	14
Aqueous	10	7.5	9.5	8.5	9.5	9.5
	30	8	13.5	7	7	5.5
	100	nil	9.5	7.5	6.5	9
Crude	10	11	8.5	7	5	8.5
	30	11	9	4.5	8	10.5
	100	14.5	11	9	8	12.5
Ceftriaxone	1	15	16	7.5	11	15.5

**Table 11:** Minimum inhibitory concentration of the *B. indica* extracts.

Fractions	*B. subtilis*	*S. aureus*	*P. aeruginosa*	*S. typhi*	*E. coli*
	mg/mL				
*n*-Hexane	>100	>100	>100	>100	Nil
Dichloromethane	3.91	7.81	3.91	62.5	31.25
Ethyl acetate	1.95	3.91	3.91	7.81	3.91
*n*-Butanol	3.91	31.25	3.91	31.25	3.91
Aqueous	31.25	62.5	31.25	62.5	62.5
Crude	31.25	31.25	31.25	62.5	62.5
Ceftriaxone	1.95	3.91	0.98	0.98	7.81

**Table 12:** Molecular docking scores of the *n*-butanol, ethyl acetate, and *n*-hexane fractions with the targeted proteins.

Compounds	*B. subtilis*	*E. coli*	*P. aeruginosa*	*S. aureus*	*S. typhi*
	kcal/mol				
NB1	−6.6	−7.3	−8.0	−7.5	−7.5
NB7	−5.7	−7.4	−7.4	−7.0	−6.4
NB26	−7.2	−6.6	−8.4	−7.8	−7.3
NB27	−6.3	−6.5	−7.8	−7.3	−7.5
EA10	−5.7	−5.4	−6.3	−7.2	−6.2
EA12	−7.5	−7.5	−8.4	−7.8	−8.6
EA15	−6.6	−7.1	−8.8	−8.0	−8.3
EA16	−6.6	−7.1	−7.6	−7.3	−7.5
EA17	−4.2	−4.4	−5.3	−6.4	−5.6
EA18	−8.2	−8.5	−9.1	−9.2	−9.4
EA20	−7.5	−7.4	−8.7	−8.1	−9.3
EA21	−7.4	−6.6	−7.5	−7.7	−8.6
EA22	−6.8	−6.0	−7.3	−7.4	−7.0
EA23	−7.5	−7.1	−8.3	−7.8	−8.1
NH1	−4.2	−4.5	−4.8	−4.6	−4.2
NH2	−4.8	−5.5	−5.9	−5.5	−5.0
NH3	−5.5	−5.9	−6.4	−6.4	−5.5
NH4	−5.8	−6.1	−6.8	−6.7	−5.8
NH14	−5.0	−6.2	−6.4	−5.8	−5.3

## Data Availability

Data can be provided upon reasonable request.

## Supplementary Materials

The following supporting information can be downloaded at: https://www.mdpi.com/article/10.3390/plants13131753/s1, Figure S1. The predicted 3D(A) and 2D(B) binding mode of the co-crystal ligand against the DNA gyrase subunit B of *B. subtilis*; Figure S2. The predicted 3D(A) and 2D(B) binding mode of ceftriaxone against the DNA gyrase subunit B of *B. subtilis*; Figure S3. The predicted 3D(A) and 2D(B) binding mode of the co-crystal ligand against the DNA gyrase subunit B of *E. coli*; Figure S4. The predicted 3D(A) and 2D(B) binding mode of ceftriaxone against the DNA gyrase subunit B of *E. coli*; Figure S5. The predicted 3D(A) and 2D(B) binding mode of the co-crystal ligand against the DNA gyrase subunit B of *P. aeruginosa*; Figure S6. The predicted 3D(A) and 2D(B) binding mode of ceftriaxone against the DNA gyrase subunit B of *P. aeruginosa*; Figure S7. The predicted 3D(A) and 2D(B) binding mode of the co-crystal ligand against the DNA gyrase subunit B of *S. aureus*; Figure S8. The predicted 3D(A) and 2D(B) binding mode of ceftriaxone against the DNA gyrase subunit B of *S. aureus*; Figure S9. The predicted 3D(A) and 2D(B) binding mode of ceftriaxone against the DNA gyrase subunit B of *S. typhi*; Table S1. Molecular docking scores of the co-crystal ligands with the targeted proteins.

## References

[1] WHO (1993). Guidelines on the Conservation of Medicinal Plants.

[2] Chen S.-L., Yu H., Luo H.-M., Wu Q., Li C.-F., Steinmetz A. (2016). Conservation and sustainable use of medicinal plants: Problems, progress, and prospects. Chin. Med..

[3] Ekor M. (2014). The growing use of herbal medicines: Issues relating to adverse reactions and challenges in monitoring safety. Front. Pharmacol..

[4] Puneshwar K., Hany A.E.-S. (2021). Controversy, adulteration and substitution: Burning problems in ayurveda practices. Natural Medicinal Plants.

[5] Khan S.A., Barkatullah, Ibrar M., Ullah S. (2019). Microscopic investigations and pharmacognostic techniques used for the standardization of leaf of *Rhus succedanea* var. Himalaica JD Hook. Microsc. Res. Tech..

[6] Ichim M.C. (2019). The DNA-based authentication of commercial herbal products reveals their globally widespread adulteration. Front. Pharmacol..

[7] Bokhari S.W.A., Sharif H., Gilani S.M.U., Ali S.T., Ahmed S., Siddiqui M.U.A., Hasan M.M. (2022). Pharmacognostic and phytochemical study of the flowers of *Cordia sebestena* L. Pak. J. Pharm. Sci.

[8] Uza N.U., Dastagir G. (2022). Microscopic and pharmacognostic standardization of *Astragalus scorpiurus* Bunge. Microsc. Res. Tech..

[9] WFO (2023). *Bassia indica* (Wight) A.J. Scott. https://wfoplantlist.org/taxon/wfo-0000561151-2024-06?page=1.

[10] Grabowska K., Buzdygan W., Galanty A., Wróbel-Biedrawa D., Sobolewska D., Podolak I. (2023). Current knowledge on genus *Bassia* All: A comprehensive review on traditional use, phytochemistry, pharmacological activity, and nonmedical applications. Phytochem. Rev..

[11] Youssef S. (2013). Medicinal and non-medicinal uses of some plants found in the middle region of Saudi Arabia. J. Med. Plant Res..

[12] El-Gendy Z.A., Taher R.F., Elgamal A.M., Serag A., Hassan A., Jaleel G.A.A., Farag M.A., Elshamy A.I. (2023). Metabolites profiling and bioassays reveal *Bassia indica* ethanol extract protective effect against stomach ulcers development via HMGB1/TLR-4/NF-κB pathway. Antioxidants.

[13] Qureshi M.Z., Javed S., Javaid A., Al-Taie A.H. (2020). Identification of antimicrobial compounds from n-hexane stem extract of *Kochia indica* by GC-MS analysis. Mycopath.

[14] Mohammed M., Heikal E.A., Ellessy F.M., Aboushousha T., Ghareeb M.A. (2023). Comprehensive chemical profiling of *Bassia indica* Wight. aerial parts extract using UPLC-ESI–MS/MS, and its antiparasitic activity in *Trichinella spiralis* infected mice: In silico supported in vivo study. BMC Complement. Med. Ther..

[15] Othman A., Amen Y., Inoue Y., Shimizu K. (2022). Phytochemical analysis, anti-inflammatory, and anticancer activities of the halophyte herb *Bassia indica*. Nat. Prod. Commun..

[16] Bouaziz M., Dhouib A., Loukil S., Boukhris M., Sayadi S. (2009). Polyphenols content, antioxidant and antimicrobial activities of extracts of some wild plants collected from the south of Tunisia. Afr. J. Biotechnol..

[17] Bibi H., Hussain M., Jan G., Shah G., Khan S., Ullah I. (2021). Phytochemical analysis and antimicrobial activities of *Kochia indica* (Wight), plant growing in District Karak Khyber Puhktunkhuwa. Pure Appl. Biol..

[18] Javed S., Javaid A., Qureshi M.Z. (2018). Antifungal phytocomponents in n-butanol fraction of leaf extract of *Kochia indica* Wight. Int. J. Biol. Biotechnol..

[19] Othman A., Sayed A.M., Amen Y., Shimizu K. (2022). Possible neuroprotective effects of amide alkaloids from *Bassia indica* and *Agathophora alopecuroides*: In vitro and in silico investigations. RSC Adv..

[20] Jubair N., Rajagopal M., Chinnappan S., Abdullah N.B., Fatima A. (2021). Review on the antibacterial mechanism of plant-derived compounds against multidrug-resistant bacteria (MDR). Evid. Based Complement. Altern. Med..

[21] Kim O.K., Barrett J.F., Ohemeng K. (2001). Advances in DNA gyrase inhibitors. Expert Opin. Investig. Drug.

[22] Zhang M., Wang C., Zhang R., Chen Y., Zhang C., Heidi H., Li M. (2021). Comparison of the guidelines on good agricultural and collection practices in herbal medicine of the European Union, China, the WHO, and the United States of America. Pharmacol. Res..

[23] WHO (1998). Quality Control Methods for Medicinal Plant Materials.

[24] El Shereef A.A. (2016). Kochia plant as potential forage for ruminants under desert conditions. Annu. Res. Rev. Biol..

[25] Turki Z., El-Shayeb F., Shehata F. (2006). Taxonomic studies in the camphorosmeae (chenopodiaceae) in Egypt. Flora Mediterr..

[26] Zaman S., Barkatulllah, Zahoor M., Wadood Ali Shah S., Ullah Z., Ullah R., Alotaibi A. (2022). Pharmacognostic evaluation of *Artemisia maritima* L. a highly medicinal specie of genus Artemisia. Saudi J. Biol. Sci..

[27] Adu O.T., Naidoo Y., Adu T.S., Sivaram V., Dewir Y.H. (2022). Micromorphology and histology of the secretory apparatus of *Diospyros villosa* (L.) de winter leaves and stem bark. Plants.

[28] Hameed A., Ghani N., Mughal T.A., Abbas M., Abrar A., Javed H. (2023). Pharmacognostical evaluation and physiochemical analysis of *Salsola Kali* as medicinal plant. Microsc. Res. Tech..

[29] Yusufoglu H.S. (2015). Pharmacognostic and wound healing studies of the leaves of *Bassia eriophora* (family: Chenopodiaceae) on albino rats. Annu. Res. Rev. Biol..

[30] Ukwubile C.A., Ikpefan E.O., Dibal M.Y., Umeano V.A., Menkiti D.N., Kaosi C.C., Paul S., Famurewa A.C., Nettey H., Yerima T.S. (2023). Pharmacognostic profiles, evaluation of analgesic, anti-inflammatory and anticonvulsant activities of *Newbouldia laevis* (P. Beauv.) Seem. ex Bureau leaf and root extracts in Wistar rats. J. Ethnopharmacol..

[31] Sree P.R., Thoppil J. (2021). Comparative seed morphology, pharmacognostic, phytochemical, and antioxidant potential of *Memecylon* l. Fruits. Turk. J. Pharm. Sci..

[32] Dastagir G., Hussain F., Uza N.U. (2023). Anatomical and histochemical characterization of some highly medicinal plants as a tool for quality control. Microsc. Res. Tech..

[33] Jones P.W., Chin Y.-W., Kinghorn D.A. (2006). The role of pharmacognosy in modern medicine and pharmacy. Curr. Drug Targets.

[34] Pirintsos S., Panagiotopoulos A., Bariotakis M., Daskalakis V., Lionis C., Sourvinos G., Karakasiliotis I., Kampa M., Castanas E. (2022). From traditional ethnopharmacology to modern natural drug discovery: A methodology discussion and specific examples. Molecules.

[35] Balami S., Sharma A., Karn R. (2019). Significance of nutritional value of fish for human health. Malays. J. Halal Res. J..

[36] Radha, Kumar M. (2021). Evaluation of nutritional, phytochemical, and mineral composition of selected medicinal plants for therapeutic uses from cold desert of Western Himalaya. Plants.

[37] Ahmad A., Husain A., Mujeeb M., Khan S.A., Alhadrami H.A.A., Bhandari A. (2015). Quantification of total phenol, flavonoid content and pharmacognostical evaluation including HPTLC fingerprinting for the standardization of *Piper nigrum* Linn fruits. Asian Pac. J. Trop. Biomed..

[38] Nafea E. (2017). Nutritive values of some wetland plants in the Deltaic Mediterranean coast of Egypt. Egypt. J. Bot..

[39] Mensah E., Kyei-Baffour N., Ofori E., Obeng G., Yanful E.K. (2009). Influence of Human Activities and Land Use on Heavy Metal Concentrations in Irrigated Vegetables in Ghana and Their Health Implications.

[40] Mirzaei F. (2012). Minerals profile of forages for grazing ruminants in Pakistan. Open J. Anim. Sci..

[41] El Shaer H.M. (2010). Halophytes and salt-tolerant plants as potential forage for ruminants in the Near East region. Small Rumin. Res..

[42] Younus M., Hasan M.M., Rehman M.S., Abbas K., Sarwar G. (2019). Report: Pharmacognostic and physicochemical screening of *Euphorbia nivulia* Buch.-Ham. Pak. J. Pharm. Sci.

[43] Sajid-Ur-Rehman M., Ishtiaq S., Khalil-Ur-Rehman M., Bauer R. (2021). Pharmacognostic screening, physico-chemical and cytotoxic potential of *Sesuvium sesuvioides* (Fenzyl) Verdc. Pak. J. Pharm. Sci.

[44] Ahmed F.A., Baraka D.M., Donia A.E.R.M., Mostafa R.M., Morsy Z.M. (2022). Phytochemical investigation, HPLC analysis and antimicrobial activity of some plants from chenopodiaceae family. Egypt. Acad. J. Biol..

[45] Vasavilbazo-Saucedo A., Almaraz-Abarca N., González-Ocampo H.A., Ávila-Reyes J.A., González-Valdez L.S., Luna-González A., Delgado-Alvarado E.A., Torres-Ricario R. (2018). Phytochemical characterization and antioxidant properties of the wild edible acerola *Malpighia umbellata* Rose. CyTA J. Food.

[46] González-Ocampo H.A., Martínez-Álvarez I.G., Jaramillo-Flores M.E., Luna-González A. (2022). Comparison of phenolic and flavonoid content and antioxidant and chelating activities of *Rhizophora mangle* in different anthropogenically-polluted coastal lagoons. Front. Mar. Sci..

[47] Bouarab-Chibane L., Forquet V., Lantéri P., Clément Y., Léonard-Akkari L., Oulahal N., Degraeve P., Bordes C. (2019). Antibacterial properties of polyphenols: Characterization and QSAR (quantitative structure–activity relationship) models. Front. Microbiol..

[48] Maugeri A., Lombardo G.E., Cirmi S., Süntar I., Barreca D. (2022). Pharmacology and toxicology of tannins. Arch. Toxicol..

[49] Elekofehinti O.O., Iwaloye O. (2021). Saponins in cancer treatment: Current progress and future prospects. Pathophysiology.

[50] Maulidya V., Hasanah A.N. (2023). Quality control and authentication of black betel leaf extract (*Piper acre* Blume) from East Kalimantan as an antimicrobial agent using a combination of high-performance liquid chromatography and chemometric fourier transform infrared. Molecules.

[51] Khan I.H., Javaid A. (2019). Antifungal, antibacterial and antioxidant components of ethyl acetate extract of quinoa stem. Plant Prot..

[52] Selvakumar J.N., Chandrasekaran S.D., Doss G.P.C., Kumar T.D. (2019). Inhibition of the ATPase domain of human topoisomerase IIa on HepG2 Cells by 1, 2-benzenedicarboxylic acid, mono (2-ethylhexyl) ester: Molecular docking and dynamics simulations. Curr. Cancer Drug Targets.

[53] Fadipe L.A., Haruna A., Mohammed I. (2014). Antibacterial activity of 1, 2-benzenedicarboxylic acid, dioctyl ester isolated from the ethyl acetate soluble sub-portion of the unripe fruits of *Nauclea latifolia*. Int. J. Pure Appl. Biosci..

[54] Reddy L.H., Couvreur P. (2009). Squalene: A natural triterpene for use in disease management and therapy. Adv. Drug Deliv. Rev..

[55] Henry H.L., Norman A.W., Taylor A.N., Hartenbower D.L., Coburn J.W. (1976). Biological activity of 24,25-dihydroxycholecalciferol in chicks and rats. J. Nutr..

[56] Yang S., Chou G., Li Q. (2018). Cardioprotective role of azafrin in against myocardial injury in rats via activation of the Nrf2-ARE pathway. Phytomedicine.

[57] Brintha S., Rajesh S., Renuka R., Santhanakrishnan V., Gnanam R. (2017). Phytochemical analysis and bioactivity prediction of compounds in methanolic extracts of *Curculigo orchioides* Gaertn. J. Pharmacogn. Phytochem..

[58] Anifalaje E.O., Ibok M.G. (2020). Chemical compositions and antioxidant activities of *Albizia lebbeck* L. essential oils. J. Essent. Oil-Bear. Plants.

[59] Shokrollahi N., Ho C.-L., Mohd Zainudin N.A.I., Abdul Wahab M.A.w.B., Wong M.-Y. (2023). Plant defense inducers and antioxidant metabolites produced during oil palm-*Ganoderma boninense* interaction in vitro. Chem. Afr..

[60] Gardner D.R., Panter K.E., Stegelmeier B.L. (2010). Implication of agathic acid from *Utah juniper* bark as an abortifacient compound in cattle. J. Appl. Toxicol..

[61] Hashimoto T., Rathore H., Satoh D., Griffin J.F., From A.H., Ahmed K., Fullerton D.S., Hong G. (1986). Cardiac glycosides. 6. Gitoxigenin C16 acetates, formates, methoxycarbonates, and digitoxosides. Synthesis and Na+, K+-ATPase inhibitory activities. J. Med. Chem..

[62] Kaur P., Arora S., Singh R. (2022). Isolation, characterization and biological activities of betulin from *Acacia nilotica* bark. Sci. Rep..

[63] Bakrim S., Benkhaira N., Bourais I., Benali T., Lee L.-H., El Omari N., Sheikh R.A., Goh K.W., Ming L.C., Bouyahya A. (2022). Health benefits and pharmacological properties of stigmasterol. Antioxidants.

[64] Asami E., Kitami M., Ida T., Kobayashi T., Saeki M. (2023). Anti-inflammatory activity of 2-methoxy-4-vinylphenol involves inhibition of lipopolysaccharide-induced inducible nitric oxidase synthase by heme oxygenase-1. Immunopharmacol. Immunotoxicol..

[65] Yang S., Liu J., Jiao J., Jiao L. (2020). Ar-turmerone exerts anti-proliferative and anti-inflammatory activities in HaCaT keratinocytes by inactivating hedgehog pathway. Inflammation.

[66] Shaaban M.T., Ghaly M.F., Fahmi S.M. (2021). Antibacterial activities of hexadecanoic acid methyl ester and green-synthesized silver nanoparticles against multidrug-resistant bacteria. J. Basic Microbiol..

[67] Mohammad Shafiqur R., Jaripa B., JU C., MN A. (2007). Antibacterial activity of 1, 2-benzenedicarboxylic acid, diisooctyl ester isolated from the root of *Pumbago zeylanica* linn. Hamdard Med..

[68] Shahid A., Khan D.A., Aati H.Y., Sherif A.E., Ovatlarnporn C., Hussain M., Rao H., Khan M.I., Younus M., Basit A. (2023). Chemical profiling and biological activities of *Dipterygium glaucum* Decne.: An in-vivo, in-vitro and in-silico evaluation. S. Afr. J. Bot..

[69] Aati H.Y., Anwar M., Al-Qahtani J., Al-Taweel A., Khan K.-u.-R., Aati S., Usman F., Ghalloo B.A., Asif H.M., Shirazi J.H. (2022). Phytochemical profiling, in vitro biological activities, and *in-silico* studies of *Ficus vasta* Forssk.: An unexplored plant. Antibiotics.

[70] Iqbal S.M., Mushtaq A., Jabeen Q. (2014). Antimicrobial, antioxidant and calcium channel blocking activities of *Amberboa divaricata*. Bangladesh J. Pharmacol..

[71] Abubakar A.R., Haque M. (2020). Preparation of medicinal plants: Basic extraction and fractionation procedures for experimental purposes. J. Pharm. Bioallied Sci..

[72] Nafees M., Barkatullah, Ullah S., Ikram N. (2022). Phytochemical and pharmacognostic studies of *Buddleja asiatica* leaves. Microsc. Res. Tech..

[73] Khan S.A., Barkatullah, Khan B. (2020). Anatomy, micromorphology, and physiochemical analysis of *Rhus succedanea* var. himalaica root. Microsc. Res. Tech..

[74] Farooq M., Shoaib M.H., Yousuf R.I., Qazi F., Hanif M. (2019). Development of extended release loxoprofen sodium multiparticulates using different hydrophobic polymers. Polym. Bull..

[75] WHO (2011). Quality Control Methods for Herbal Materials.

[76] Helrich K. (1990). Official Methods of Analysis of the Association of Official Analytical Chemists.

[77] Altemimi A., Watson D.G., Kinsel M., Lightfoot D.A. (2015). Simultaneous extraction, optimization, and analysis of flavonoids and polyphenols from peach and pumpkin extracts using a TLC-densitometric method. Chem. Cent. J..

[78] Masood N., Jamil Q., Aslam M.I., Masood M.I., Shirazi J.H., Jamil Q.A., Jan M.S., Alsuwayt B., Ahmad A., Alnasser S.M.A. (2023). Antioxidant, carbonic anhydrase inhibition and diuretic activity of *Leptadenia pyrotechnica* Forssk. Decne. Heliyon.

[79] Ayaz A., Jamil Q., Hussain M., Anjum F., Sarfraz A., Alqahtani T., Hussain N., Gahtani R.M., Dera A.A., Alharbi H.M. (2022). Antioxidant and gastroprotective activity of *Suaeda fruticosa* Forssk. Ex JF Gmel. Molecules.

[80] Aslam M.I., Touqeer S., Jamil Q., Masood M.I., Sarfraz A., Khan S.Y., Jan M.S., Alnasser S.M.A., Ahmad A., Aslam F. (2024). *Cenchrus ciliaris* L. ameliorates cigarette-smoke induced acute lung injury by reducing inflammation and oxidative stress. S. Afr. J. Bot..

[81] Schulz H., Baranska M. (2007). Identification and quantification of valuable plant substances by IR and Raman spectroscopy. Vib. Spectrosc..

[82] Nawaz I., Tahir A., Iqbal S.M., Anjum F., Naseem M., Aslam M.I., Hussain M., Jamil Q.A., Shirazi J.H., Jamil Q. (2023). Anti-inflammatory, anti-nociceptive and anti-pyretic activities of *Cenchrus ciliaris* L. J. Ethnopharmacol..

[83] Mosmann T. (1983). Rapid colorimetric assay for cellular growth and survival: Application to proliferation and cytotoxicity assays. J. Immunol. Methods.

[84] Saleem U., Amin S., Ahmad B., Azeem H., Anwar F., Mary S. (2017). Acute oral toxicity evaluation of aqueous ethanolic extract of Saccharum munja Roxb. roots in albino mice as per OECD 425 TG. Toxicol. Rep..

[85] Iqbal S.M., Jamil Q., Jamil N., Kashif M., Mustafa R., Jabeen Q. (2016). Antioxidant, antibacterial and gut modulating activities of *Kalanchoe laciniata*. Acta. Pol. Pharm..

[86] Konstantinovitch K.k.Y., Arsene M.M.J., Aliya M.V., Viktorovna P.I., Elena V.G., Azova M.M., Amira A.A. (2022). Assessment of antimicrobial activity of ethanolic and aqueous extracts of *Aesculus hippocastanum* L. (horse chestnut) bark against bacteria isolated from urine of patients diagnosed positive to urinary tract infections. Front. Biosci. (Schol. Ed.).

[87] Bank P.D. (1971). Protein data bank. Nat. New Biol..

[88] Goodsell D.S., Sanner M.F., Olson A.J., Forli S. (2021). The AutoDock suite at 30. Protein Sci..

[89] Brown T. (2014). ChemDraw. Sci. Teach..

[90] Ejaz S.A., Aziz M., Zafar Z., Akhtar N., Ogaly H.A. (2023). Revisiting the inhibitory potential of protein kinase inhibitors against NEK7 protein via comprehensive computational investigations. Sci. Rep..

[91] Ejaz S.A., Aziz M., Fayyaz A., Wani T.A., Zargar S. (2023). Computer-aided approach for the identification of lead molecules as the inhibitors of cholinesterase’s and monoamine oxidases: Novel target for the treatment of Alzheimer’s disease. J. Serb. Chem. Soc..

[92] Aziz M., Ejaz S.A., Tamam N., Siddique F., Riaz N., Qais F.A., Chtita S., Iqbal J. (2022). Identification of potent inhibitors of NEK7 protein using a comprehensive computational approach. Sci. Rep..

