# Supplementary material for: Pharmacognostic Evaluation, Chemical Characterization, and Antibacterial Activity of Bassia indica (Wight) A.J. Scott
Source: Plants (Basel). 2024 Jun 25;13(13):1753. doi: 10.3390/plants13131753 (PMC11244141; doi:10.3390/plants13131753)

My Qual X-Report

Sample Name:

Run Time(min): 24.51

GCMS analysis of ethyl acetate fraction of *B. indica*

Injection Volume(µl):1 1.00

Low Mass(m/z): 50

High Mass(m/z): 650

Instrument Name:Thermo Scientific (GC-MS) DSQ

Instrument Model:Thermo Scientific GC Focus Series DSQ

Instrument Software Version:2.0.7 1

RT: 0.00 - 27.51

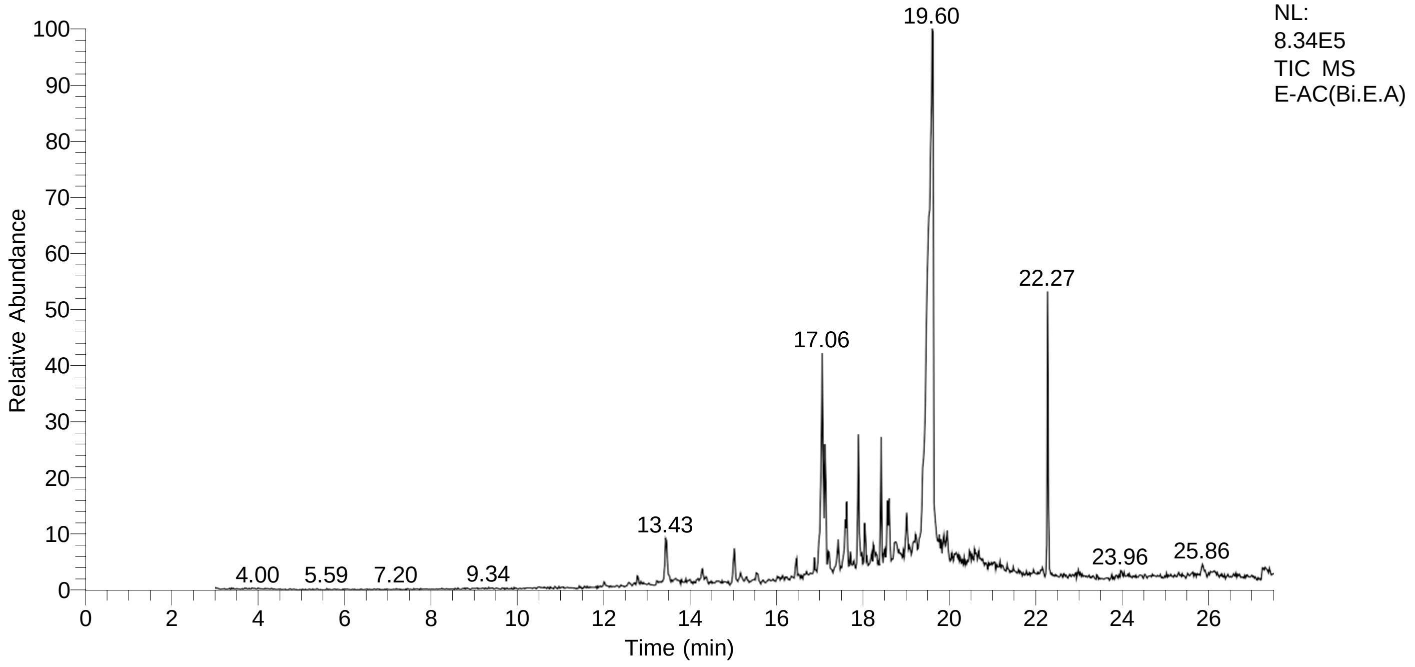

RT	Peak Area	Peak Height
12.00	20791.12	2372.70
12.80	82141.97	5167.19
13.45	437346.07	29701.20
14.29	131044.78	10226.13
15.02	218395.75	16418.37
15.53	55067.21	5601.27
16.10	16027.86	1475.14
16.44	63564.90	7595.22
17.06	1673594.84	142364.71
17.61	407589.89	34679.15
17.89	464672.99	43393.55
18.42	556934.30	28693.17
19.01	145017.37	16868.60
19.56	7714808.19	521876.73
20.17	15537.61	2407.85
20.62	227835.71	12376.04
21.01	100722.76	4206.08
22.27	771874.01	80659.23
22.98	66194.59	3843.92
23.98	93114.66	5220.05
24.43	23026.54	1647.10
24.72	18258.01	1463.97
25.86	341146.40	10303.28
26.63	86961.30	3547.15
27.30	93506.21	8566.91

My Qual X-Report

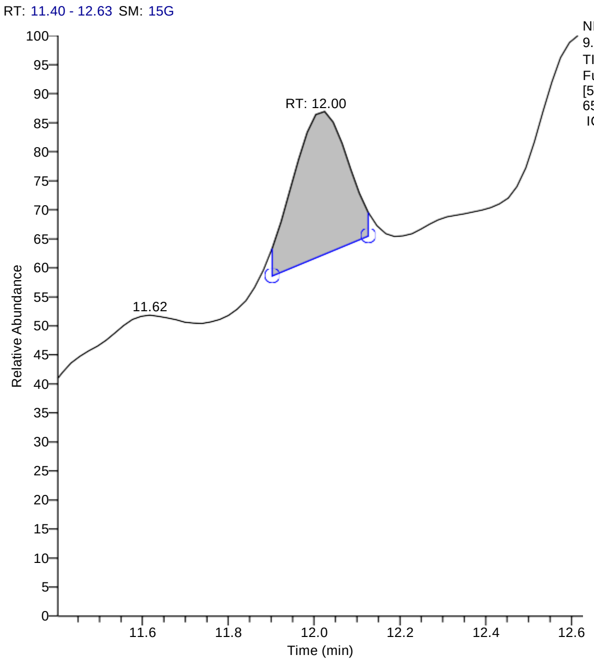

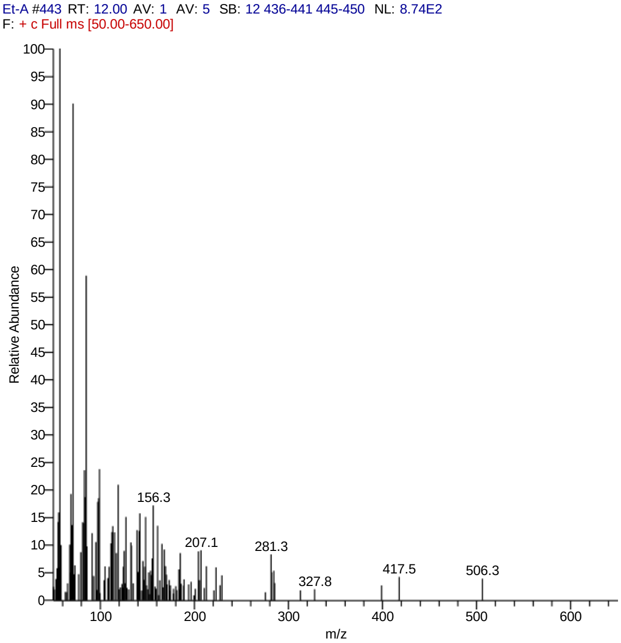

SI	RSI	Compound Name	Library	Probability	Area %	Area	RT
592	605	Octadecane, 3-ethyl-5-(2-ethylbutyl)-	replib	19.26	0.15	20791.12	12.00
589	608	Octadecane, 3-ethyl-5-(2-ethylbutyl)-	MAINLIB	19.26	0.15	20791.12	12.00
555	678	Tetradecane, 2,6,10-trimethyl-	MAINLIB	4.69	0.15	20791.12	12.00

Hit Spectrum | Delta | Compound Structure

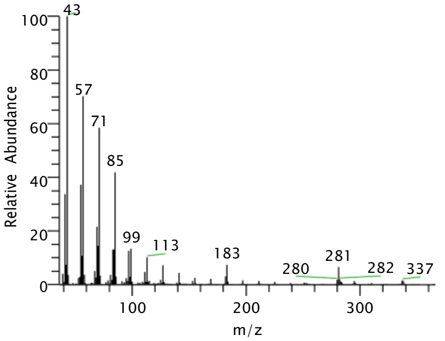

Raw data - Library entry

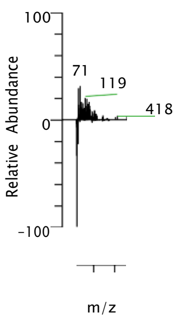

Octadecane, 3-ethyl-5-(2-ethylbutyl)-
Formula C26H54, MW 366, CAS# 55282-12-7, Entry# 2126
3-Ethyl-5-(2'-ethylbutyl)octadecane

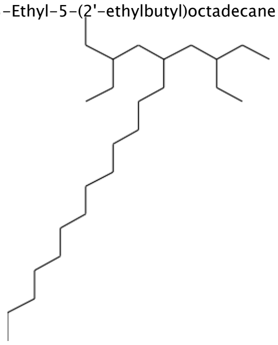

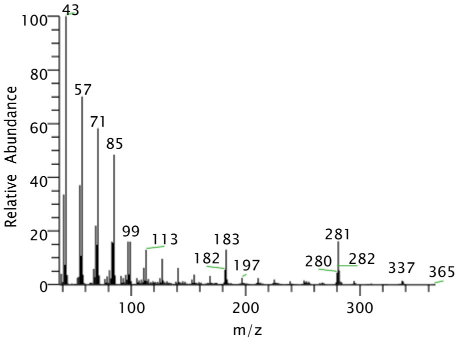

Raw data - Library entry

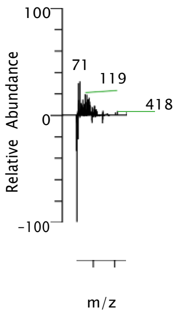

Octadecane, 3-ethyl-5-(2-ethylbutyl)-
Formula C26H54, MW 366, CAS# 55282-12-7, Entry# 7093
3-Ethyl-5-(2'-ethylbutyl)octadecane

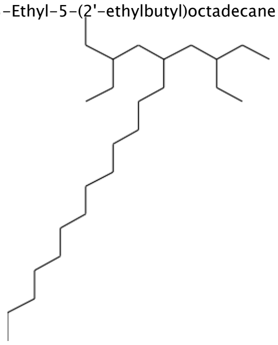

My Qual X-Report

Hit Spectrum | Delta | Compound Structure

Raw data - Library entry

Tetradecane, 2,6,10-trimethyl-
Formula C17H36, MW 240, CAS# 14905-56-7, Entry# 20933
2,6,10-Trimethyltetradecane

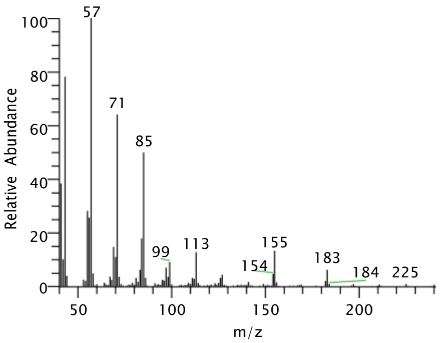

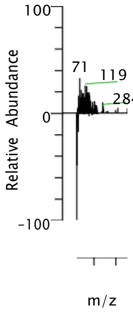

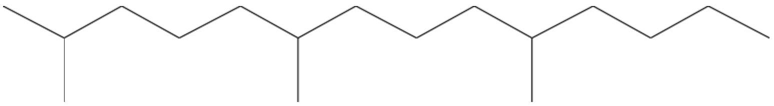

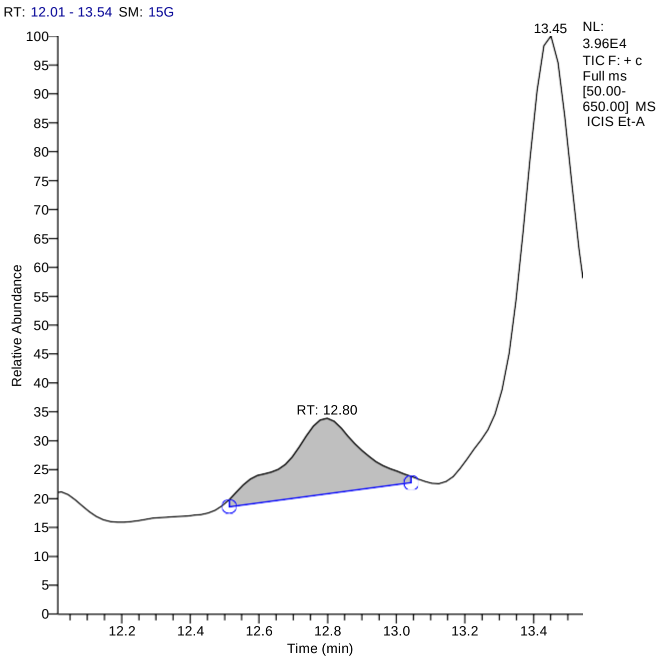

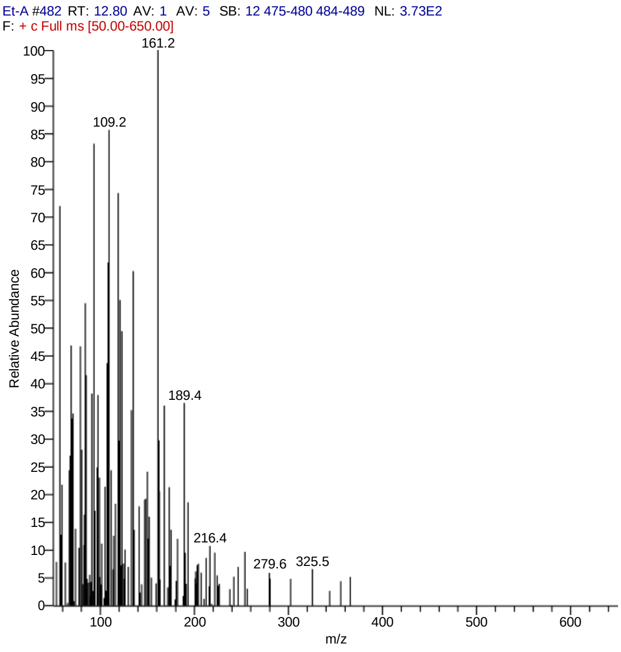

SI	RSI	Compound Name	Library	Probability	Area %	Area	RT
562	642	Illudol	MAINLIB	4.91	0.59	82141.97	12.80
559	685	Cyclohexanemethanol, 4-ethenyl-à,à,4-trimethyl-3-(1-methylethenyl)-, [1R-(1à,3à,4á)]-	MAINLIB	4.33	0.59	82141.97	12.80
559	669	3,7-Cyclodecadiene-1-methanol, à,à,4,8-tetramethyl-, [s-(Z,Z)]	MAINLIB	4.33	0.59	82141.97	12.80

Hit Spectrum | Delta | Compound Structure

SI 562, RSI 642, MAINLIB, Entry# 2582, CAS# 16981-75-2, Illudol

Raw data - Library entry

Illudol
Formula C15H26O, MW 222, CAS# 16981-75-2, Entry# 2582
3,6,6,7b-Tetramethyldecahydro-1H-cyclobuta[e]inden-3-ol #

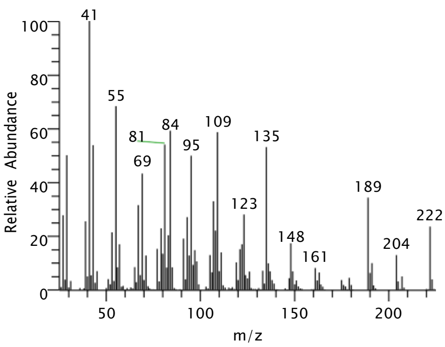

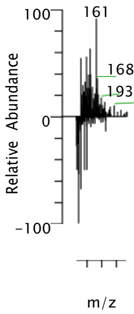

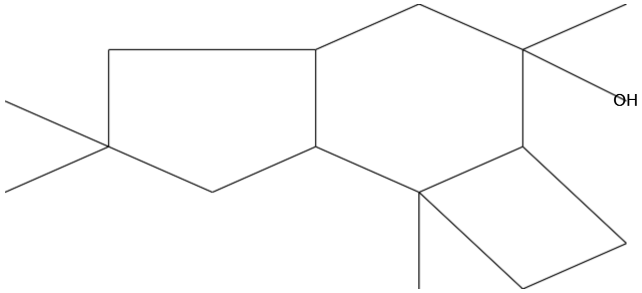

My Qual X-Report

Hit Spectrum	Delta	Compound Structure

Raw data - Library entry

Cyclohexanemethanol, 4-ethenyl-à,à,4-trimethyl-3-(1-methylethenyl)-, [1R-(1à,3à,4á)]-
Formula C15H26O, MW 222, CAS# 639-99-6, Entry# 25073
o-Menth-8-ene-4-methanol, à,à-dimethyl-1-vinyl-, (1S,2S,4R)-(-)-

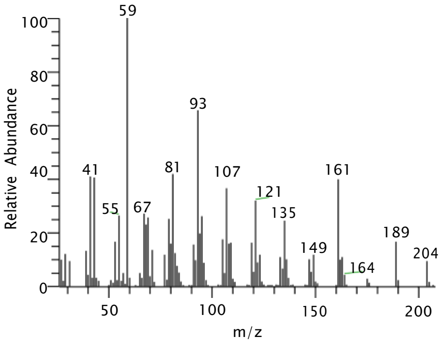

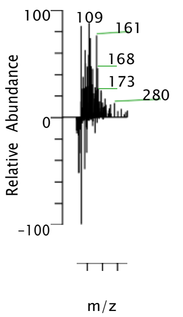

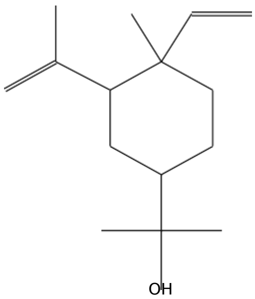

Raw data - Library entry

3,7-Cyclodecadiene-1-methanol, à,à,4,8-tetramethyl-, [s-(Z,Z)]
Formula C15H26O, MW 222, CAS# 21657-90-9, Entry# 25077
Hedycaryol

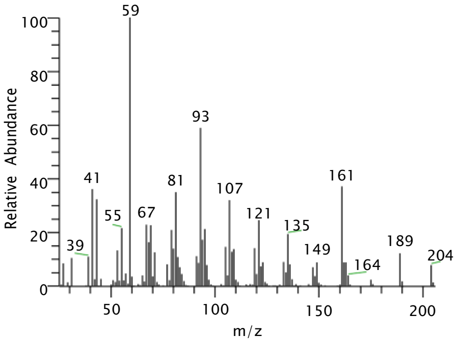

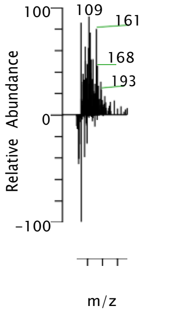

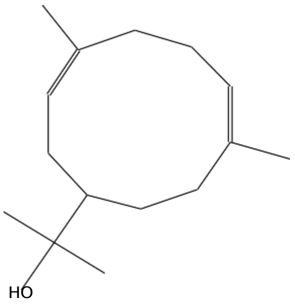

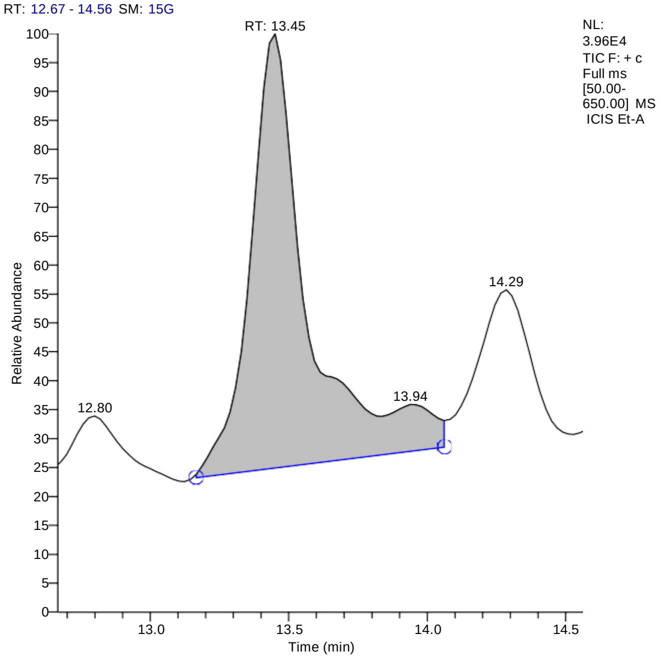

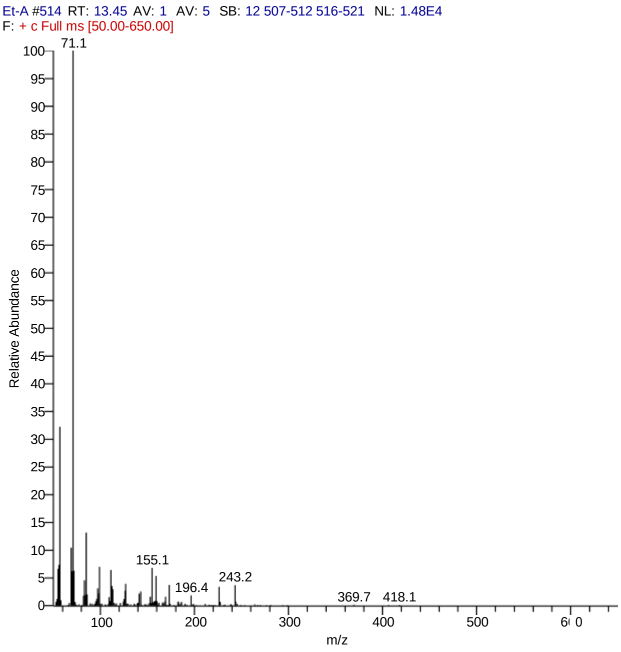

SI	RSI	Compound Name	Library	Probability	Area %	Area	RT
704	804	Hexadecane	replib	7.04	3.16	437346.07	13.45
692	796	Hexadecane	replib	7.04	3.16	437346.07	13.45
689	792	Heptacosane	replib	4.26	3.16	437346.07	13.45

My Qual X-Report

Hit Spectrum	Delta	Compound Structure

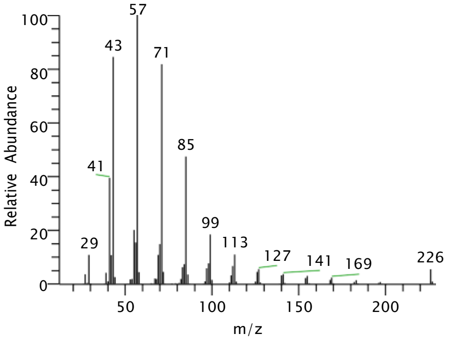

Raw data - Library entry

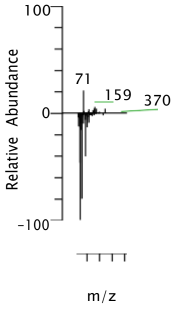

Hexadecane
Formula C16H34, MW 226, CAS# 544-76-3, Entry# 5517
n-Cetane

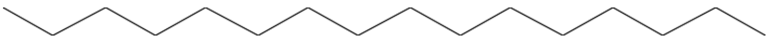

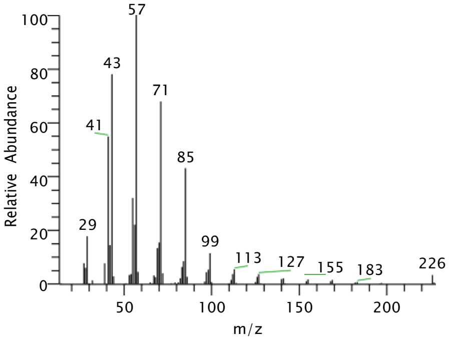

Raw data - Library entry

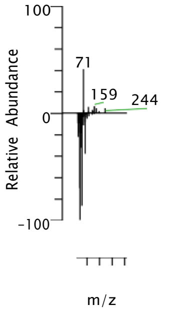

Hexadecane
Formula C16H34, MW 226, CAS# 544-76-3, Entry# 5518
n-Cetane

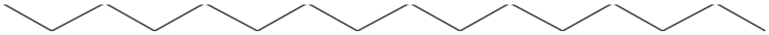

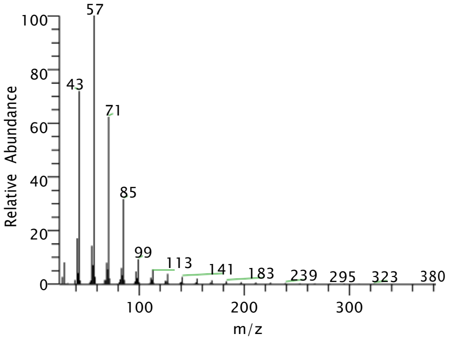

Raw data - Library entry

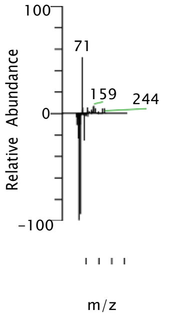

Heptacosane
Formula C27H56, MW 380, CAS# 593-49-7, Entry# 5462
n-Heptacosane

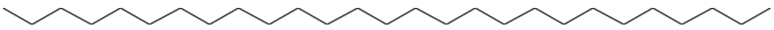

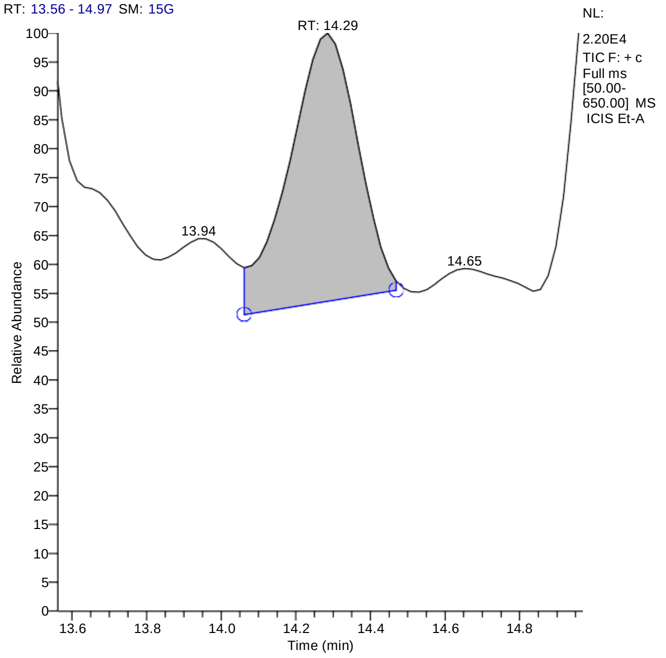

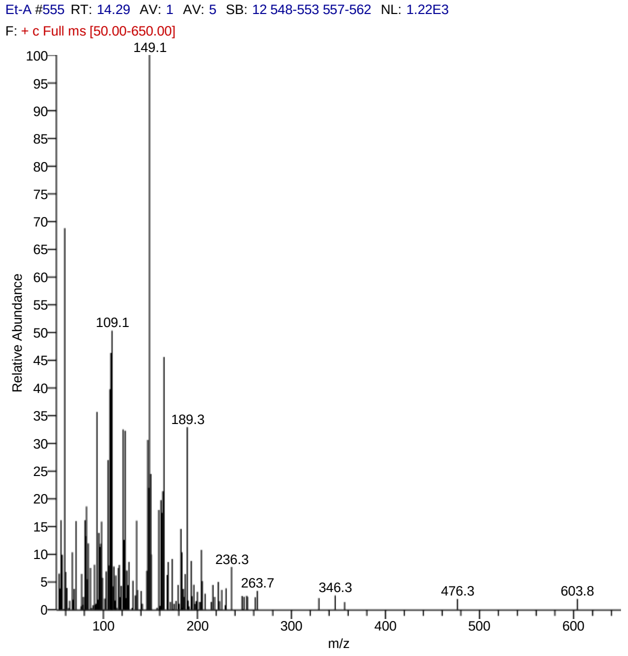

My Qual X-Report

SI	RSI	Compound Name	Library	Probability	Area %	Area	RT
611	737	2-Naphthalenemethanol, decahydro-à,à,4a-trimethyl-8-methylene-, [2R-(2à,4aà,8aá)]-	MAINLIB	20.23	0.95	131044.78	14.29
606	734	2-Naphthalenemethanol, decahydro-à,à,4a-trimethyl-8-methylene-, [2R-(2à,4aà,8aá)]-	replib	20.23	0.95	131044.78	14.29
595	677	2-Naphthalenemethanol, 1,2,3,4,4a,5,6,8a-octahydro-à,à,4a,8-tetramethyl-, (2à,4aà,8aà)-	MAINLIB	11.65	0.95	131044.78	14.29

Hit Spectrum | Delta | Compound Structure

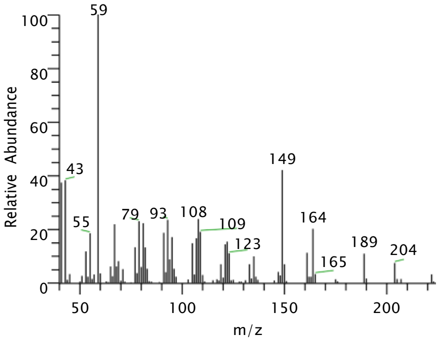

Raw data - Library entry

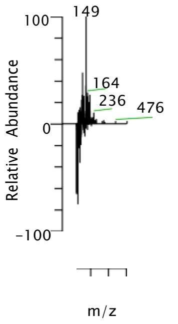

2-Naphthalenemethanol, decahydro-à,à,4a-trimethyl-8-methylene-, [2R-(2à,4aà,8aá)]-
Formula C15H26O, MW 222, CAS# 473-15-4, Entry# 25231
Eudesm-4(14)-en-11-ol

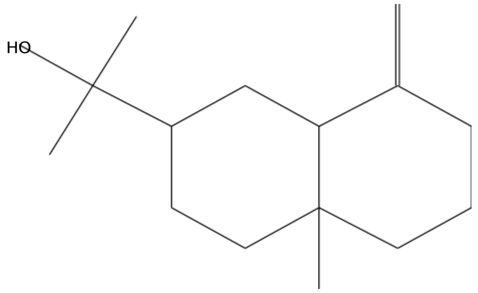

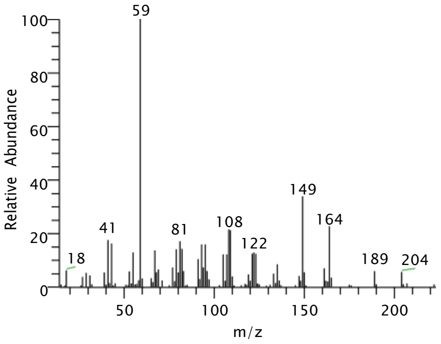

Raw data - Library entry

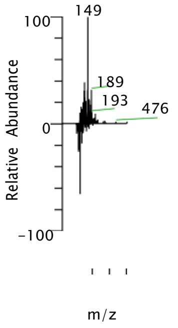

2-Naphthalenemethanol, decahydro-à,à,4a-trimethyl-8-methylene-, [2R-(2à,4aà,8aá)]-
Formula C15H26O, MW 222, CAS# 473-15-4, Entry# 6632
Eudesm-4(14)-en-11-ol

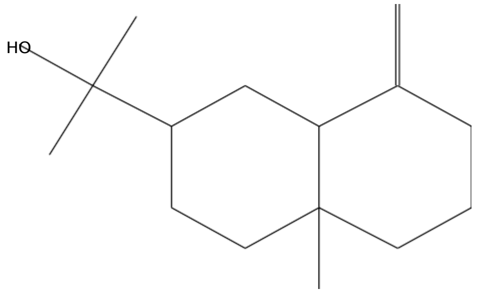

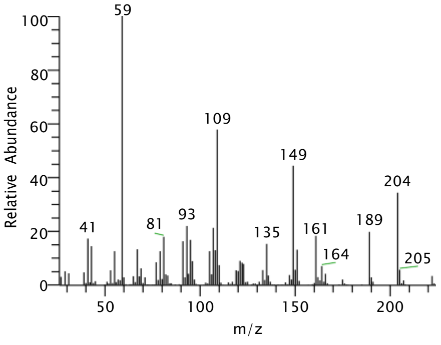

Raw data - Library entry

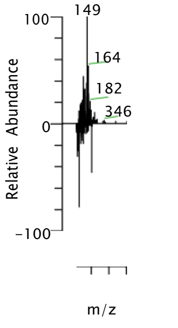

2-Naphthalenemethanol, 1,2,3,4,4a,5,6,8a-octahydro-à,à,4a,8-tetramethyl-, (2à,4aà,8aà)-
Formula C15H26O, MW 222, CAS# 79254-46-9, Entry# 25133
2-(4a,8-Dimethyl-1,2,3,4,4a,5,6,8a-octahydro-2-naphthalenyl)-2-propanol #

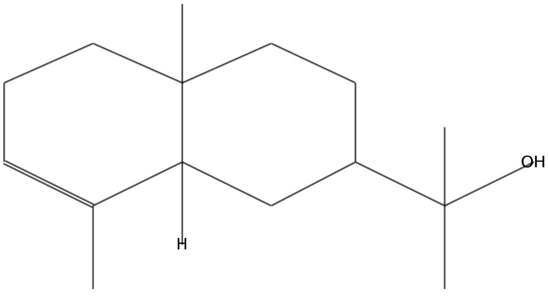

My Qual X-Report

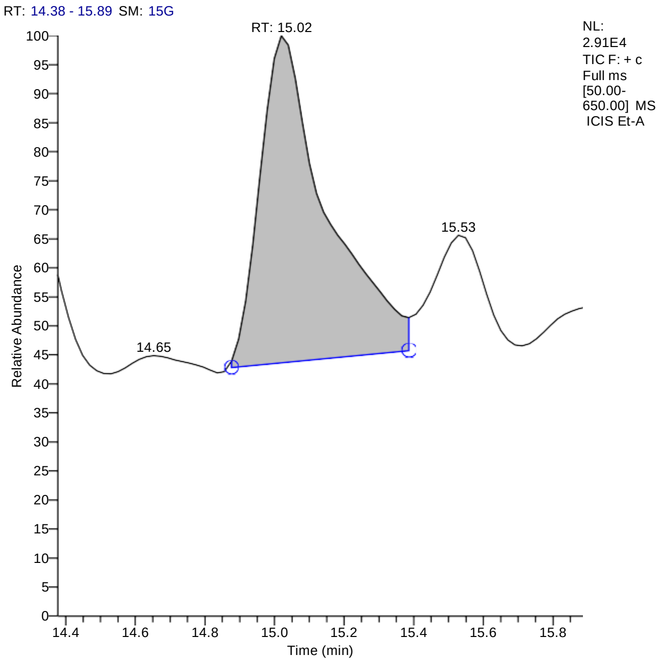

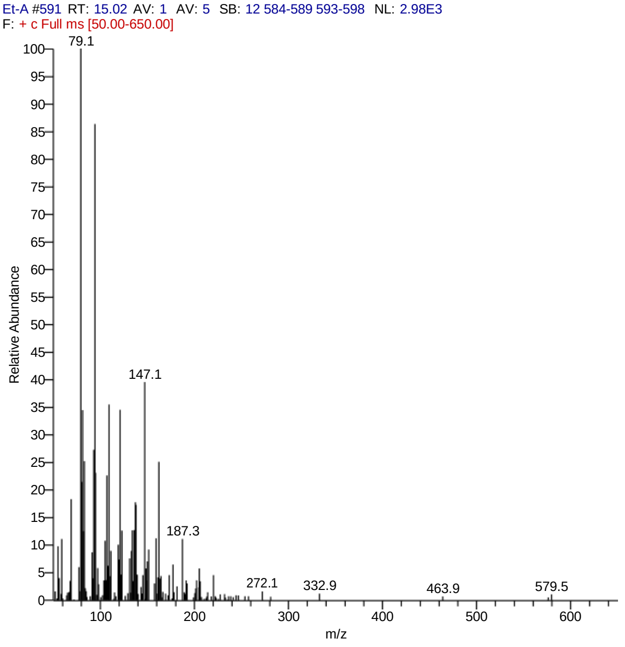

SI	RSI	Compound Name	Library	Probability	Area %	Area	RT
670	703	Isoaromadendrene epoxide	MAINLIB	18.50	1.58	218395.75	15.02
658	692	Diepicedrene-1-oxide	MAINLIB	12.32	1.58	218395.75	15.02
656	679	Longifolenaldehyde	MAINLIB	11.36	1.58	218395.75	15.02

Hit Spectrum | Delta | Compound Structure

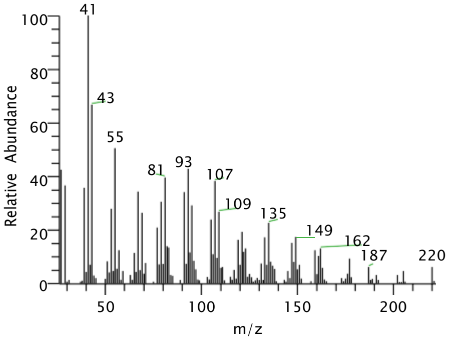

Raw data - Library entry

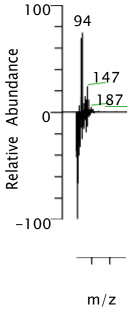

Isoaromadendrene epoxide
Formula C15H24O, MW 220, CAS# NA, Entry# 2095

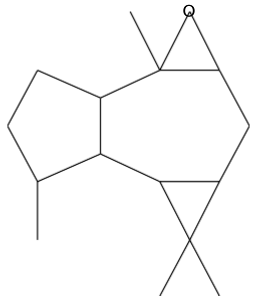

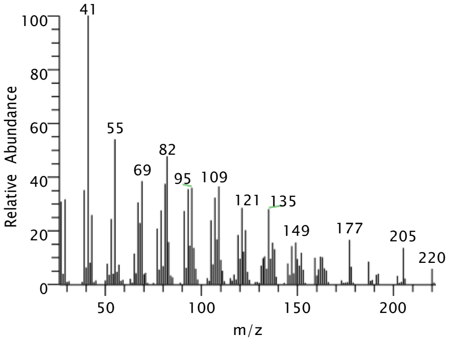

Raw data - Library entry

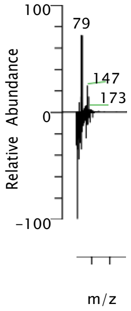

Diepicedrene-1-oxide
Formula C15H24O, MW 220, CAS# NA, Entry# 2571

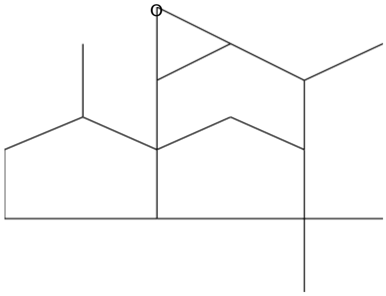

My Qual X-Report

Hit Spectrum | Delta | Compound Structure

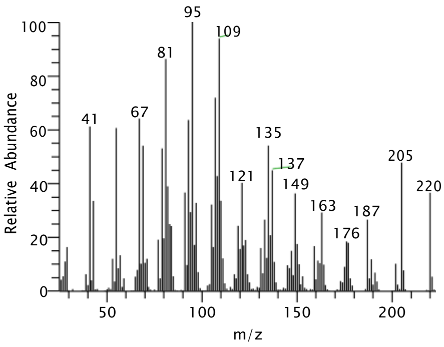

Raw data - Library entry

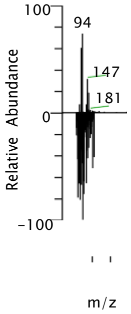

Longifolenaldehyde
Formula C15H24O, MW 220, CAS# 19890-84-7, Entry# 53336

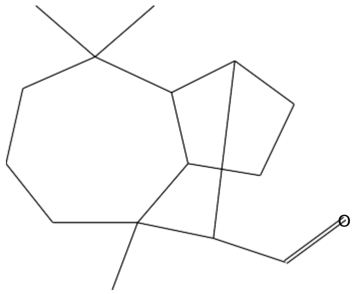

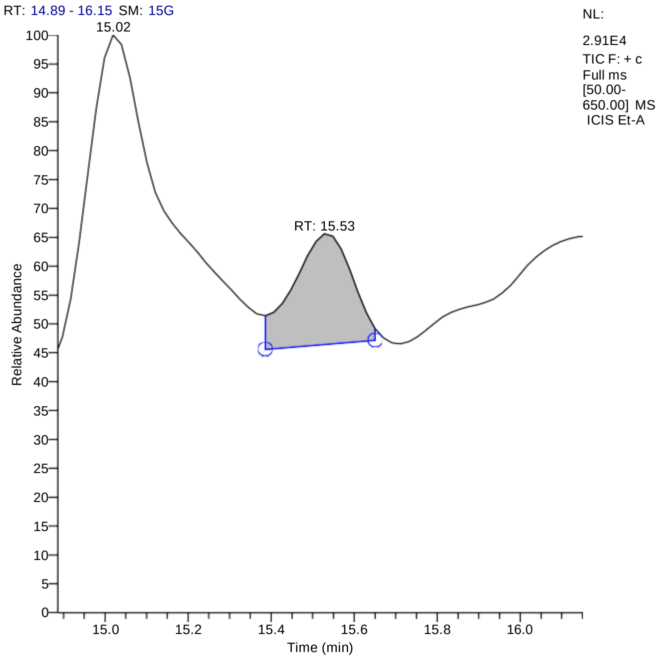

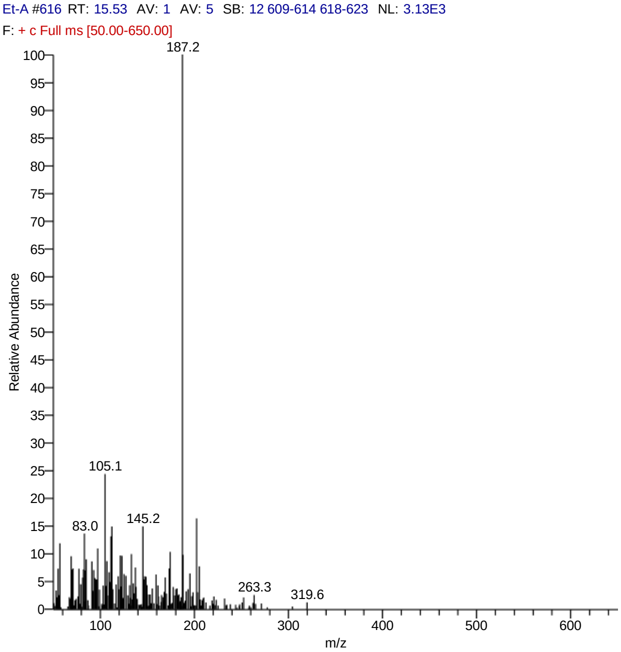

SI	RSI	Compound Name	Library	Probability	Area %	Area	RT
618	627	á-Santanol acetate	MAINLIB	19.89	0.40	55067.21	15.53
610	690	2-(4a,8-Dimethyl-1,2,3,4,4a,5,6,7-octahydro-naphthalen-2-yl)-prop-2-en-1-ol	MAINLIB	14.84	0.40	55067.21	15.53
599	645	9á-Acetoxy-3,5à,8-trimethyltricyclo[6.3.1.0(1,5)]dodec-3-ene	MAINLIB	10.18	0.40	55067.21	15.53

Hit Spectrum | Delta | Compound Structure

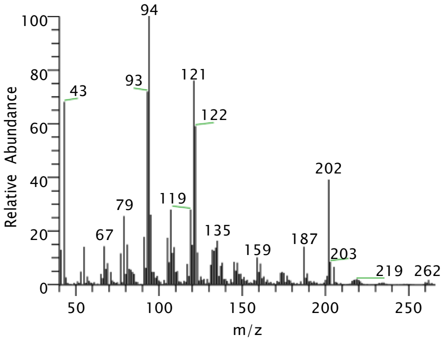

Raw data - Library entry

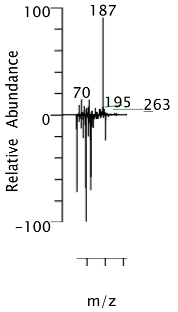

á-Santanol acetate
Formula C17H26O2, MW 262, CAS# NA, Entry# 52517

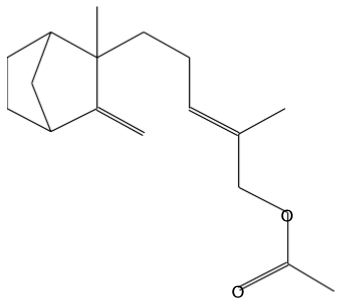

My Qual X-Report

Hit Spectrum | Delta | Compound Structure

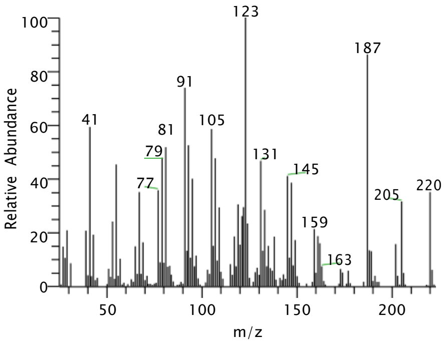

Raw data - Library entry

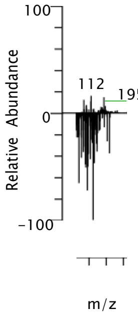

2-(4a,8-Dimethyl-1,2,3,4,4a,5,6,7-octahydro-naphthalen-2-yl)-prop-2-en-1-ol
Formula C15H24O, MW 220, CAS# NA, Entry# 76877

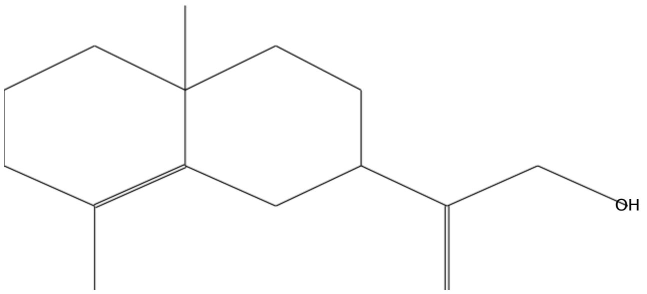

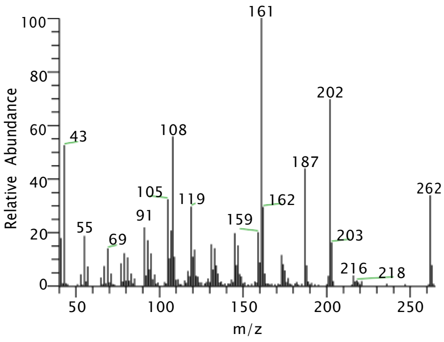

Raw data - Library entry

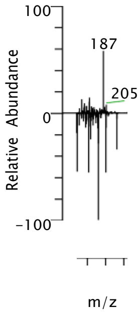

9á-Acetoxy-3,5à,8-trimethyltricyclo[6.3.1.0(1,5)]dodec-3-ene
Formula C17H26O2, MW 262, CAS# NA, Entry# 103662

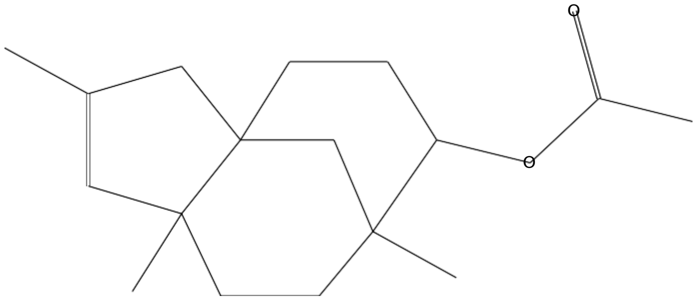

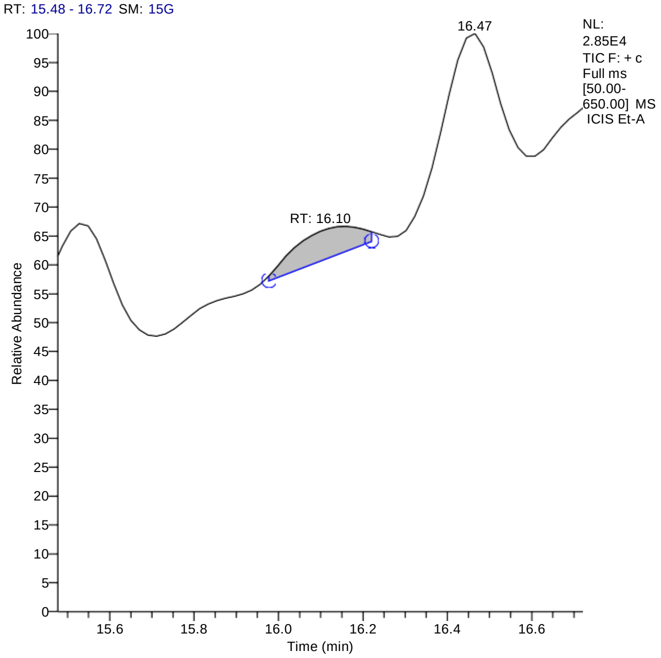

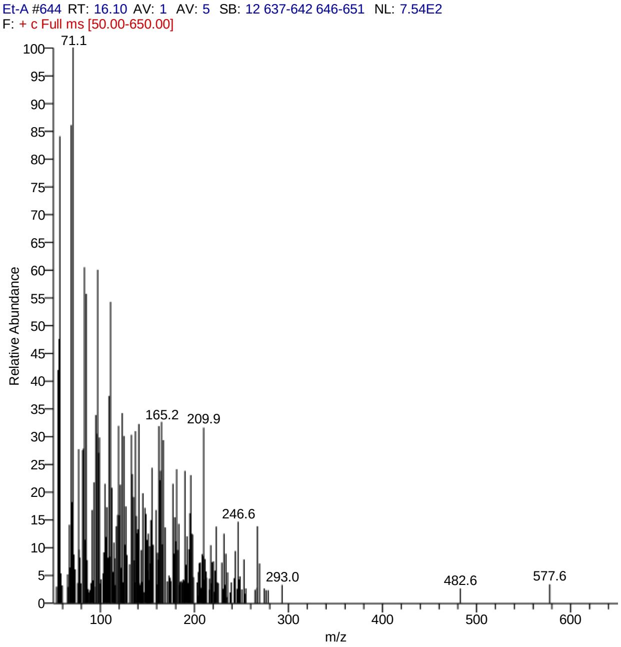

SI	RSI	Compound Name	Library	Probability	Area %	Area	RT
601	604	17-Pentatriacontene	MAINLIB	18.35	0.12	16027.86	16.10
584	589	2-Nonadecanone 2,4-dinitrophenylhydrazine	MAINLIB	10.01	0.12	16027.86	16.10
579	630	6,11-Bis(2-hydroxyfuran-4(5H)-on-3-yl) -7,10-diazahexadecan-6,10-diene	MAINLIB	8.07	0.12	16027.86	16.10

My Qual X-Report

Hit Spectrum | Delta | Compound Structure

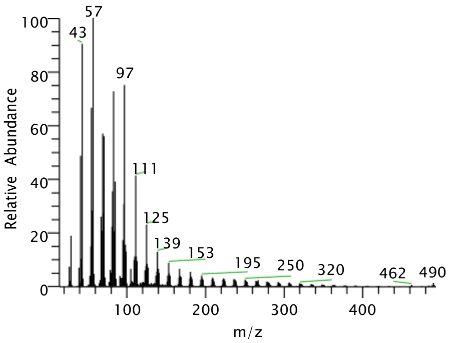

Raw data – Library entry

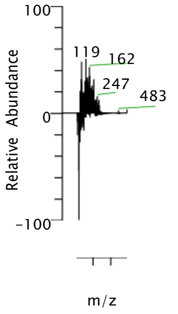

17-Pentatriacontene
Formula C35H70, MW 490, CAS# 6971-40-0, Entry# 21018
(17E)-17-Pentatriacontene #

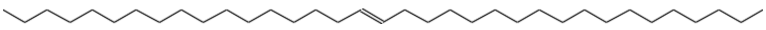

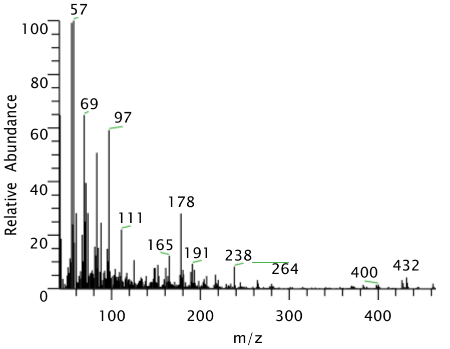

Raw data – Library entry

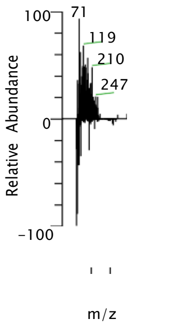

2-Nonadecanone 2,4-dinitrophenylhydrazine
Formula C25H42N4O4, MW 462, CAS# 28813-61-8, Entry# 21203
2-Nonadecanone, (2,4-dinitrophenyl)hydrazone

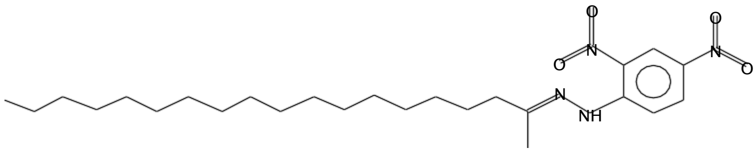

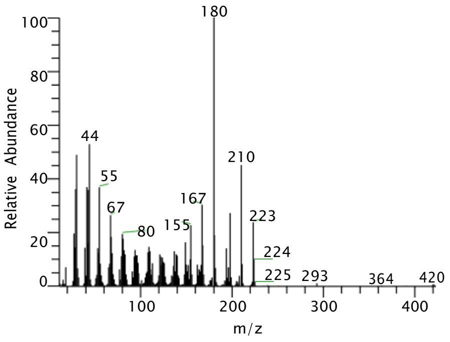

Raw data – Library entry

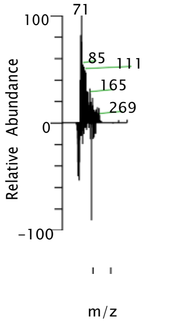

6,11-Bis(2-hydroxyfuran-4(5H)-on-3-yl)-7,10-diazahexadecan-6,10-diene
Formula C22H32N2O6, MW 420, CAS# NA, Entry# 114013

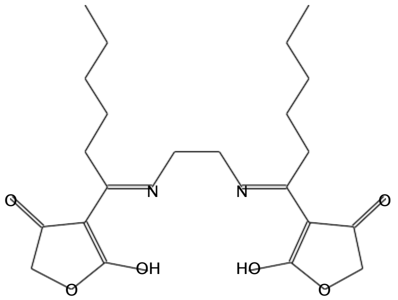

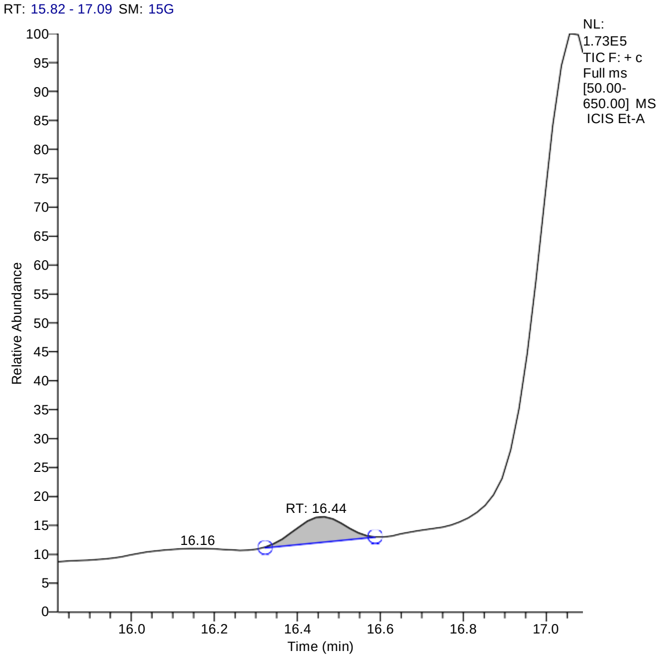

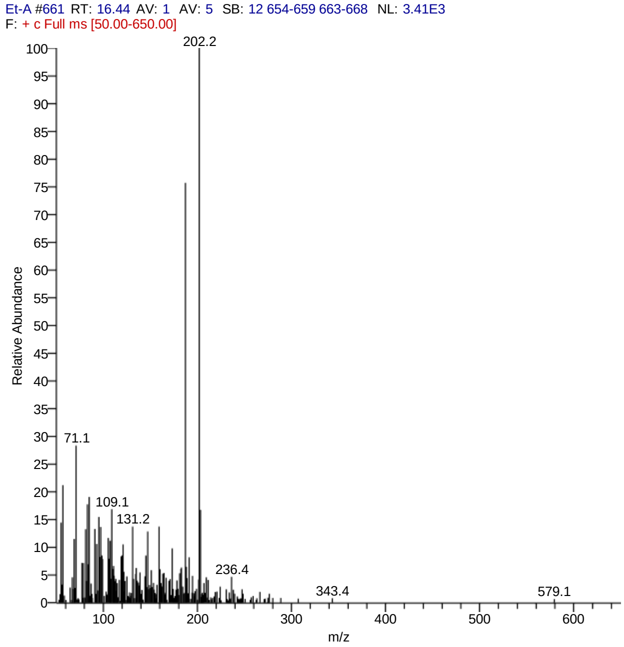

My Qual X-Report

SI	RSI	Compound Name	Library	Probability	Area %	Area	RT
651	671	Acetate, [6-(acetyloxy)-5,5,8a-trimethyl-2-methyleneperhydro-1-naphthalenyl]methyl ester	MAINLIB	33.43	0.46	63564.90	16.44
637	711	9á-Acetoxy-3á-hydroxy-3,5à,8-trimethyltricyclo[6.3.1.0(1,5)]dodecane	MAINLIB	20.93	0.46	63564.90	16.44
619	674	9á-Acetoxy-3,5à,8-trimethyltricyclo[6.3.1.0(1,5)]dodec-3-ene	MAINLIB	10.79	0.46	63564.90	16.44

Hit Spectrum	Delta	Compound Structure

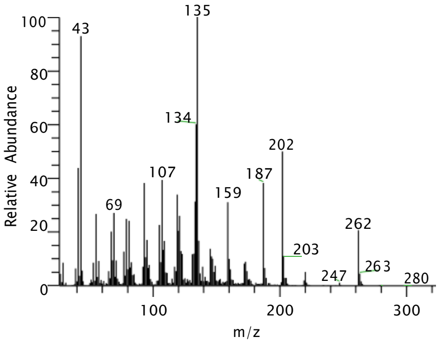

Raw data - Library entry

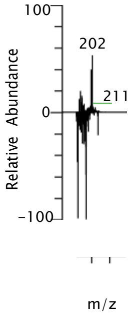

Acetate, [6-(acetyloxy)-5,5,8a-trimethyl-2-methyleneperhydro-1-naphthalenyl]methyl ester
Formula C19H30O4, MW 322, CAS# NA, Entry# 84620

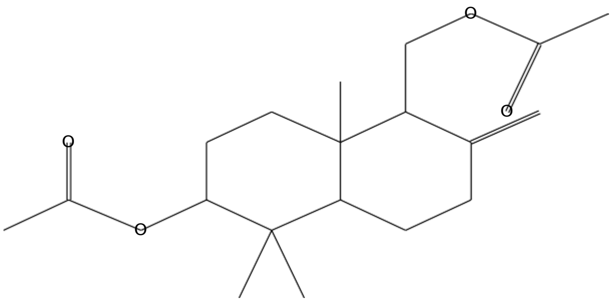

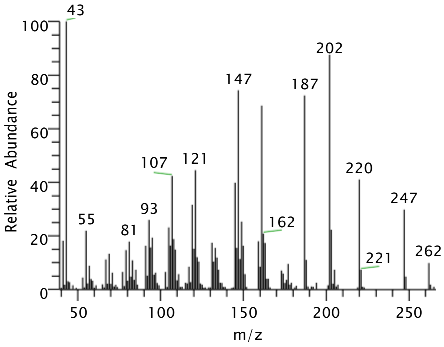

Raw data - Library entry

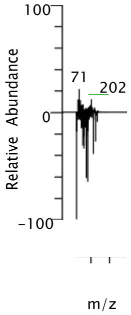

9á-Acetoxy-3á-hydroxy-3,5à,8-trimethyltricyclo[6.3.1.0(1,5)]dodecane
Formula C17H28O3, MW 280, CAS# NA, Entry# 11868

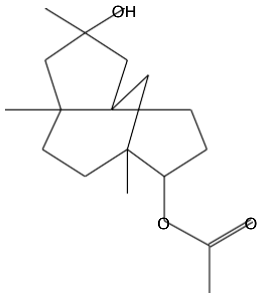

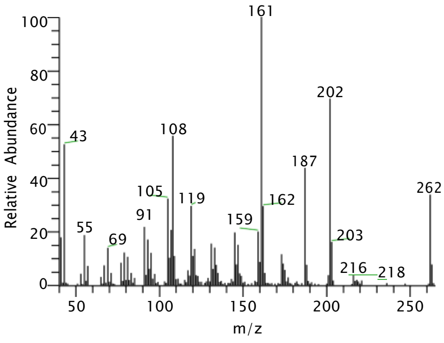

Raw data - Library entry

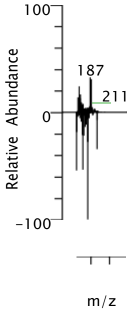

9á-Acetoxy-3,5à,8-trimethyltricyclo[6.3.1.0(1,5)]dodec-3-ene
Formula C17H26O2, MW 262, CAS# NA, Entry# 103662

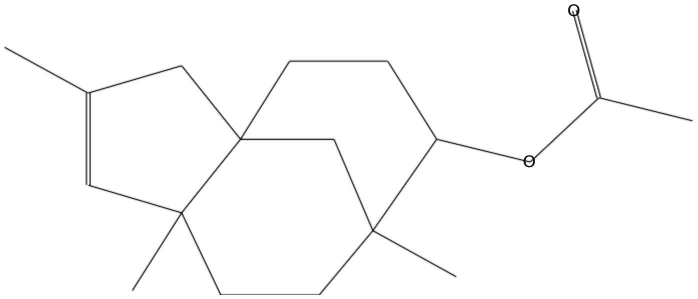

My Qual X-Report

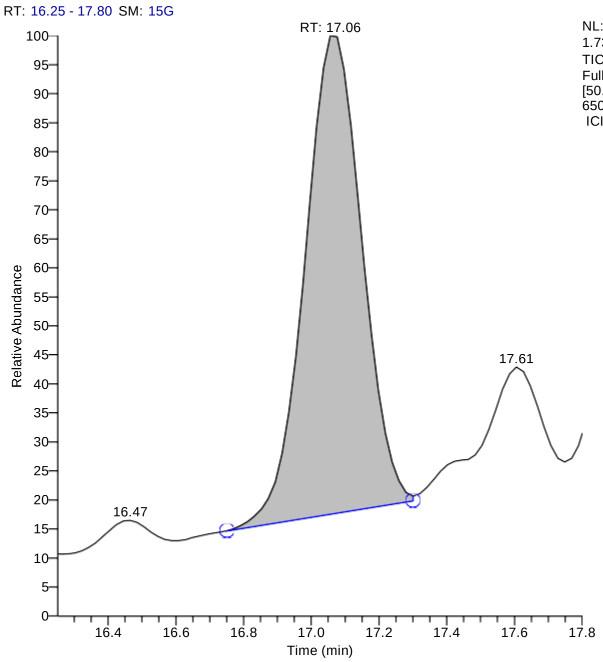

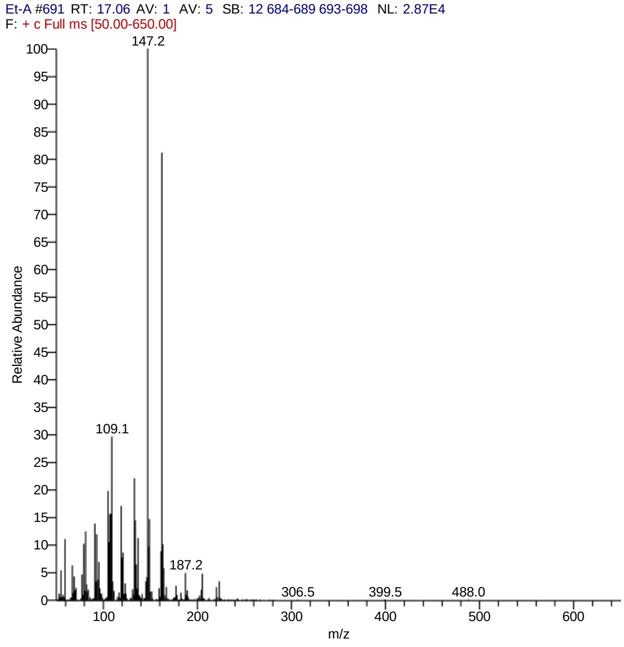

SI	RSI	Compound Name	Library	Probability	Area %	Area	RT
700	742	Bicyclo[2.2.2]octa-2,5-diene, 1,2,3,6-tetramethyl-	MAINLIB	17.70	12.11	1673594.84	17.06
683	719	4,4-Dimethyladamantan-2-ol	MAINLIB	9.66	12.11	1673594.84	17.06
672	759	Naphthalene, 2,3,4,4a,5,6,7,8-octahydro-2-hydroxy-[à/á]-2,4a-dimethyl-	MAINLIB	6.62	12.11	1673594.84	17.06

Hit Spectrum | Delta | Compound Structure

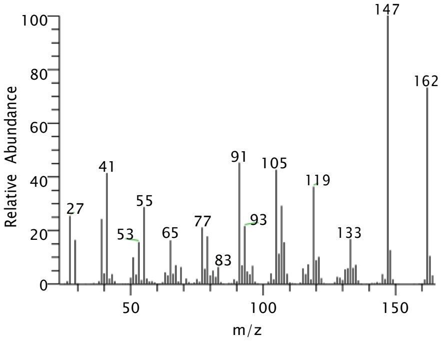

Raw data - Library entry

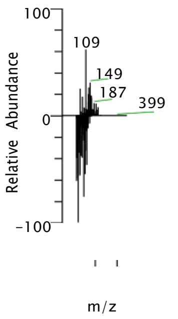

Bicyclo[2.2.2]octa-2,5-diene, 1,2,3,6-tetramethyl-
Formula C12H18, MW 162, CAS# 62338-43-6, Entry# 94366
1,2,3,6-Tetramethylbicyclo[2.2.2]octa-2,5-diene #

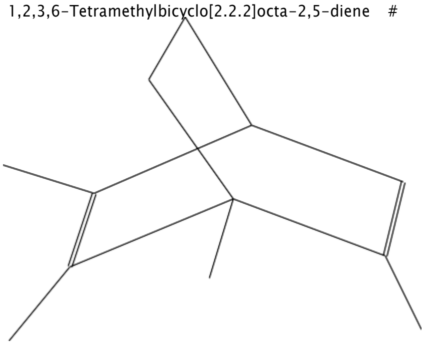

Raw data - Library entry

4,4-Dimethyladamantan-2-ol
Formula C12H20O, MW 180, CAS# NA, Entry# 94362

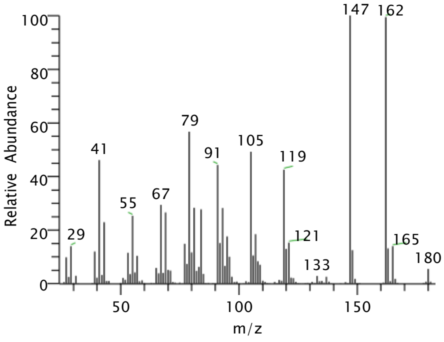

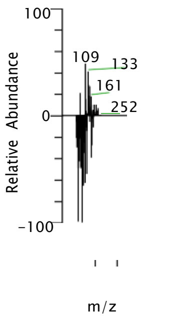

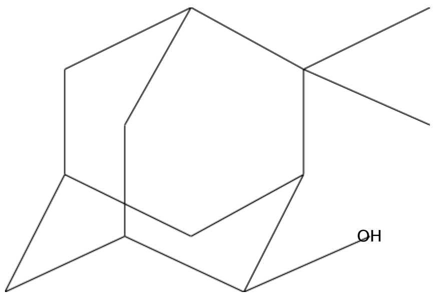

My Qual X-Report

Hit Spectrum | Delta | Compound Structure

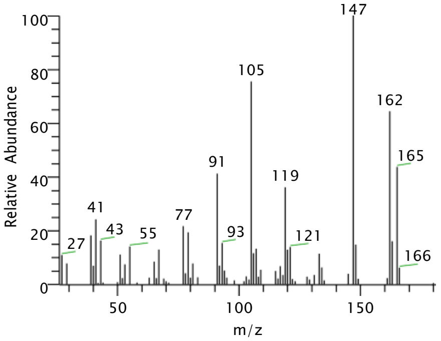

Raw data - Library entry

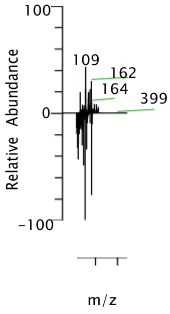

Naphthalene, 2,3,4,4a,5,6,7,8-octahydro-2-hydroxy-[à/á]-2,4a-dimethyl-
Formula C12H20O, MW 180, CAS# NA, Entry# 94105

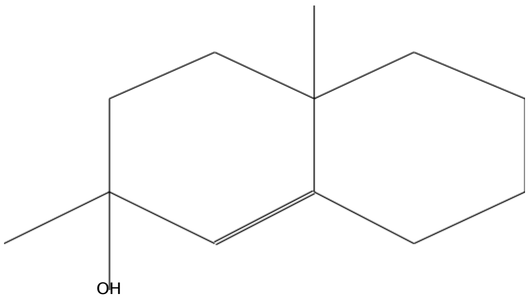

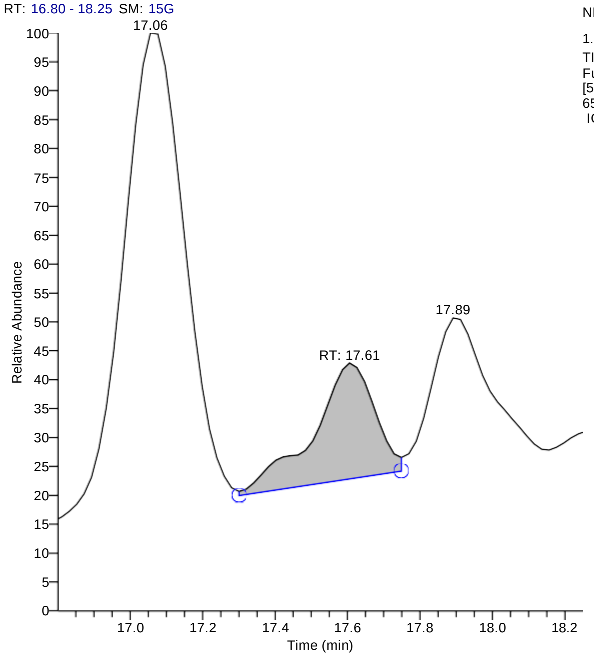

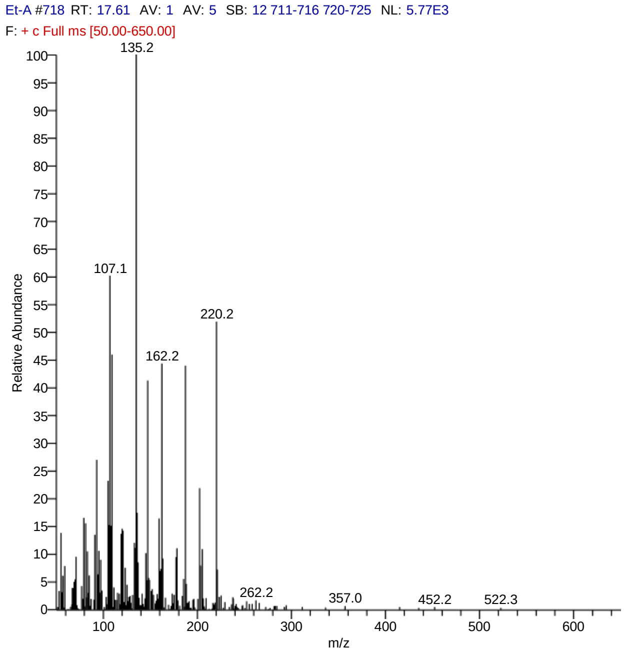

SI	RSI	Compound Name	Library	Probability	Area %	Area	RT
673	695	5-Hydroxymethyl-1,1,4a-trimethyl-6-methylenedecahydronaphthalen-2-ol	MAINLIB	9.54	2.95	407589.89	17.61
666	705	1H-Cycloprop[e]azulen-7-ol, decahydro-1,1,7-trimethyl-4-methylene-, [1ar-(1aà,4aà,7á,7aá,7bà)]-	MAINLIB	7.31	2.95	407589.89	17.61
665	682	Acetate, [6-(acetyloxy)-5,5,8a-trimethyl-2-methyleneperhydro-1-naphthalenyl]methyl ester	MAINLIB	7.03	2.95	407589.89	17.61

Hit Spectrum | Delta | Compound Structure

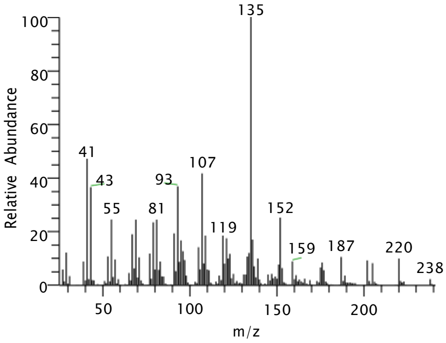

Raw data - Library entry

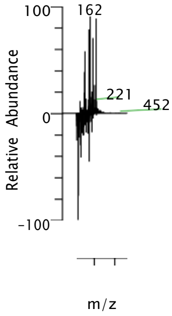

5-Hydroxymethyl-1,1,4a-trimethyl-6-methylenedecahydronaphthalen-2-ol
Formula C15H26O2, MW 238, CAS# NA, Entry# 84576

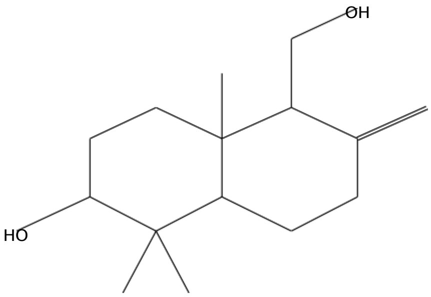

My Qual X-Report

Hit Spectrum | Delta | Compound Structure

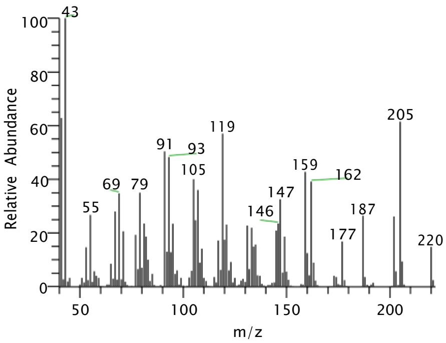

Raw data - Library entry

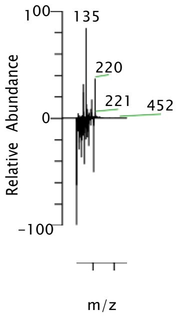

Formula C15H24O, MW 220, CAS# 6750-60-3, Entry# 5810

Spathulenol

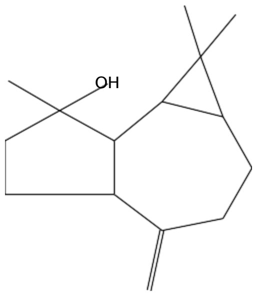

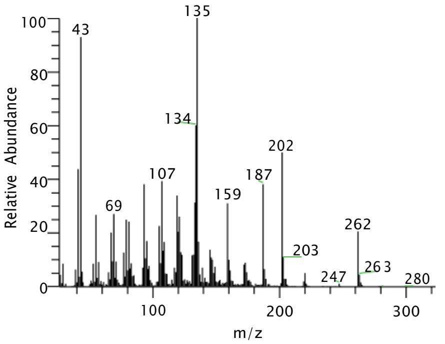

Raw data - Library entry

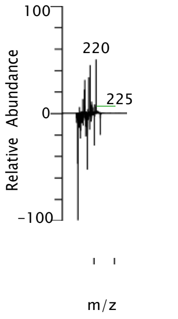

Acetate, [6-(acetyloxy)-5,5,8a-trimethyl-2-methyleneperhydro-1-naphthalenyl]methyl ester

Formula C19H30O4, MW 322, CAS# NA, Entry# 84620

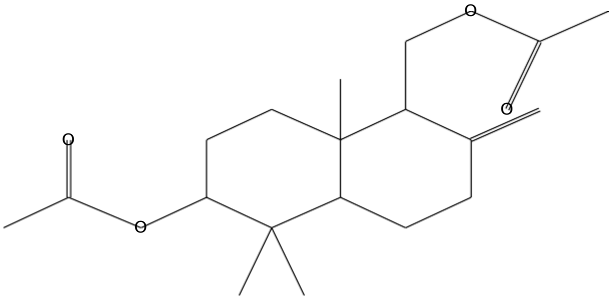

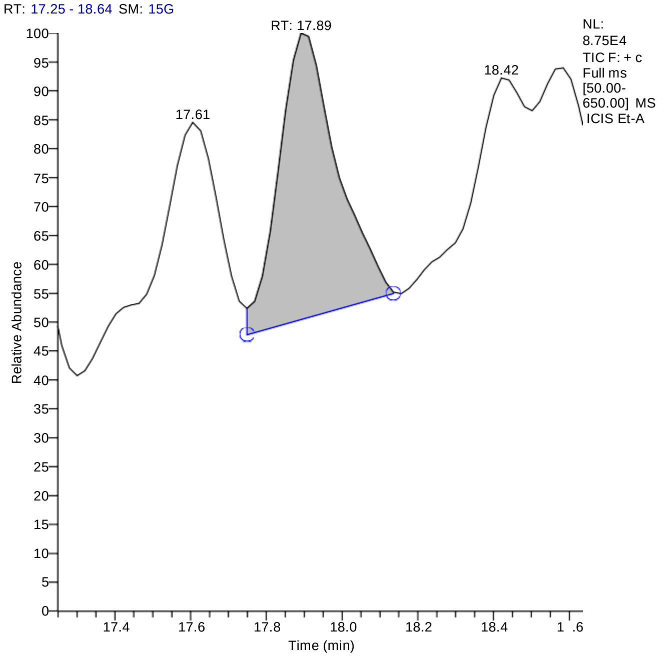

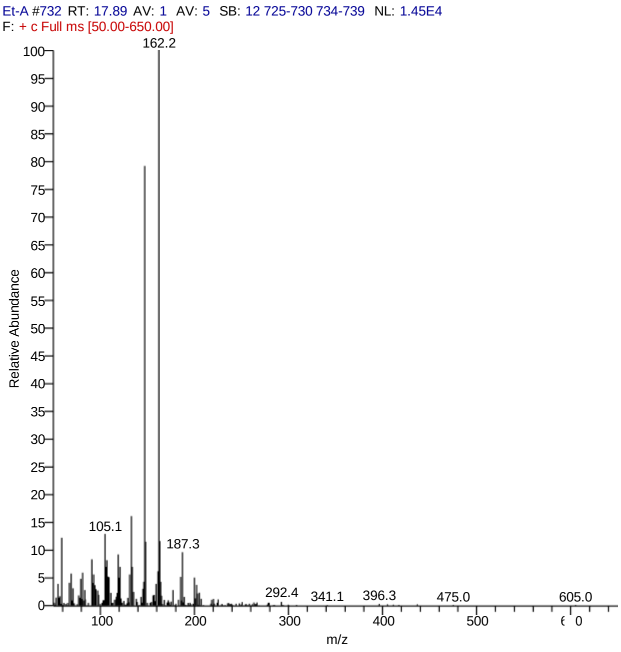

SI	RSI	Compound Name	Library	Probability	Area %	Area	RT
667	766	Benzene, hexamethyl-	replib	11.44	3.36	464672.99	17.89
666	786	Naphthalene, 2,3,4,4a,5,6,7,8-octahydro-2-hydroxy-[à/á]-2,4a-dimethyl-	MAINLIB	10.99	3.36	464672.99	17.89
661	751	Bicyclo[2.2.2]octa-2,5-diene, 1,2,3,6-tetramethyl-	MAINLIB	8.86	3.36	464672.99	17.89

My Qual X-Report

Hit Spectrum | Delta | Compound Structure

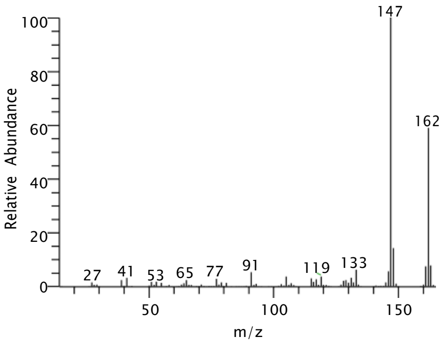

Raw data - Library entry

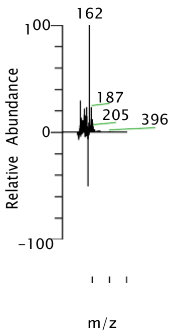

Benzene, hexamethyl-
Formula C12H18, MW 162, CAS# 87-85-4, Entry# 19595
Hexamethylbenzene

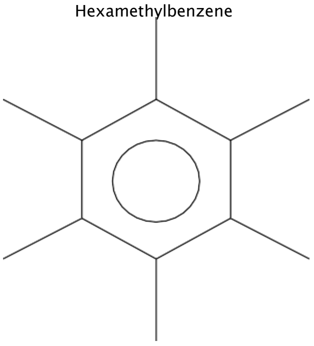

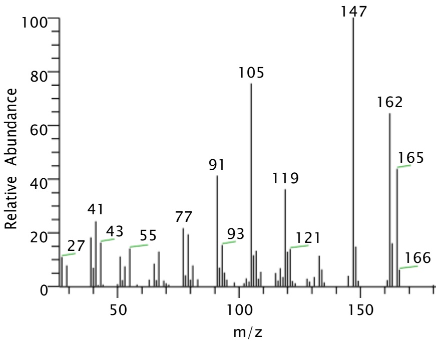

Raw data - Library entry

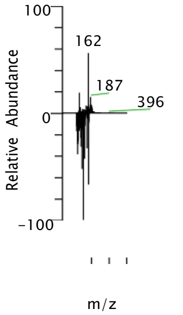

Naphthalene, 2,3,4,4a,5,6,7,8-octahydro-2-hydroxy-[à/á]-2,4a-dimethyl-
Formula C12H20O, MW 180, CAS# NA, Entry# 94105

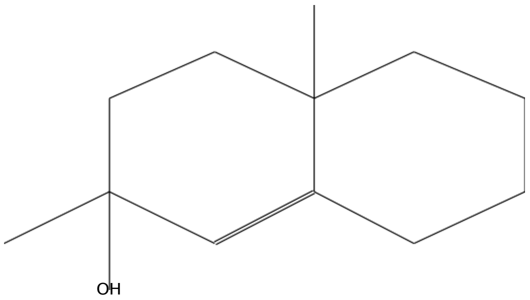

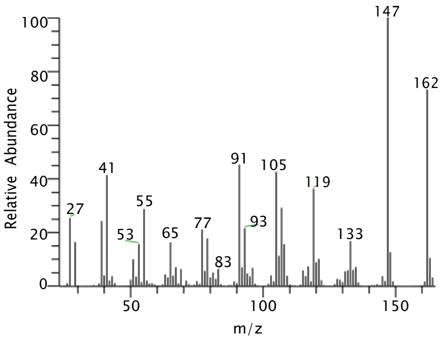

Raw data - Library entry

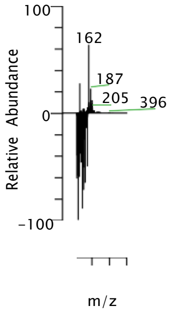

Bicyclo[2.2.2]octa-2,5-diene, 1,2,3,6-tetramethyl-
Formula C12H18, MW 162, CAS# 62338-43-6, Entry# 94366
1,2,3,6-Tetramethylbicyclo[2.2.2]octa-2,5-diene #

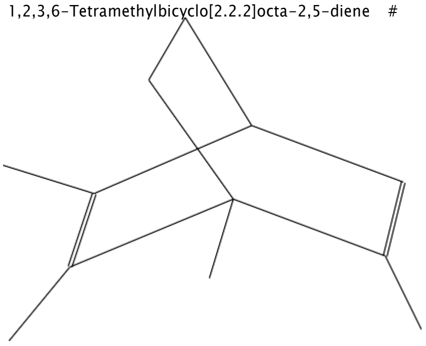

RT: 17.68 - 19.35 SM: 15G

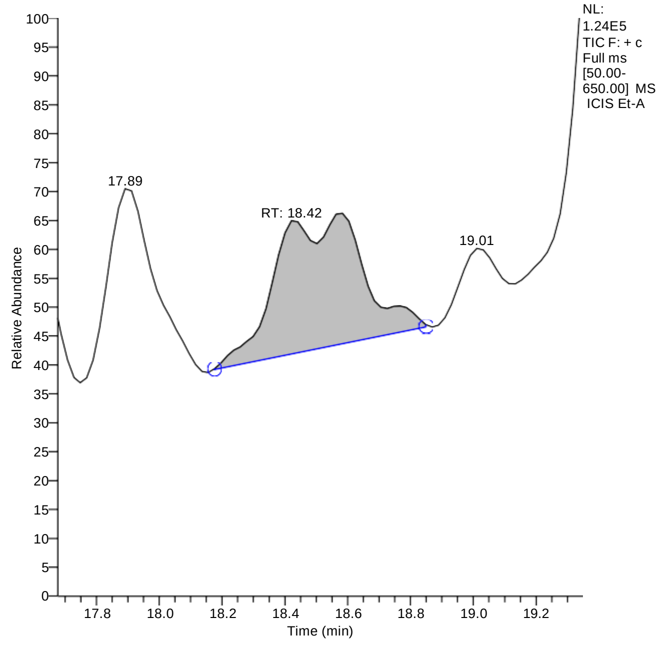

Et-A #758 RT: 18.42 AV: 1 AV: 5 SB: 12 751-756 760-765 NL: 4.90E3
F: + c Full ms [50.00-650.00]

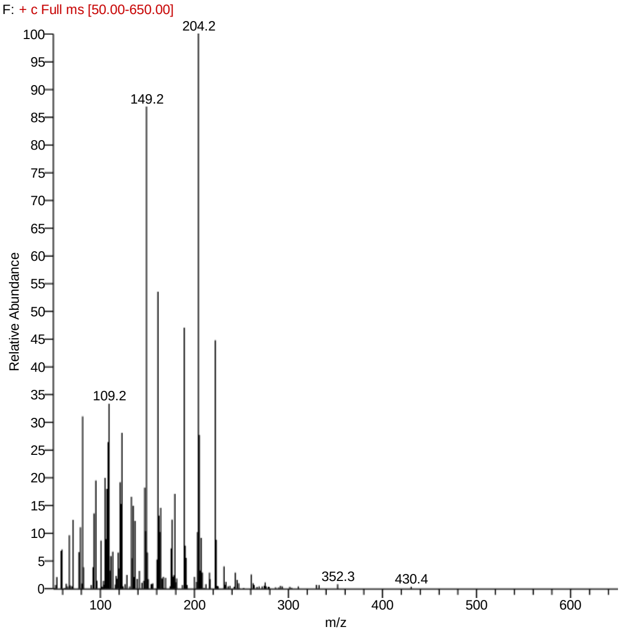

My Qual X-Report

SI	RSI	Compound Name	Library	Probability	Area %	Area	RT
684	719	1-Naphthalenol, decahydro-1,4a-dimethyl-7-(1-methylet hylidene)-, [1R-(1à,4aá,8aà)]-	replib	15.40	4.03	556934.30	18.42
683	738	Selina-6-en-4-ol	MAINLIB	14.80	4.03	556934.30	18.42
676	739	2-Naphthalenemethanol, 1,2,3,4,4a,5,6,8a-octahydro-à,à,4a,8-tet ramethyl-, [2R-(2à,4aà,8aá)]-	replib	11.34	4.03	556934.30	18.42

Hit Spectrum — Delta — Compound Structure

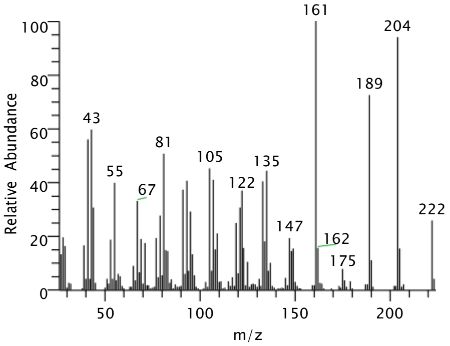

Raw data - Library entry

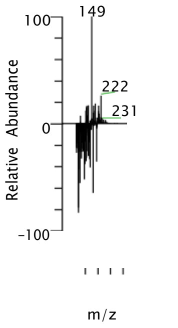

1-Naphthalenol, decahydro-1,4a-dimethyl-7-(1-methylethylidene)-, [1R-(1à,4aá,8aà)]-
Formula C15H26O, MW 222, CAS# 473-04-1, Entry# 20992
Eudesm-7(11)-en-4-ol

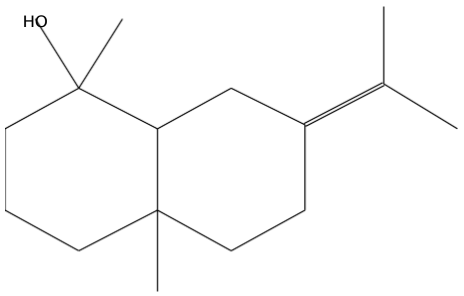

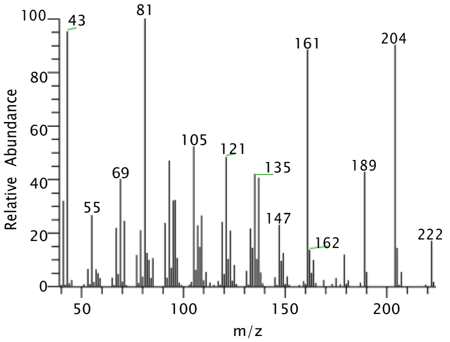

Raw data - Library entry

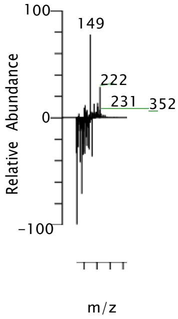

Selina-6-en-4-ol
Formula C15H26O, MW 222, CAS# NA, Entry# 39468

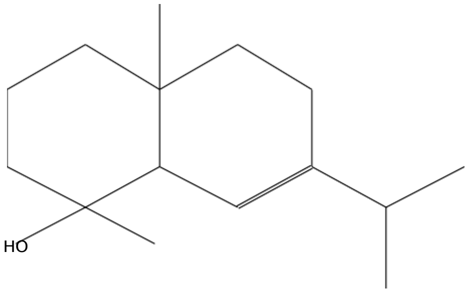

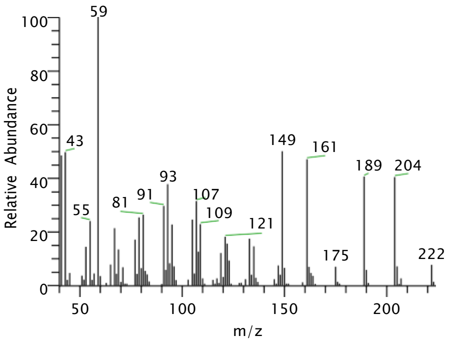

Raw data - Library entry

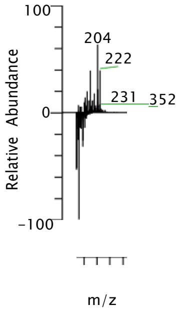

Formula C15H26O, MW 222, CAS# 473-16-5, Entry# 6631
à-Eudesmol

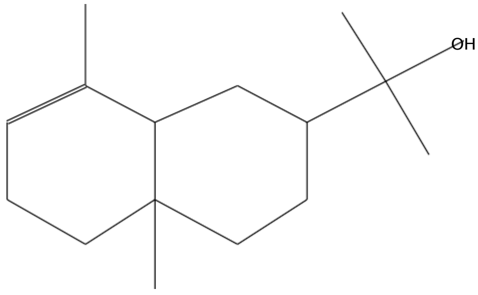

My Qual X-Report

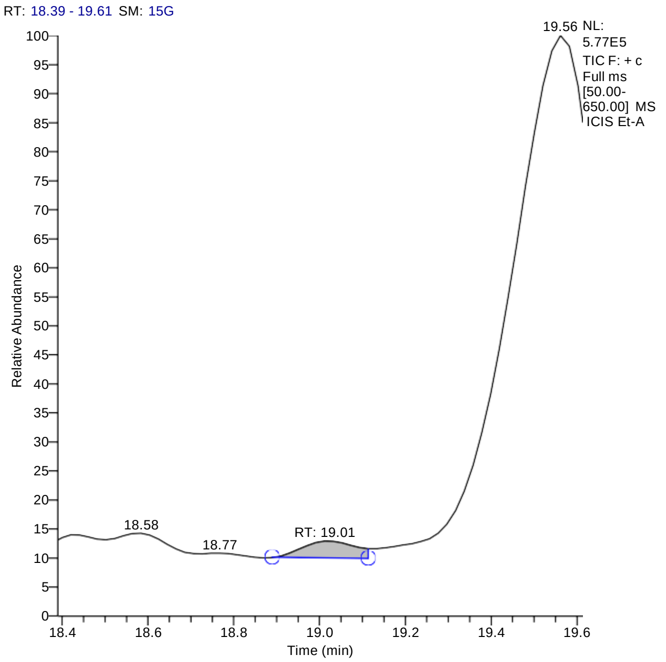

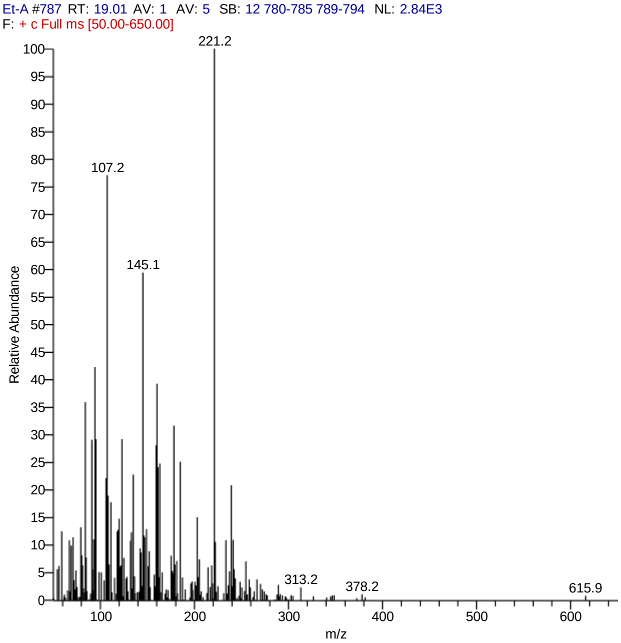

SI	RSI	Compound Name	Library	Probability	Area %	Area	RT
499	514	Gitoxigenin	replib	6.00	1.05	145017.37	19.01
496	558	Bicyclo[4.4.0]dec-2-ene-4-ol, 2-methyl-9-(prop-1-en-3-ol-2-yl)-	MAINLIB	5.30	1.05	145017.37	19.01
495	505	Retinoyl-á-glucuronide 6',3'-lactone	MAINLIB	5.10	1.05	145017.37	19.01

Hit Spectrum	Delta	Compound Structure

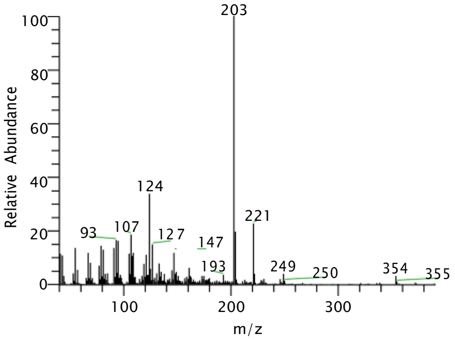

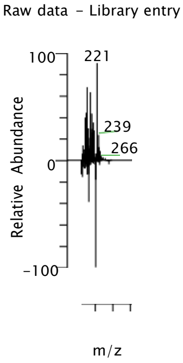

Gitoxigenin
Formula C23H34O5, MW 390, CAS# 545-26-6, Entry# 24021
Bigitaligenin

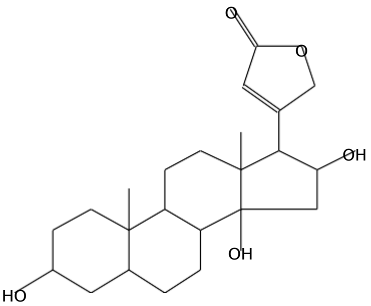

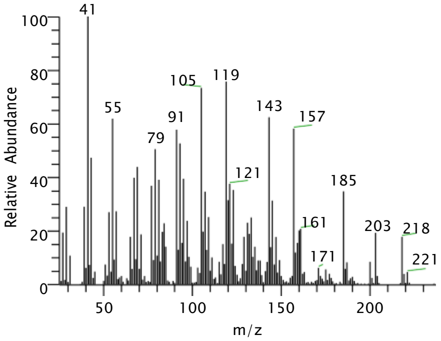

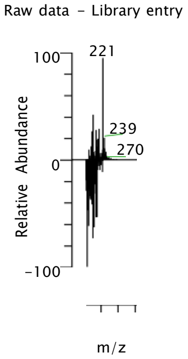

Bicyclo[4.4.0]dec-2-ene-4-ol, 2-methyl-9-(prop-1-en-3-ol-2-yl)-
Formula C15H24O2, MW 236, CAS# NA, Entry# 3585

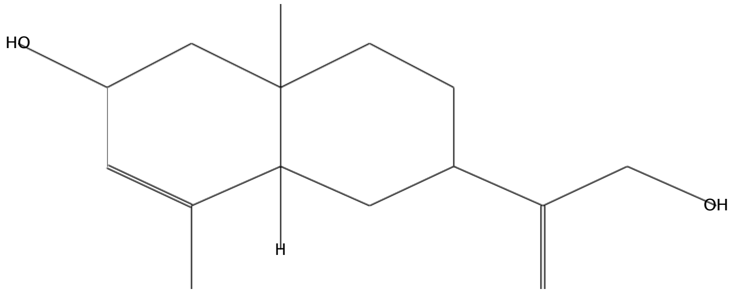

My Qual X-Report

Hit Spectrum | Delta | Compound Structure

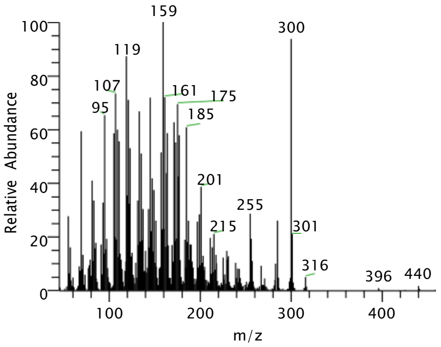

Raw data - Library entry

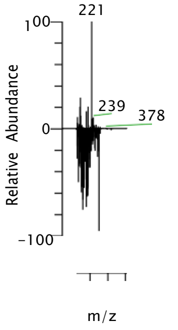

Retinoyl-á-glucuronide 6',3'-lactone
Formula C26H34O7, MW 458, CAS# 101470-87-5, Entry# 102434

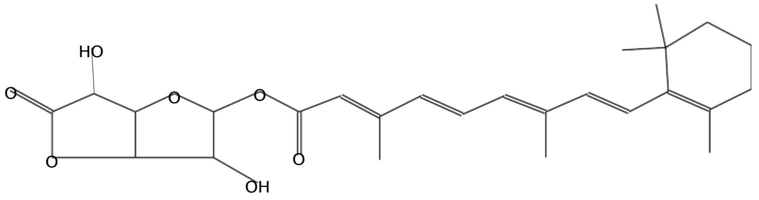

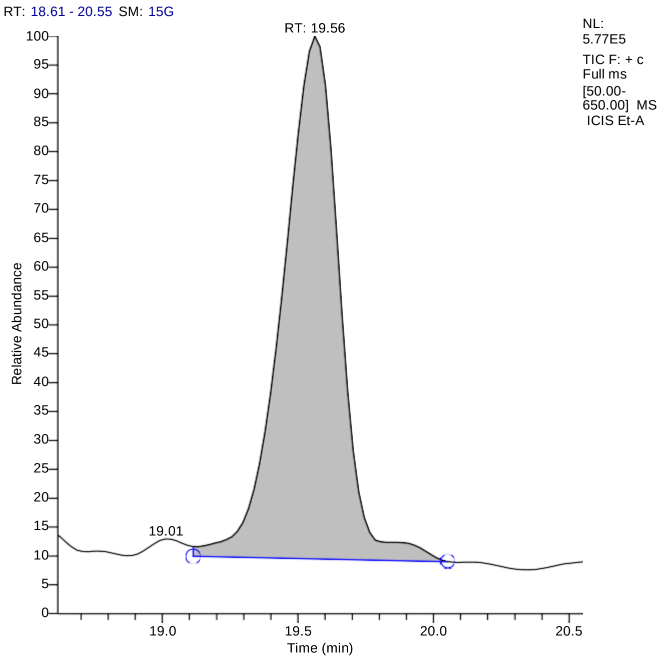

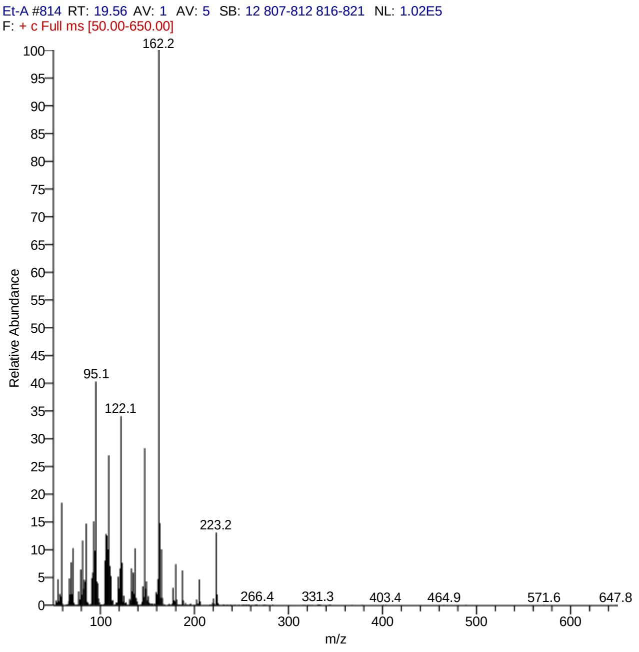

SI	RSI	Compound Name	Library	Probability	Area %	Area	RT
662	703	4,4-Dimethyladamantan-2-ol	MAINLIB	11.73	55.80	7714808.19	19.56
655	713	trans-8-Methyl-1á-acetyl-hydrindane	MAINLIB	8.99	55.80	7714808.19	19.56
644	697	cis-8-Methyl-1á-acetyl-hydrindane	MAINLIB	6.16	55.80	7714808.19	19.56

Hit Spectrum | Delta | Compound Structure

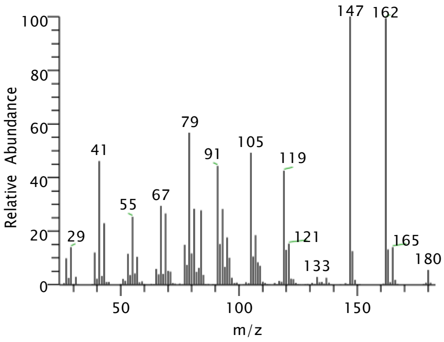

Raw data - Library entry

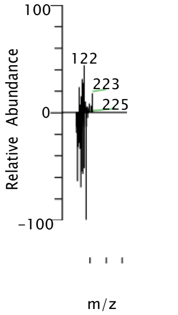

4,4-Dimethyladamantan-2-ol
Formula C12H20O, MW 180, CAS# NA, Entry# 94362

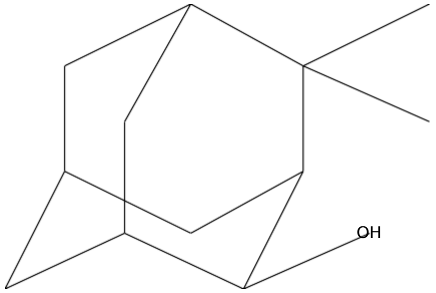

My Qual X-Report

Hit Spectrum	Delta	Compound Structure

Raw data - Library entry

trans-8-Methyl-1á-acetyl-hydrindane
Formula C12H20O, MW 180, CAS# 54832-11-0, Entry# 10295
1-(2-Methyloctahydro-3ah-inden-3a-yl)ethanone #

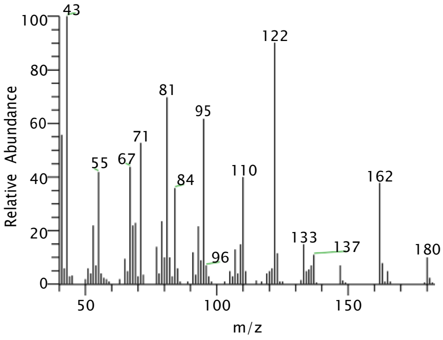

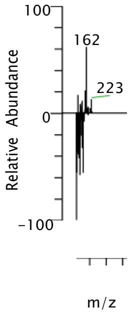

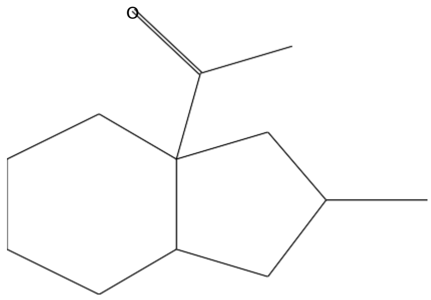

Raw data - Library entry

cis-8-Methyl-1á-acetyl-hydrindane
Formula C12H20O, MW 180, CAS# NA, Entry# 10284

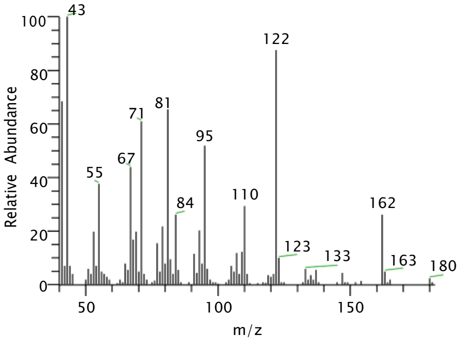

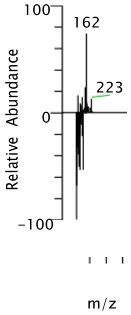

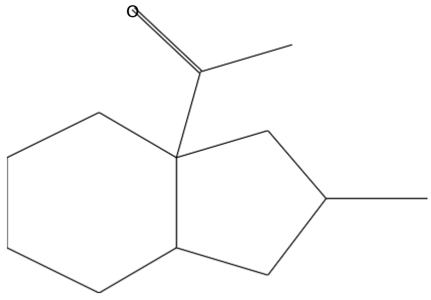

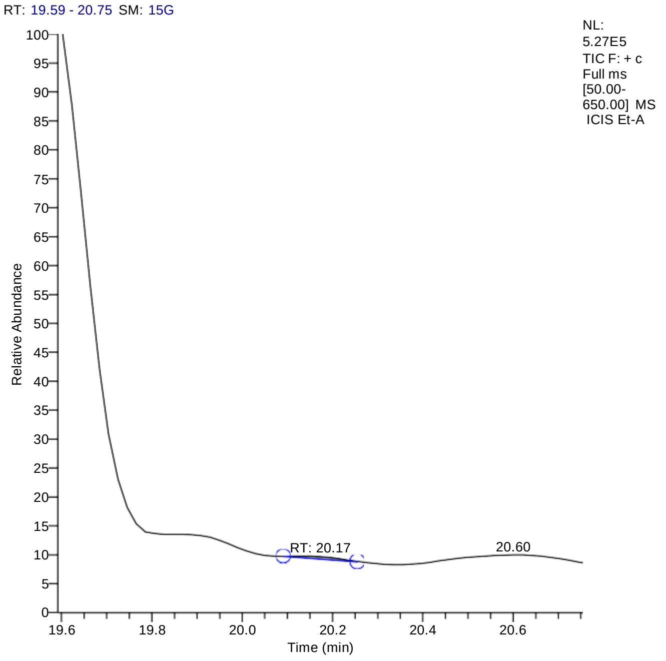

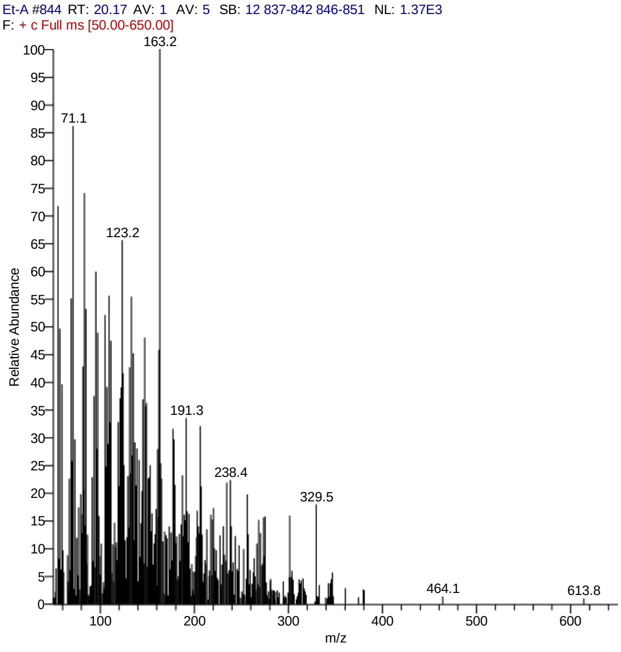

SI	RSI	Compound Name	Library	Probability	Area %	Area	RT
676	775	Androst-4-en-3-one, 9-fluoro-11,17-dihydroxy-17-methyl-, (11á,17á)-	replib	38.17	0.11	15537.61	20.17
652	702	Preg-4-en-3-one, 12,17-dihydroxy-20-nitrilo-	MAINLIB	12.79	0.11	15537.61	20.17
640	652	Gibbane-1,10-dicarboxylic acid, 2,3-epoxy-4a,7-dihydroxy-1-methyl-8-methylene-, 1,4a-lactone, 10-methyl ester, (1à,2á,3á,4aà,4bà,10á)-	MAINLIB	8.52	0.11	15537.61	20.17

My Qual X-Report

Hit Spectrum | Delta | Compound Structure

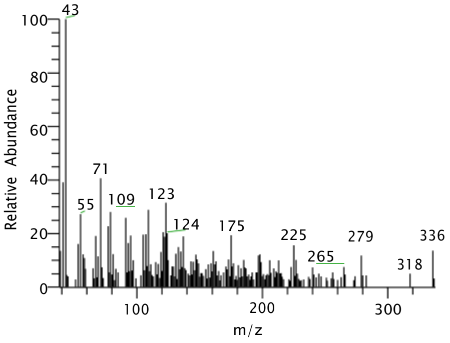

Raw data - Library entry

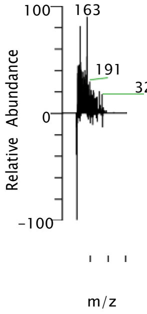

Androst-4-en-3-one, 9-fluoro-11,17-dihydroxy-17-methyl-, (11á,17á)-
Formula C20H29FO3, MW 336, CAS# 76-43-7, Entry# 2391
component of Halodrin

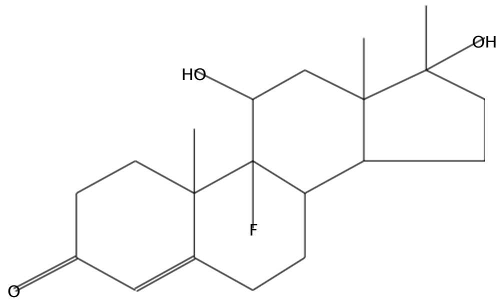

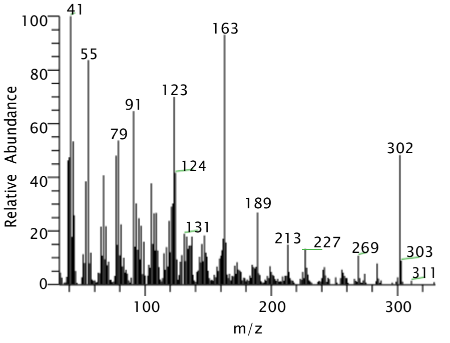

Raw data - Library entry

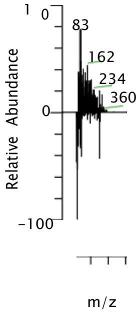

Preg-4-en-3-one, 12,17-dihydroxy-20-nitrilo-
Formula C20H27NO3, MW 329, CAS# NA, Entry# 3728

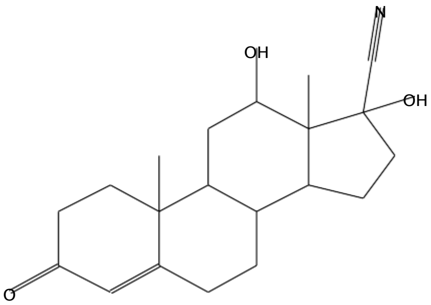

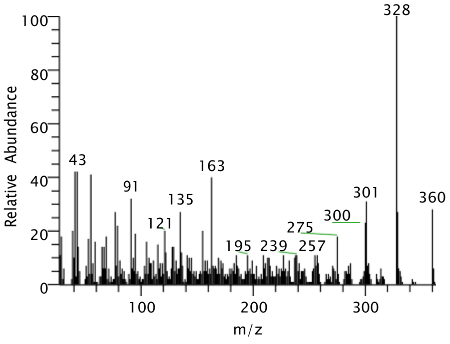

Raw data - Library entry

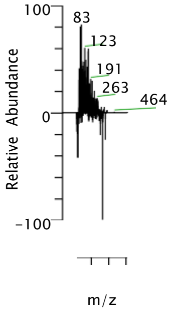

Formula C20H24O6, MW 360, CAS# 22882-57-1, Entry# 155090

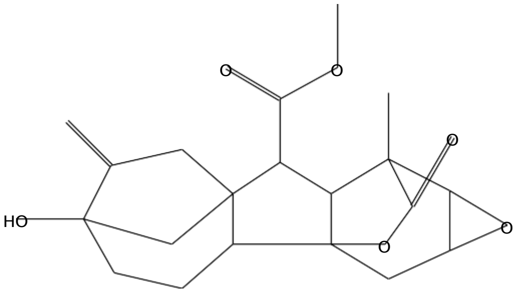

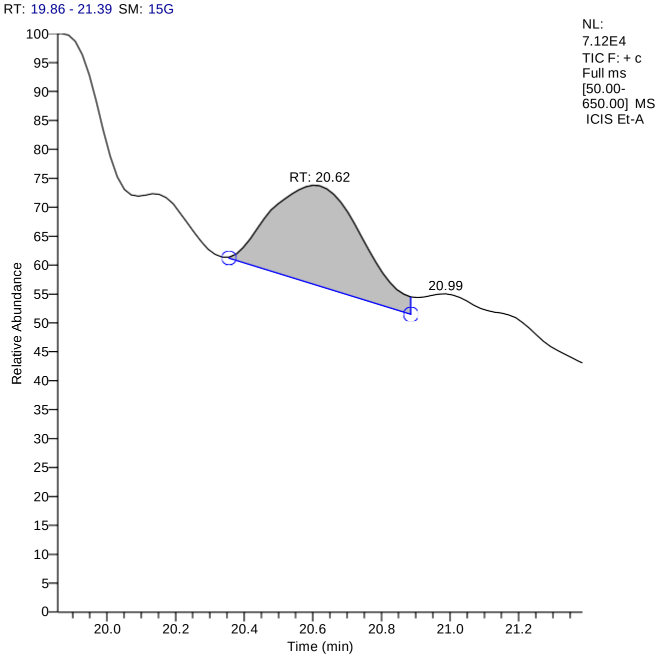

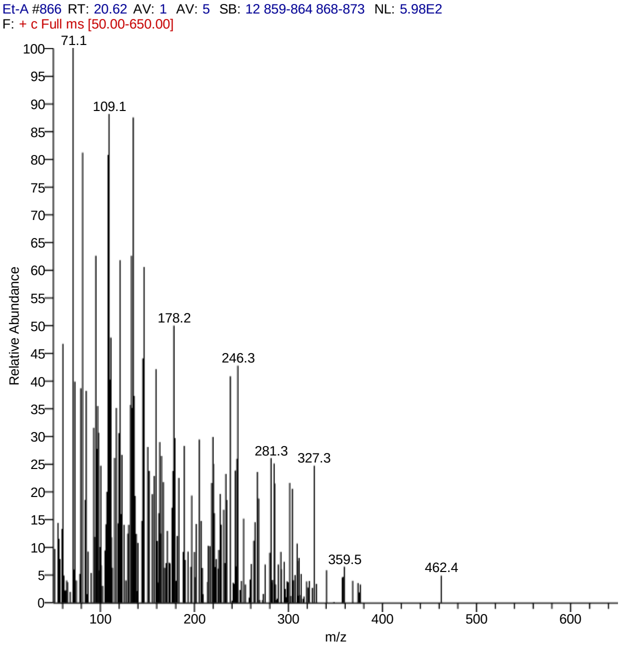

My Qual X-Report

SI	RSI	Compound Name	Library	Probability	Area %	Area	RT
525	544	Cholan-24-oic acid, 3-(acetyloxy)-7,12-dioxo-, methyl ester, (3à,5á)-	MAINLIB	30.72	1.65	227835.71	20.62
506	570	2-Pentenoic acid, 5-(decahydro-5,5,8a-trimethyl-2-methyl ene-1-naphthalenyl)-3-methyl-, [1S-[1à(E),4aá,8aà]]-	MAINLIB	14.91	1.65	227835.71	20.62
499	608	5à-Androstan-17á-ol, 2á,3á-epoxy-3-methyl-	MAINLIB	11.42	1.65	227835.71	20.62

Hit Spectrum | Delta | Compound Structure

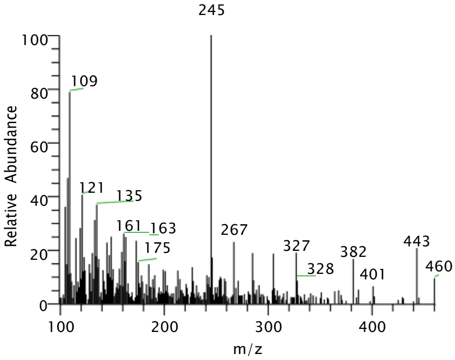

Raw data - Library entry

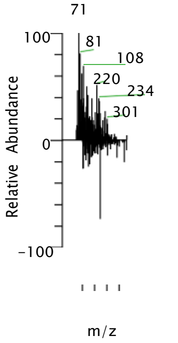

Cholan-24-oic acid, 3-(acetyloxy)-7,12-dioxo-, methyl ester, (3à,5á)-
Formula C27H40O6, MW 460, CAS# 7753-73-3, Entry# 139165
Methyl 3-(acetyloxy)-7,12-dioxocholan-24-oate #

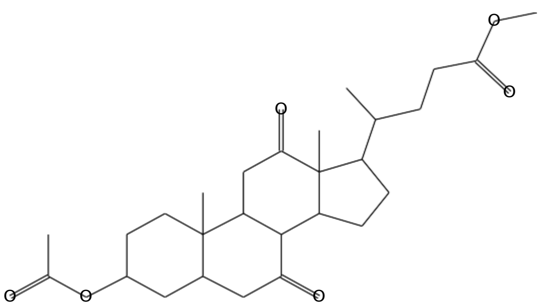

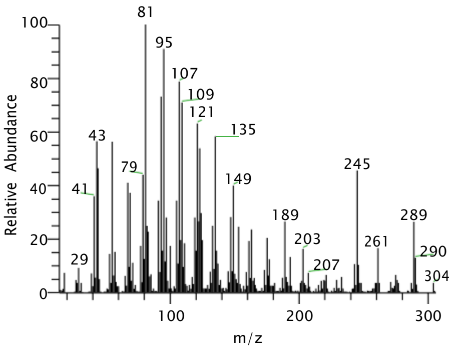

Raw data - Library entry

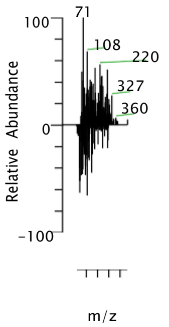

Formula C20H32O2, MW 304, CAS# 24470-48-2, Entry# 39949
Labda-8(20),13-dien-15-oic acid, (E)-(+)-

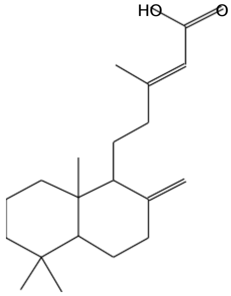

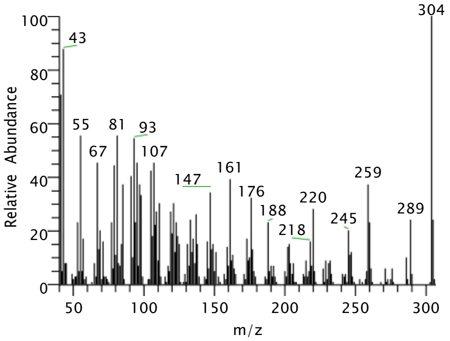

Raw data - Library entry

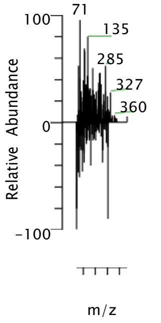

5à-Androstan-17á-ol, 2á,3á-epoxy-3-methyl-
Formula C20H32O2, MW 304, CAS# 16394-67-5, Entry# 151887
6a,8a,10a-Trimethylhexadecahydro-1H-cyclopenta[7,8]phenanthro[2,3-b]oxiren-1-ol #

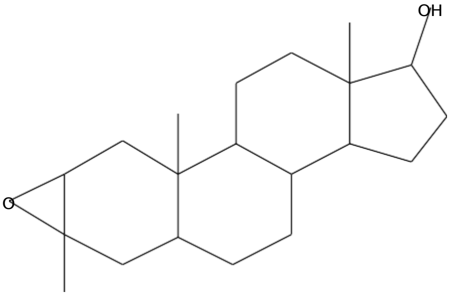

My Qual X-Report

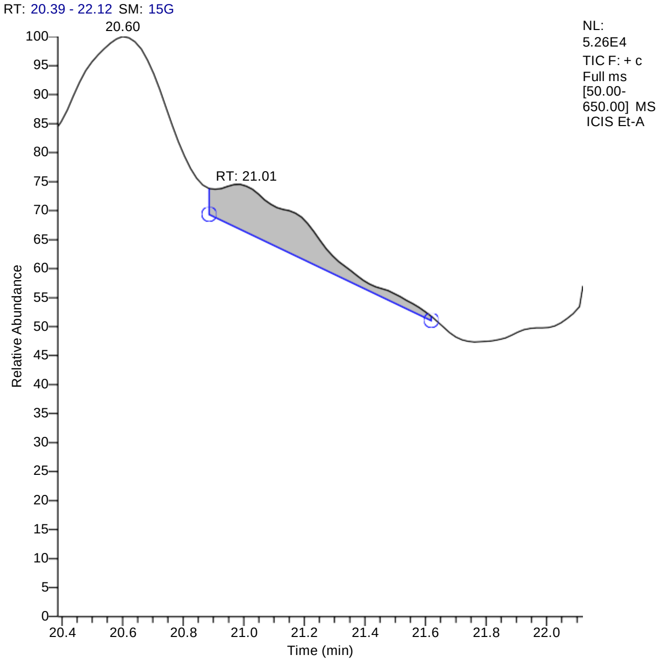

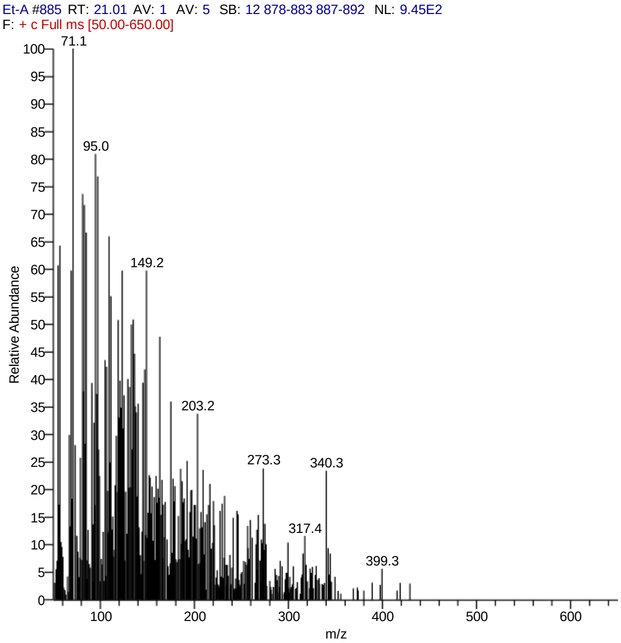

SI	RSI	Compound Name	Library	Probability	Area %	Area	RT
658	705	1-Naphthalenecarboxylic acid, 5-(4-carboxy-3-methyl-3-butenyl)decahydro-1,4a-dimethyl-6-methylene-, [1S-[1à,4aà,5à(E),8aá]]-	MAINLIB	14.05	0.73	100722.76	21.01
654	712	Dasycarpidan-1-methanol, acetate (ester)	MAINLIB	11.86	0.73	100722.76	21.01
649	653	3-Methoxymethoxy-3,7,16,20-tetramethyl-heneicosa-1,7,11,15,19-pentaene	MAINLIB	9.56	0.73	100722.76	21.01

Hit Spectrum	Delta	Compound Structure

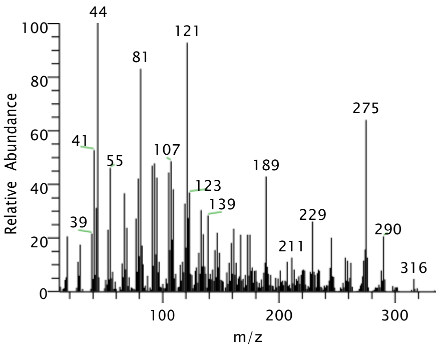

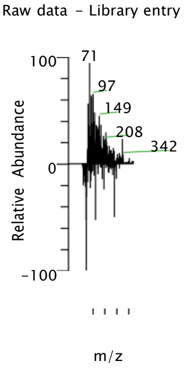

Formula C20H30O4, MW 334, CAS# 640-28-8, Entry# 14110
Labda-8(20)-13-diene-15,19-dioic acid, (E)-

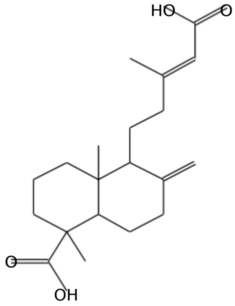

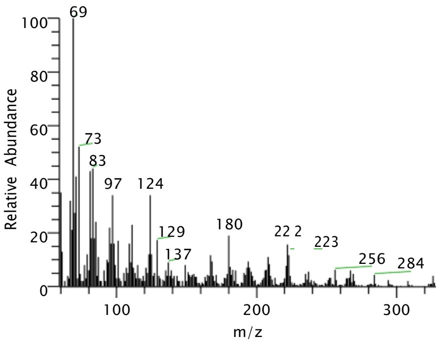

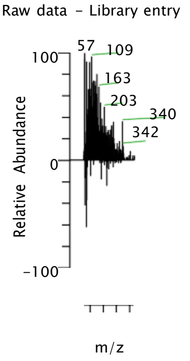

Dasycarpidan-1-methanol, acetate (ester)
Formula C20H26N2O2, MW 326, CAS# 55724-48-6, Entry# 28935

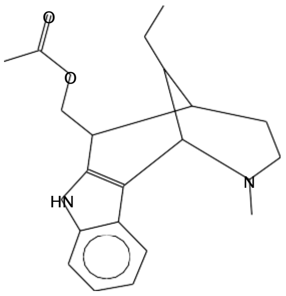

My Qual X-Report

Hit Spectrum | Delta | Compound Structure

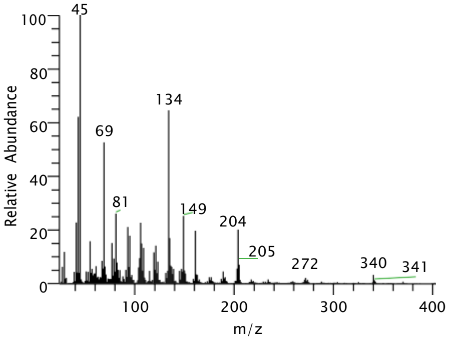

Raw data - Library entry

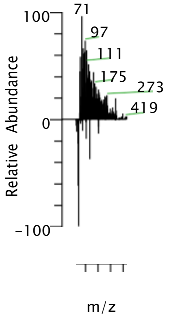

3-Methoxymethoxy-3,7,16,20-tetramethyl-heneicosa-1,7,11,15,19-pentaene
Formula C27H46O2, MW 402, CAS# NA, Entry# 15311

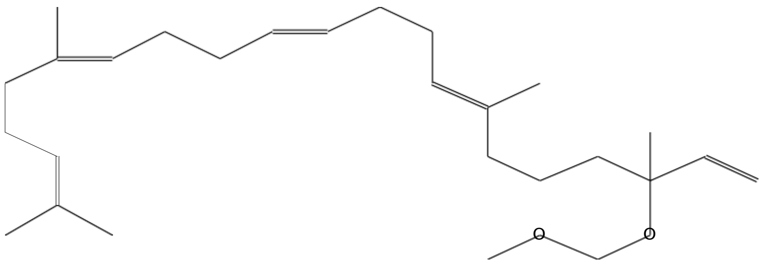

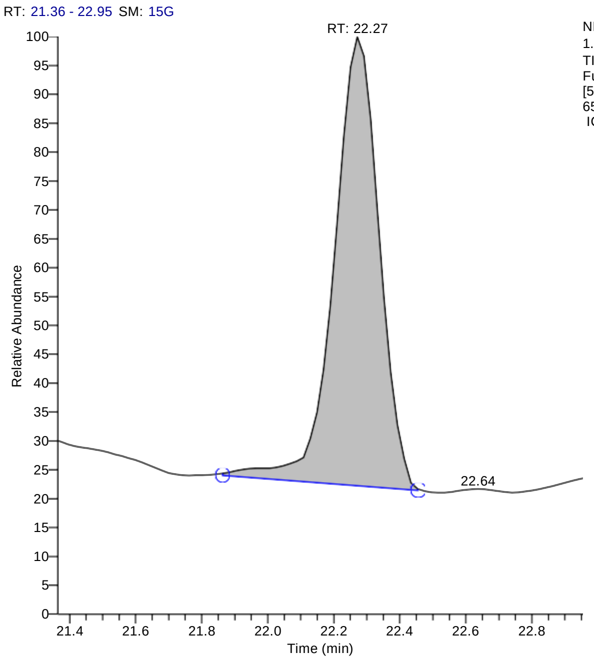

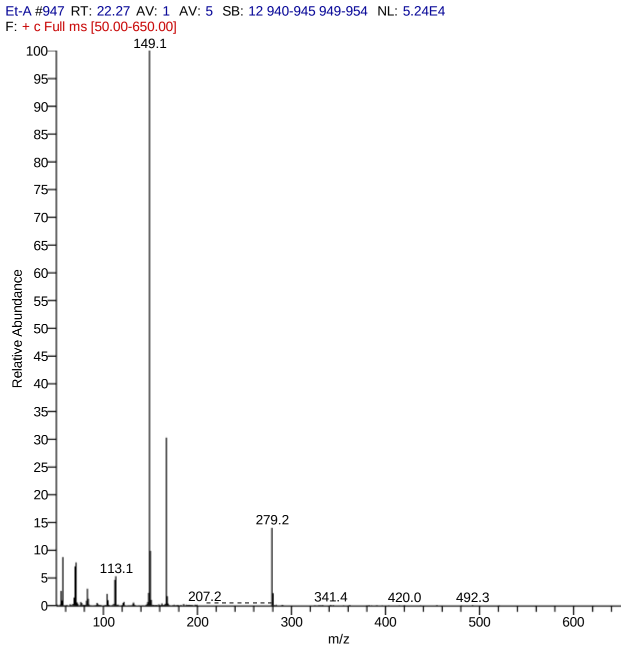

SI	RSI	Compound Name	Library	Probability	Area %	Area	RT
865	884	1,2-Benzenedicarboxylic acid, diisooctyl ester	replib	33.62	5.58	771874.01	22.27
853	866	1,2-Benzenedicarboxylic acid, mono(2-ethylhexyl) ester	MAINLIB	22.39	5.58	771874.01	22.27
852	860	1,2-Benzenedicarboxylic acid, diisooctyl ester	MAINLIB	33.62	5.58	771874.01	22.27

Hit Spectrum | Delta | Compound Structure

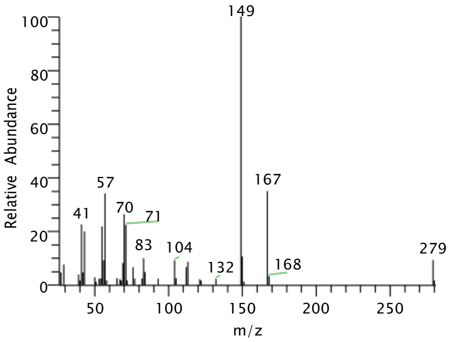

Raw data - Library entry

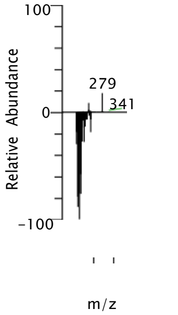

1,2-Benzenedicarboxylic acid, diisooctyl ester
Formula C24H38O4, MW 390, CAS# 27554-26-3, Entry# 19804
Diisooctyl phthalate

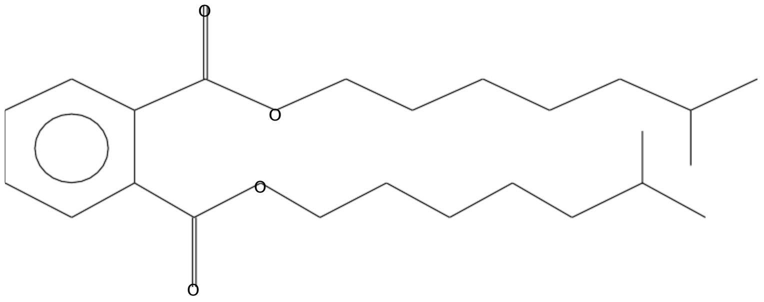

My Qual X-Report

Hit Spectrum | Delta | Compound Structure

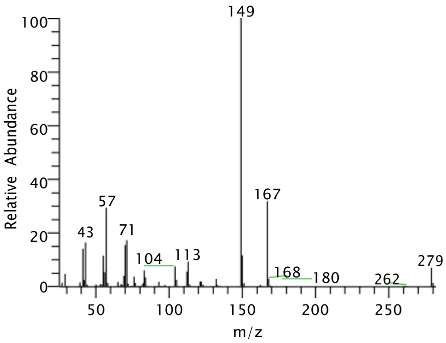

Raw data - Library entry

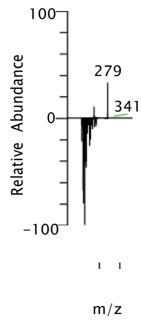

1,2-Benzenedicarboxylic acid, mono(2-ethylhexyl) ester
Formula C16H22O4, MW 278, CAS# 4376-20-9, Entry# 96269
Mono(2-ethylhexyl) phthalate

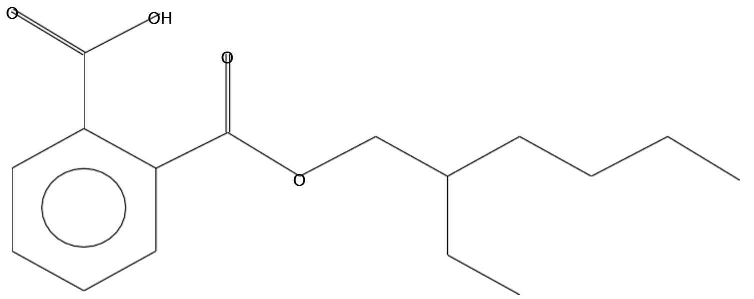

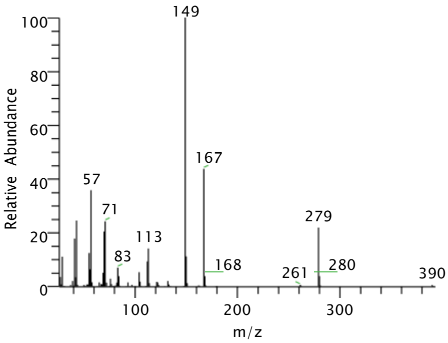

Raw data - Library entry

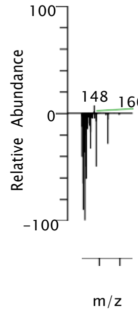

1,2-Benzenedicarboxylic acid, diisooctyl ester
Formula C24H38O4, MW 390, CAS# 27554-26-3, Entry# 96271
Diisooctyl phthalate

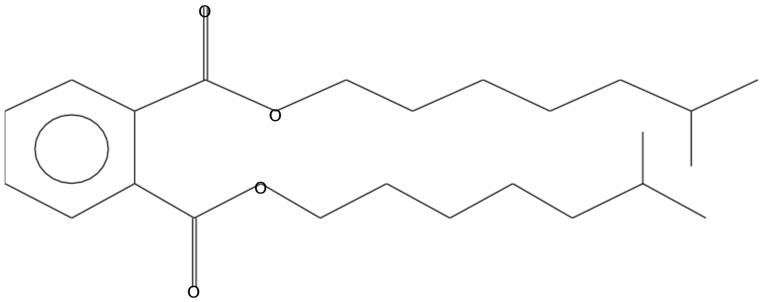

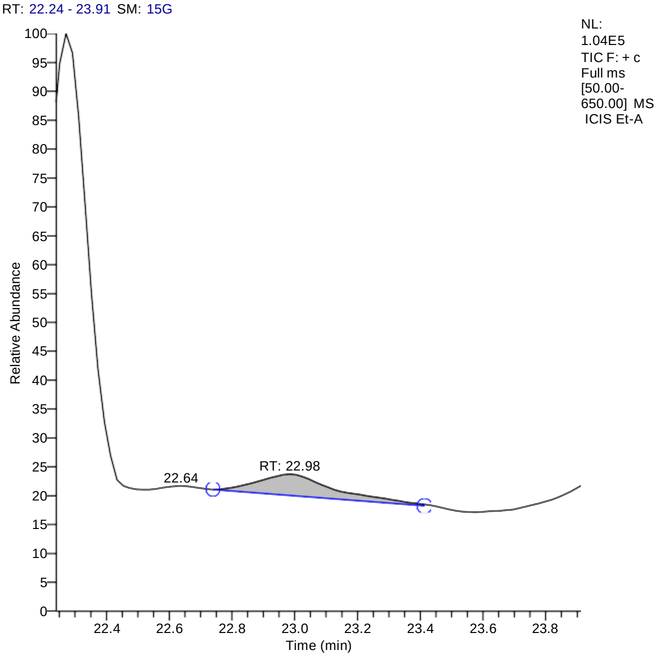

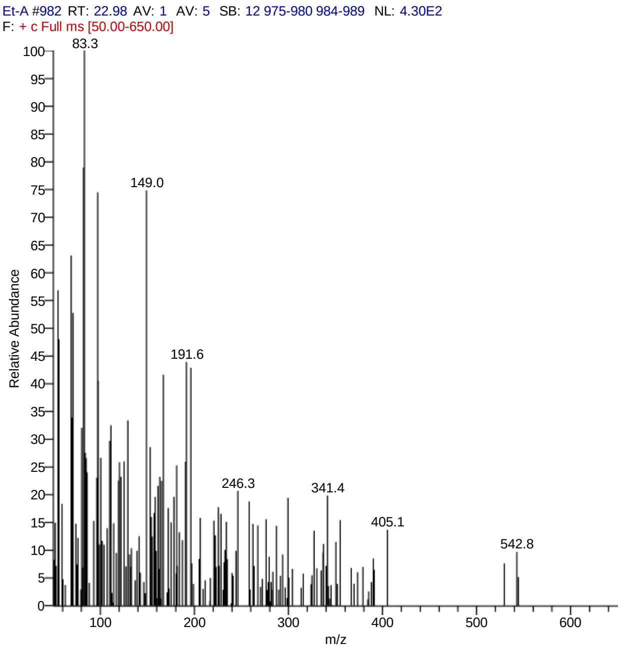

SI	RSI	Compound Name	Library	Probability	Area %	Area	RT
401	486	2,7-Diphenyl-1,6-dioxopyridazino[4,5:2',3']pyrrolo[4',5'-d]pyridazine	MAINLIB	6.47	0.48	66194.59	22.98
394	408	5H-Cyclopropa[3,4]benz[1,2-e]azulen-5-one, 9,9a-bis(acetyloxy)-1,1a,1b,2,4a,7a,7b,8,9,9a-decahydro-2,4a,7b-trihydroxy-3-(hydroxymethyl)-1,1,6,8-tetramethyl-, [1aR-(1aà,1bá,2á,4aá,7aà,7bà,8à,9á,9aà)]-	MAINLIB	4.96	0.48	66194.59	22.98

My Qual X-Report

SI	RSI	Compound Name	Library	Probability	Area %	Area	RT
393	414	5aH-3a,12-Methano-1H-cyclopropa[5',6']cyclodeca[1',2':1,5]cyclopenta[1,2-d][1,3]dioxol-13-one, 1a,2,3,9,12,12a-hexahydro-9-hydroxy-10-(hydroxymethyl)-1,1,3,5,7,7-hexamethyl-, [1aR-(1aà,3à,3aà,5aà,8aR*,9á,12à,12aà)]-	MAINLIB	4.76	0.48	66194.59	22.98

Hit Spectrum	Delta	Compound Structure

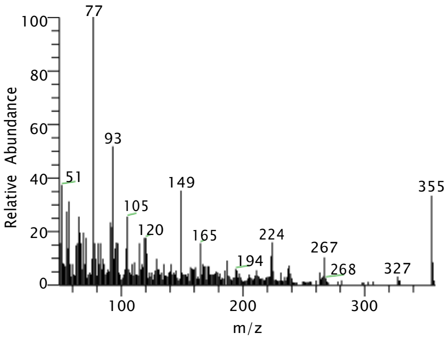

Raw data - Library entry

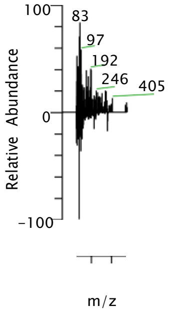

2,7-Diphenyl-1,6-dioxopyridazino[4,5:2',3']pyrrolo[4',5'-d]pyridazine
Formula C20H13N5O2, MW 355, CAS# 91757-06-1, Entry# 36966

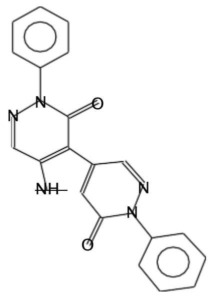

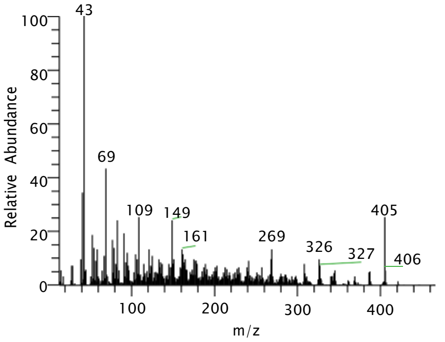

Raw data - Library entry

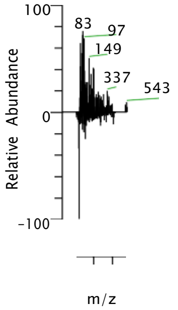

Formula C24H32O9, MW 464, CAS# 77573-19-4, Entry# 7601

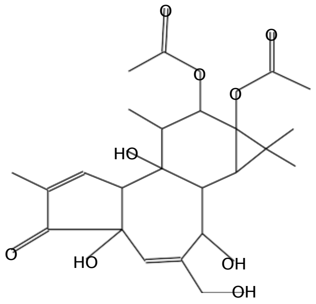

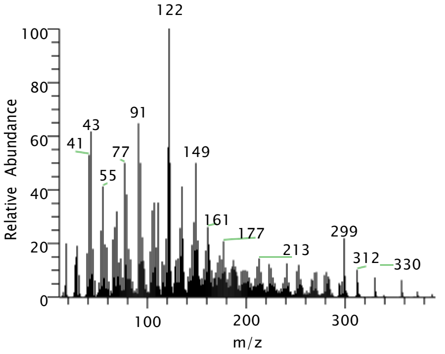

Raw data - Library entry

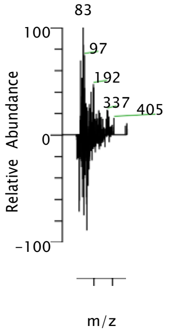

Formula C23H32O5, MW 388, CAS# 77520-49-1, Entry# 75261

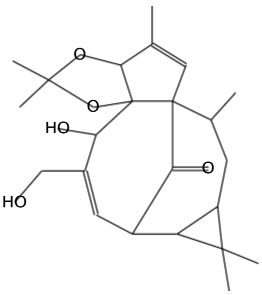

My Qual X-Report

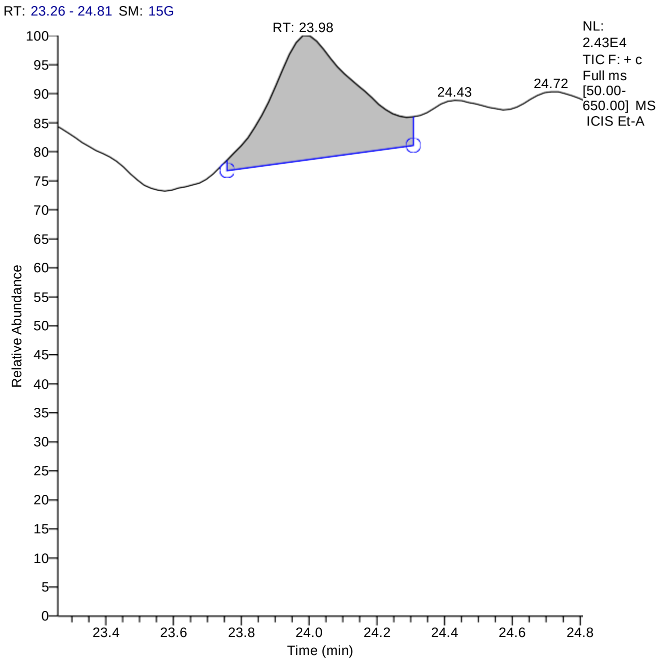

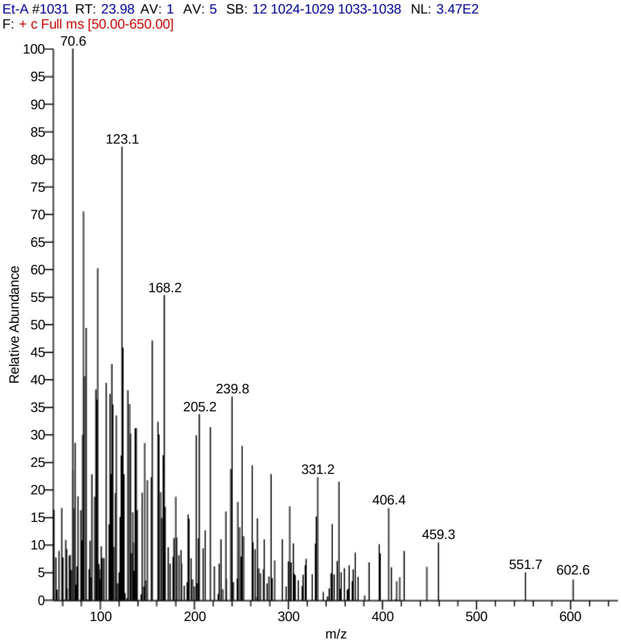

SI	RSI	Compound Name	Library	Probability	Area %	Area	RT
432	460	9-Octadecene, 1-[2-(octadecyloxy)ethoxy]-	MAINLIB	10.97	0.67	93114.66	23.98
414	445	Butanoic acid, 1a,2,5,5a,6,9,10,10a-octahydro-5,5a-dihydroxy-4-(hydroxymethyl)-1,1,7,9-tetramethyl-11-oxo-1H-2,8a-methanocyclopenta[a]cyclopropa[e]cyclodecen-6-yl ester, [1aR-(1aà,2à,5á,5aá,6á,8aà,9à,10aà)]-	MAINLIB	5.66	0.67	93114.66	23.98
400	468	4-[4-Chloroanilino]-6-methoxy-8-nitroquinoline	MAINLIB	3.54	0.67	93114.66	23.98

Hit Spectrum | Delta | Compound Structure

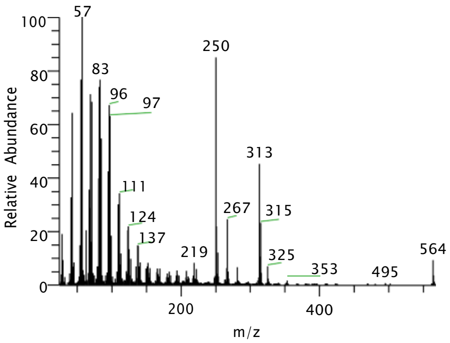

Raw data - Library entry

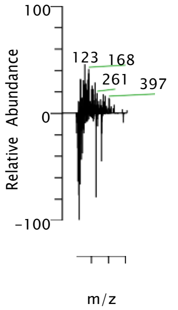

9-Octadecene, 1-[2-(octadecyloxy)ethoxy]-
Formula C38H76O2, MW 564, CAS# 56599-41-8, Entry# 22918
(9E)-1-[2-(Octadecyloxy)ethoxy]-9-octadecene #

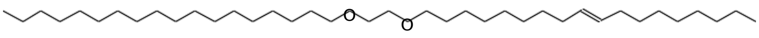

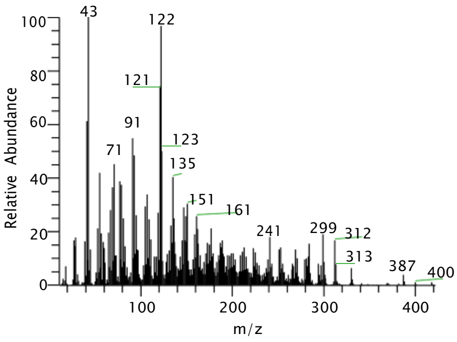

Raw data - Library entry

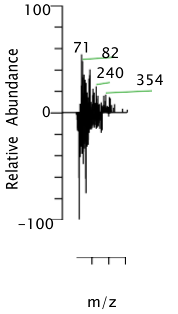

Formula C24H34O6, MW 418, CAS# 77508-67-9, Entry# 10305

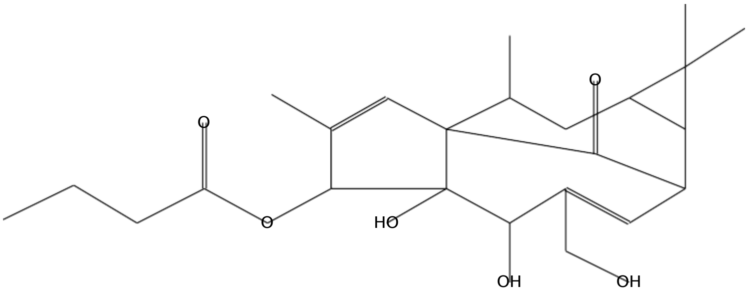

My Qual X-Report

Hit Spectrum	Delta	Compound Structure

Raw data - Library entry

4-[4-Chloroanilino]-6-methoxy-8-nitroquinoline
Formula C16H12ClN3O3, MW 329, CAS# 63456-81-5, Entry# 155306
N-(4-Chlorophenyl)-6-methoxy-8-nitro-4-quinolinamine #

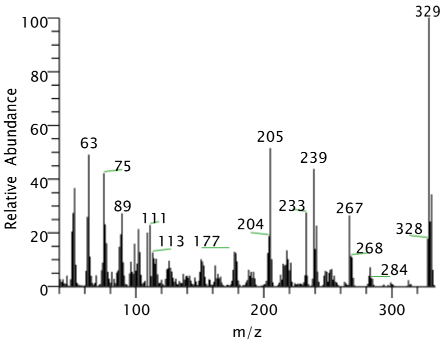

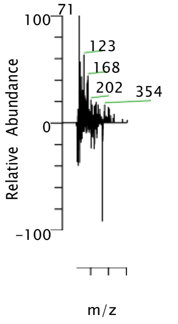

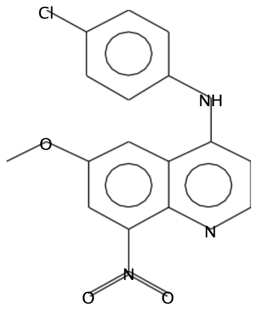

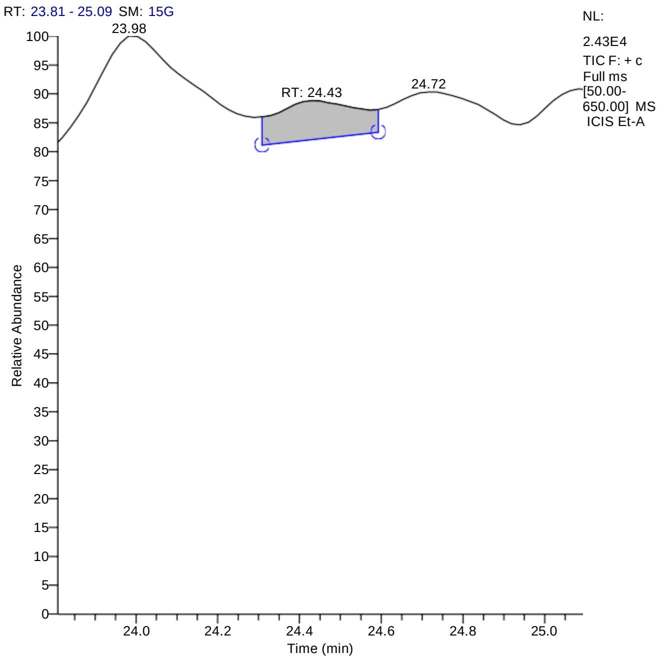

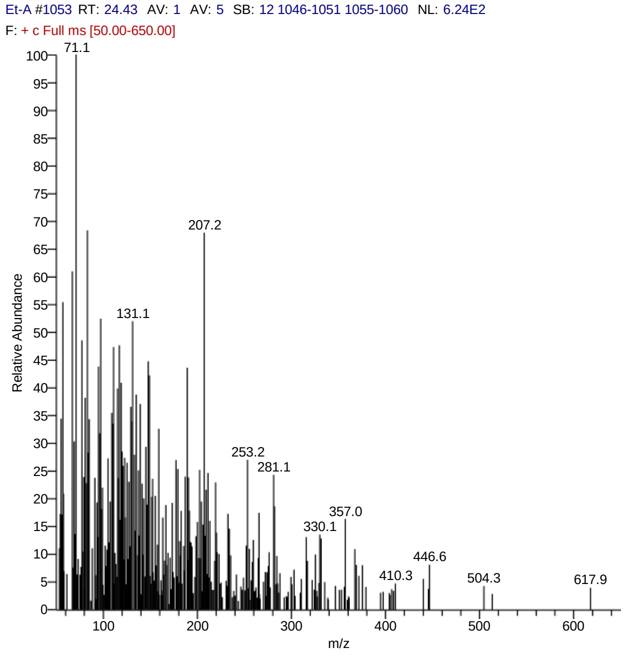

SI	RSI	Compound Name	Library	Probability	Area %	Area	RT
638	664	5aH-3a,12-Methano-1H-cyclopropa[5',6']cyclodeca[1',2':1,5]cyclopenta[1,2-d][1,3]dioxol-13-one, 1a,2,3,9,12,12a-hexahydro-9-hydroxy-10-(hydroxymethyl)-1,1,3,5,7,7-hexamethyl-, [1aR-(1aà,3à,3aà,5aà,8aR*,9á,12à,12aà)]-	MAINLIB	45.27	0.17	23026.54	24.43
607	617	Betulin	replib	12.34	0.17	23026.54	24.43
591	693	9,10-Secocholesta-5,7,10(19)-triene-3,24,25-triol, (3á,5Z,7E)-	MAINLIB	7.10	0.17	23026.54	24.43

Hit Spectrum	Delta	Compound Structure

Raw data - Library entry

Formula C23H32O5, MW 388, CAS# 77520-49-1, Entry# 75261

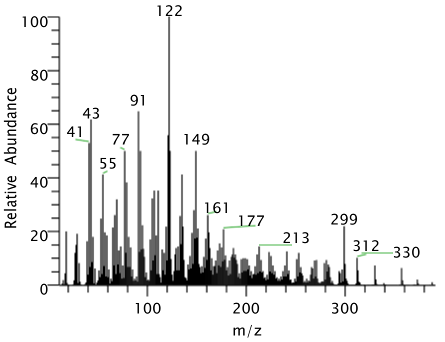

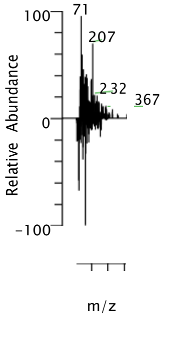

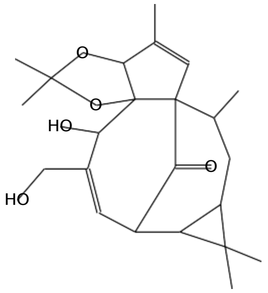

My Qual X-Report

Hit Spectrum | Delta | Compound Structure

SI 607, RSI 617, replib, Entry# 23168, CAS# 473-98-3, Betulin

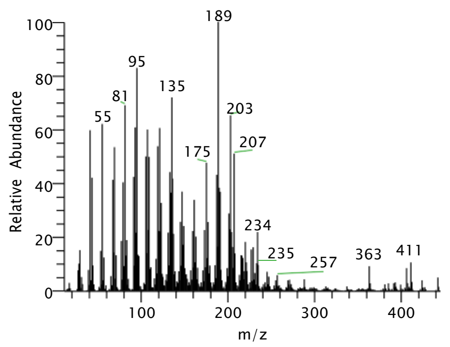

Raw data - Library entry

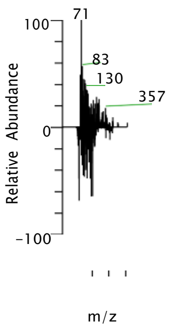

Betulin
Formula C30H50O2, MW 442, CAS# 473-98-3, Entry# 23168
Lup-20(29)-ene-3,28-diol, (3á)-

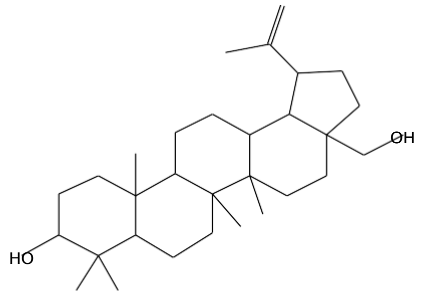

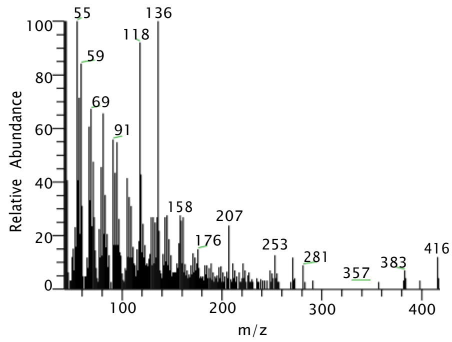

Raw data - Library entry

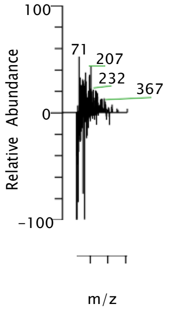

9,10-Secocholesta-5,7,10(19)-triene-3,24,25-triol, (3á,5Z,7E)-
Formula C27H44O3, MW 416, CAS# 40013-87-4, Entry# 6060
24,25-Dihydroxycholecalciferol

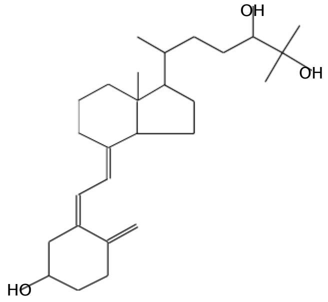

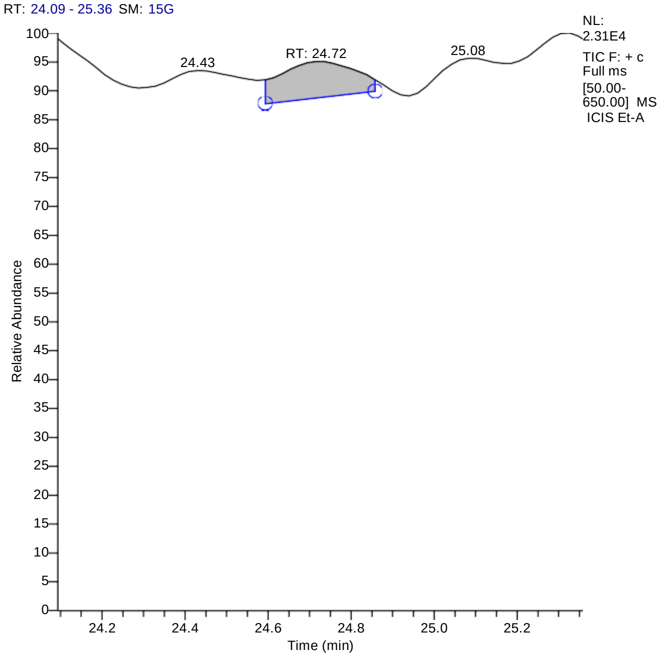

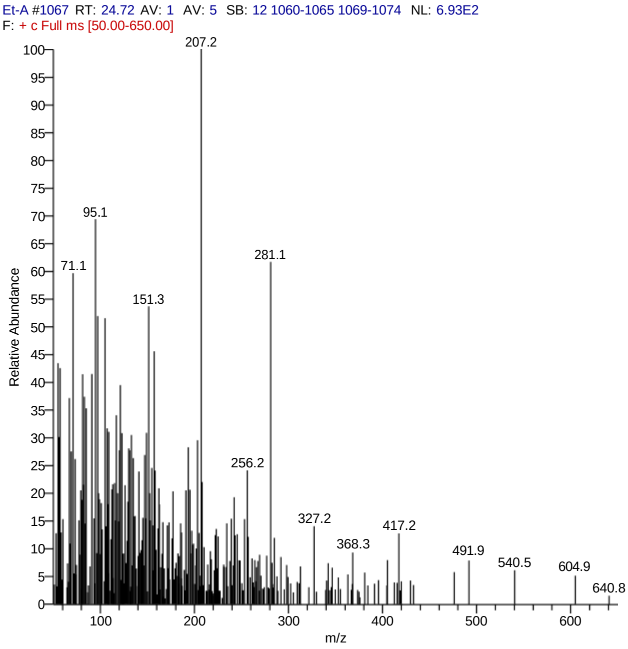

SI	RSI	Compound Name	Library	Probability	Area %	Area	RT
614	710	9,10-Secocholesta-5,7,10(19)-triene-3,24,25-triol, (3á,5Z,7E)-	MAINLIB	28.81	0.13	18258.01	24.72
613	633	9-Desoxo-9-x-acetoxy-3,8,12-tri-O-acetylingol	MAINLIB	27.69	0.13	18258.01	24.72
587	661	9,12,15-Octadecatrienoic acid, 2,3-bis[(trimethylsilyl)oxy]propyl ester, (Z,Z,Z)-	replib	8.29	0.13	18258.01	24.72

My Qual X-Report

Hit Spectrum	Delta	Compound Structure

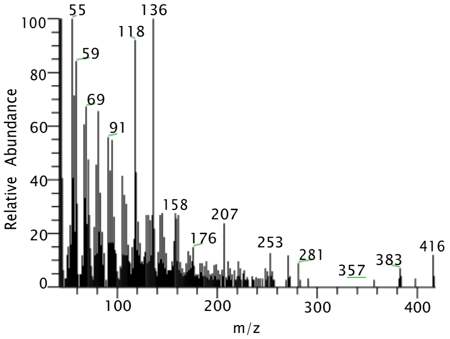

Raw data – Library entry

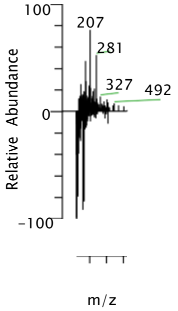

9,10-Secocholesta-5,7,10(19)-triene-3,24,25-triol, (3á,5Z,7E)-
Formula C27H44O3, MW 416, CAS# 40013-87-4, Entry# 6060
24,25-Dihydroxycholecalciferol

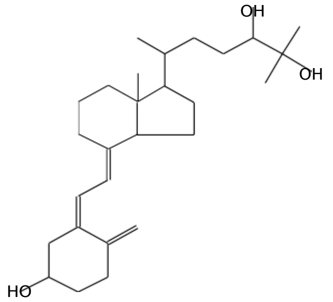

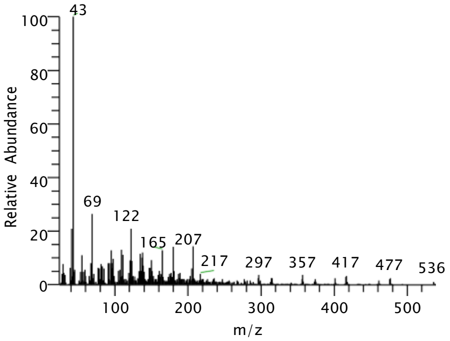

Raw data – Library entry

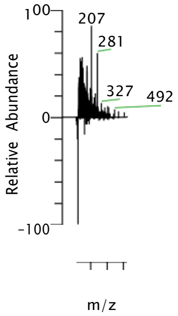

9-Desoxo-9-x-acetoxy-3,8,12-tri-O-acetylingol
Formula C28H40O10, MW 536, CAS# NA, Entry# 7587

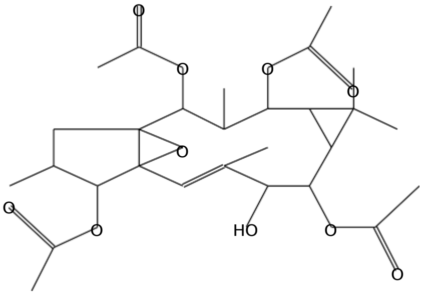

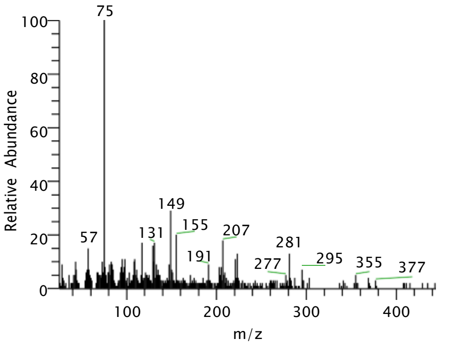

Raw data – Library entry

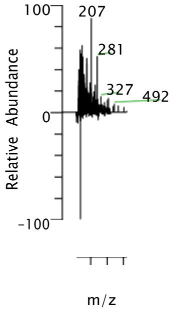

9,12,15-Octadecatrienoic acid, 2,3-bis[(trimethylsilyl)oxy]propyl ester, (Z,Z,Z)-
Formula C27H52O4Si2, MW 496, CAS# 55521-22-7, Entry# 9235
2,3-Bis[(trimethylsilyl)oxy]propyl (9E,12E,15E)-9,12,15-octadecatrienoate #

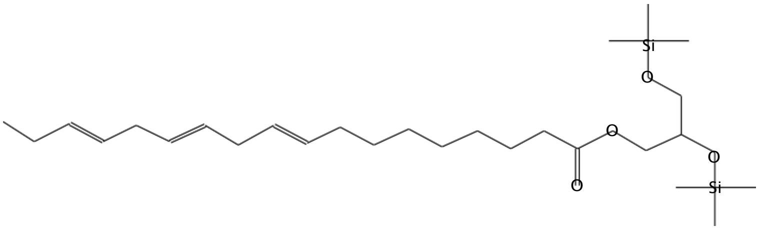

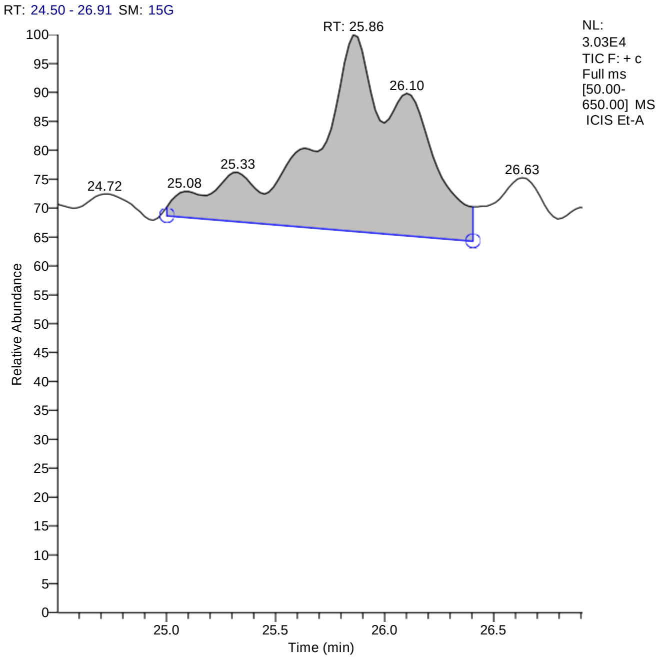

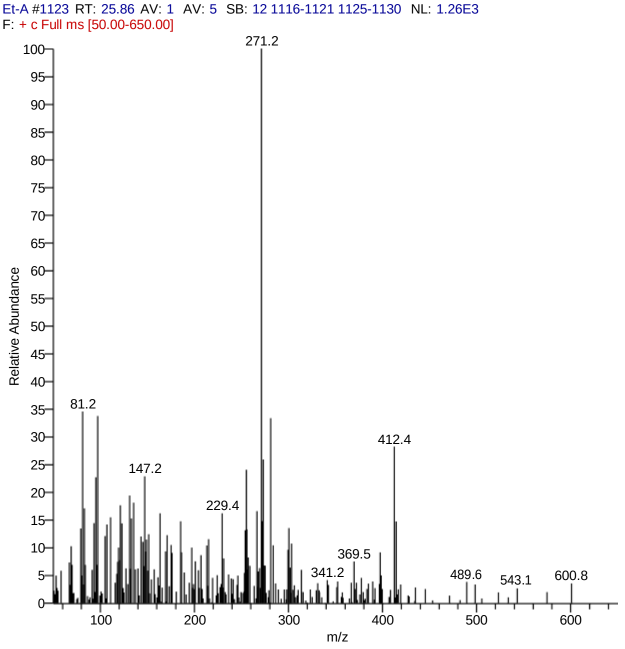

SI	RSI	Compound Name	Library	Probability	Area %	Area	RT
564	589	Stigmasterol	replib	25.51	2.47	341146.40	25.86

My Qual X-Report

SI	RSI	Compound Name	Library	Probability	Area %	Area	RT
537	634	Stigmasterol	replib	25.51	2.47	341146.40	25.86
535	616	28,33-Dinorgorgost-5-en-24-one, 3-hydroxy-, (3á)-	MAINLIB	7.23	2.47	341146.40	25.86

Hit Spectrum | Delta | Compound Structure

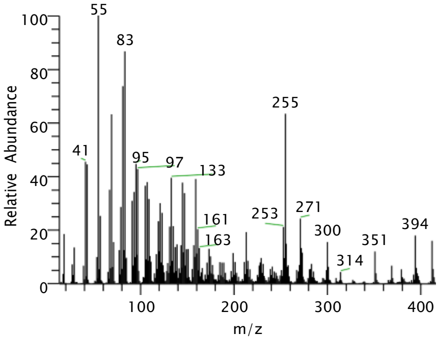

Raw data - Library entry

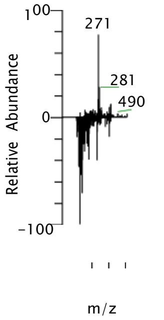

Stigmasterol
Formula C29H48O, MW 412, CAS# 83-48-7, Entry# 4606
Stigmasta-5,22-dien-3-ol, (3á,22E)-

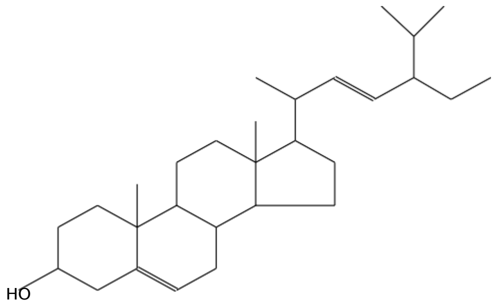

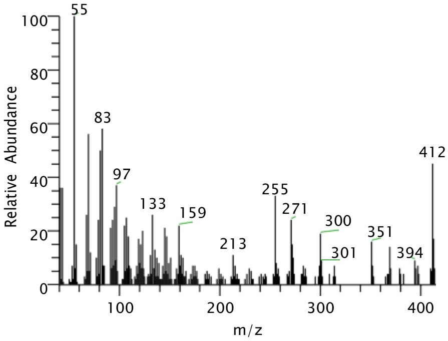

Raw data - Library entry

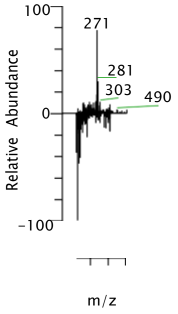

Stigmasterol
Formula C29H48O, MW 412, CAS# 83-48-7, Entry# 4602
Stigmasta-5,22-dien-3-ol, (3á,22E)-

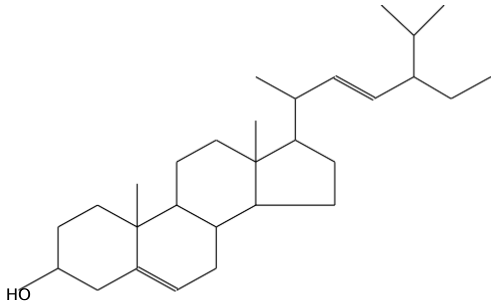

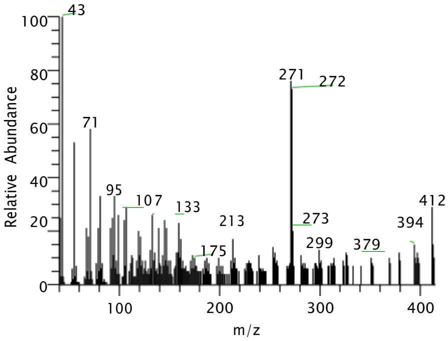

Raw data - Library entry

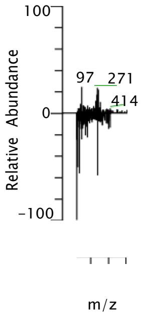

28,33-Dinorgorgost-5-en-24-one, 3-hydroxy-, (3á)-
Formula C28H44O2, MW 412, CAS# 55064-61-4, Entry# 12488
3á-Hydroxy-22,23-methylenecholest-5-en-24-one

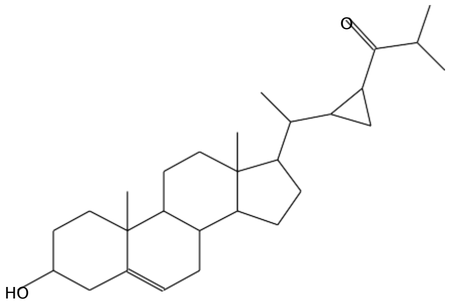

My Qual X-Report

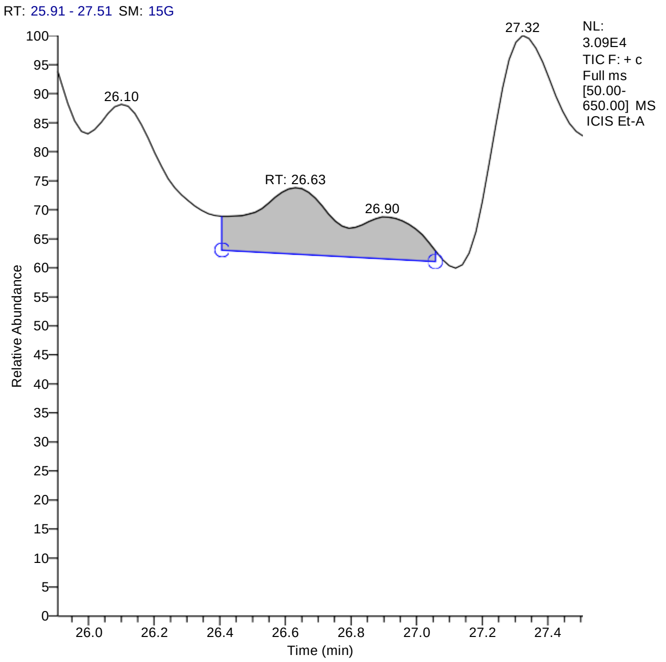

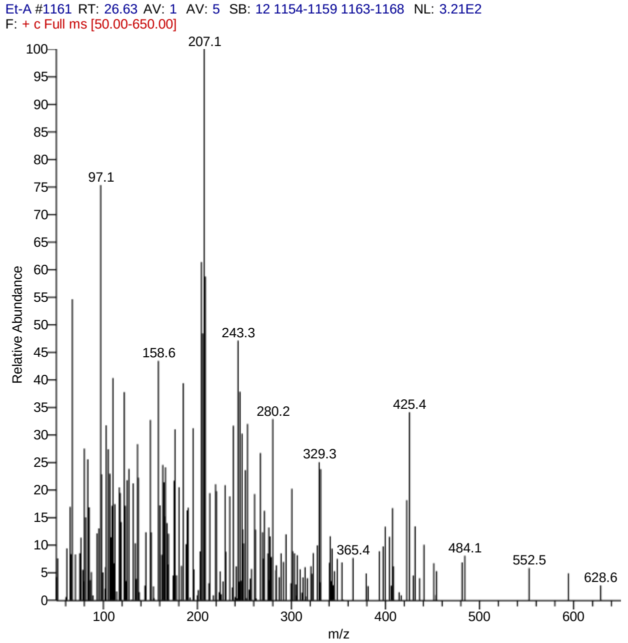

SI	RSI	Compound Name	Library	Probability	Area %	Area	RT
404	453	Bicyclo[2.2.1]heptane,2,2,3,5,5-pentachloro-7,7-bis(chloromethyl)-1-dichoromethyl-	MAINLIB	18.06	0.63	86961.30	26.63
383	418	20.Xi.-Lanosta-7,9(11)-diene-3á,18,20-triol	MAINLIB	7.68	0.63	86961.30	26.63
374	429	Bornane, 2,2,5-endo,6-exo,8,9,10-heptachloro-	MAINLIB	5.58	0.63	86961.30	26.63

Hit Spectrum | Delta | Compound Structure

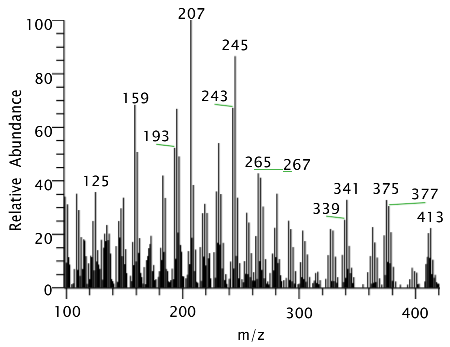

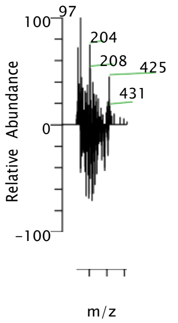

Bicyclo[2.2.1]heptane,2,2,3,5,5-pentachloro-7,7-bis(chloromethyl)-1-dichoromethyl-
Formula C10H9Cl9, MW 444, CAS# 165820-20-2, Entry# 126853
2,2,3,5,5-Pentachloro-7,7-bis(chloromethyl)-1-(dichloromethyl)bicyclo[2.2.1]heptane #

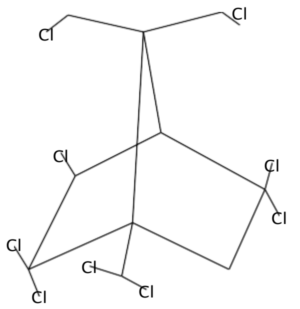

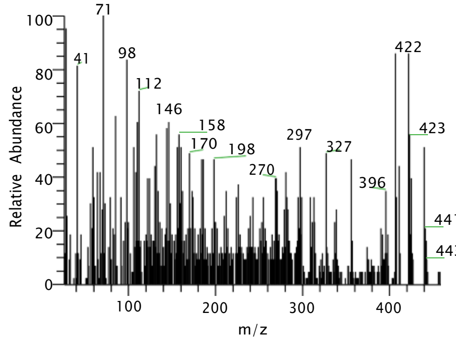

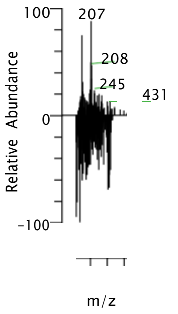

20.Xi.-Lanosta-7,9(11)-diene-3á,18,20-triol
Formula C30H50O3, MW 458, CAS# 25116-58-9, Entry# 30664
Lanosta-7,9(11)-diene-3,18,20-triol #

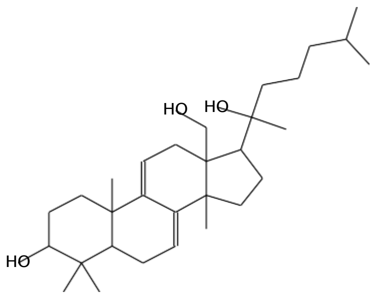

My Qual X-Report

Hit Spectrum | Delta | Compound Structure

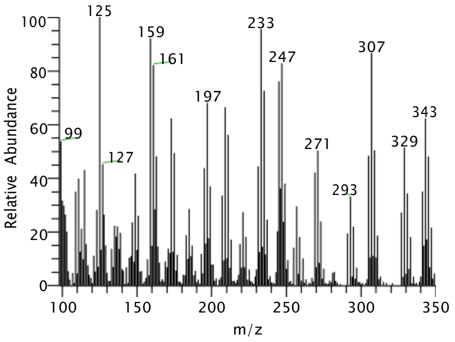

Raw data - Library entry

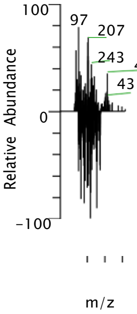

Bornane, 2,2,5-endo,6-exo,8,9,10-heptachloro-
Formula C10H11Cl7, MW 376, CAS# 51775-36-1, Entry# 78642
Bicyclo(2.2.1)heptane, 2,2,5,6-tetrachloro-1,7,7-tris(chloromethyl)-, (5-endo,6-exo)-

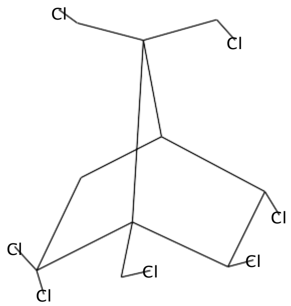

RT: 26.66 - 27.51 SM: 15G

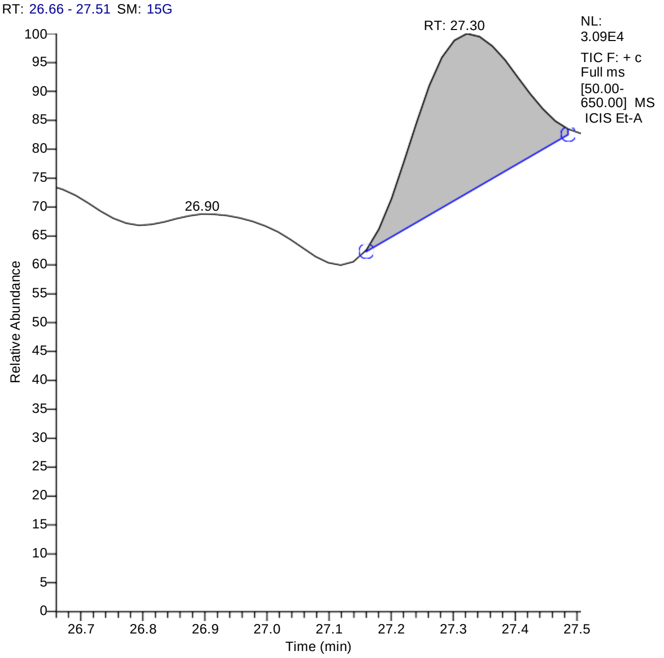

Et-A #1194 RT: 27.30 AV: 1 AV: 5 SB: 12 1187-1192 1196-1201 NL: 5.48E2
F: + c Full ms [50.00-650.00]

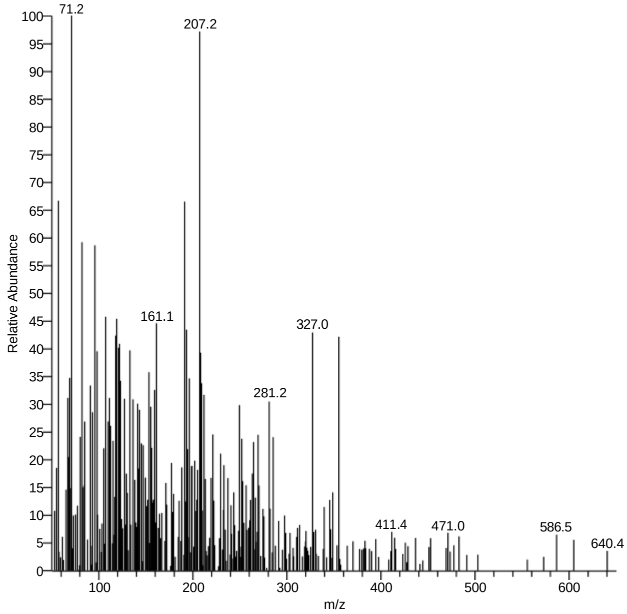

SI	RSI	Compound Name	Library	Probability	Area %	Area	RT
477	578	9,12,15-Octadecatrienoic acid, 2,3-bis[(trimethylsilyl)oxy]propyl ester, (Z,Z,Z)-	replib	28.21	0.68	93506.21	27.30
439	464	9-Octadecene, 1-[2-(octadecyloxy)ethoxy]-	MAINLIB	6.76	0.68	93506.21	27.30
437	497	1,5,8-Trimethoxy-12a-methyl-1,2,3,3a,3b,4,5,6,7,8,9,10,10b,11,12,12a-hexadecahydro-benzo[3,4]cyclohepta[1,2-E]indene	MAINLIB	6.23	0.68	93506.21	27.30

Hit Spectrum | Delta | Compound Structure

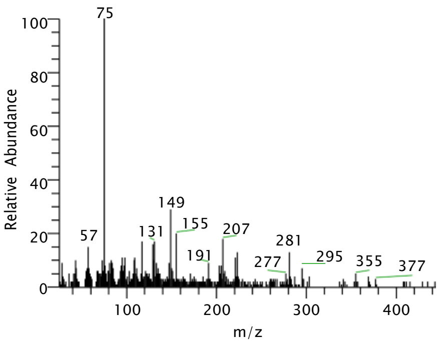

Raw data - Library entry

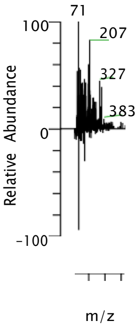

9,12,15-Octadecatrienoic acid, 2,3-bis[(trimethylsilyl)oxy]propyl ester, (Z,Z,Z)-
Formula C27H52O4Si2, MW 496, CAS# 55521-22-7, Entry# 9235
2,3-Bis[(trimethylsilyl)oxy]propyl (9E,12E,15E)-9,12,15-octadecatrienoate #

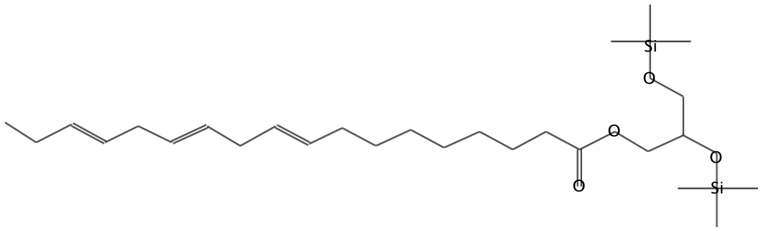

My Qual X-Report

Hit Spectrum | Delta | Compound Structure

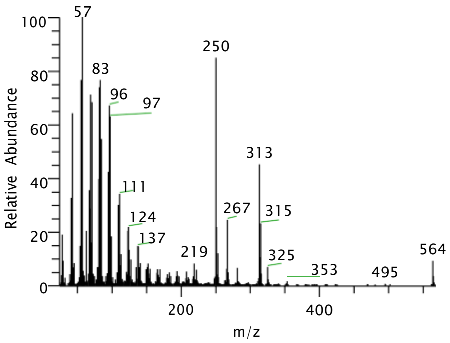

Raw data - Library entry

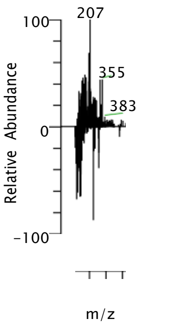

9-Octadecene, 1-[2-(octadecyloxy)ethoxy]-
Formula C38H76O2, MW 564, CAS# 56599-41-8, Entry# 22918
(9E)-1-[2-(Octadecyloxy)ethoxy]-9-octadecene #

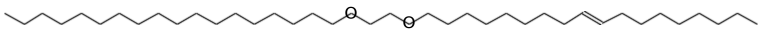

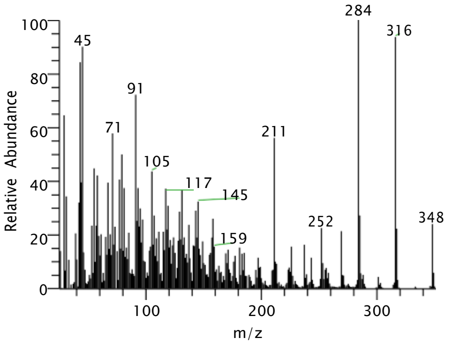

Raw data - Library entry

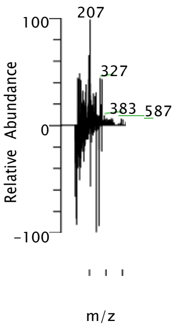

Formula C22H36O3, MW 348, CAS# NA, Entry# 148479

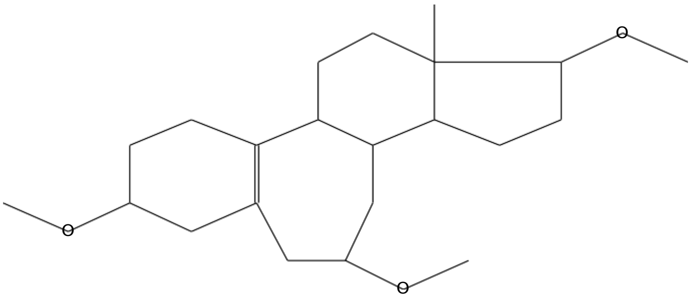

My Qual X-Report

Sample Name:

Run Time(min): 24.51

Injection Volume(µl):1 1.00

Low Mass(m/z): 50

High Mass(m/z): 650

Instrument Name:Thermo Scientific (GC-MS) DSQ

Instrument Model:Thermo Scientific GC Focus Series DSQ

Instrument Software Version:2.0.7 1

GCMS analysis of n-butanol fraction of *B. indica*

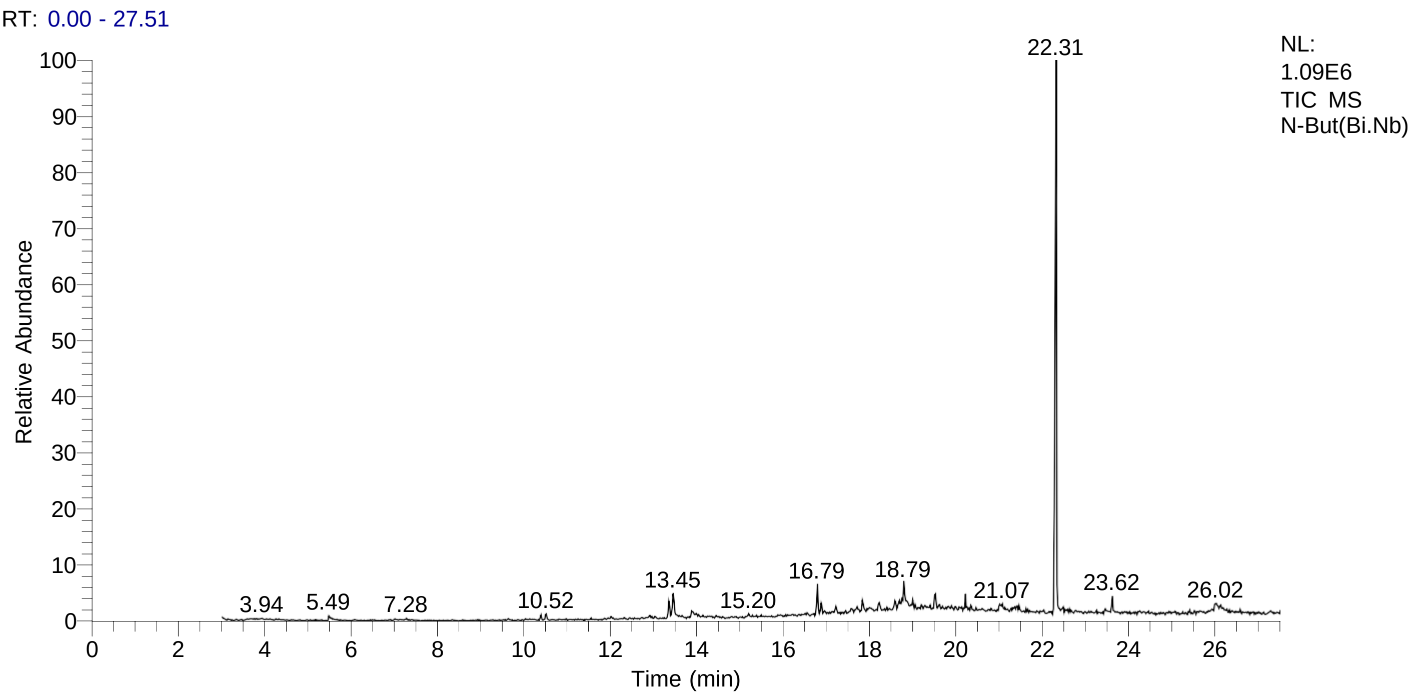

RT	Peak Area	Peak Height
3.92	59367.94	1956.68
4.28	9440.15	889.72
5.53	42129.02	3506.45
7.28	48468.64	1739.10
9.62	7910.64	850.67
10.50	69095.94	3885.08
12.01	26590.81	2251.07
12.31	10860.14	691.80
12.92	39868.15	2824.64
13.43	280842.30	19760.14
13.92	170746.16	7852.52
15.20	41660.04	2471.82
15.92	8084.64	1004.37
16.18	8806.89	813.99
16.51	7292.53	970.07
16.81	182892.89	15153.77
17.20	41543.94	4002.73
17.83	128725.65	6105.17
18.22	36702.42	4243.52
18.79	436560.90	22947.72
19.52	298557.26	11696.22
20.21	111976.95	7497.05
20.56	6154.09	857.41
21.05	95523.40	7671.50
21.40	100586.15	6700.83
21.99	7338.81	990.87
22.31	2602412.09	275657.83
23.62	112446.19	8258.10
23.92	12018.57	1067.72

My Qual X-Report

RT	Peak Area	Peak Height
24.29	41095.49	2183.26
25.00	29263.31	2090.72
26.04	319482.82	13402.93
27.28	6904.09	1099.20

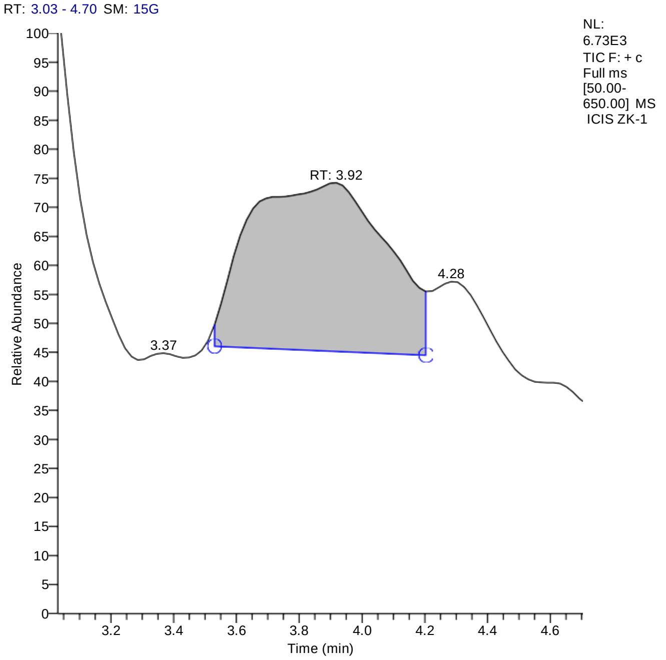

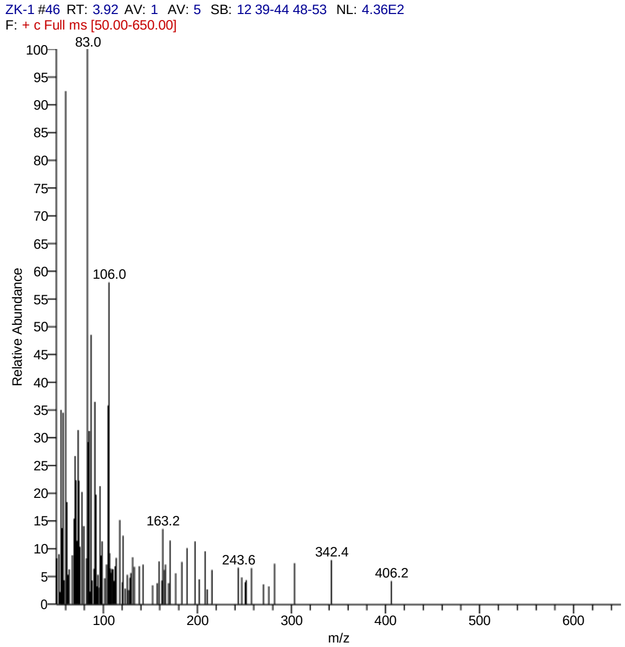

SI	RSI	Compound Name	Library	Probability	Area %	Area	RT
453	466	10,13-Octadecadiynoic acid, methyl ester	MAINLIB	9.44	1.10	59367.94	3.92
450	524	6-Methoxy-2-phenyl-hexahydropyrano[2,3-b][1,3]dioxine-7,8-diol	MAINLIB	8.34	1.10	59367.94	3.92
446	480	Benzene, (1-methylnonadecyl)-	replib	7.05	1.10	59367.94	3.92

Hit Spectrum | Delta | Compound Structure

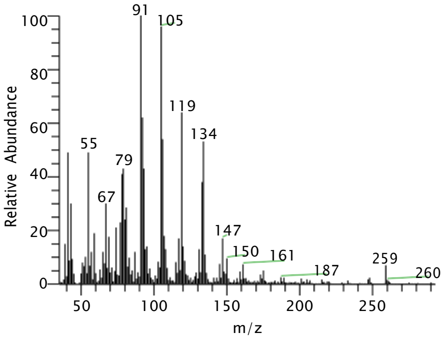

Raw data - Library entry

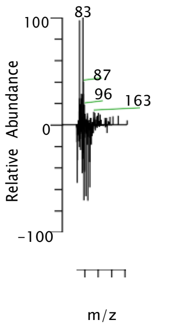

10,13-Octadecadiynoic acid, methyl ester
Formula C19H30O2, MW 290, CAS# 18202-24-9, Entry# 47817
Methyl 10,13-octadecadiynoate #

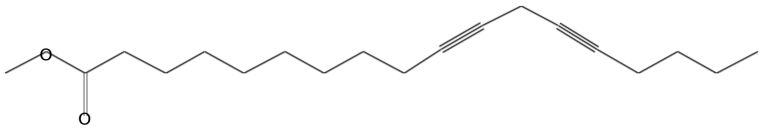

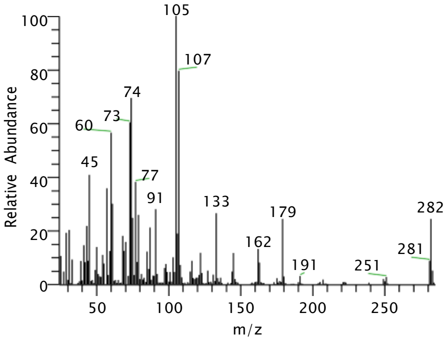

Raw data - Library entry

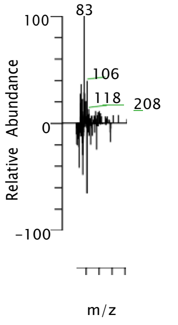

6-Methoxy-2-phenyl-hexahydropyrano[2,3-b][1,3]dioxine-7,8-diol
Formula C14H18O6, MW 282, CAS# NA, Entry# 61451

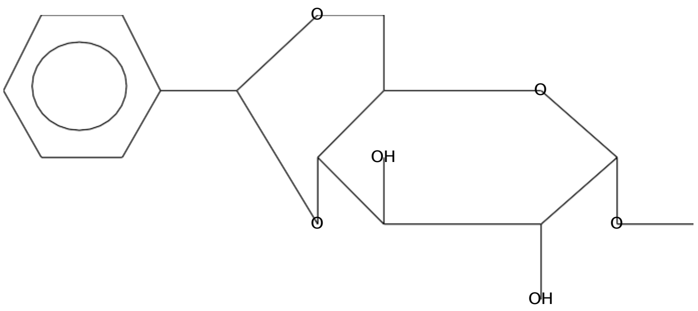

My Qual X-Report

Hit Spectrum	Delta	Compound Structure

Raw data - Library entry

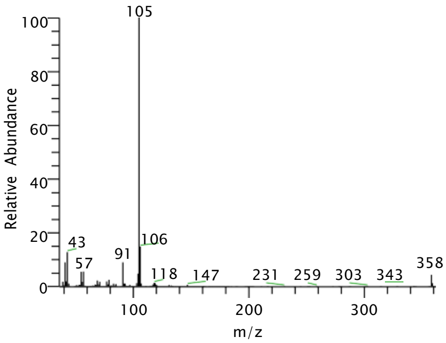

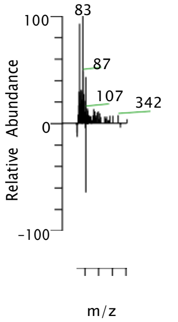

Benzene, (1-methylnonadecyl)-
Formula C26H46, MW 358, CAS# 2398-66-5, Entry# 13877
Eicosane, 2-phenyl-

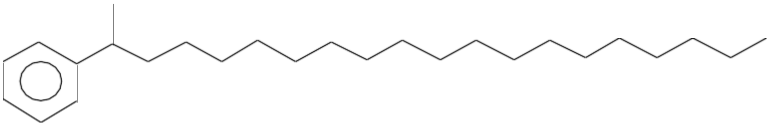

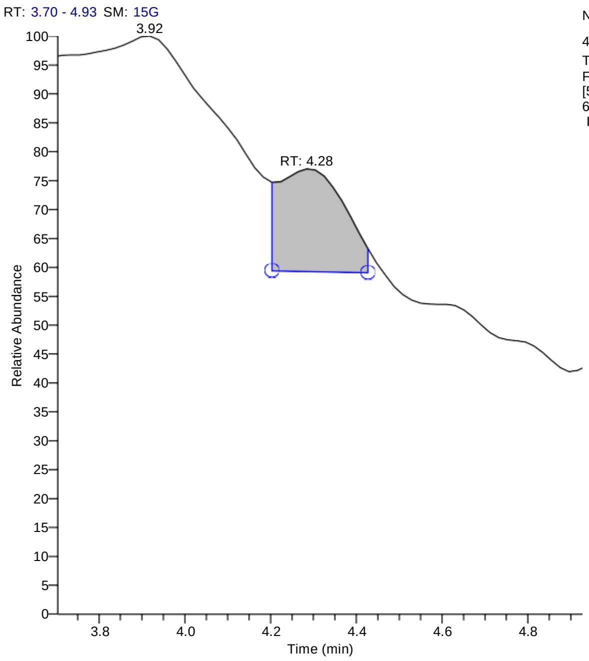

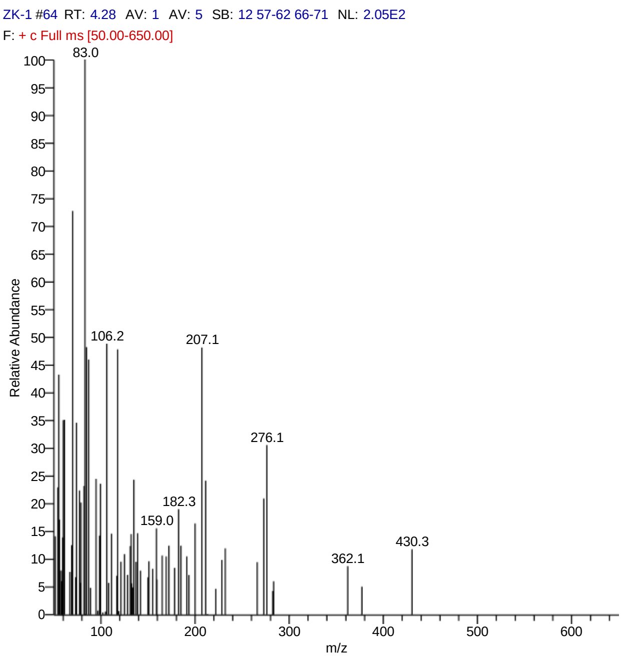

SI	RSI	Compound Name	Library	Probability	Area %	Area	RT
319	650	2,5-Dihydro-5-methoxy-2-furanone	MAINLIB	14.80	0.17	9440.15	4.28
311	374	2-(1-Chloro-2,3,3-trifluoro-cyclobutyl)-cyclopropanecarboxylic acid methyl ester	MAINLIB	11.04	0.17	9440.15	4.28
290	376	N,N'-Dicyclohexyl-1,2,4,5-tetrazine-3,6-diamine	MAINLIB	4.69	0.17	9440.15	4.28

Hit Spectrum	Delta	Compound Structure

Raw data - Library entry

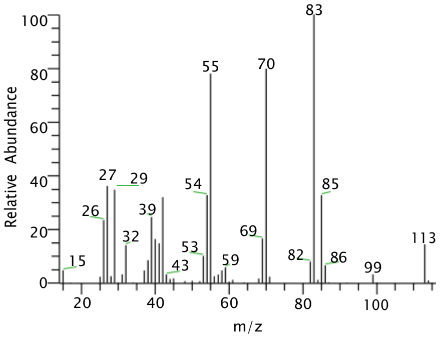

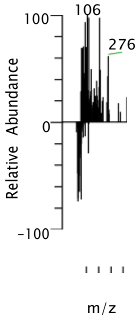

2,5-Dihydro-5-methoxy-2-furanone
Formula C5H6O3, MW 114, CAS# 10449-66-8, Entry# 41434
2(5H)-Furanone, 5-methoxy-

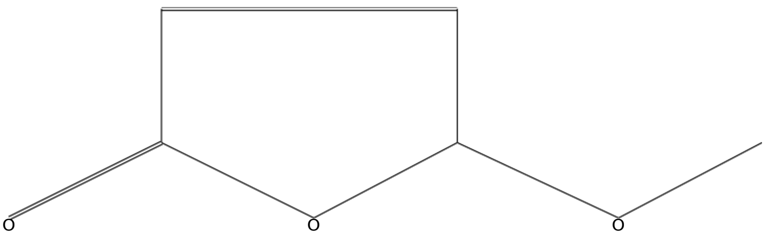

My Qual X-Report

Hit Spectrum	Delta	Compound Structure

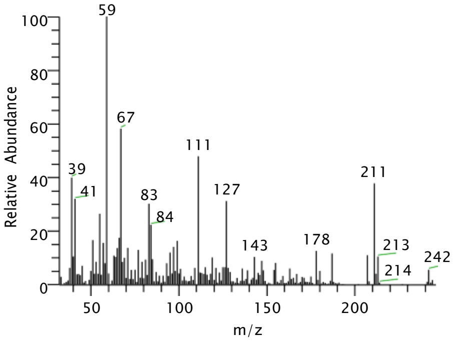

Raw data - Library entry

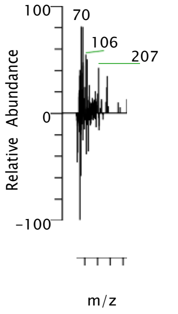

2-(1-Chloro-2,3,3-trifluoro-cyclobutyl)-cyclopropanecarboxylic acid methyl ester
Formula C9H10ClF3O2, MW 242, CAS# NA, Entry# 24893

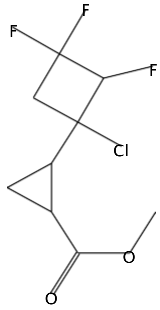

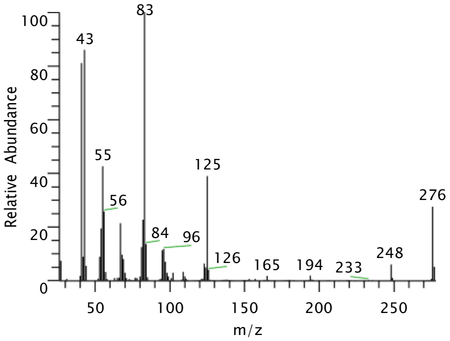

Raw data - Library entry

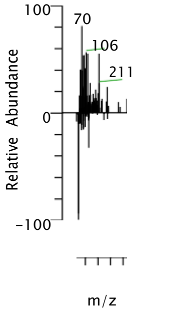

N,N'-Dicyclohexyl-1,2,4,5-tetrazine-3,6-diamine
Formula C14H24N6, MW 276, CAS# NA, Entry# 40970

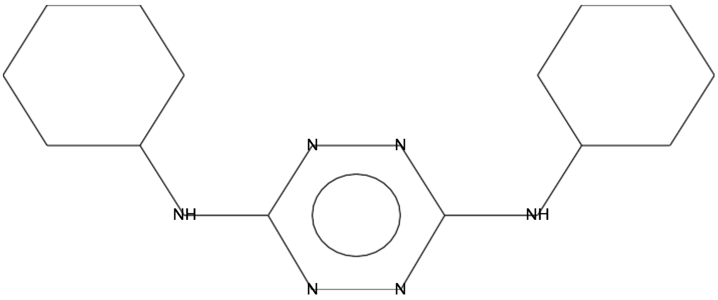

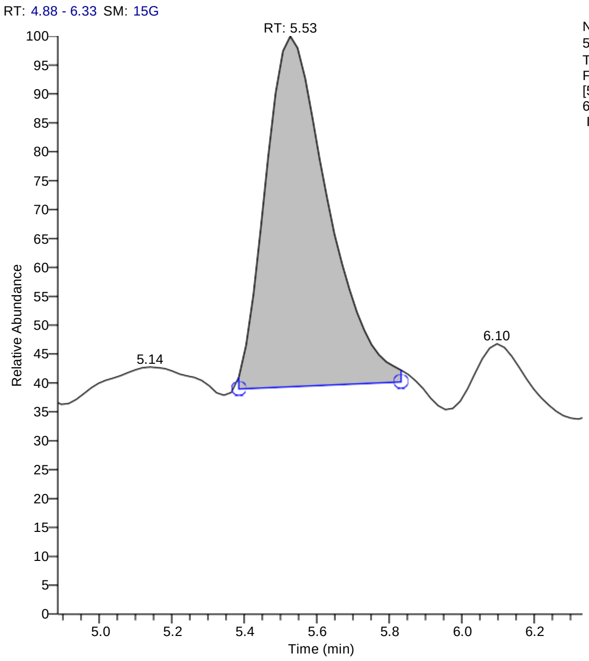

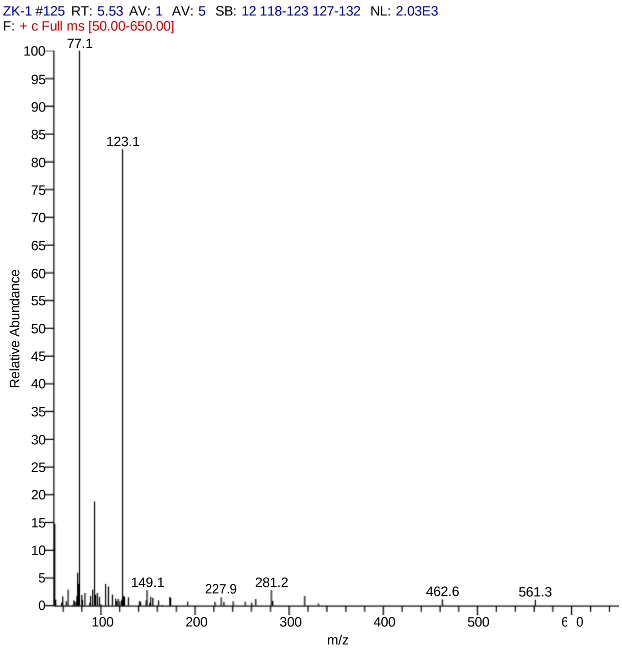

SI	RSI	Compound Name	Library	Probability	Area %	Area	RT
640	807	Benzene, nitro-	MAINLIB	79.38	0.78	42129.02	5.53
634	798	Benzene, nitro-	replib	79.38	0.78	42129.02	5.53
630	776	Benzene, nitro-	replib	79.38	0.78	42129.02	5.53

My Qual X-Report

Hit Spectrum | Delta | Compound Structure

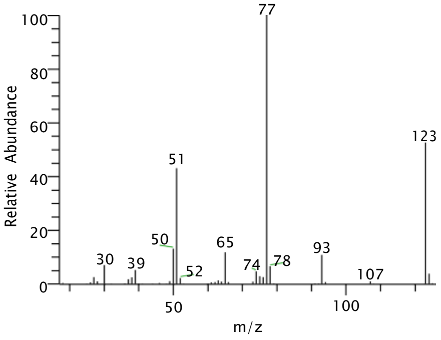

Raw data - Library entry

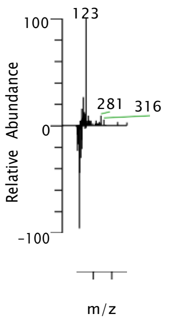

Benzene, nitro-
Formula C6H5NO2, MW 123, CAS# 98-95-3, Entry# 37273
Essence of Mirbane

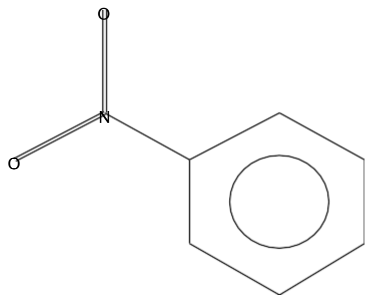

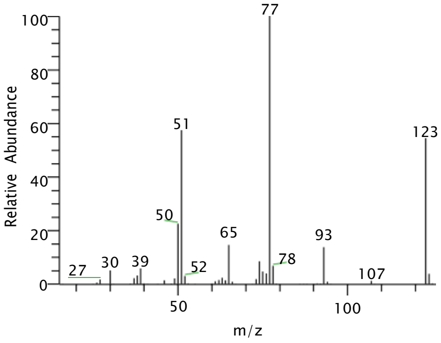

Raw data - Library entry

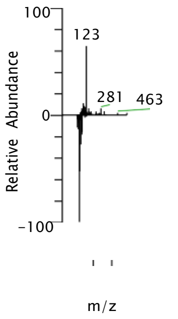

Benzene, nitro-
Formula C6H5NO2, MW 123, CAS# 98-95-3, Entry# 9318
Essence of Mirbane

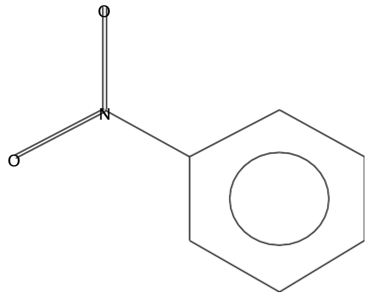

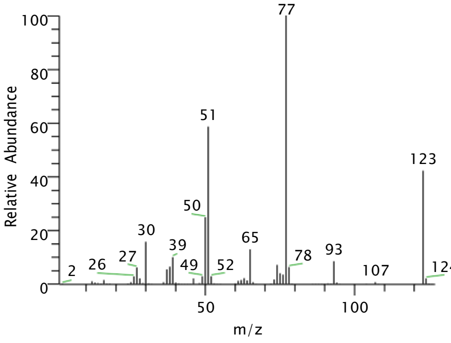

Raw data - Library entry

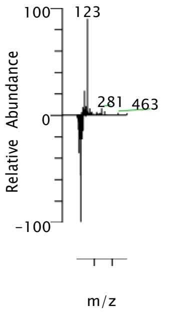

Benzene, nitro-
Formula C6H5NO2, MW 123, CAS# 98-95-3, Entry# 9316
Essence of Mirbane

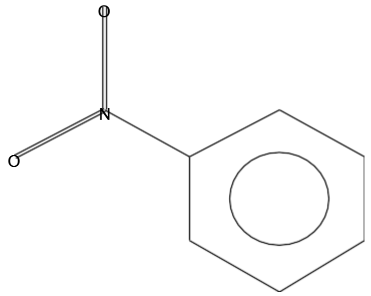

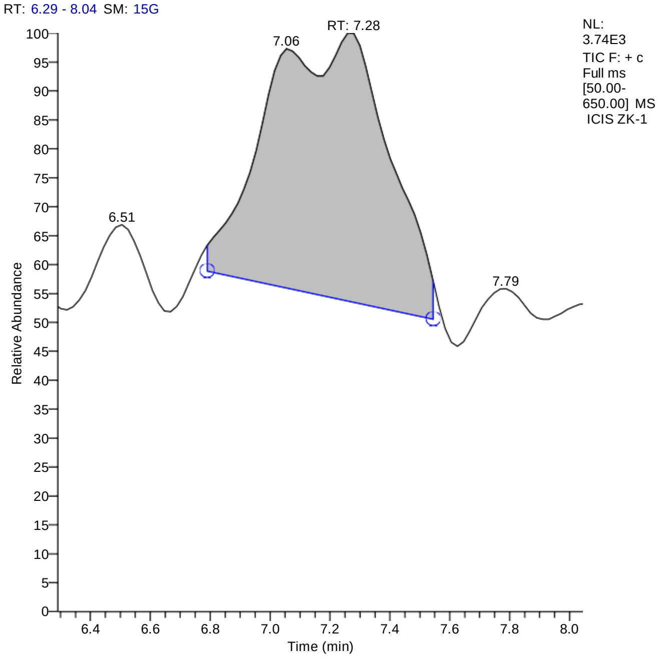

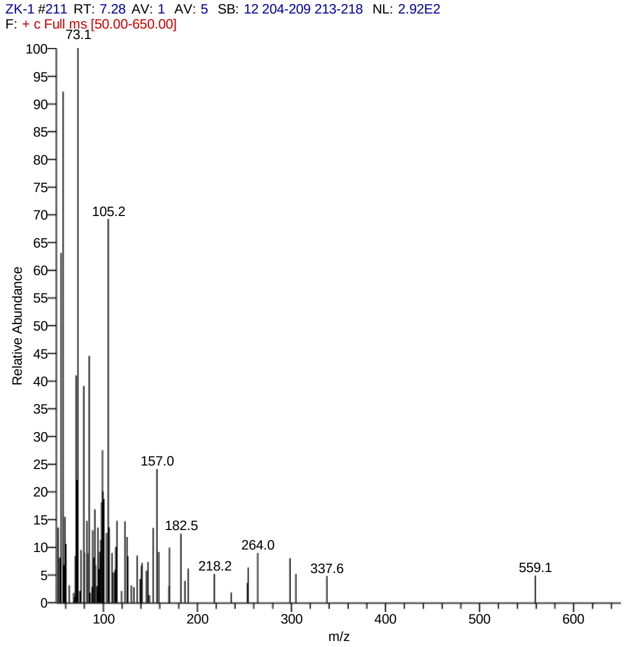

My Qual X-Report

SI	RSI	Compound Name	Library	Probability	Area %	Area	RT
464	471	9-Octadecenoic acid, (2-phenyl-1,3-dioxolan-4-yl)methyl ester, cis-	MAINLIB	7.08	0.90	48468.64	7.28
449	551	2-t-Butylperoxy-2-ethylbutan-1-ol, propionate ester	MAINLIB	4.29	0.90	48468.64	7.28
447	466	9-Octadecenoic acid, (2-phenyl-1,3-dioxolan-4-yl)methyl ester, trans-	MAINLIB	3.96	0.90	48468.64	7.28

Hit Spectrum | Delta | Compound Structure

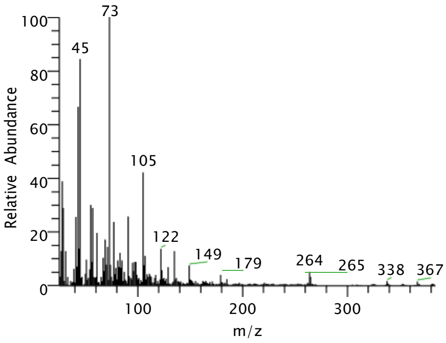

Raw data - Library entry

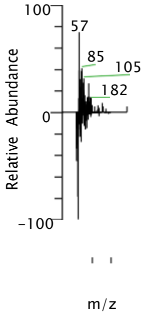

9-Octadecenoic acid, (2-phenyl-1,3-dioxolan-4-yl)methyl ester, cis-
Formula C28H44O4, MW 444, CAS# 56599-45-2, Entry# 32353
(2-Phenyl-1,3-dioxolan-4-yl)methyl (9E)-9-octadecenoate #

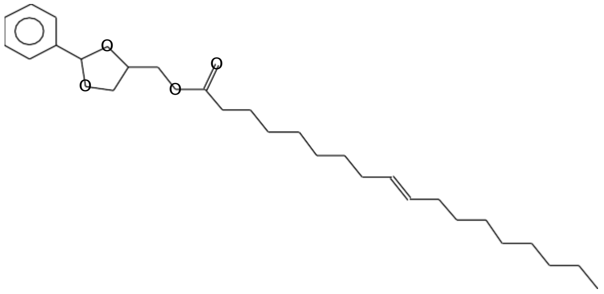

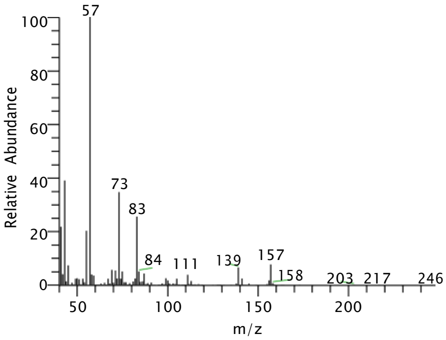

Raw data - Library entry

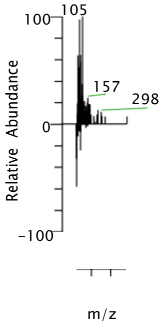

2-t-Butylperoxy-2-ethylbutan-1-ol, propionate ester
Formula C13H26O4, MW 246, CAS# 139727-32-5, Entry# 20971
2-(tert-Butylperoxy)-2-ethylbutyl propionate #

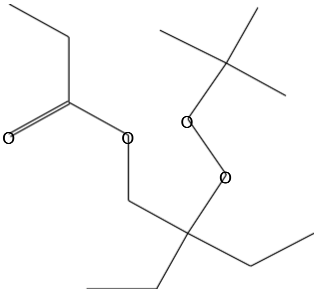

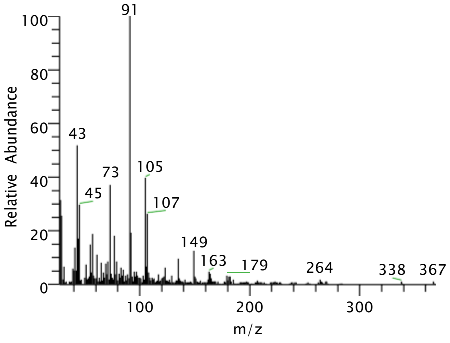

Raw data - Library entry

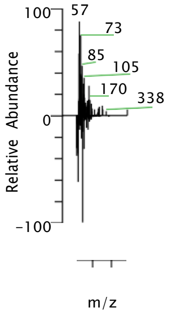

9-Octadecenoic acid, (2-phenyl-1,3-dioxolan-4-yl)methyl ester, trans-
Formula C28H44O4, MW 444, CAS# 56599-46-3, Entry# 46255
(2-Phenyl-1,3-dioxolan-4-yl)methyl (9E)-9-octadecenoate #

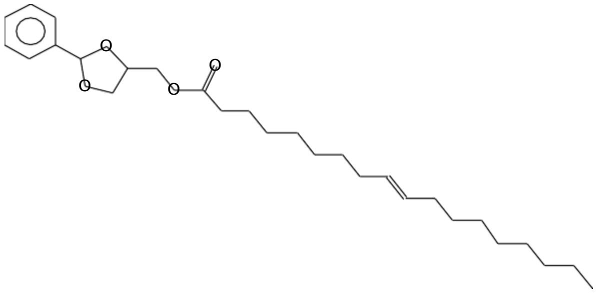

My Qual X-Report

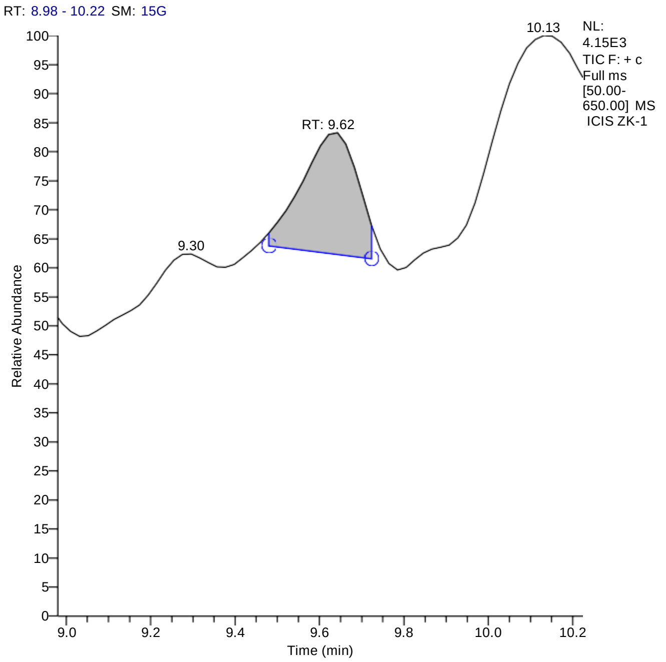

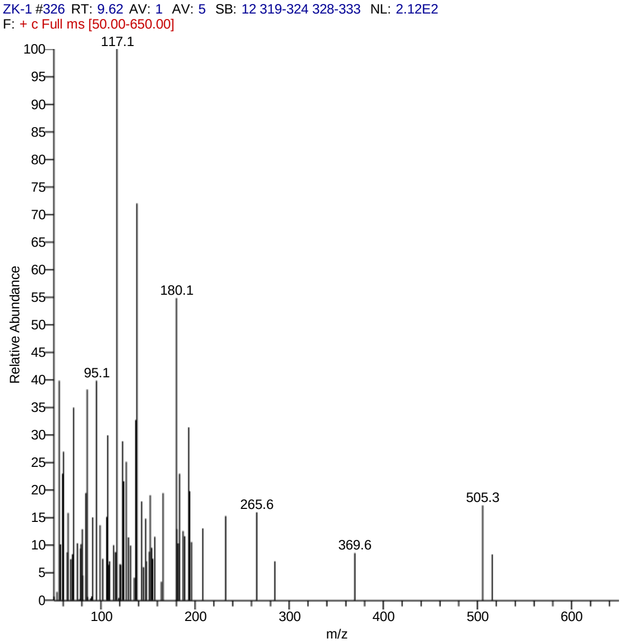

SI	RSI	Compound Name	Library	Probability	Area %	Area	RT
392	429	Acetamide, N-methyl-N-[4-[2-acetoxymethyl-1-pyrrolidyl]-2-butynyl]-	MAINLIB	8.21	0.15	7910.64	9.62
385	470	diallyl(2,2,4a,7,7-pentamethyl-1,2,3,4,4a,5,6,7-octahydro[1,8]naphthyridin-1-yl) phosphate	MAINLIB	6.29	0.15	7910.64	9.62
375	449	6,8a-Epidioxy-4a-methyl-2-oxo-3,4,4a,5,6,7,8,8a-octahydro-2H-1-benzofur	MAINLIB	4.44	0.15	7910.64	9.62

Hit Spectrum | Delta | Compound Structure

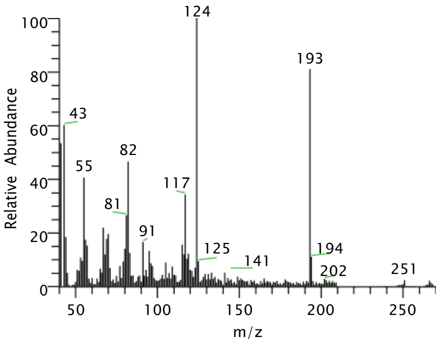

Raw data - Library entry

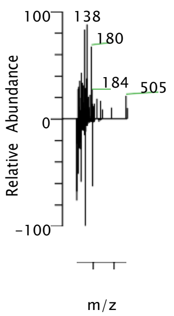

Acetamide, N-methyl-N-[4-[2-acetoxymethyl-1-pyrrolidyl]-2-butynyl]-
Formula C14H22N2O3, MW 266, CAS# 132377-28-7, Entry# 77757
(1-(4-[Acetyl(methyl)amino]-2-butynyl)-2-pyrrolidinyl)methyl acetate #

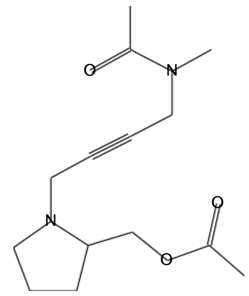

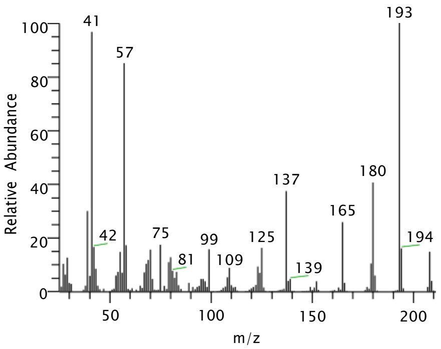

Raw data - Library entry

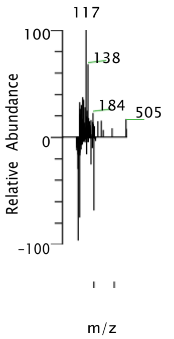

diallyl(2,2,4a,7,7-pentamethyl-1,2,3,4,4a,5,6,7-octahydro[1,8]naphthyridin-1-yl)phosphate
Formula C19H33N2O4P, MW 384, CAS# NA, Entry# 120640

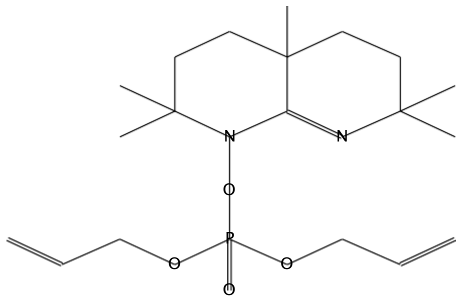

My Qual X-Report

Hit Spectrum | Delta | Compound Structure

Raw data - Library entry

6,8a-Epidioxy-4a-methyl-2-oxo-3,4,4a,5,6,7,8,8a-octahydro-2H-1-benzofur
Formula C10H14O4, MW 198, CAS# NA, Entry# 88421

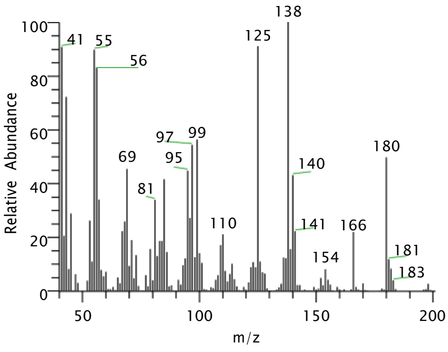

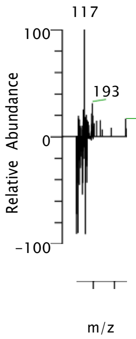

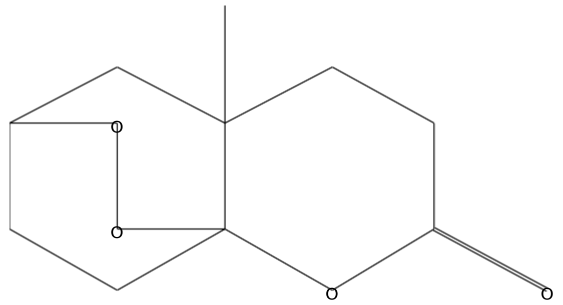

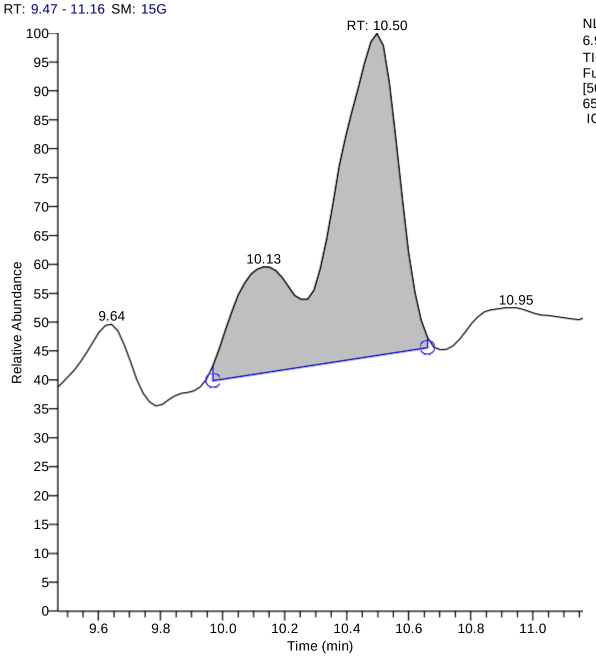

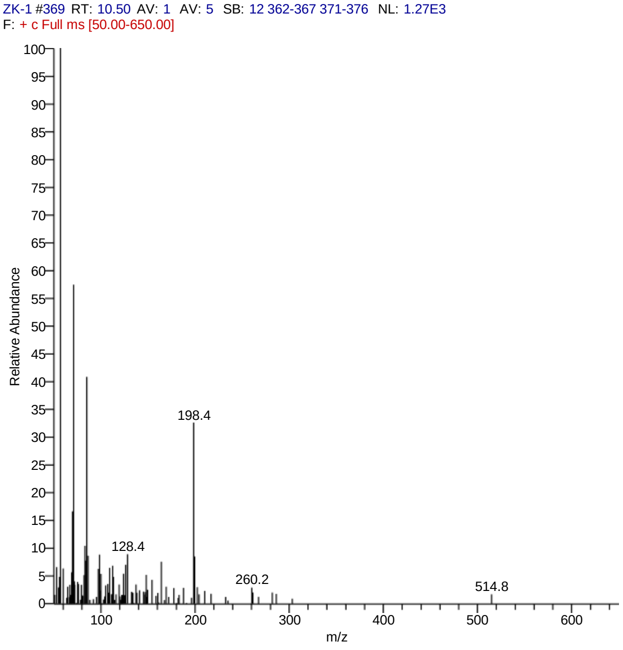

SI	RSI	Compound Name	Library	Probability	Area %	Area	RT
587	747	Tetradecane	replib	36.77	1.28	69095.94	10.50
578	739	Tetradecane	MAINLIB	36.77	1.28	69095.94	10.50
575	733	Tetradecane	replib	36.77	1.28	69095.94	10.50

Hit Spectrum | Delta | Compound Structure

Raw data - Library entry

Tetradecane
Formula C14H30, MW 198, CAS# 629-59-4, Entry# 5465
n-Tetradecane

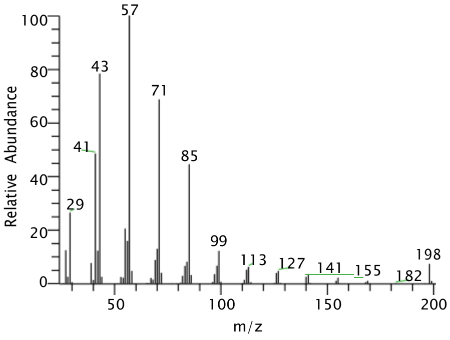

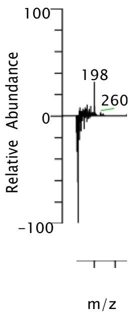

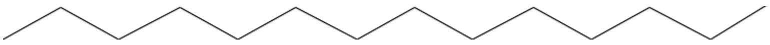

My Qual X-Report

Hit Spectrum	Delta	Compound Structure

Raw data – Library entry

Tetradecane
Formula C14H30, MW 198, CAS# 629-59-4, Entry# 20857
n-Tetradecane

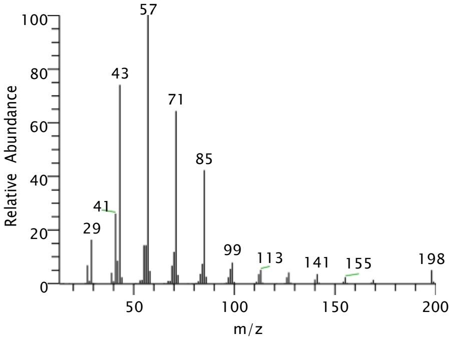

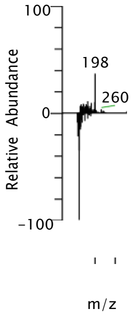

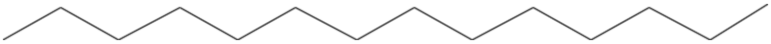

Raw data – Library entry

Tetradecane
Formula C14H30, MW 198, CAS# 629-59-4, Entry# 5463
n-Tetradecane

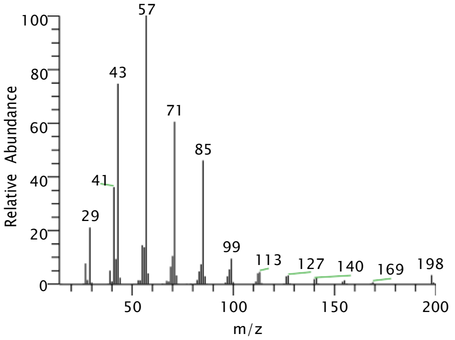

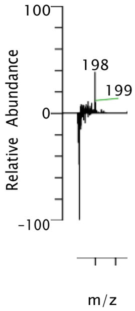

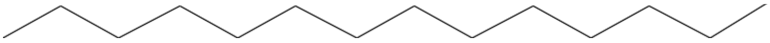

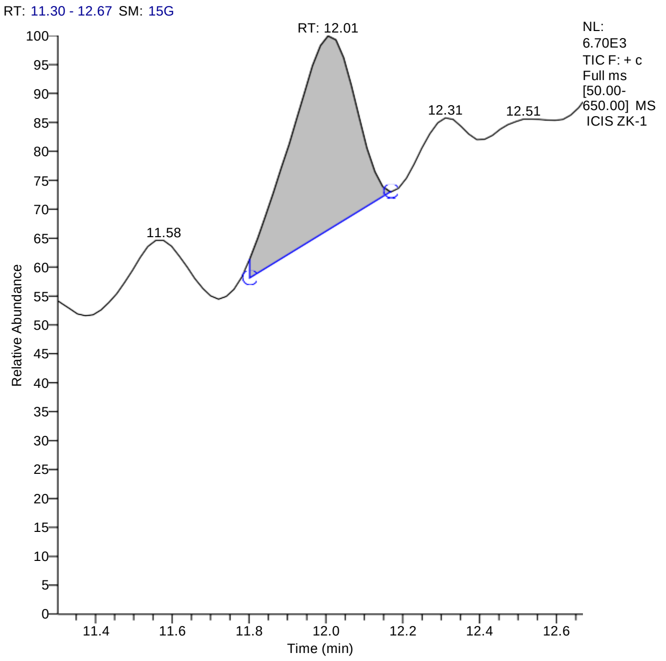

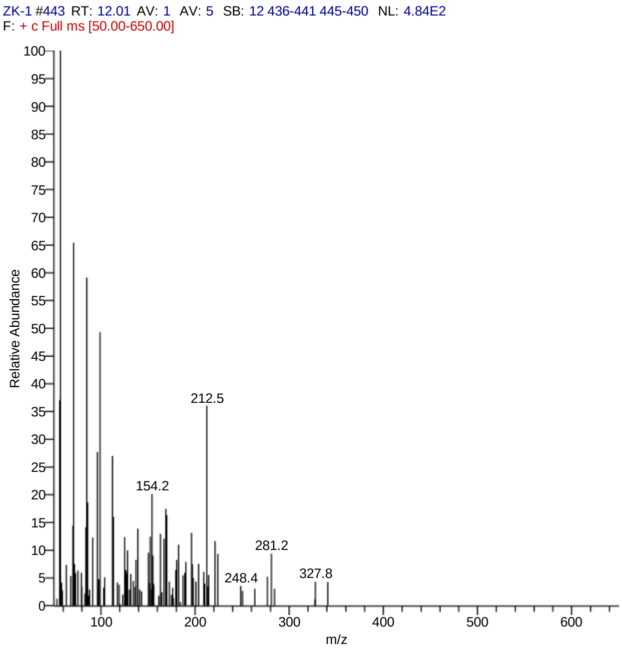

SI	RSI	Compound Name	Library	Probability	Area %	Area	RT
509	532	Dodecane, 5,8-diethyl-	MAINLIB	28.35	0.49	26590.81	12.01
445	491	Hexadecane, 5-butyl-	MAINLIB	4.65	0.49	26590.81	12.01
439	487	Tetracosane	replib	3.66	0.49	26590.81	12.01

My Qual X-Report

Hit Spectrum | Delta | Compound Structure

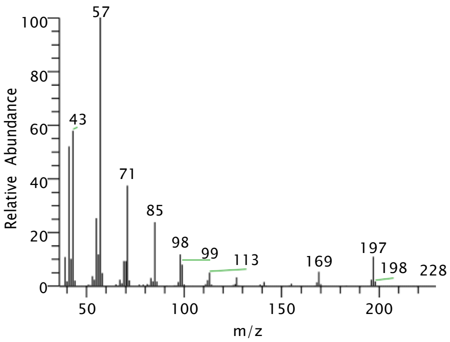

Raw data - Library entry

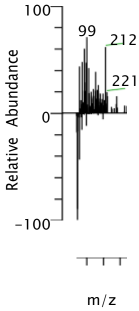

Dodecane, 5,8-diethyl-
Formula C16H34, MW 226, CAS# 24251-86-3, Entry# 20683
5,8-Diethyldodecane

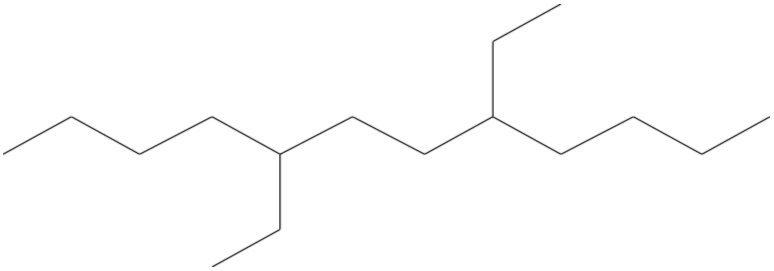

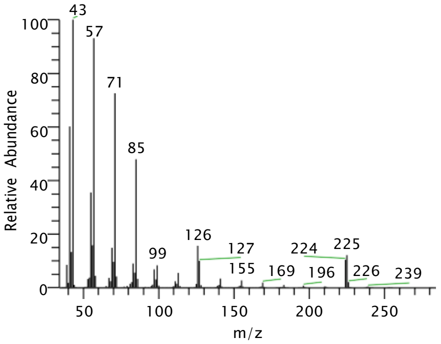

Raw data - Library entry

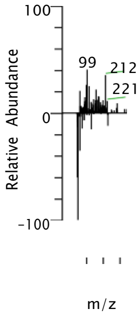

Hexadecane, 5-butyl-
Formula C20H42, MW 282, CAS# 6912-07-8, Entry# 7065
5-n-Butylhexadecane

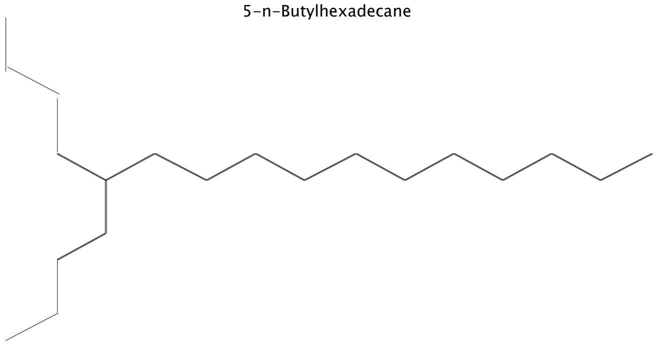

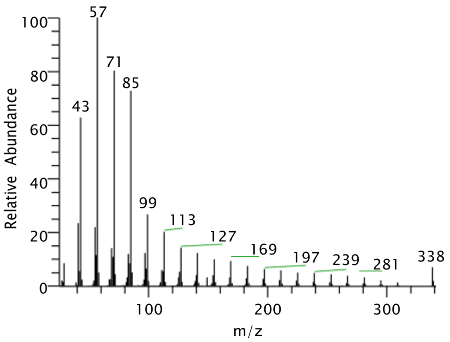

Raw data - Library entry

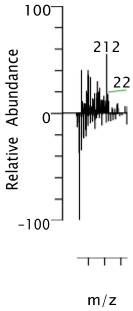

Tetracosane
Formula C24H50, MW 338, CAS# 646-31-1, Entry# 5747
n-Tetracosane

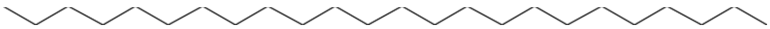

RT: 11.69 - 13.12 SM: 15G

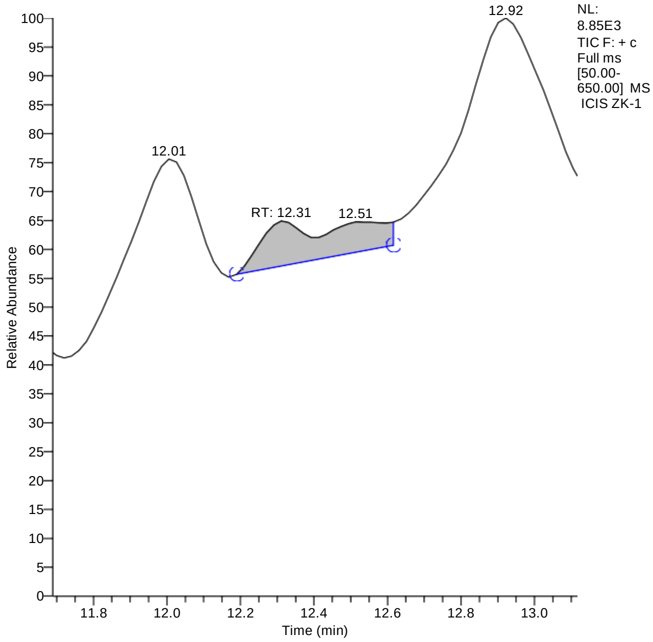

ZK-1 #458 RT: 12.31 AV: 1 AV: 5 SB: 12 451-456 460-465 NL: 4.84E2
F: + c Full ms [50.00-650.00]

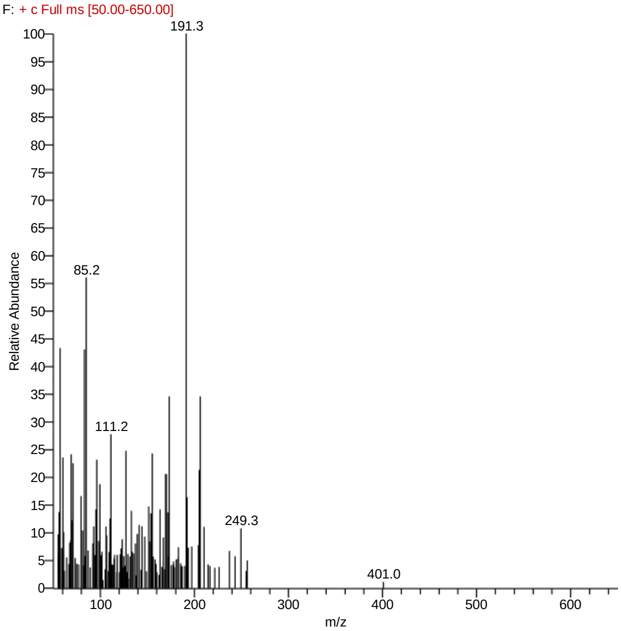

My Qual X-Report

SI	RSI	Compound Name	Library	Probability	Area %	Area	RT
498	536	1-Penten-3-one, 1-(2,6,6-trimethyl-1-cyclohexen-1-yl)-	MAINLIB	7.67	0.20	10860.14	12.31
490	523	2H-Indeno[1,2-b]furan-2-one, 3,3a,4,5,6,7,8,8b-octahydro-8,8-dimethyl	MAINLIB	5.72	0.20	10860.14	12.31
489	506	Benzo[e]isobenzofuran-1,4-dione,1,3,4,5,5a,6,7,8,9,9a-decahydro-6,6,9a-trimethyl	MAINLIB	5.50	0.20	10860.14	12.31

Hit Spectrum	Delta	Compound Structure

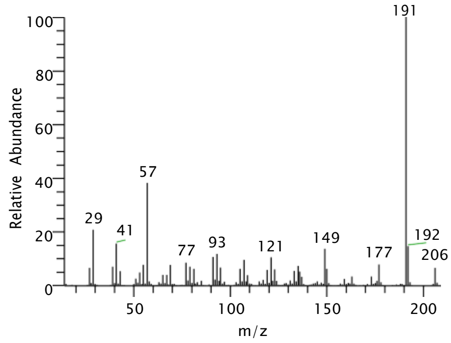

Raw data - Library entry

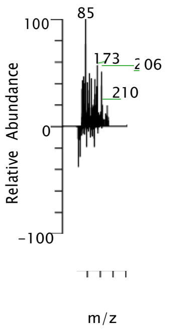

1- Penten-3-one, 1-(2,6,6-trimethyl-1-cyclohexen-1-yl)-
Formula C14H22O, MW 206, CAS# 127-43-5, Entry# 119694
á-Ionone, methyl-

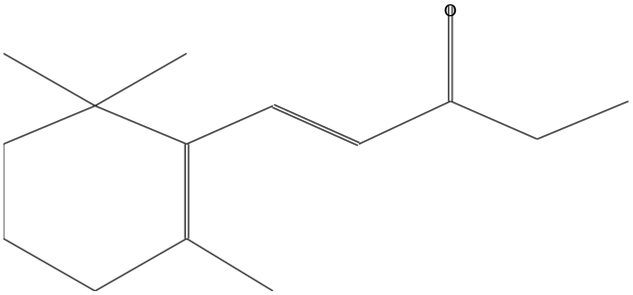

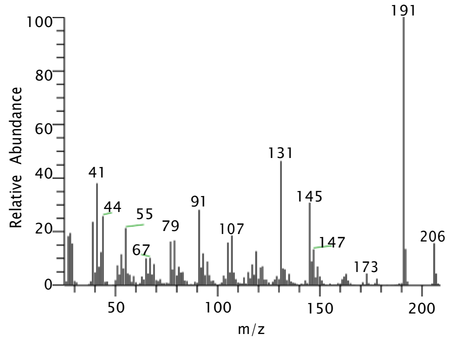

Raw data - Library entry

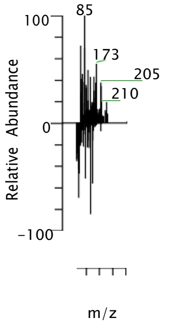

2H-Indeno[1,2-b]furan-2-one, 3,3a,4,5,6,7,8,8b-octahydro-8,8-dimethyl
Formula C13H18O2, MW 206, CAS# NA, Entry# 119785

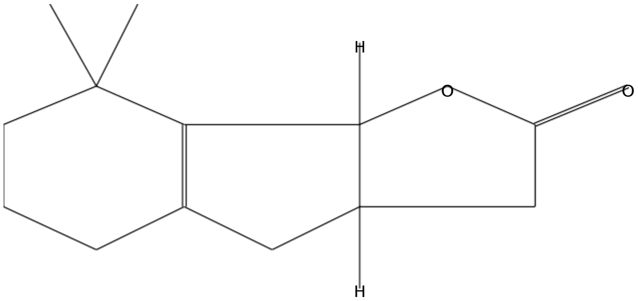

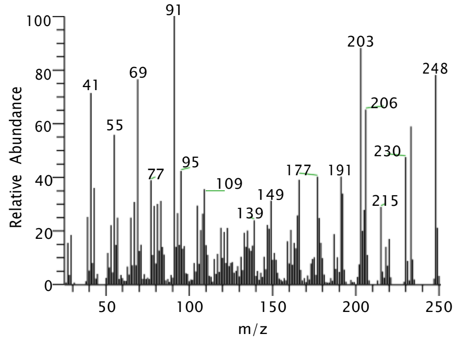

Raw data - Library entry

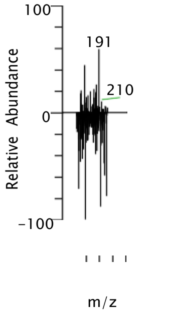

Benzo[e]isobenzofuran-1,4-dione,1,3,4,5,5a,6,7,8,9,9a-decahydro-6,6,9a-trimethyl
Formula C15H20O3, MW 248, CAS# NA, Entry# 49760

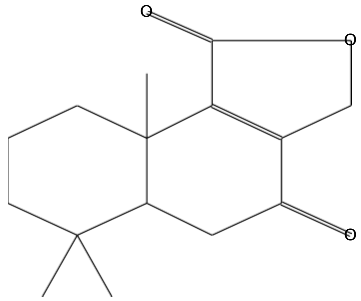

My Qual X-Report

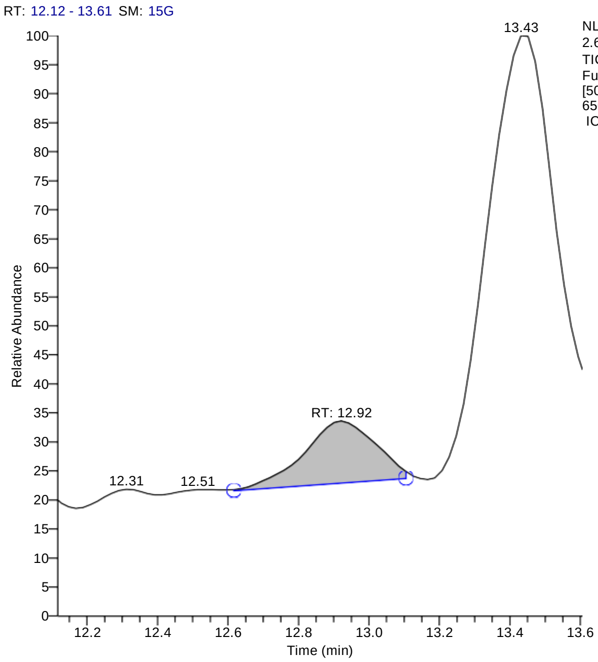

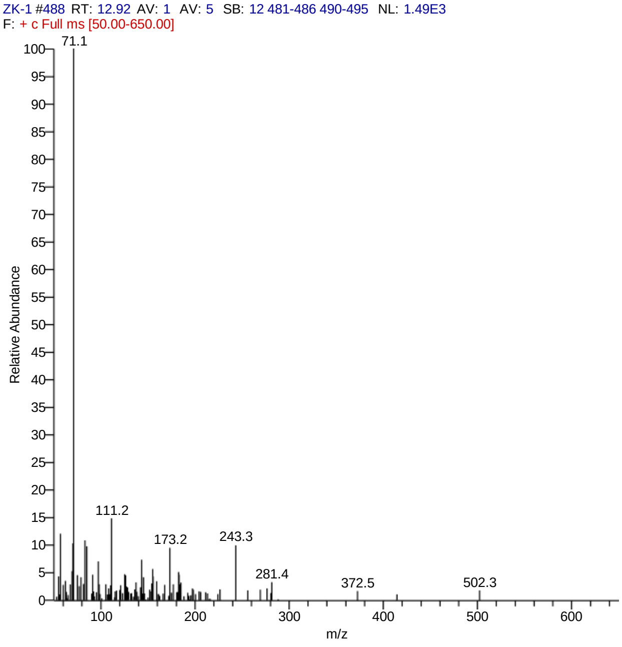

SI	RSI	Compound Name	Library	Probability	Area %	Area	RT
502	688	Pentanoic acid, 2,2,4-trimethyl-3-carboxyisopropyl, isobutyl ester	MAINLIB	12.01	0.74	39868.15	12.92
490	575	Eicosane, 7-hexyl-	MAINLIB	8.00	0.74	39868.15	12.92
487	512	Octadecane, 3-ethyl-5-(2-ethylbutyl)-	MAINLIB	7.07	0.74	39868.15	12.92

Hit Spectrum	Delta	Compound Structure

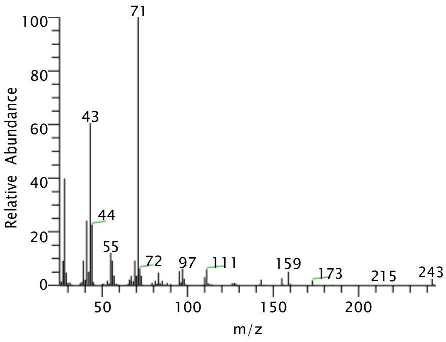

Raw data - Library entry

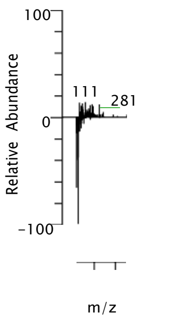

Pentanoic acid, 2,2,4-trimethyl-3-carboxyisopropyl, isobutyl ester
Formula C16H30O4, MW 286, CAS# NA, Entry# 30755

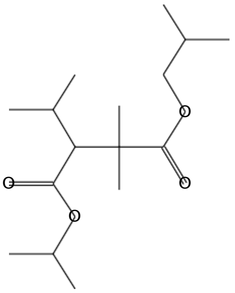

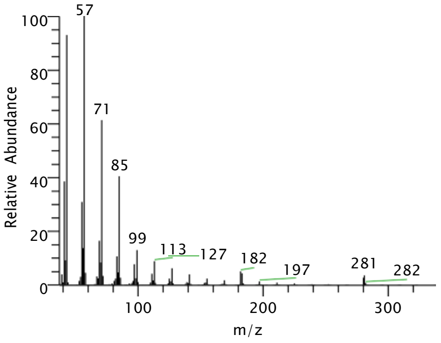

Raw data - Library entry

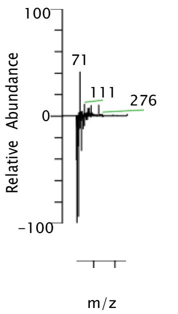

Eicosane, 7-hexyl-
Formula C26H54, MW 366, CAS# 55333-99-8, Entry# 20869
7-n-Hexyleicosane

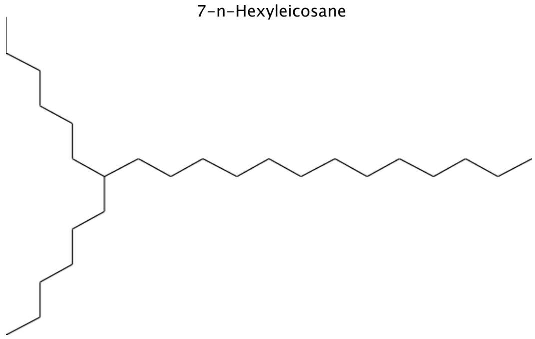

My Qual X-Report

Hit Spectrum | Delta | Compound Structure

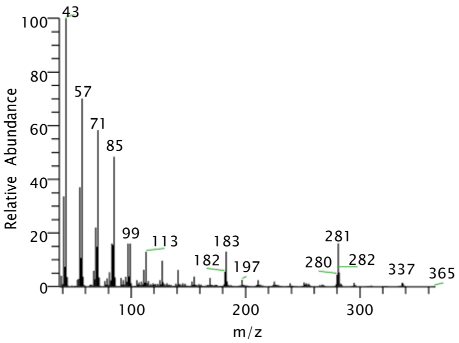

Raw data - Library entry

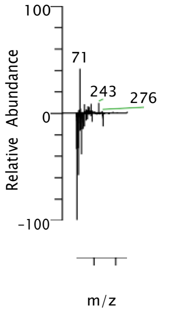

Octadecane, 3-ethyl-5-(2-ethylbutyl)-
Formula C26H54, MW 366, CAS# 55282-12-7, Entry# 7093
3-Ethyl-5-(2'-ethylbutyl)octadecane

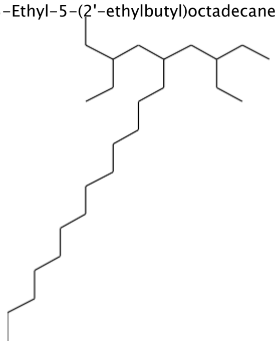

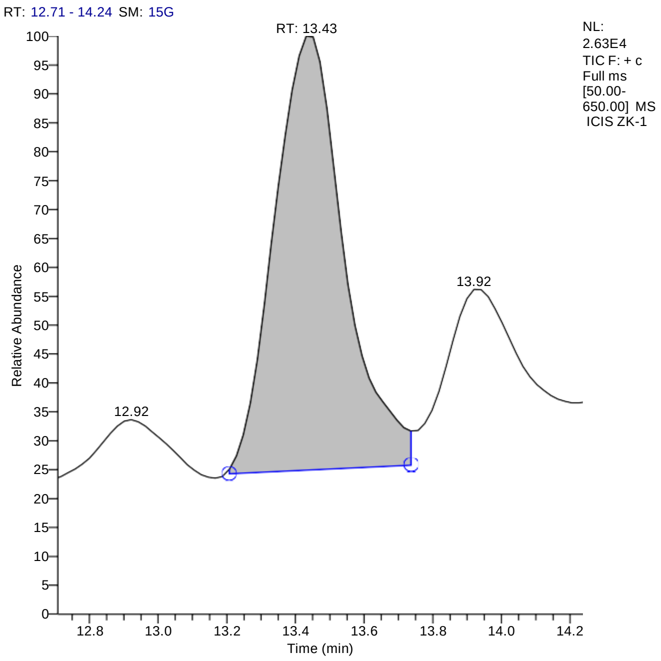

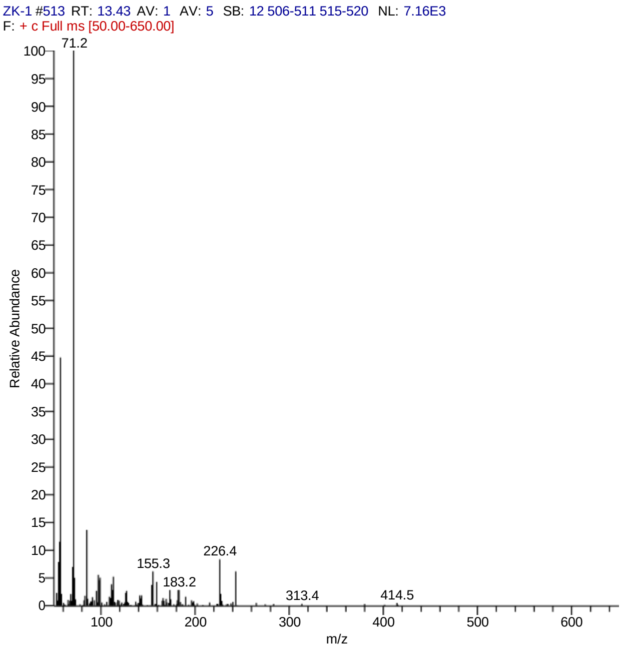

SI	RSI	Compound Name	Library	Probability	Area %	Area	RT
655	767	Hexadecane	replib	12.86	5.20	280842.30	13.43
642	762	Hexadecane	replib	12.86	5.20	280842.30	13.43
642	744	Hexadecane	MAINLIB	12.86	5.20	280842.30	13.43

Hit Spectrum | Delta | Compound Structure

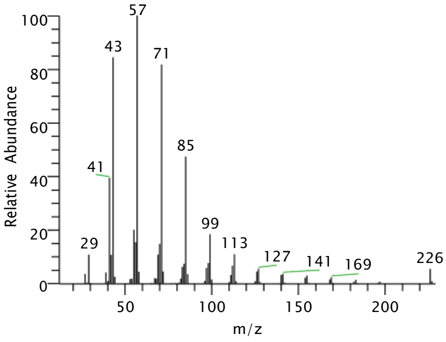

Raw data - Library entry

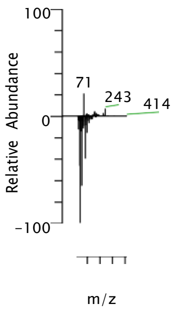

Hexadecane
Formula C16H34, MW 226, CAS# 544-76-3, Entry# 5517
n-Cetane

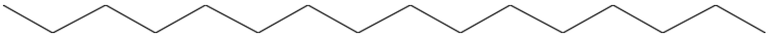

My Qual X-Report

Hit Spectrum | Delta | Compound Structure

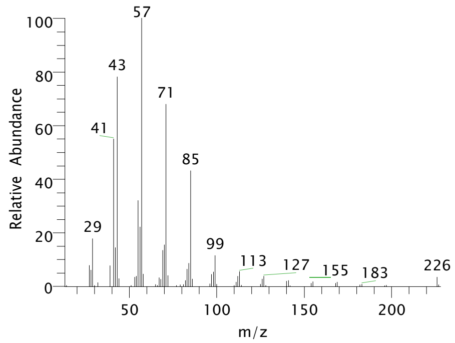

Raw data - Library entry

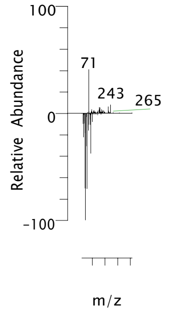

Hexadecane
Formula C16H34, MW 226, CAS# 544-76-3, Entry# 5518
n-Cetane

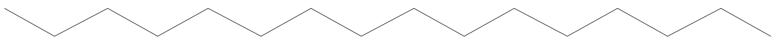

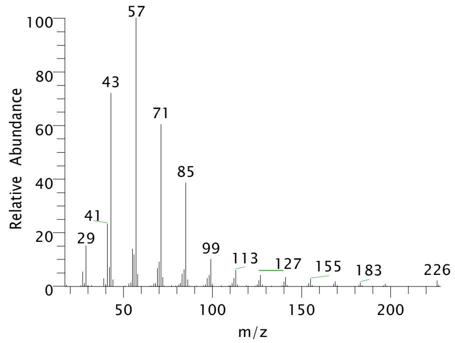

Raw data - Library entry

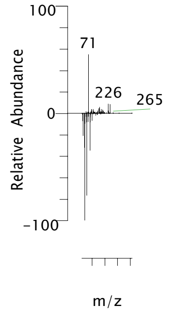

Hexadecane
Formula C16H34, MW 226, CAS# 544-76-3, Entry# 20955
n-Cetane

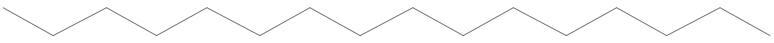

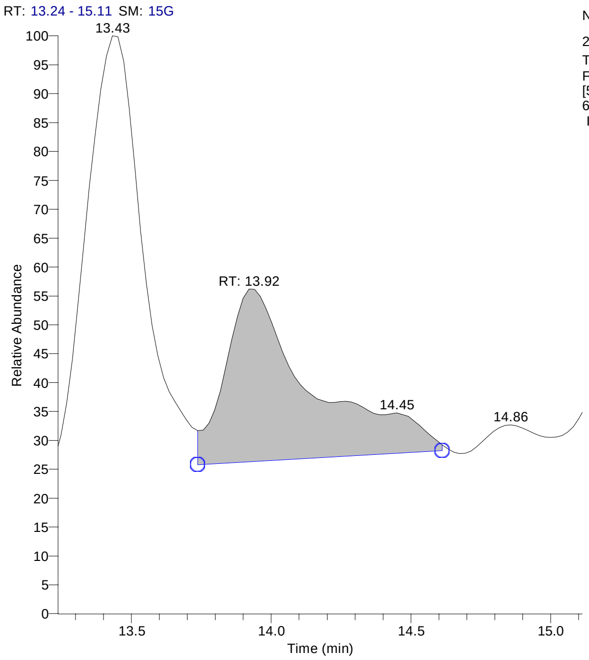

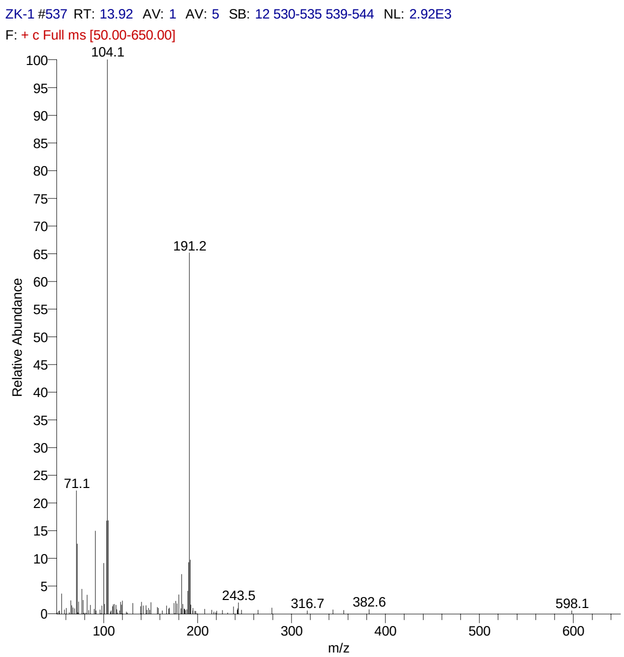

SI	RSI	Compound Name	Library	Probability	Area %	Area	RT
555	643	Acetamide, N-(4-phenylbutyl)-	MAINLIB	33.26	3.16	170746.16	13.92
550	618	2-Pyrrolidinone, 3-hydroxy-3-methyl-4-phenyl-, (3R-cis)-	MAINLIB	26.81	3.16	170746.16	13.92
531	726	4-Isothiazolol, 5-methyl-3-phenyl-	MAINLIB	13.01	3.16	170746.16	13.92

My Qual X-Report

Hit Spectrum	Delta	Compound Structure

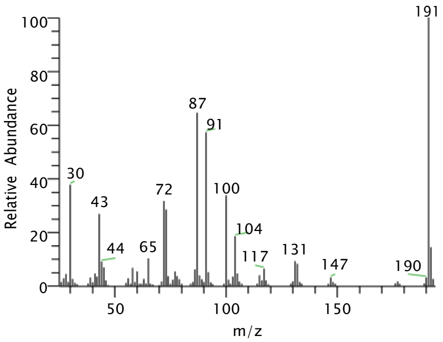

Raw data - Library entry

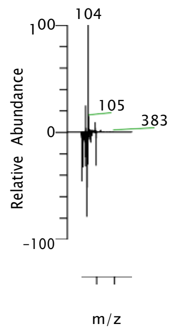

Acetamide, N-(4-phenylbutyl)-
Formula C12H17NO, MW 191, CAS# 34059-11-5, Entry# 119728
N-Acetyl-4-phenylbutylamine

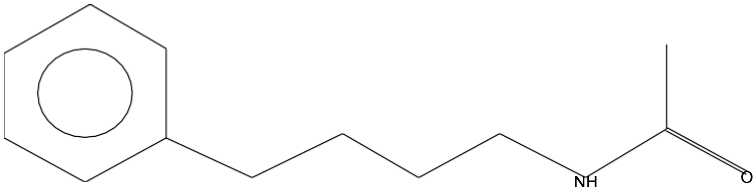

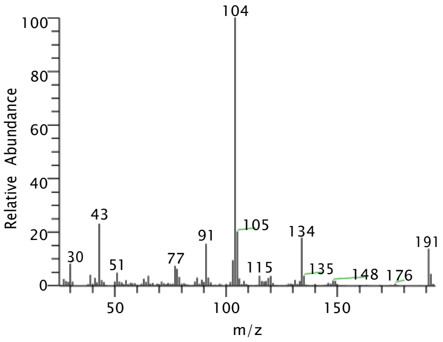

Raw data - Library entry

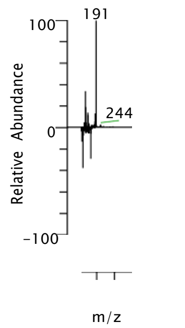

2-Pyrrolidinone, 3-hydroxy-3-methyl-4-phenyl-, (3R-cis)-
Formula C11H13NO2, MW 191, CAS# 104194-16-3, Entry# 58473
3-Hydroxy-3-methyl-4-phenyl-2-pyrrolidinone #

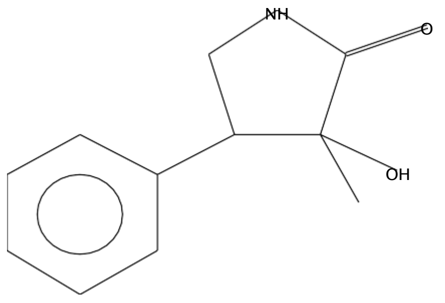

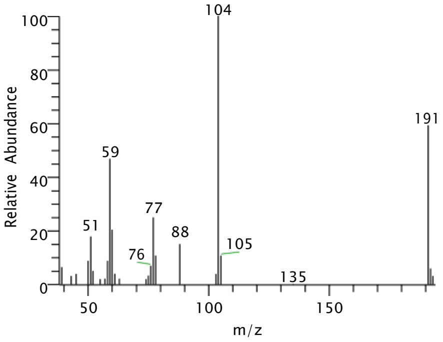

Raw data - Library entry

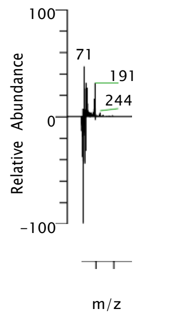

4-
5-

Isothiazolol, 5-methyl-3-phenyl-
Formula C10H9NOS, MW 191, CAS# 19389-29-8, Entry# 59135
Methyl-3-phenyl-4-isothiazolol #

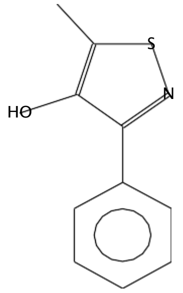

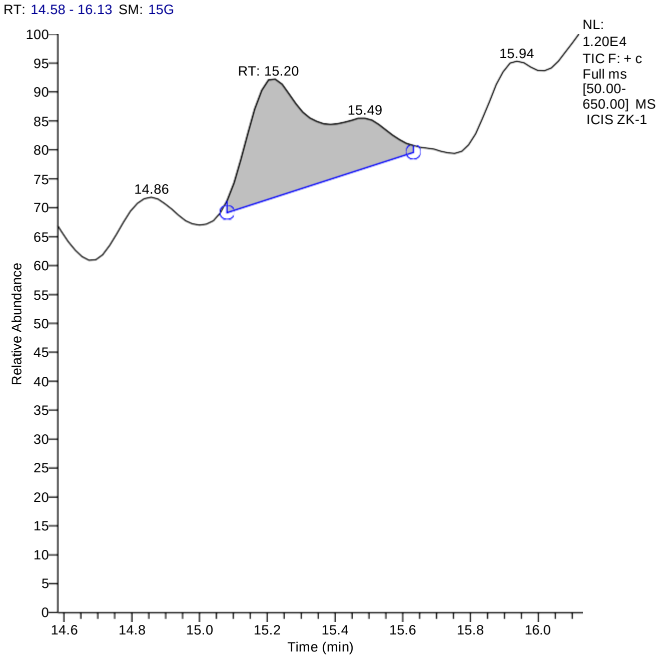

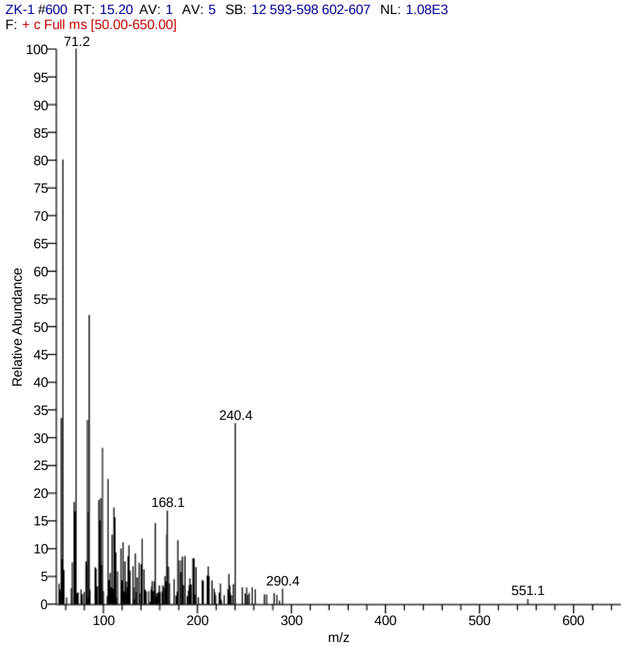

SI	RSI	Compound Name	Library	Probability	Area %	Area	RT
590	614	Ethanol, 2-(tetradecyloxy)-	MAINLIB	23.65	0.77	41660.04	15.20

My Qual X-Report

SI	RSI	Compound Name	Library	Probability	Area %	Area	RT
549	709	Ethanol, 2-(hexadecyloxy)-	replib	5.39	0.77	41660.04	15.20
548	558	Octadecane, 1-[2-(hexadecyloxy)ethoxy]-	replib	5.18	0.77	41660.04	15.20

Hit Spectrum | Delta | Compound Structure

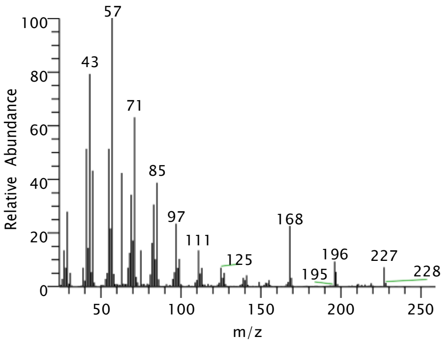

Raw data - Library entry

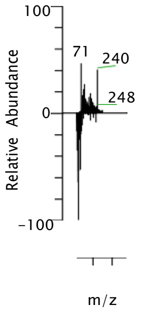

Ethanol, 2-(tetradecyloxy)-
Formula C16H34O2, MW 258, CAS# 2136-70-1, Entry# 20879
Myristyl monoethoxylate

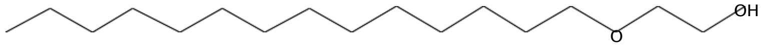

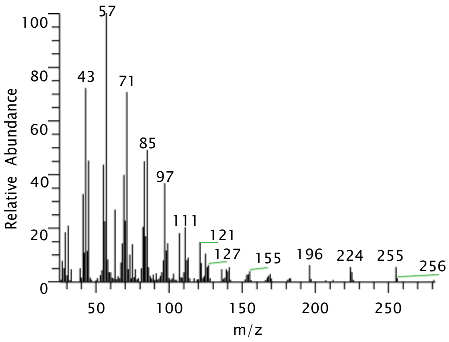

Raw data - Library entry

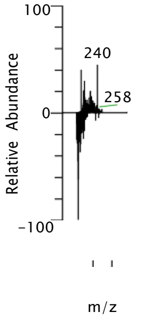

Ethanol, 2-(hexadecyloxy)-
Formula C18H38O2, MW 286, CAS# 2136-71-2, Entry# 5480
2-
Hexadecoxyethanol

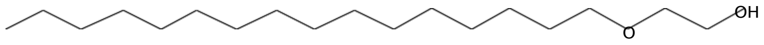

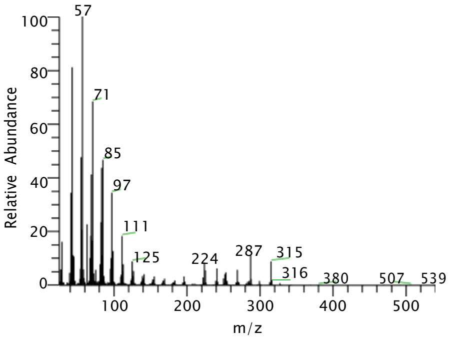

Raw data - Library entry

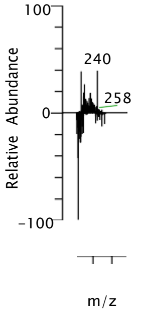

Octadecane, 1-[2-(hexadecyloxy)ethoxy]-
Formula C36H74O2, MW 538, CAS# 17367-10-1, Entry# 5454
Ethane, 1-(hexadecyloxy)-2-(octadecyloxy)-

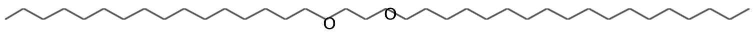

My Qual X-Report

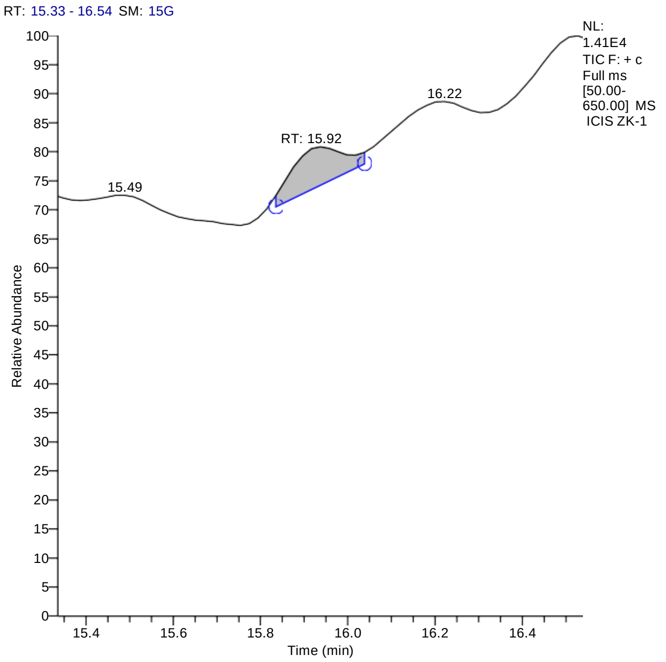

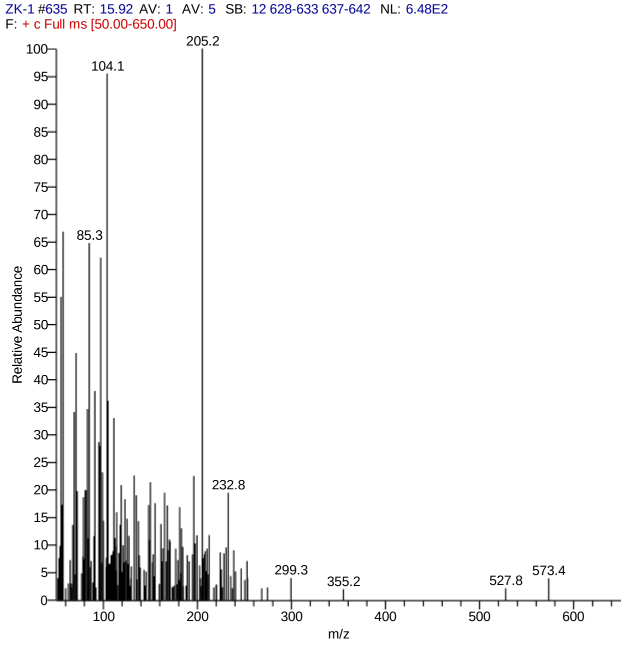

SI	RSI	Compound Name	Library	Probability	Area %	Area	RT
520	550	2(3H)-Furanone, dihydro-5-tetradecyl-	MAINLIB	18.58	0.15	8084.64	15.92
512	540	Tetrapentacontane, 1,54-dibromo-	MAINLIB	13.86	0.15	8084.64	15.92
506	575	2-(5-Acetyl-3-cyano-6-methyl-pyridin-2-ylsulfanyl)-N-(3-trifluoromethyl-phenyl)-acetamide	MAINLIB	10.89	0.15	8084.64	15.92

Hit Spectrum | Delta | Compound Structure

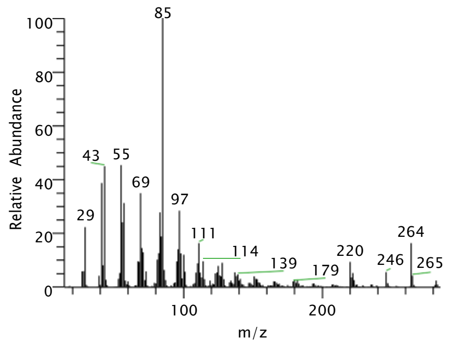

Raw data - Library entry

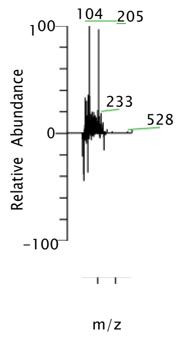

2(3H)-Furanone, dihydro-5-tetradecyl-
Formula C18H34O2, MW 282, CAS# 502-26-1, Entry# 43052
ç-Stearolactone

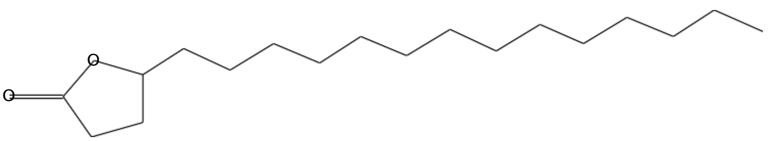

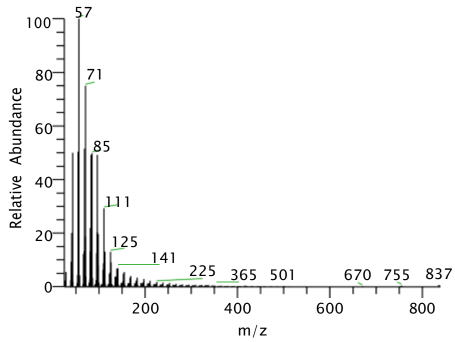

Raw data - Library entry

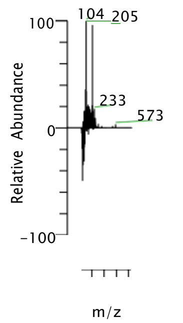

Tetrapentacontane, 1,54-dibromo-
Formula C54H108Br2, MW 914, CAS# NA, Entry# 21687

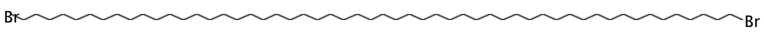

My Qual X-Report

Hit Spectrum	Delta	Compound Structure
	Raw data - Library entry	2-(5-Acetyl-3-cyano-6-methyl-pyridin-2-ylsulfanyl)-N-(3-trifluoromethyl-phenyl)-acetamide<br>Formula C18H14F3N3O2S, MW 393, CAS# NA, Entry# 6240

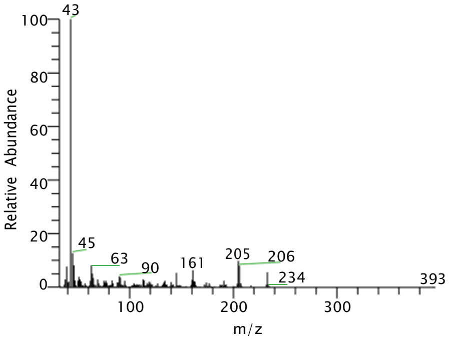

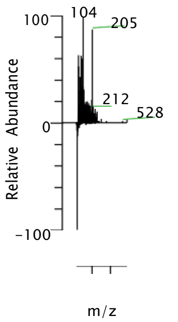

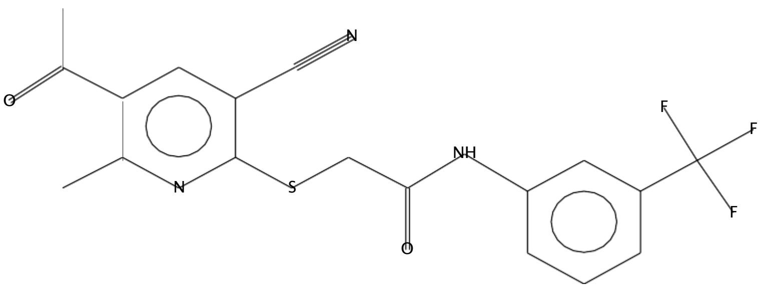

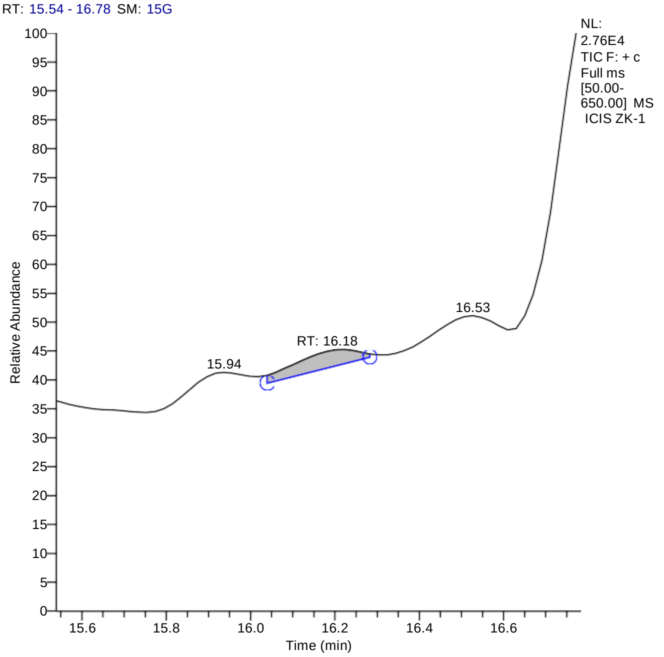

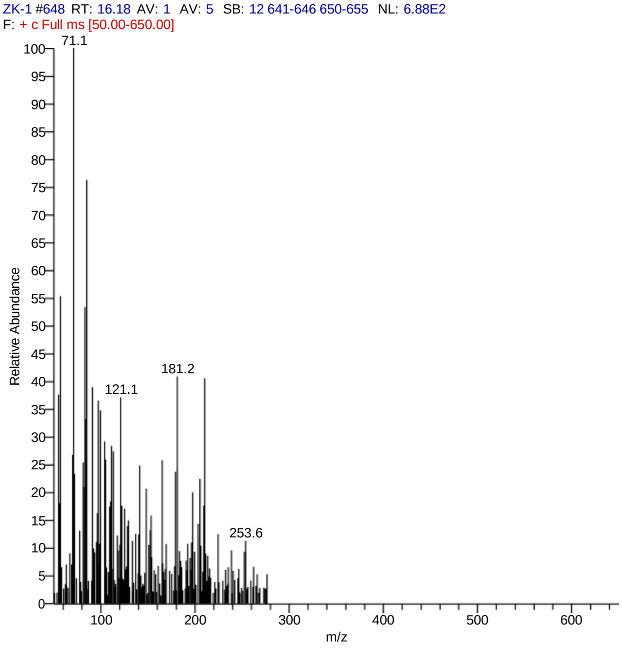

SI	RSI	Compound Name	Library	Probability	Area %	Area	RT
544	568	trans-1,2-Diaminocyclohexane-N,N,N',N'-tetraacetic acid	MAINLIB	20.39	0.16	8806.89	16.18
524	532	Oxirane-2-carboxylic acid, 3-(3,4,5-trimethoxyphenyl)-, methyl ester	MAINLIB	9.29	0.16	8806.89	16.18
520	520	Octadecane, 1-[2-(hexadecyloxy)ethoxy]-	MAINLIB	7.84	0.16	8806.89	16.18

Hit Spectrum	Delta	Compound Structure
	Raw data - Library entry	trans-1,2-Diaminocyclohexane-N,N,N',N'-tetraacetic acid<br>Formula C14H22N2O8, MW 346, CAS# 13291-61-7, Entry# 4045<br>Cyclohexane-1,2-diaminetetraacetic acid

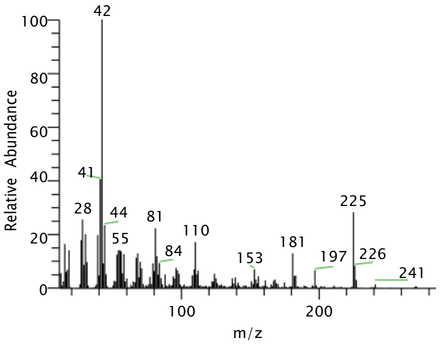

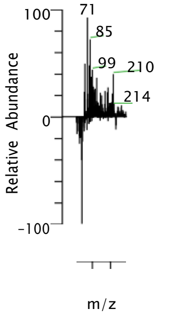

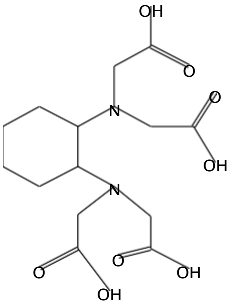

My Qual X-Report

Hit Spectrum | Delta | Compound Structure

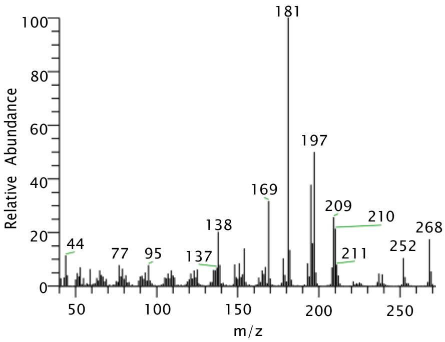

Raw data - Library entry

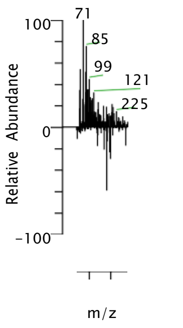

Oxirane-2-carboxylic acid, 3-(3,4,5-trimethoxyphenyl)-, methyl ester
Formula C13H16O6, MW 268, CAS# 14546-70-4, Entry# 115043
Oxirane-2-carboxylic acid,3-[3,4,5-trimethoxyphenyl]-, methyl ester

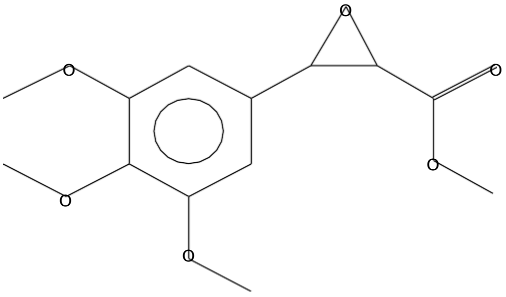

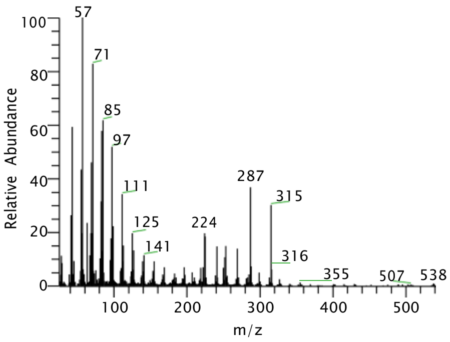

Raw data - Library entry

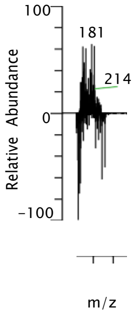

Octadecane, 1-[2-(hexadecyloxy)ethoxy]-
Formula C36H74O2, MW 538, CAS# 17367-10-1, Entry# 21707
Ethane, 1-(hexadecyloxy)-2-(octadecyloxy)-

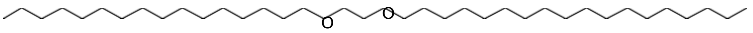

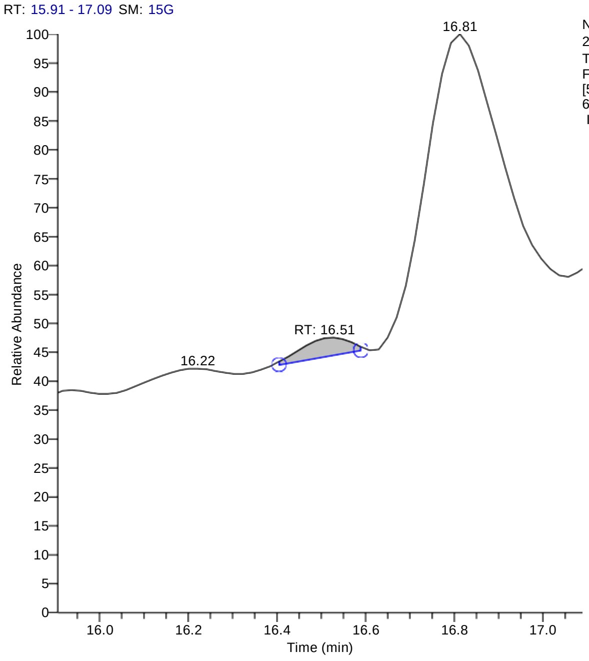

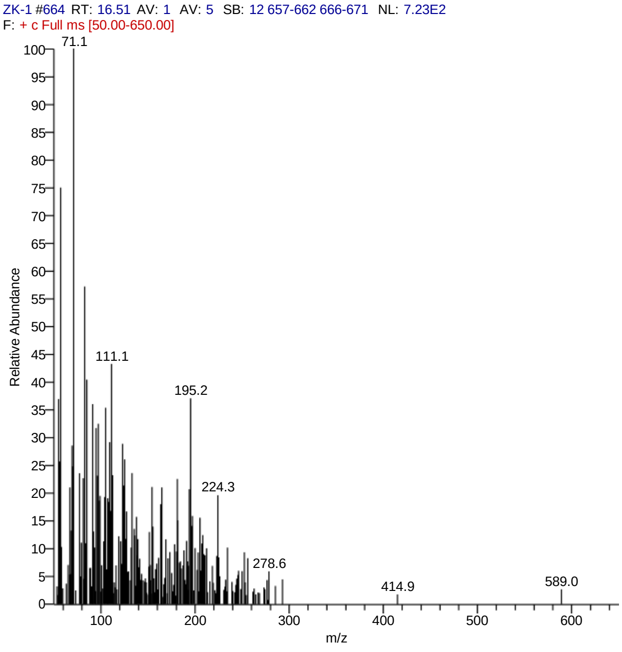

SI	RSI	Compound Name	Library	Probability	Area %	Area	RT
613	616	17-Pentatriacontene	MAINLIB	13.14	0.14	7292.53	16.51
595	634	tert-Hexadecanethiol	MAINLIB	6.78	0.14	7292.53	16.51
593	651	1-Methyl-8-propyl-3,6-diazahomoadamantan-9-ol	MAINLIB	6.25	0.14	7292.53	16.51

My Qual X-Report

Hit Spectrum	Delta	Compound Structure

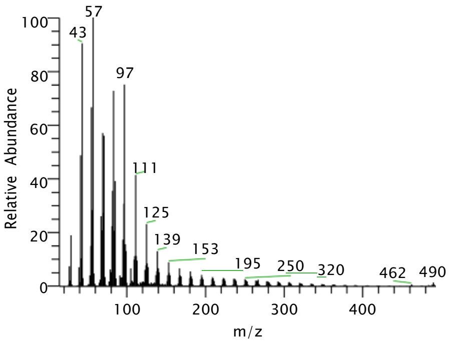

Raw data - Library entry

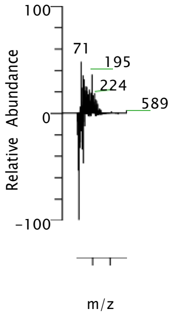

17-Pentatriacontene
Formula C35H70, MW 490, CAS# 6971-40-0, Entry# 21018
(17E)-17-Pentatriacontene #

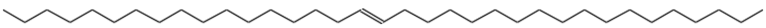

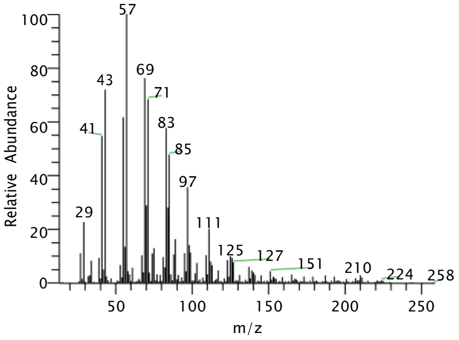

Raw data - Library entry

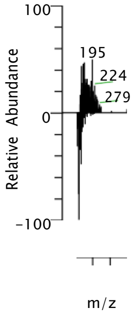

tert-Hexadecanethiol
Formula C16H34S, MW 258, CAS# 25360-09-2, Entry# 21483
1,1-Dimethyltetradecyl hydrosulfide #

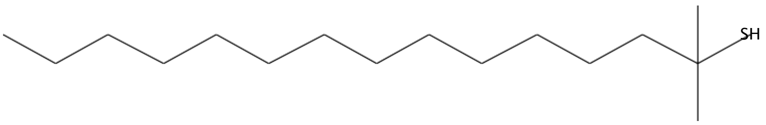

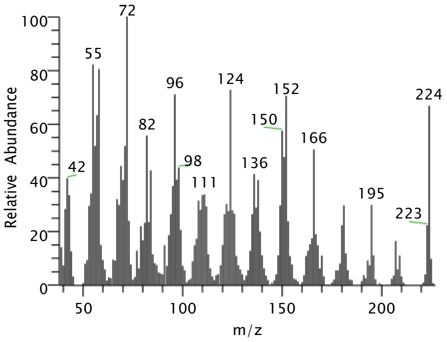

Raw data - Library entry

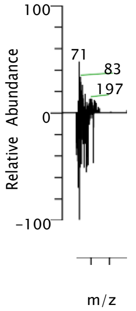

1-Methyl-8-propyl-3,6-diazahomoadamantan-9-ol
Formula C13H24N2O, MW 224, CAS# 138023-30-0, Entry# 31655

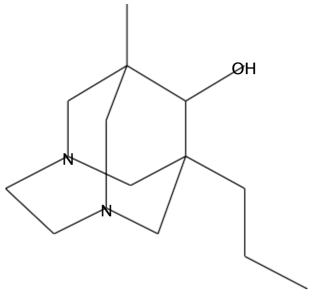

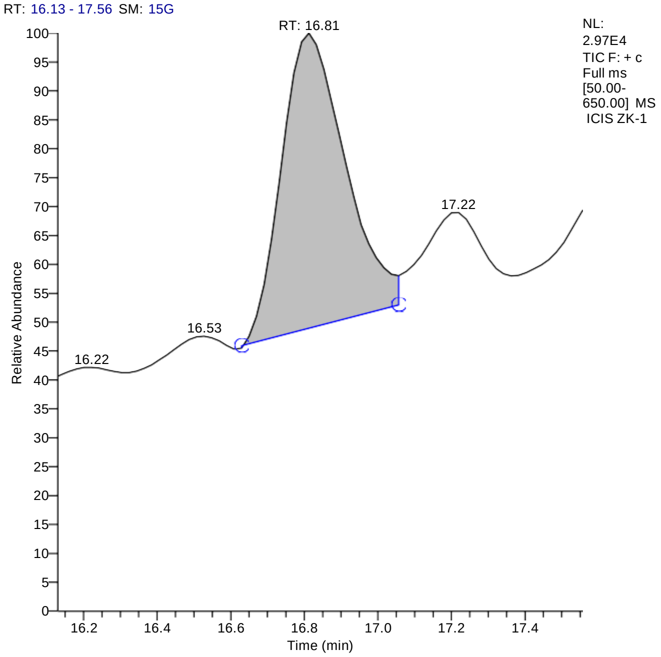

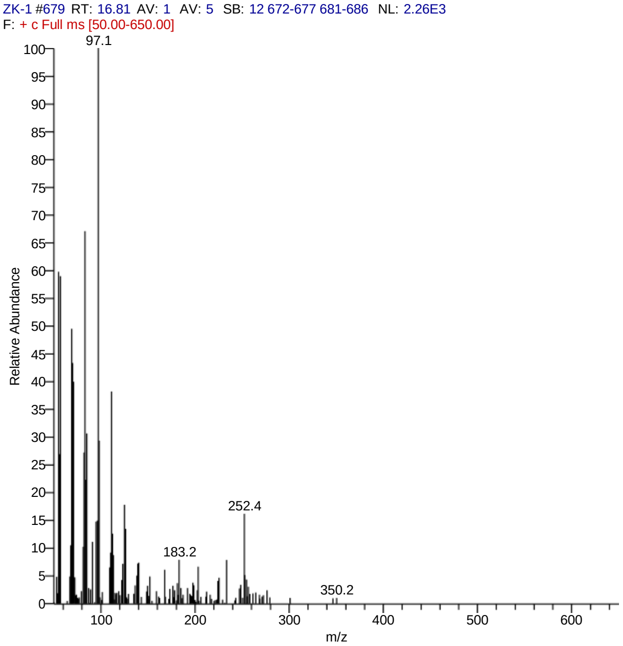

SI	RSI	Compound Name	Library	Probability	Area %	Area	RT
684	713	Bacteriochlorophyll-c-stearyl	MAINLIB	6.33	3.39	182892.89	16.81

My Qual X-Report

SI	RSI	Compound Name	Library	Probability	Area %	Area	RT
673	787	1-Octadecanol	replib	4.34	3.39	182892.89	16.81
673	775	1-Octadecene	replib	4.34	3.39	182892.89	16.81

Hit Spectrum | Delta | Compound Structure

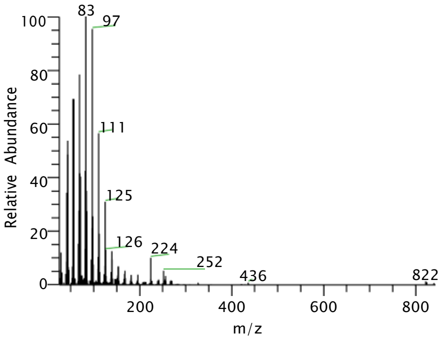

Raw data - Library entry

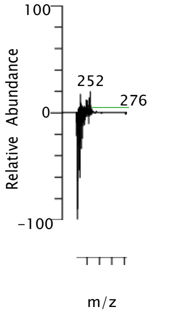

Bacteriochlorophyll-c-stearyl
Formula C52H72MgN4O4, MW 840, CAS# NA, Entry# 41593

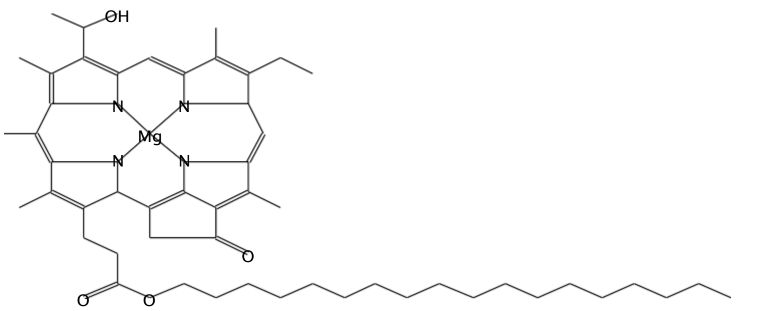

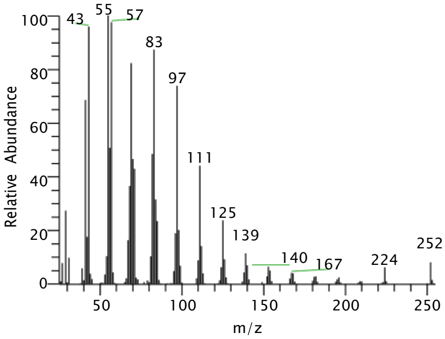

Raw data - Library entry

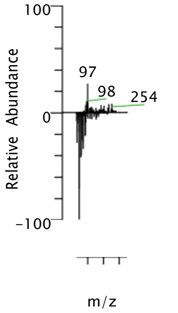

1-Octadecanol
Formula C18H38O, MW 270, CAS# 112-92-5, Entry# 4405
n-Octadecanol

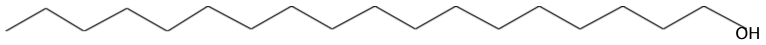

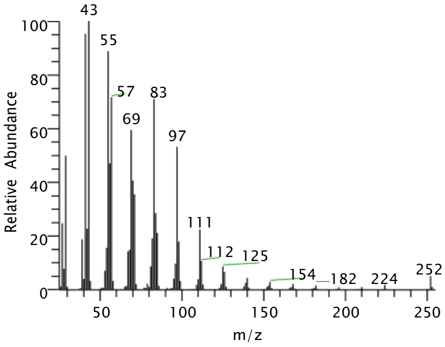

Raw data - Library entry

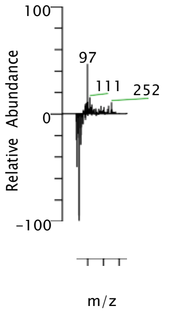

1-Octadecene
Formula C18H36, MW 252, CAS# 112-88-9, Entry# 1671
à-Octadecene

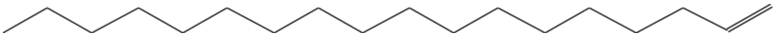

My Qual X-Report

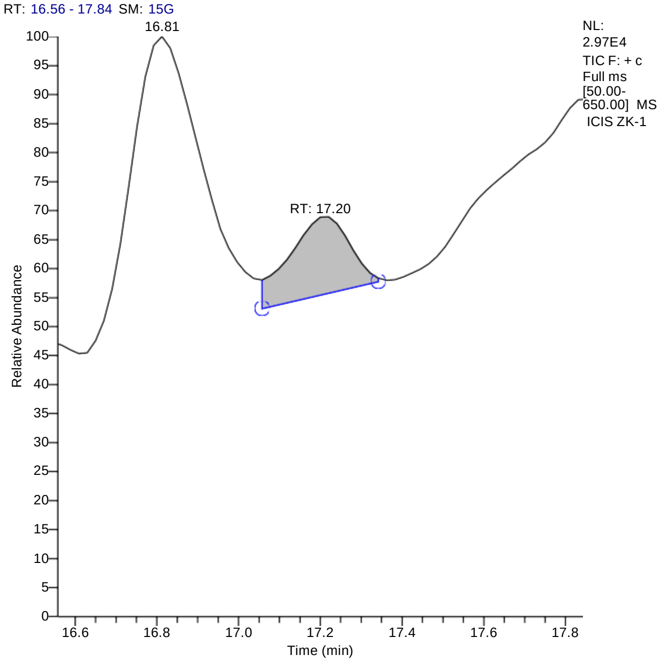

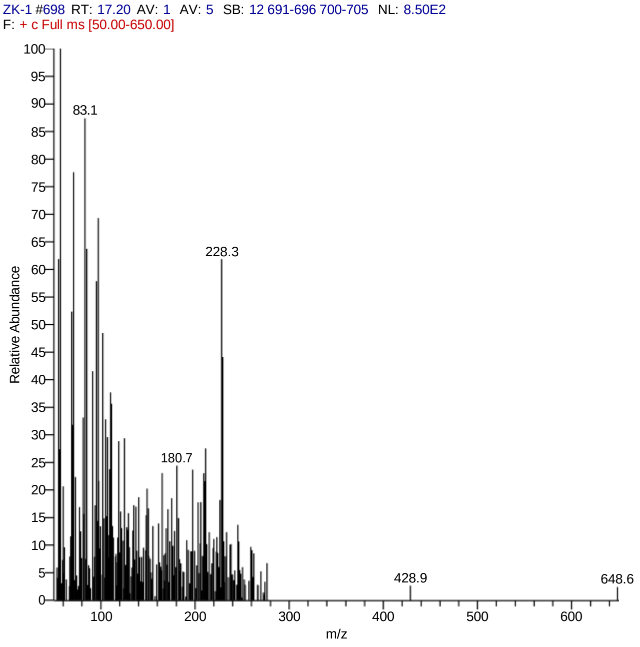

SI	RSI	Compound Name	Library	Probability	Area %	Area	RT
584	594	2-Nonadecanone 2,4-dinitrophenylhydrazine	MAINLIB	21.32	0.77	41543.94	17.20
571	574	17-Pentatriacontene	MAINLIB	13.77	0.77	41543.94	17.20
562	578	1,3-O-Benzylidene glyceryl-2-myristate	MAINLIB	10.00	0.77	41543.94	17.20

Hit Spectrum | Delta | Compound Structure

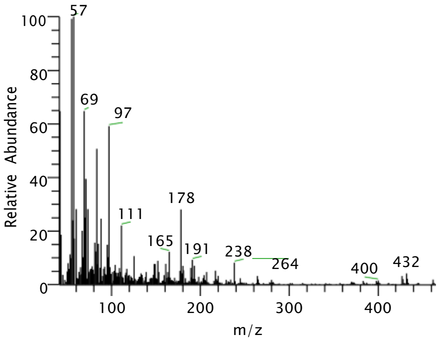

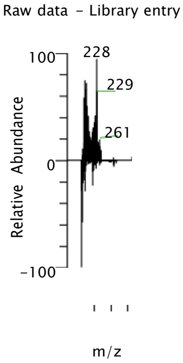

2-Nonadecanone 2,4-dinitrophenylhydrazine
Formula C25H42N4O4, MW 462, CAS# 28813-61-8, Entry# 21203
2-Nonadecanone, (2,4-dinitrophenyl)hydrazone

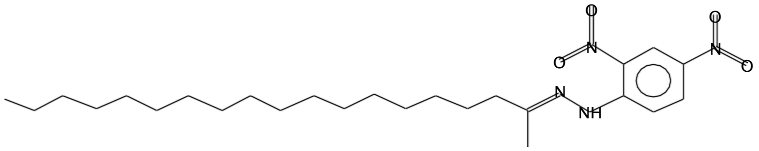

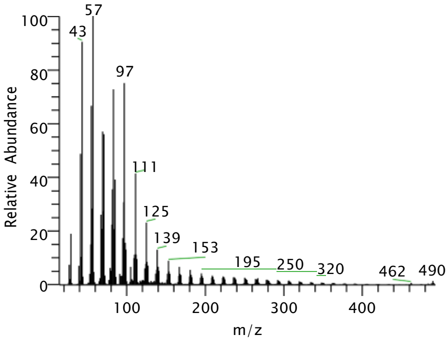

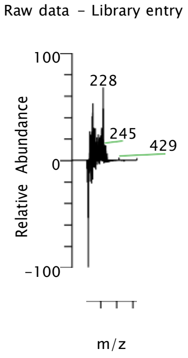

17-Pentatriacontene
Formula C35H70, MW 490, CAS# 6971-40-0, Entry# 21018
(17E)-17-Pentatriacontene #

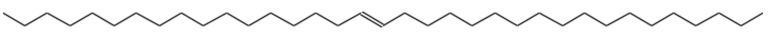

My Qual X-Report

Hit Spectrum | Delta | Compound Structure

Raw data - Library entry

1,3-O-Benzylidene glyceryl-2-myristate
Formula C24H38O3, MW 374, CAS# NA, Entry# 5301

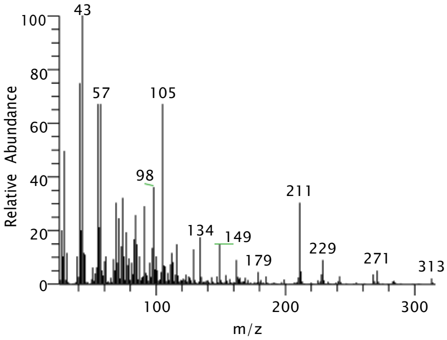

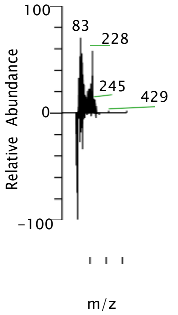

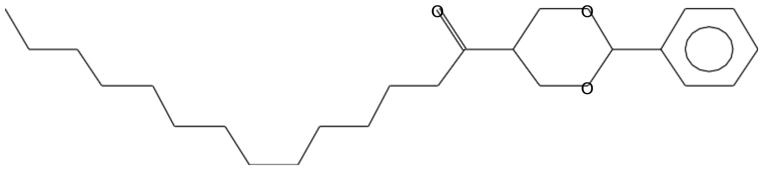

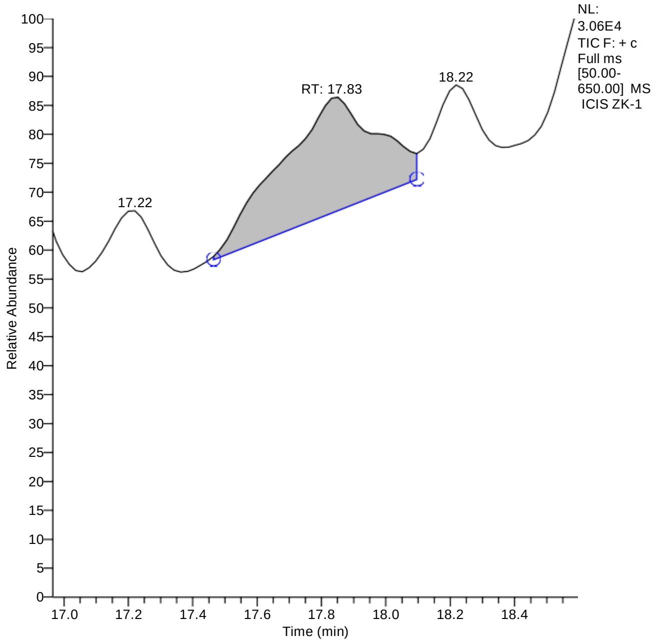

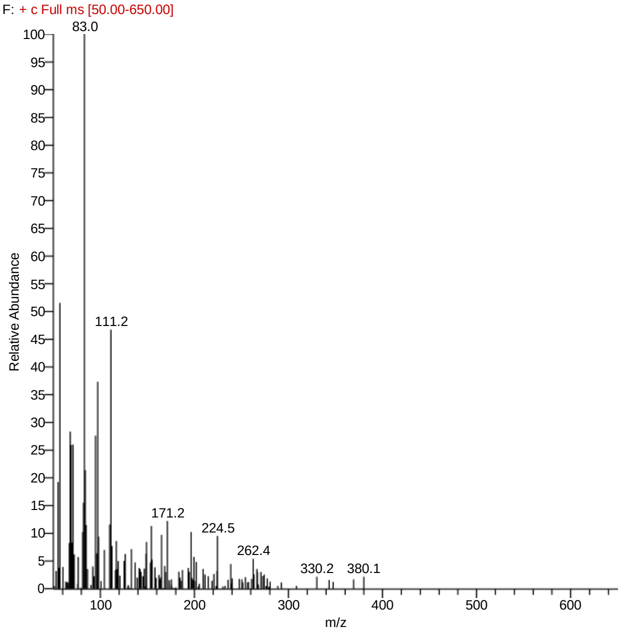

SI	RSI	Compound Name	Library	Probability	Area %	Area	RT
587	624	17-Pentatriacontene	replib	18.29	2.38	128725.65	17.83
565	620	1-Docosanol	replib	7.23	2.38	128725.65	17.83
551	589	Bacteriochlorophyll-c-stearyl	MAINLIB	4.52	2.38	128725.65	17.83

Hit Spectrum | Delta | Compound Structure

Raw data - Library entry

17-Pentatriacontene
Formula C35H70, MW 490, CAS# 6971-40-0, Entry# 2071
(17E)-17-Pentatriacontene #

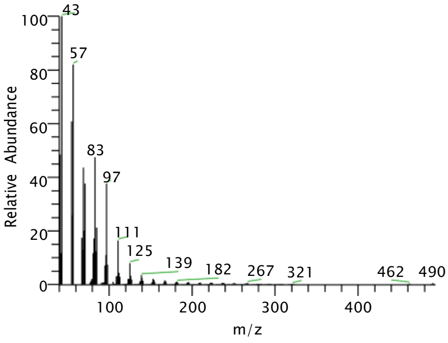

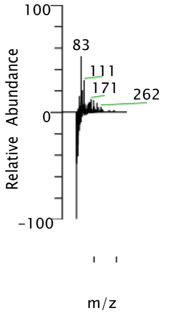

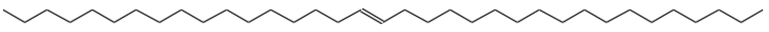

My Qual X-Report

Hit Spectrum	Delta	Compound Structure

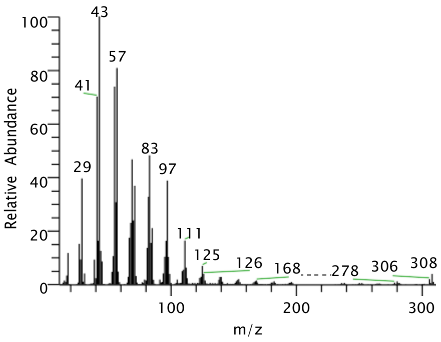

Raw data - Library entry

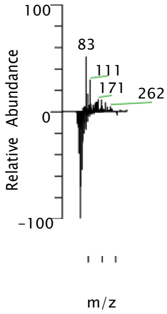

1-Docosanol
Formula C22H46O, MW 326, CAS# 661-19-8, Entry# 2083
Behenic alcohol

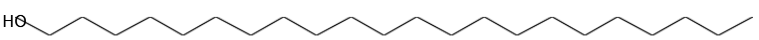

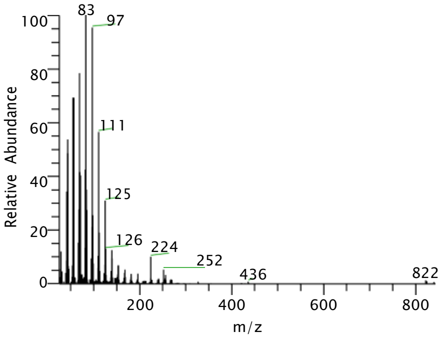

Raw data - Library entry

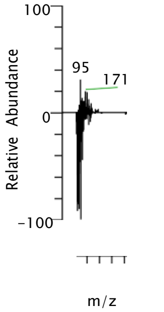

Bacteriochlorophyll-c-stearyl
Formula C52H72MgN4O4, MW 840, CAS# NA, Entry# 41593

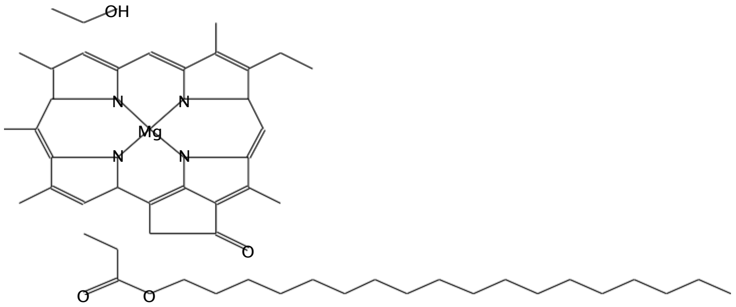

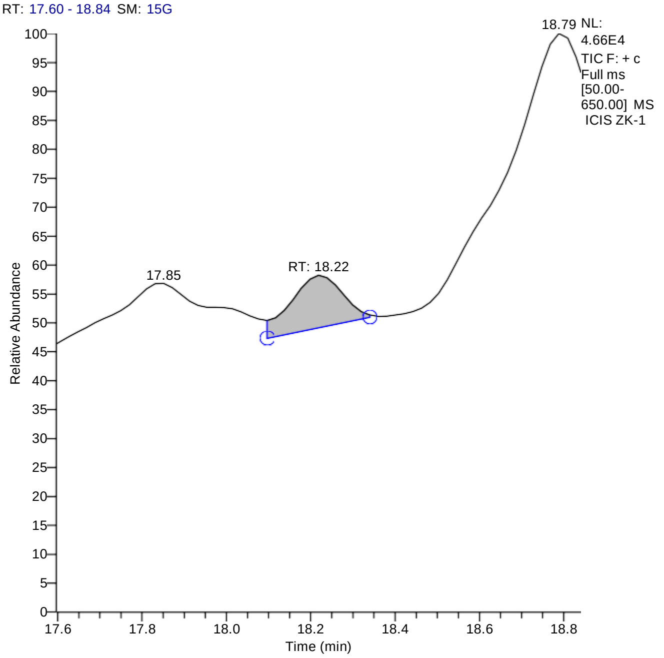

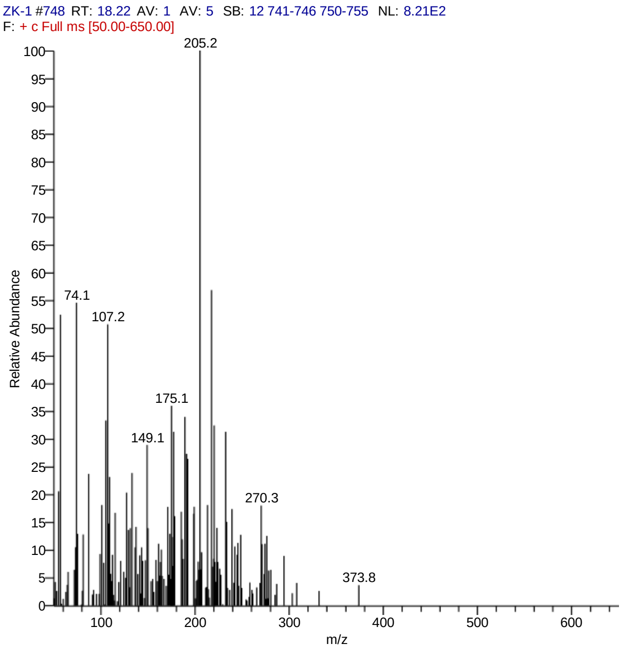

SI	RSI	Compound Name	Library	Probability	Area %	Area	RT
573	738	7,9-Di-tert-butyl-1-oxaspiro(4,5)deca-6, 9-diene-2,8-dione	MAINLIB	57.96	0.68	36702.42	18.22
571	660	7,9-Di-tert-butyl-1-oxaspiro(4,5)deca-6, 9-diene-2,8-dione	replib	57.96	0.68	36702.42	18.22
534	556	Emetan, 1',2'-didehydro-6',7',10,11-tetramethoxy-	MAINLIB	13.66	0.68	36702.42	18.22

My Qual X-Report

Hit Spectrum | Delta | Compound Structure

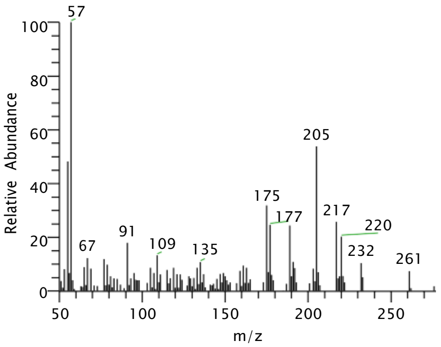

Raw data - Library entry

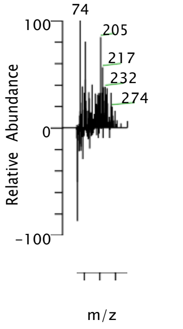

7,9-Di-tert-butyl-1-oxaspiro(4,5)deca-6,9-diene-2,8-dione
Formula C17H24O3, MW 276, CAS# 82304-66-3, Entry# 22806
1-Oxa-spiro[4.5]deca-6,9-diene-2,8-dione, 7,9-di-tert-butyl-

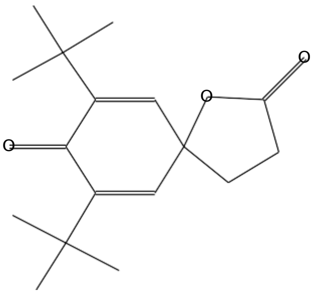

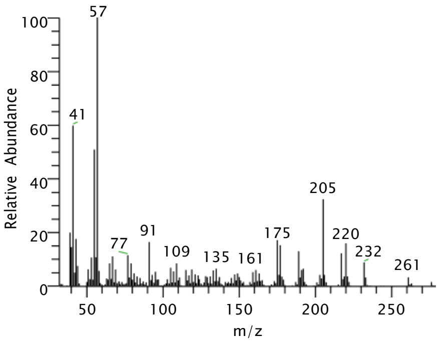

Raw data - Library entry

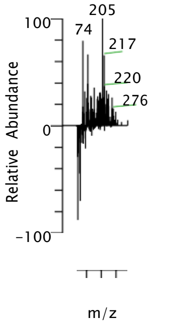

7,9-Di-tert-butyl-1-oxaspiro(4,5)deca-6,9-diene-2,8-dione
Formula C17H24O3, MW 276, CAS# 82304-66-3, Entry# 5238
1-Oxa-spiro[4.5]deca-6,9-diene-2,8-dione, 7,9-di-tert-butyl-

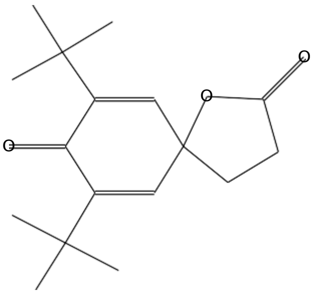

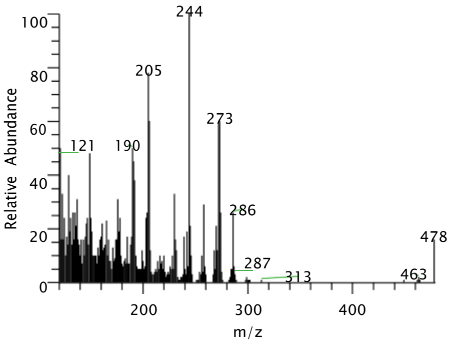

Raw data - Library entry

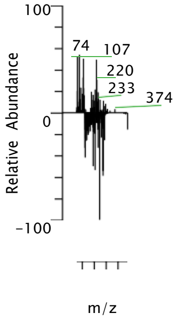

Emetan, 1',2'-didehydro-6',7',10,11-tetramethoxy-
Formula C29H38N2O4, MW 478, CAS# 523-01-3, Entry# 138986
Emetine, 1',2'-didehydro-

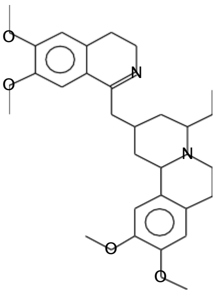

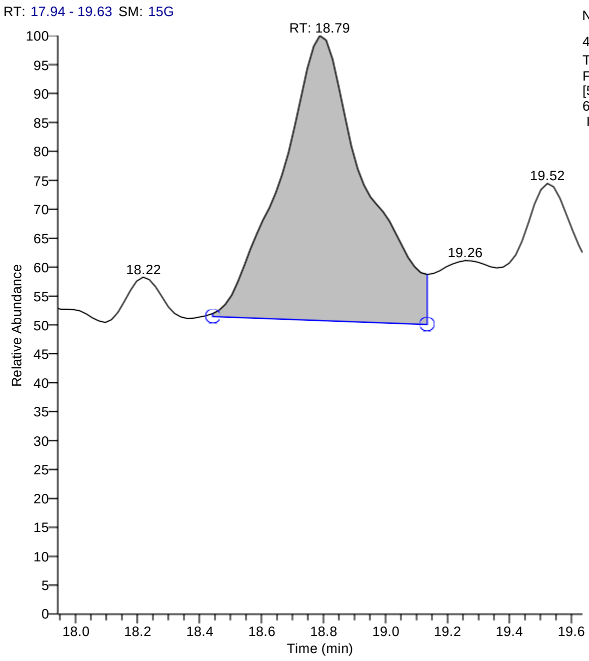

NL:
4.66E4
TIC F: + c Full ms [50.00-650.00] MS ICIS ZK-1

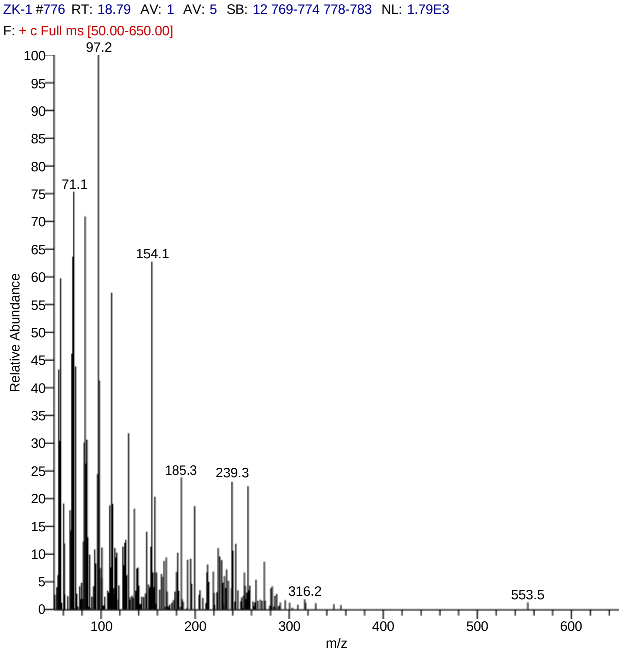

My Qual X-Report

SI	RSI	Compound Name	Library	Probability	Area %	Area	RT
592	600	Hexadecanoic acid, 1-(hydroxymethyl)-1,2-ethanediyl ester	MAINLIB	17.75	8.08	436560.90	18.79
573	642	Estra-1,3,5(10)-trien-17á-ol	MAINLIB	8.62	8.08	436560.90	18.79
564	611	Palmitic anhydride	MAINLIB	6.25	8.08	436560.90	18.79

Hit Spectrum | Delta | Compound Structure

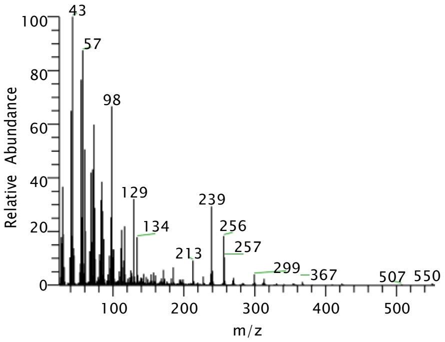

Raw data - Library entry

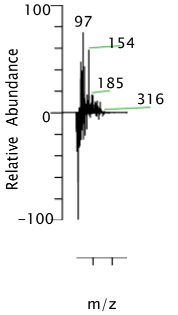

Hexadecanoic acid, 1-(hydroxymethyl)-1,2-ethanediyl ester
Formula C35H68O5, MW 568, CAS# 761-35-3, Entry# 6952
Palmitin, 1,2-di-

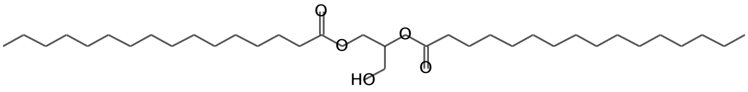

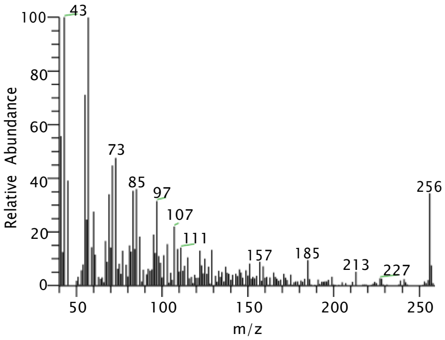

Raw data - Library entry

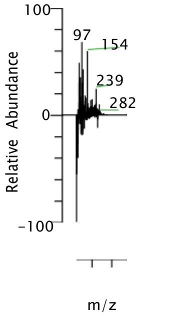

Estra-1,3,5(10)-trien-17á-ol
Formula C18H24O, MW 256, CAS# 2529-64-8, Entry# 6965
Estra-1,3,5(10)-trien-17-ol, (17á)-

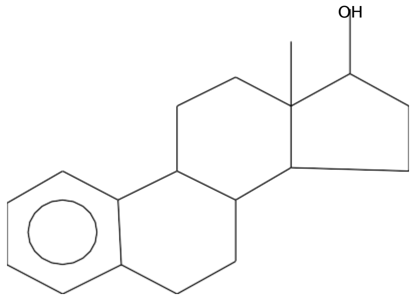

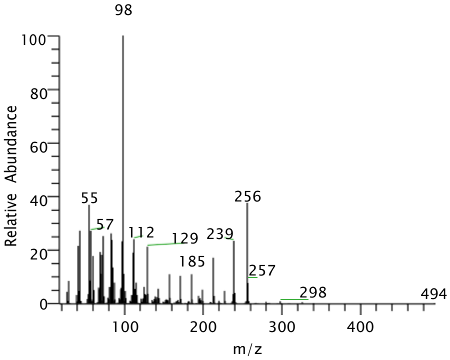

Raw data - Library entry

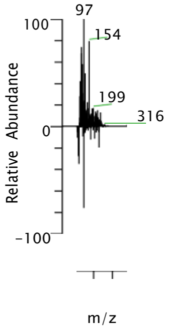

Palmitic anhydride
Formula C32H62O3, MW 494, CAS# 623-65-4, Entry# 55804
Hexadecanoic acid, anhydride

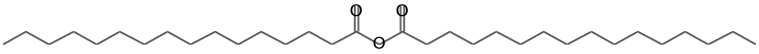

My Qual X-Report

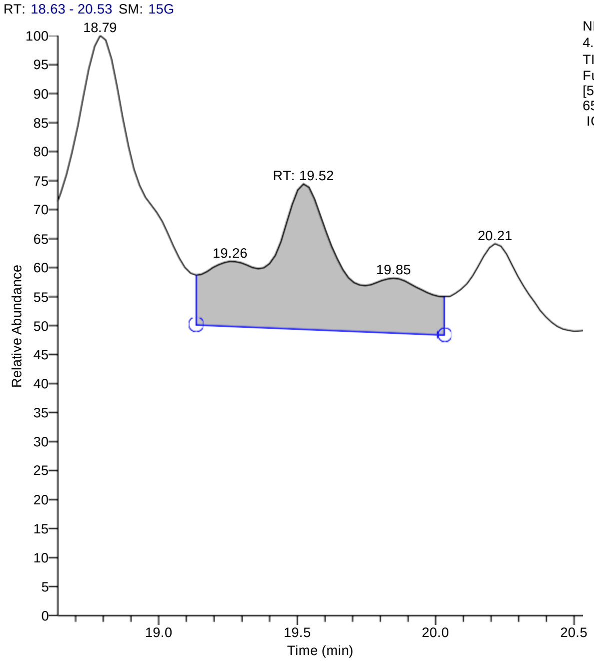

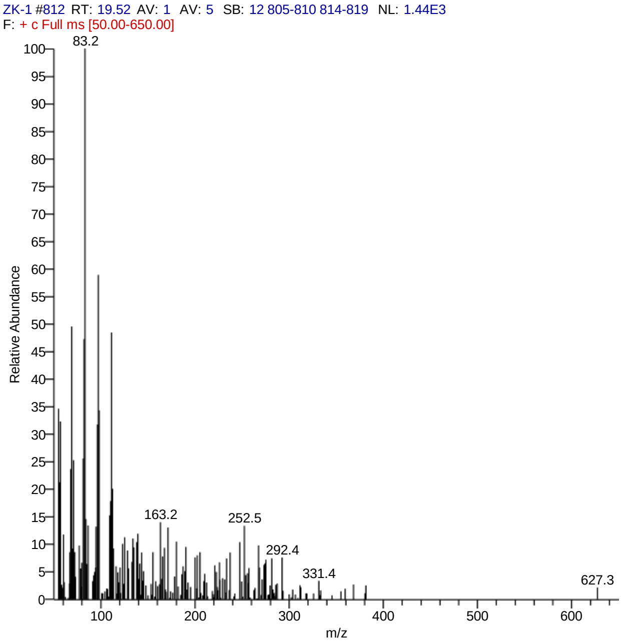

SI	RSI	Compound Name	Library	Probability	Area %	Area	RT
536	586	17-Pentatriacontene	replib	4.71	5.53	298557.26	19.52
534	601	Bacteriochlorophyll-c-stearyl	MAINLIB	4.34	5.53	298557.26	19.52
526	600	5-Octadecenal	MAINLIB	3.24	5.53	298557.26	19.52

Hit Spectrum | Delta | Compound Structure

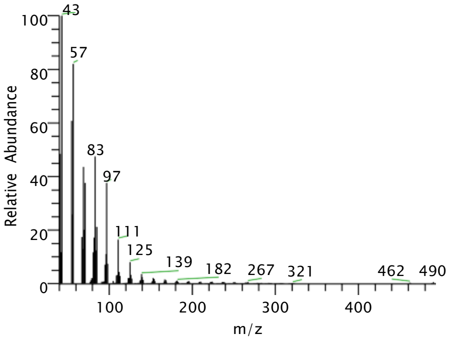

Raw data - Library entry

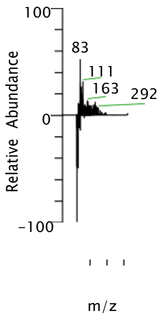

17-Pentatriacontene
Formula C35H70, MW 490, CAS# 6971-40-0, Entry# 2071
(17E)-17-Pentatriacontene #

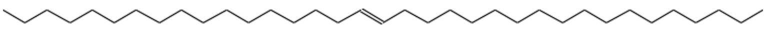

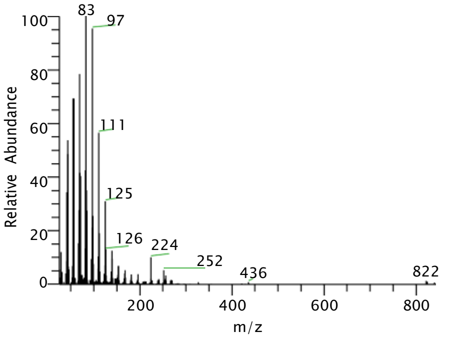

Raw data - Library entry

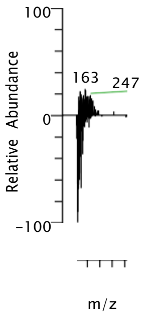

Bacteriochlorophyll-c-stearyl
Formula C52H72MgN4O4, MW 840, CAS# NA, Entry# 41593

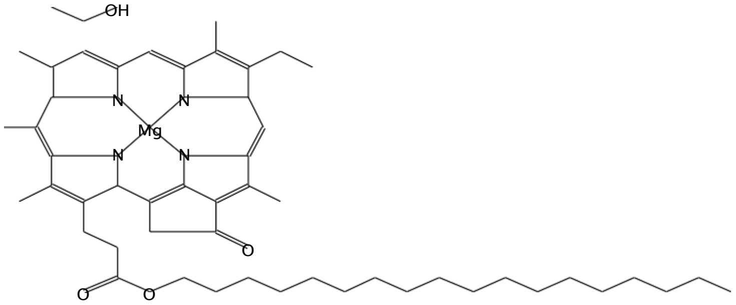

My Qual X-Report

Hit Spectrum	Delta	Compound Structure

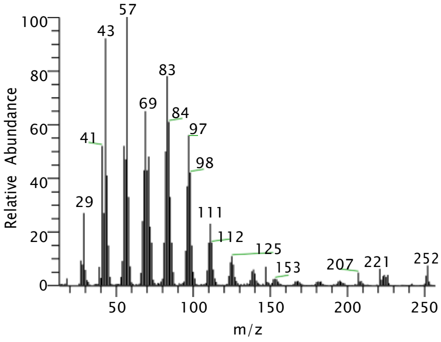

Raw data - Library entry

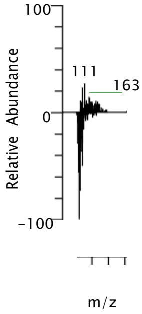

5-Octadecenal
Formula C18H34O, MW 266, CAS# 56554-88-2, Entry# 20992
(5E)-5-Octadecenal #

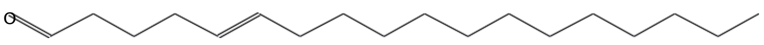

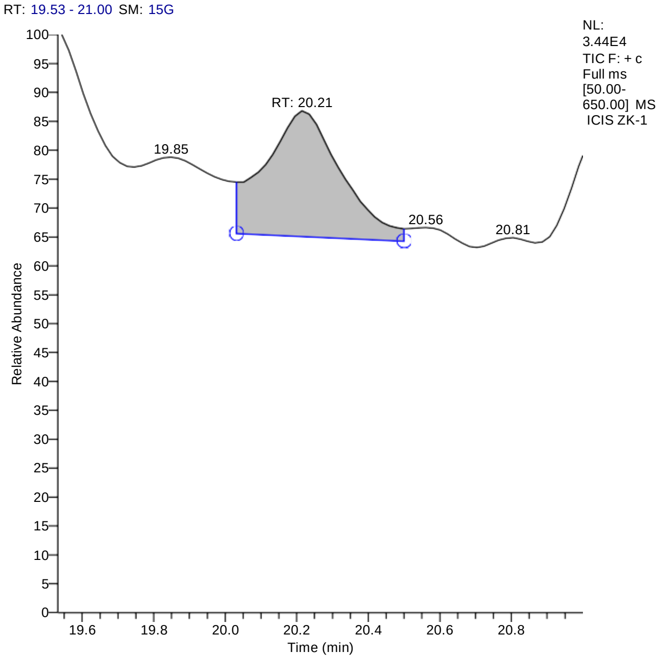

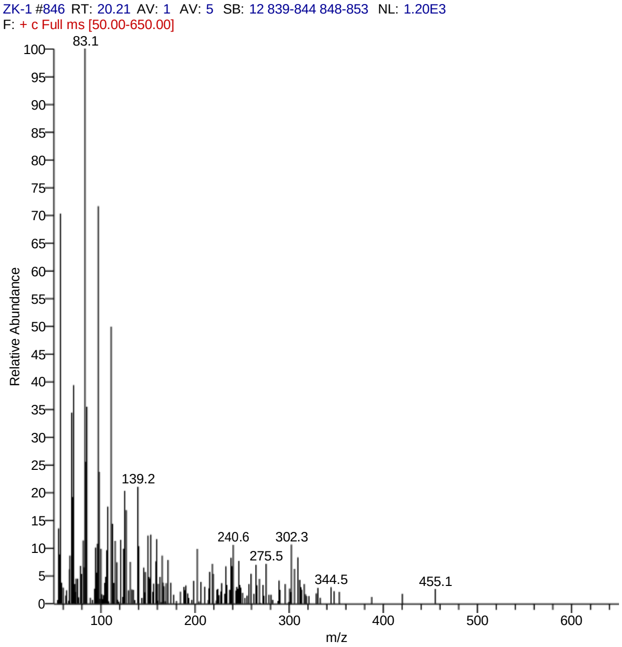

SI	RSI	Compound Name	Library	Probability	Area %	Area	RT
523	529	17-Pentatriacontene	MAINLIB	16.05	2.07	111976.95	20.21
519	568	1-Tricosanol	replib	13.56	2.07	111976.95	20.21
487	511	1-Hexacosanol	replib	3.62	2.07	111976.95	20.21

Hit Spectrum	Delta	Compound Structure

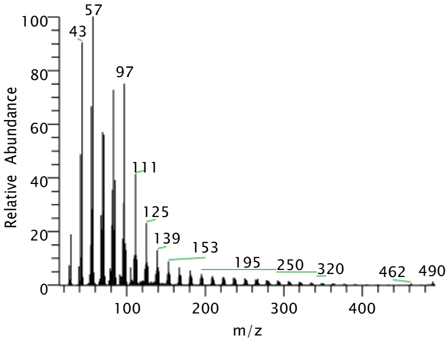

Raw data - Library entry

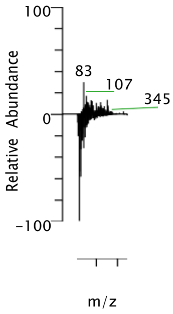

17-Pentatriacontene
Formula C35H70, MW 490, CAS# 6971-40-0, Entry# 21018
(17E)-17-Pentatriacontene #

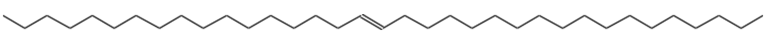

My Qual X-Report

Hit Spectrum | Delta | Compound Structure

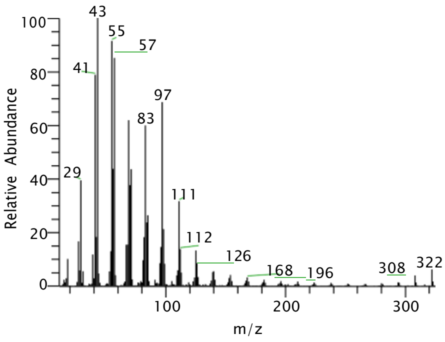

Raw data - Library entry

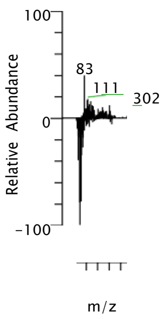

1-Tricosanol
Formula C23H48O, MW 340, CAS# 3133-01-5, Entry# 1931
Tricosanol

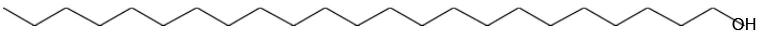

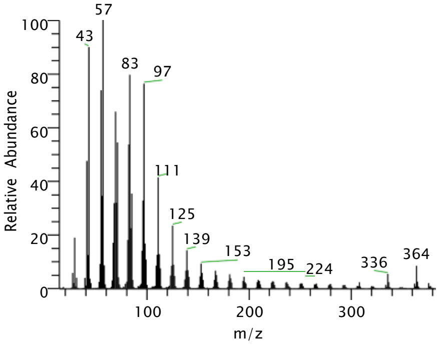

Raw data - Library entry

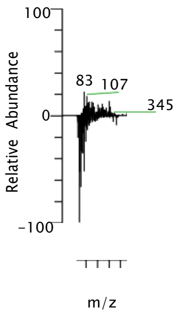

1-Hexacosanol
Formula C26H54O, MW 382, CAS# 506-52-5, Entry# 5523
n-Hexacosanol

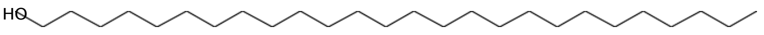

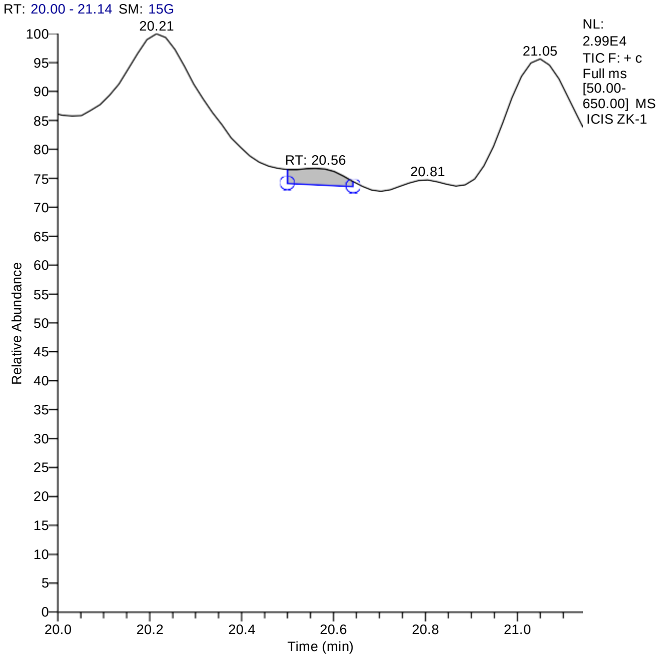

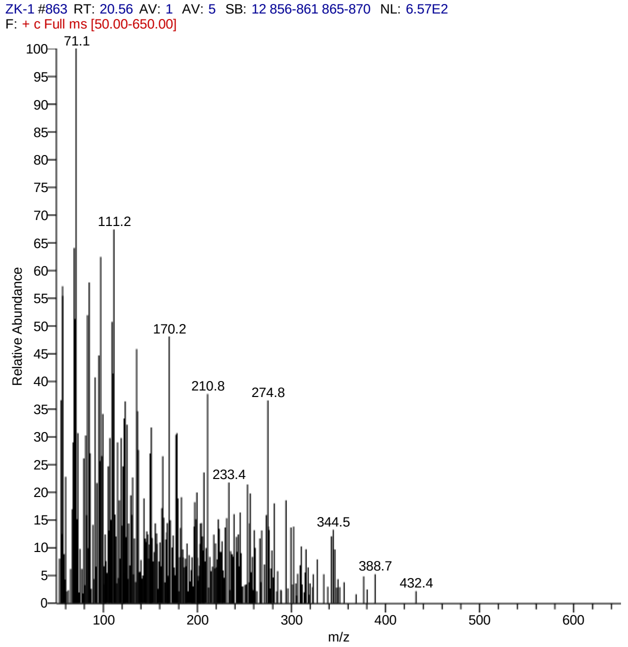

SI	RSI	Compound Name	Library	Probability	Area %	Area	RT
632	632	3-Pyridinecarboxylic acid, 2,7,10-tris(acetyloxy)-1,1a,2,3,4,6,7,10,11,11a-decahydro-1,1,3,6,9-pentamethyl-4-oxo-4a,7a-epoxy-5H-cyclopenta[a]cyclopropa[f]cycloundecen-11-yl ester, [1aR-(1aR*,2R*,3S*,4aR*,6S*,7S*,7aS*,8E,10R*,11R*,11aS*)]-	MAINLIB	22.81	0.11	6154.09	20.56
631	650	Acetic acid, 5-cyano-10-formyl-13-methyl-3-oxohexadecahydrocyclopenta[a]phenanthren-17-yl ester	MAINLIB	21.93	0.11	6154.09	20.56
571	593	7-Hydroxy-9-[2-(3-hydroxy-hept-6-enyl)-[1,3]dithian-2-yl]-non-4-ynoic acid	MAINLIB	3.89	0.11	6154.09	20.56

My Qual X-Report

Hit Spectrum | Delta | Compound Structure

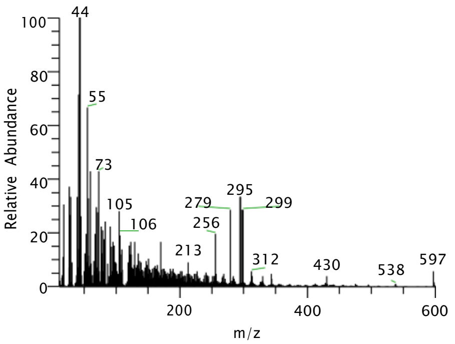

Raw data - Library entry

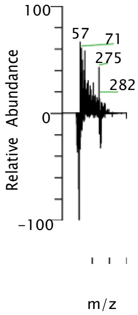

Formula C32H39NO10, MW 597, CAS# 51906-00-4, Entry# 6012

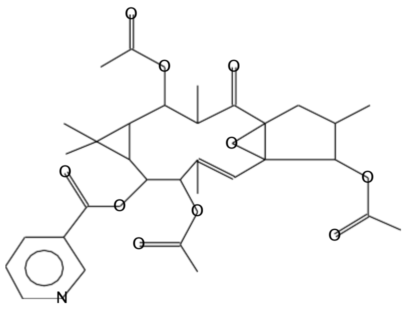

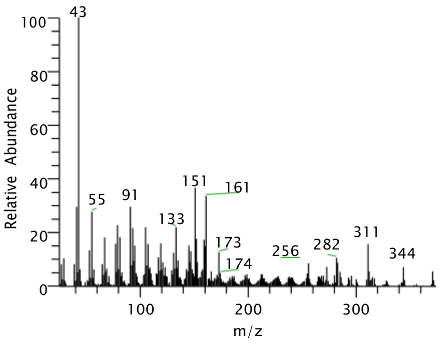

Raw data - Library entry

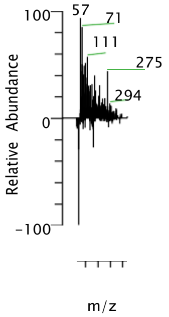

Formula C22H29NO4, MW 371, CAS# NA, Entry# 11057

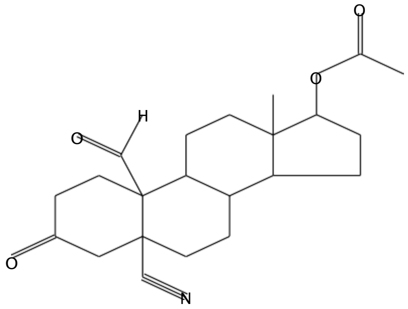

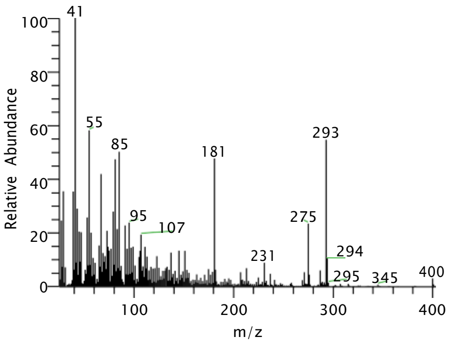

Raw data - Library entry

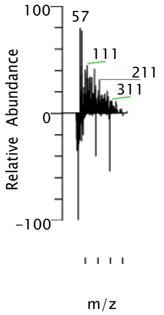

7-Hydroxy-9-[2-(3-hydroxy-hept-6-enyl)-[1,3]dithian-2-yl]-non-4-ynoic acid
Formula C20H32O4S2, MW 400, CAS# NA, Entry# 2638

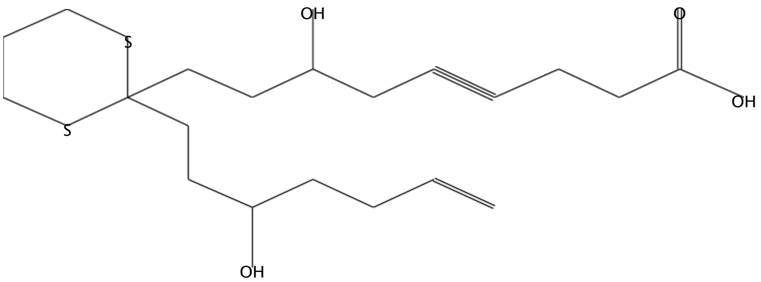

RT: 20.24 - 21.71 SM: 15G

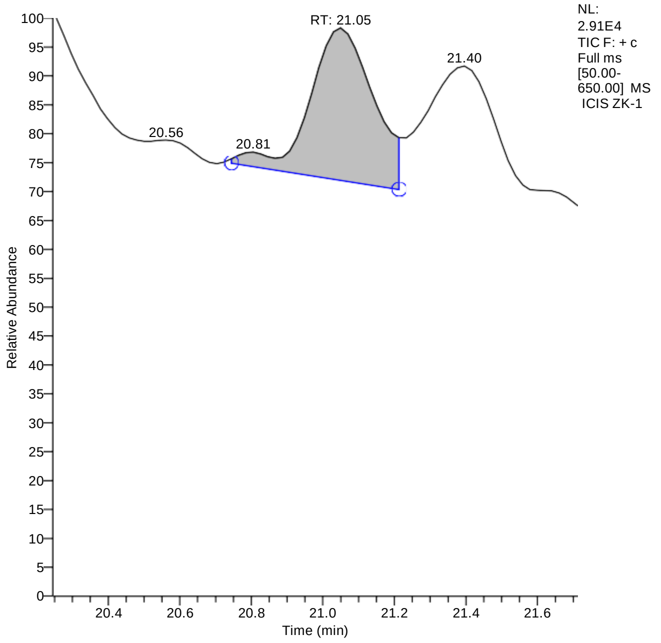

ZK-1 #887 RT: 21.05 AV: 1 AV: 5 SB: 12 880-885 889-894 NL: 4.56E3
F: + c Full ms [50.00-650.00]

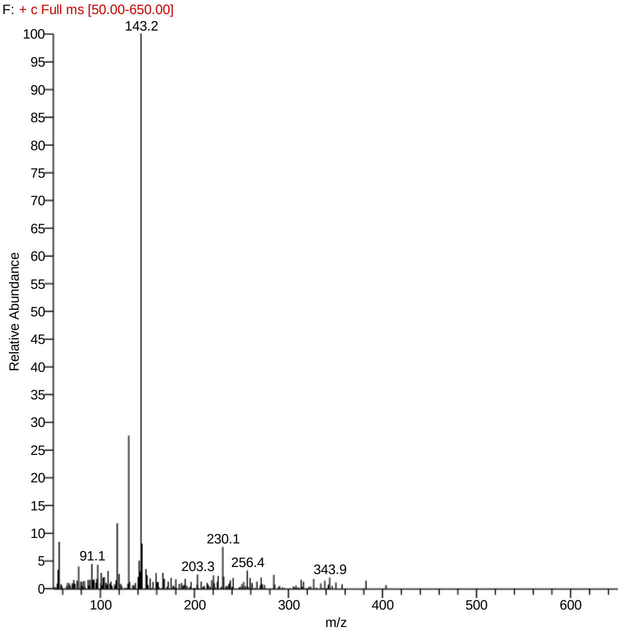

My Qual X-Report

SI	RSI	Compound Name	Library	Probability	Area %	Area	RT
504	711	Dodecanecarboxamide, N-[2-(3-indolyl)ethyl]-	MAINLIB	18.25	1.77	95523.40	21.05
491	642	2H-Pyrrol-2-one, 4-acetyl-5-(3-fluorophenyl)-1,5-dihydro -3-hydroxy-1-[2-(1H-indol-3-yl)ethyl]-	MAINLIB	11.79	1.77	95523.40	21.05
482	541	Tryptamine, N-[4-hydroxyhydrocinnamoyl]-	MAINLIB	8.56	1.77	95523.40	21.05

Hit Spectrum | Delta | Compound Structure

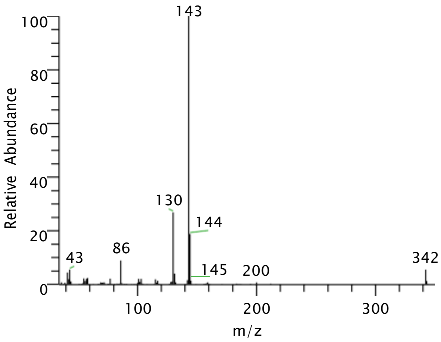

Raw data - Library entry

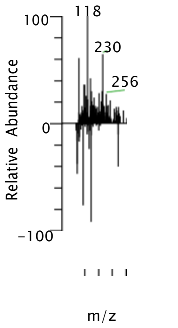

Dodecanecarboxamide, N-[2-(3-indolyl)ethyl]-
Formula C22H34N2O, MW 342, CAS# 21469-18-1, Entry# 91732
N-[2-(1H-Indol-3-yl)ethyl]dodecanamide #

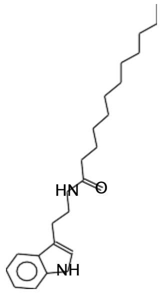

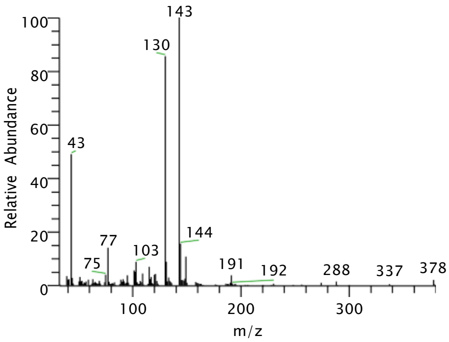

Raw data - Library entry

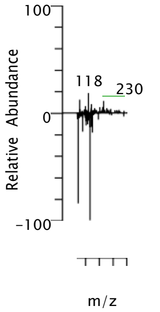

2H-Pyrrol-2-one, 4-acetyl-5-(3-fluorophenyl)-1,5-dihydro-3-hydroxy-1-[2-(1H-indol-3-yl)ethyl]-
Formula C22H19FN2O3, MW 378, CAS# NA, Entry# 91720

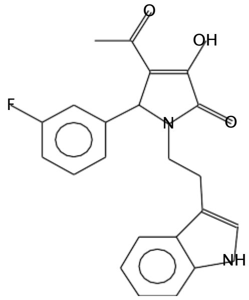

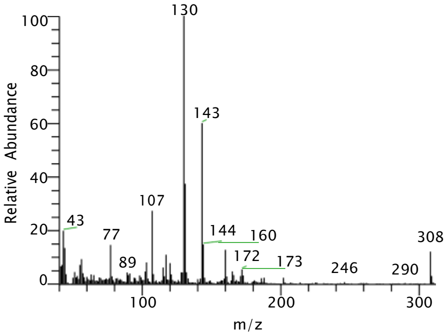

Raw data - Library entry

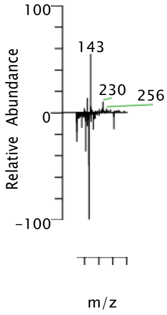

Tryptamine, N-[4-hydroxyhydrocinnamoyl]-
Formula C19H20N2O2, MW 308, CAS# 106827-63-8, Entry# 81616
3-(4-Hydroxyphenyl)-N-[2-(1H-indol-3-yl)ethyl]propanamide #

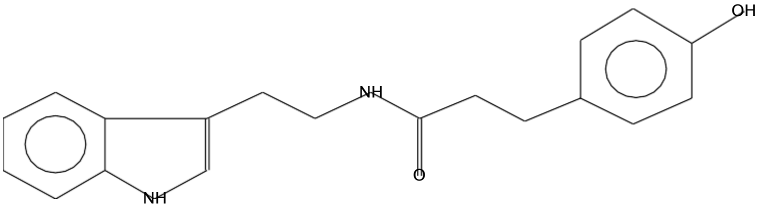

My Qual X-Report

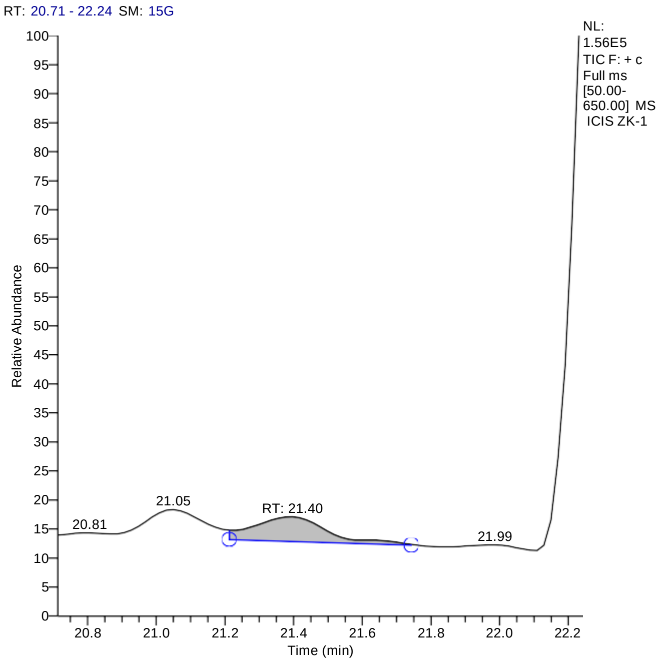

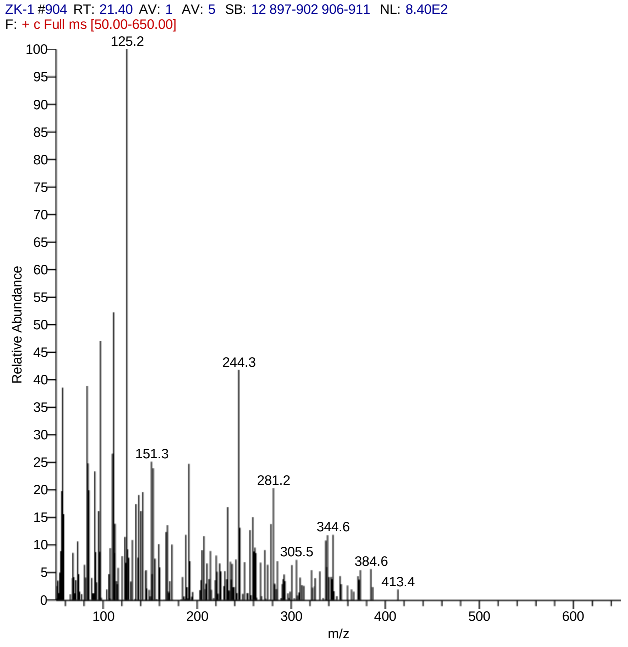

SI	RSI	Compound Name	Library	Probability	Area %	Area	RT
414	463	Erucic acid	replib	10.52	1.86	100586.15	21.40
411	453	6á-Hydroxyfluoxymesterone	MAINLIB	9.29	1.86	100586.15	21.40
401	412	Octadecane, 3-ethyl-5-(2-ethylbutyl)-	replib	6.56	1.86	100586.15	21.40

Hit Spectrum | Delta | Compound Structure

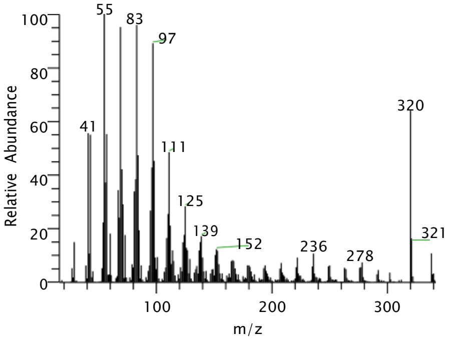

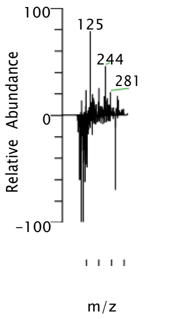

Erucic acid
Formula C22H42O2, MW 338, CAS# 112-86-7, Entry# 4603
13-Docosenoic acid, (Z)-

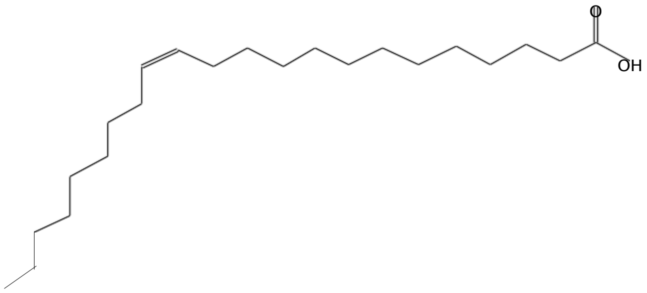

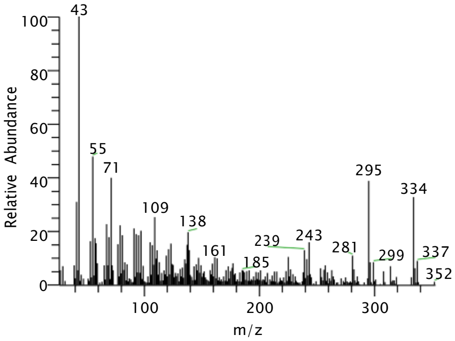

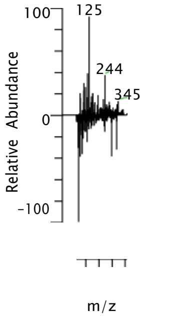

6á-Hydroxyfluoxymesterone
Formula C20H29FO4, MW 352, CAS# 88936-08-7, Entry# 6598
9-Fluoro-6,11,17-trihydroxy-17-methylandrost-4-en-3-one #

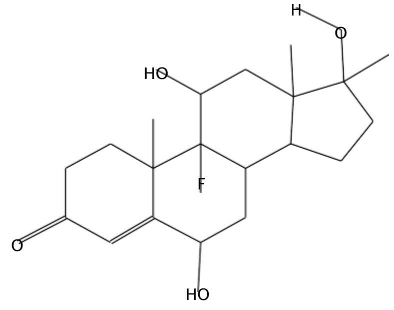

My Qual X-Report

Hit Spectrum | Delta | Compound Structure

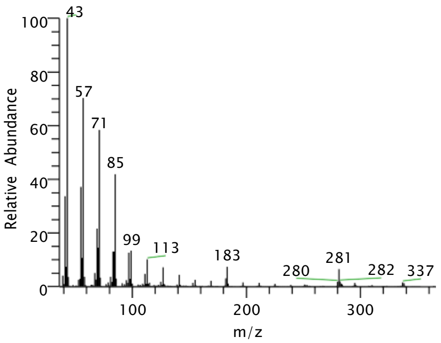

Raw data - Library entry

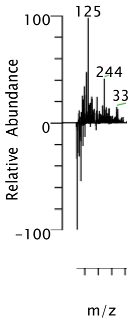

Octadecane, 3-ethyl-5-(2-ethylbutyl)-
Formula C26H54, MW 366, CAS# 55282-12-7, Entry# 2126
3-Ethyl-5-(2'-ethylbutyl)octadecane

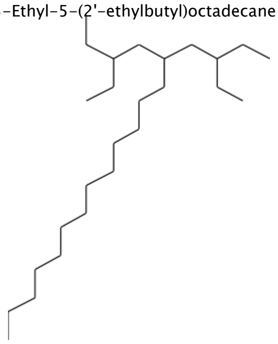

RT: 21.38 - 22.57 SM: 15G

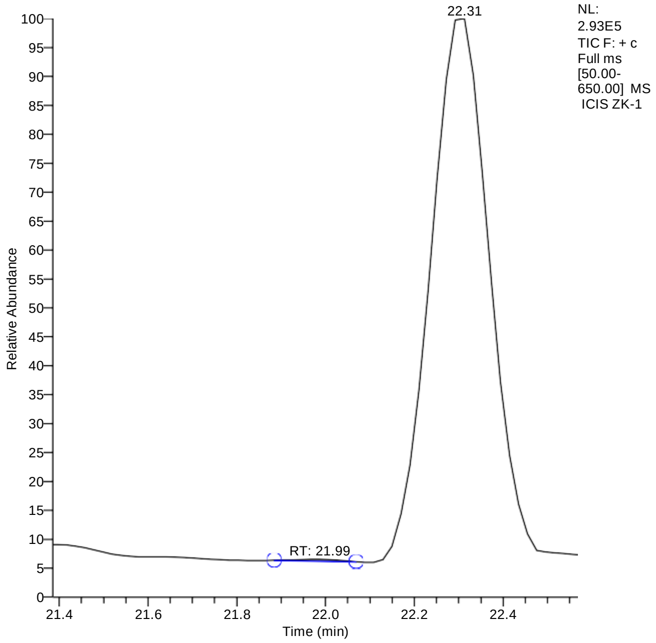

ZK-1 #933 RT: 21.99 AV: 1 AV: 5 SB: 12 926-931 935-940 NL: 6.80E2
F: + c Full ms [50.00-650.00]

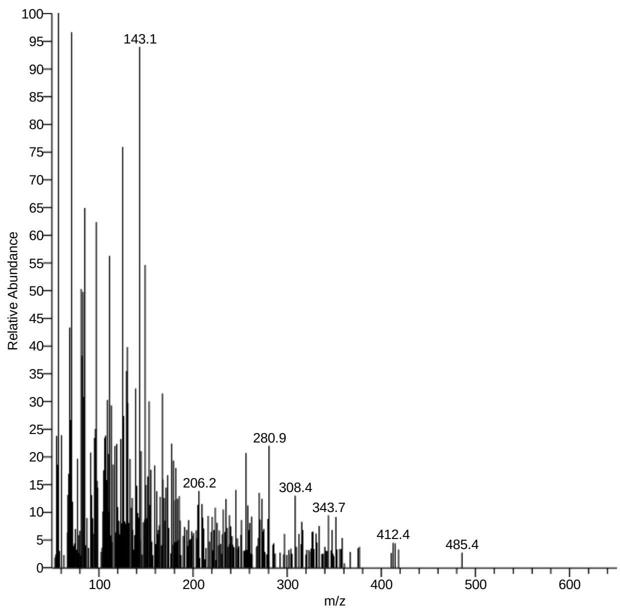

SI	RSI	Compound Name	Library	Probability	Area %	Area	RT
605	667	18,19-Secoyohimban-19-oic acid, 16,17,20,21-tetradehydro-16-(hydroxy methyl)-, methyl ester, (15á,16E)-	MAINLIB	18.38	0.14	7338.81	21.99
599	649	8H-Azecino[5,4-b]indol-8-one, 5-ethylidene-1,2,3,4,5,6,7,9-octahydro-6 -(2-hydroxyethyl)-3-methyl-, [S-(E)]-	MAINLIB	14.45	0.14	7338.81	21.99
596	606	5H-Cyclopropa[3,4]benz[1,2-e]azulen-5-one, 9,9a-bis(acetyloxy)-1,1a,1b,2,4a,7a,7b,8 ,9,9a-decahydro-2,4a,7b-trihydroxy-3-(hydroxymethyl)-1,1,6,8-tetramethyl-, [1aR-(1aà,1bá,2á,4aá,7aà,7bà,8à,9á,9aà)]-	MAINLIB	12.76	0.14	7338.81	21.99

My Qual X-Report

Hit Spectrum | Delta | Compound Structure

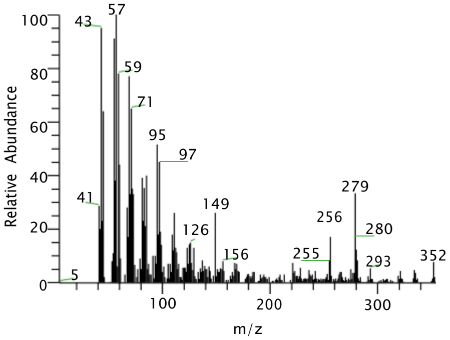

Raw data - Library entry

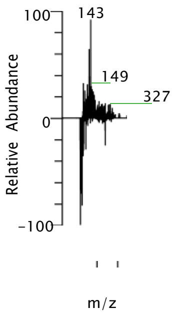

Formula C21H24N2O3, MW 352, CAS# 5523-49-9, Entry# 20709

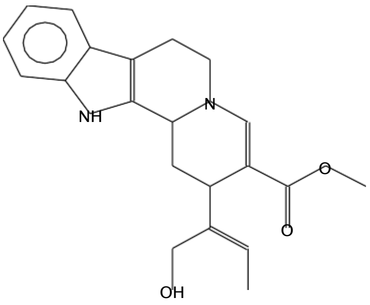

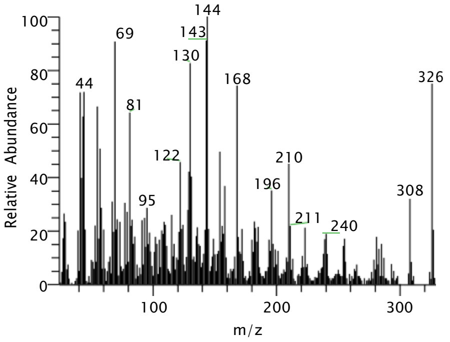

Raw data - Library entry

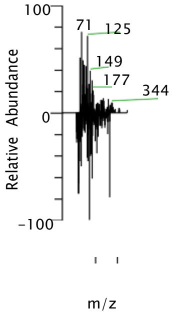

Formula C20H26N2O2, MW 326, CAS# 2134-96-5, Entry# 92362
Burnamicine

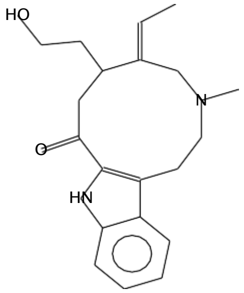

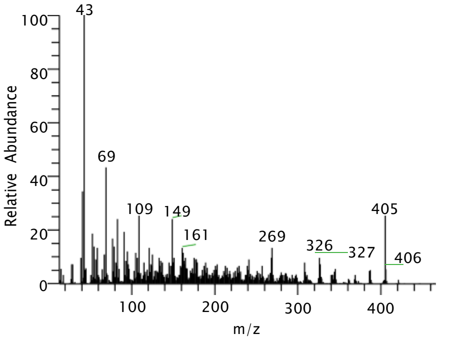

Raw data - Library entry

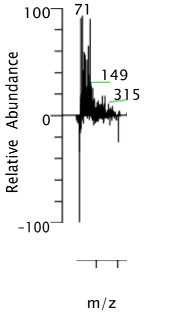

Formula C24H32O9, MW 464, CAS# 77573-19-4, Entry# 7601

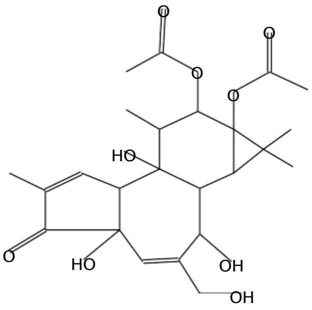

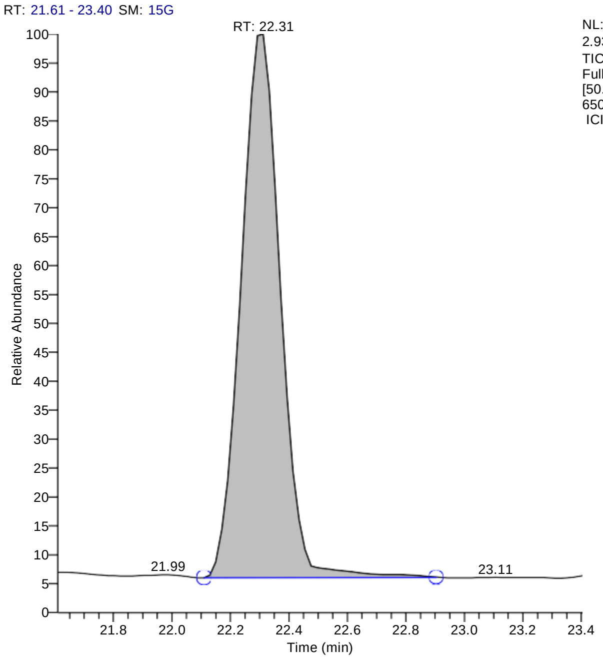

NL:
2.93E5
TIC F: + c
Full ms
[50.00-
650.00] MS
ICIS ZK-1

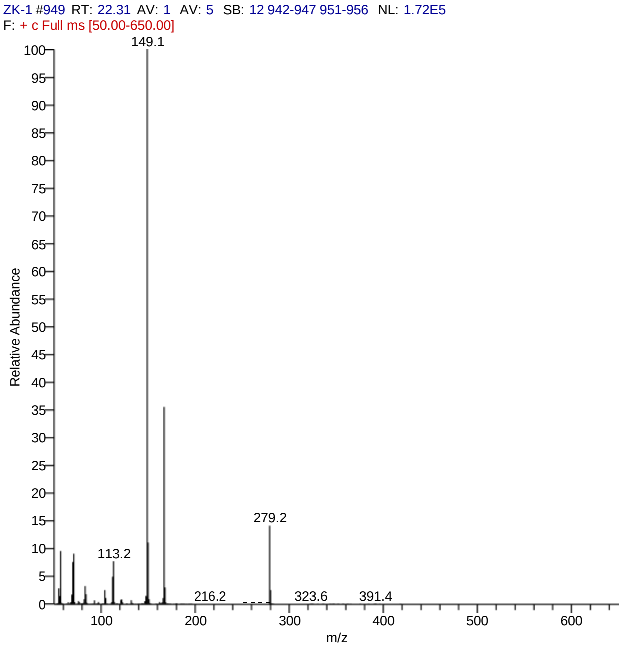

My Qual X-Report

SI	RSI	Compound Name	Library	Probability	Area %	Area	RT
878	887	1,2-Benzenedicarboxylic acid, mono(2-ethylhexyl) ester	MAINLIB	31.83	48.18	2602412.09	22.31
874	883	Bis(2-ethylhexyl) phthalate	replib	26.89	48.18	2602412.09	22.31
872	887	1,2-Benzenedicarboxylic acid, diisooctyl ester	replib	24.80	48.18	2602412.09	22.31

Hit Spectrum | Delta | Compound Structure

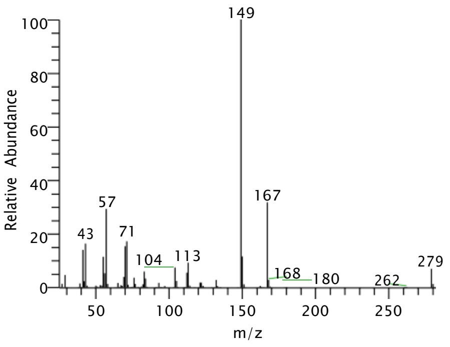

Raw data - Library entry

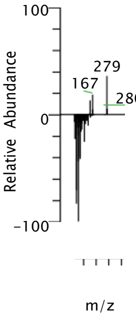

1,2-Benzenedicarboxylic acid, mono(2-ethylhexyl) ester
Formula C16H22O4, MW 278, CAS# 4376-20-9, Entry# 96269
Mono(2-ethylhexyl) phthalate

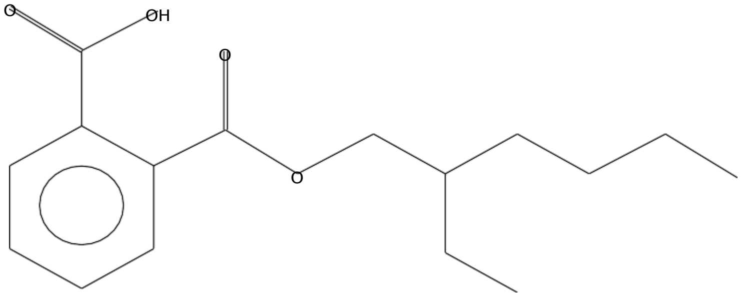

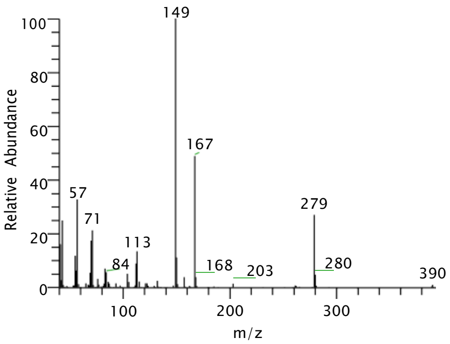

Raw data - Library entry

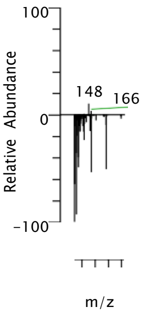

Bis(2-ethylhexyl) phthalate
Formula C24H38O4, MW 390, CAS# 117-81-7, Entry# 19802
1,2-Benzenedicarboxylic acid, bis(2-ethylhexyl) ester

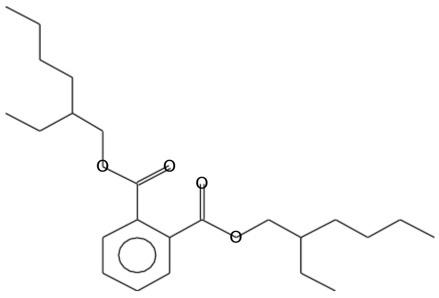

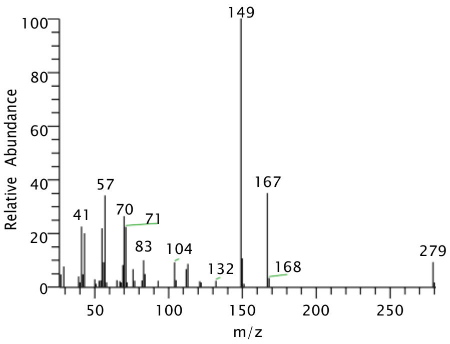

Raw data - Library entry

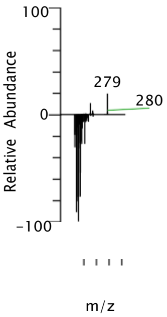

1,2-Benzenedicarboxylic acid, diisooctyl ester
Formula C24H38O4, MW 390, CAS# 27554-26-3, Entry# 19804
Diisooctyl phthalate

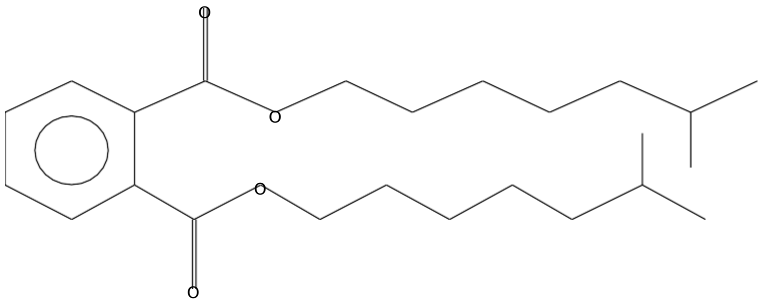

My Qual X-Report

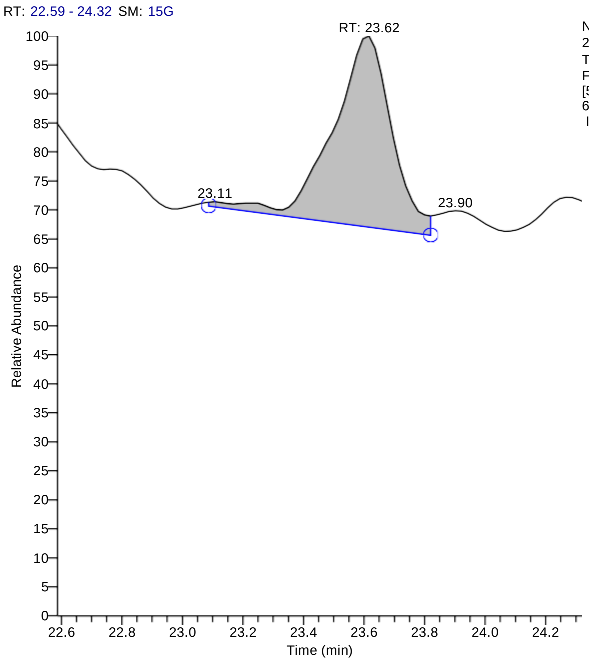

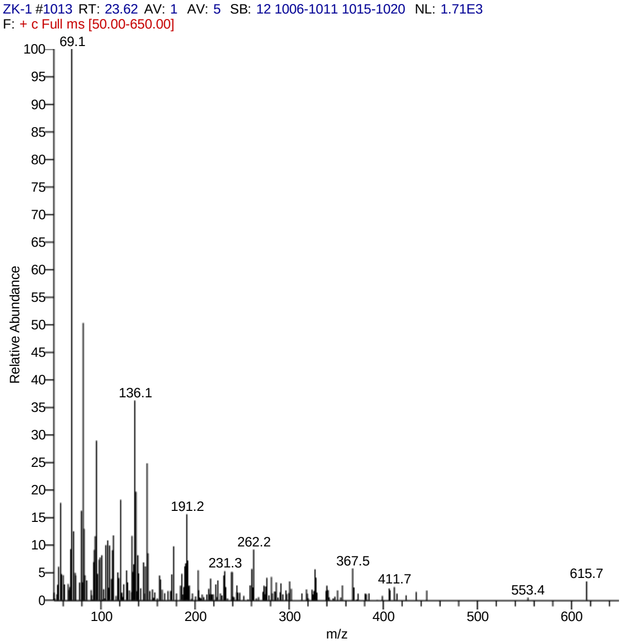

SI	RSI	Compound Name	Library	Probability	Area %	Area	RT
565	607	2,2,4-Trimethyl-3-(3,8,12,16-tetramethyl-heptadeca-3,7,11,15-tetraenyl)-cyclohexanol	MAINLIB	20.13	2.08	112446.19	23.62
560	659	Squalene	MAINLIB	16.22	2.08	112446.19	23.62
548	707	2,6,10,14,18,22-Tetracosahexaene, 2,6,10,15,19,23-hexamethyl-, (all-E)-	replib	10.80	2.08	112446.19	23.62

Hit Spectrum | Delta | Compound Structure

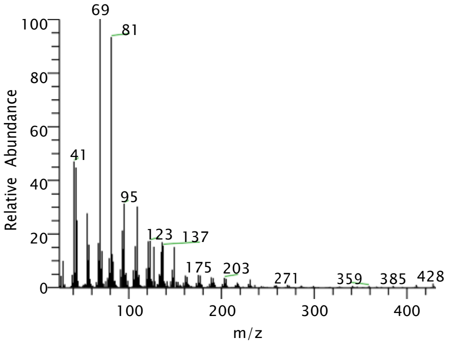

Raw data - Library entry

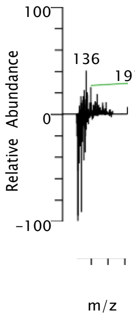

2,2,4-Trimethyl-3-(3,8,12,16-tetramethyl-heptadeca-3,7,11,15-tetraenyl)-cyclohexanol
Formula C30H52O, MW 428, CAS# NA, Entry# 28969

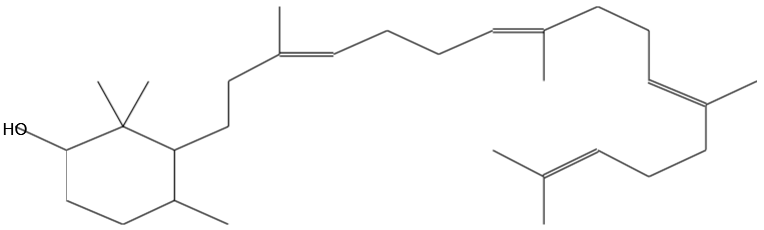

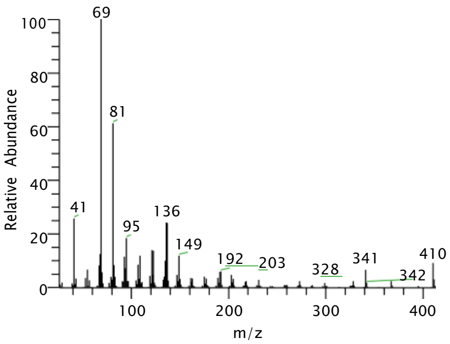

Raw data - Library entry

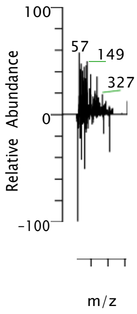

Squalene
Formula C30H50, MW 410, CAS# 7683-64-9, Entry# 28976
2,6,10,14,18,22-Tetracosahexaene, 2,6,10,15,19,23-hexamethyl-

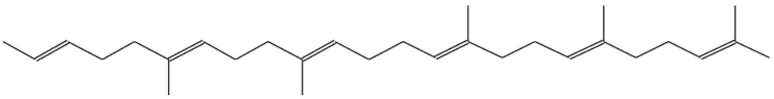

My Qual X-Report

Hit Spectrum	Delta	Compound Structure
	Raw data - Library entry	2,6,10,14,18,22-Tetracosahexaene, 2,6,10,15,19,23-hexamethyl-, (all-E)-<br>Formula C30H50, MW 410, CAS# 111-02-4, Entry# 7716<br>All-trans-Squalene

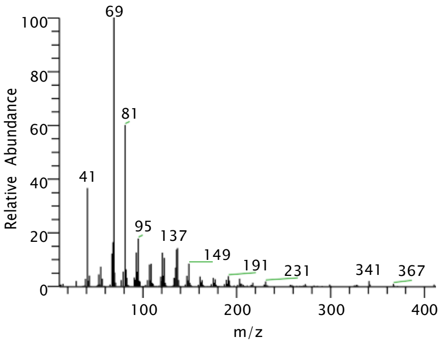

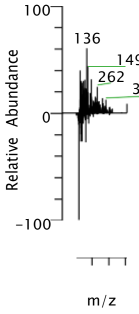

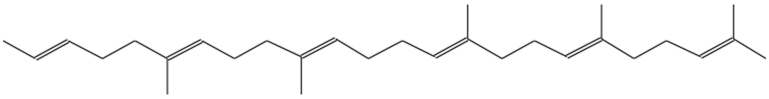

RT: 23.32 - 24.56 SM: 15G

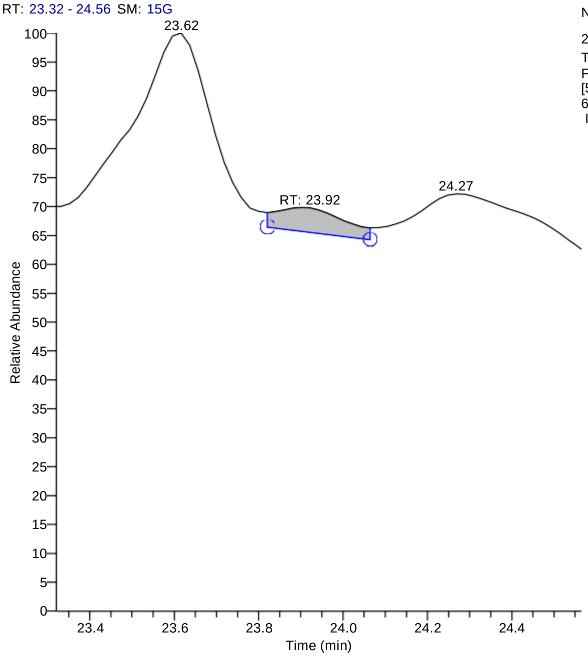

NL:
2.50E4
TIC F: + c
Full ms
[50.00-
650.00] MS
ICIS ZK-1

ZK-1 #1028 RT: 23.92 AV: 1 AV: 5 SB: 12 1021-1026 1030-1035 NL: 5.19E2
F: + c Full ms [50.00-650.00]

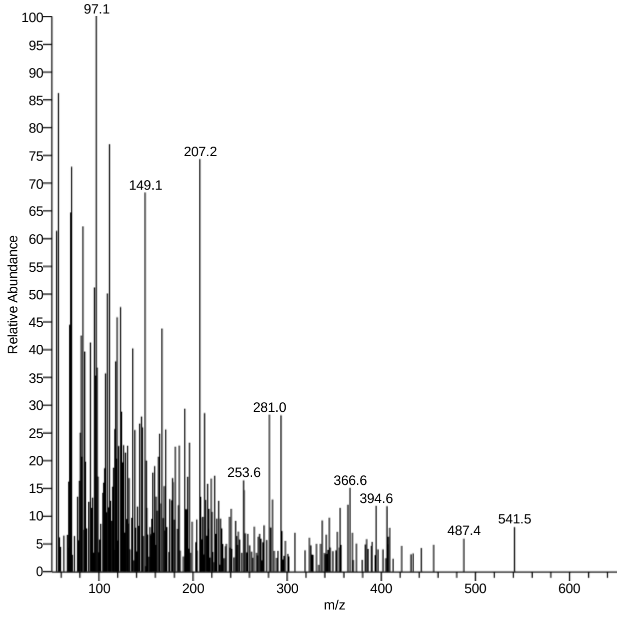

SI	RSI	Compound Name	Library	Probability	Area %	Area	RT
576	621	9-Octadecenoic acid, (2-phenyl-1,3-dioxolan-4-yl)methyl ester, cis-	replib	17.02	0.22	12018.57	23.92
566	640	9,12,15-Octadecatrienoic acid, 2,3-bis[(trimethylsilyl)oxy]propyl ester, (Z,Z,Z)-	replib	12.01	0.22	12018.57	23.92
555	662	9,10-Secocholesta-5,7,10(19)-triene-3,24,25-triol, (3á,5Z,7E)-	MAINLIB	8.24	0.22	12018.57	23.92

Hit Spectrum	Delta	Compound Structure
	Raw data - Library entry	9-Octadecenoic acid, (2-phenyl-1,3-dioxolan-4-yl)methyl ester, cis-<br>Formula C28H44O4, MW 444, CAS# 56599-45-2, Entry# 1934<br>(2-Phenyl-1,3-dioxolan-4-yl)methyl (9E)-9-octadecenoate #

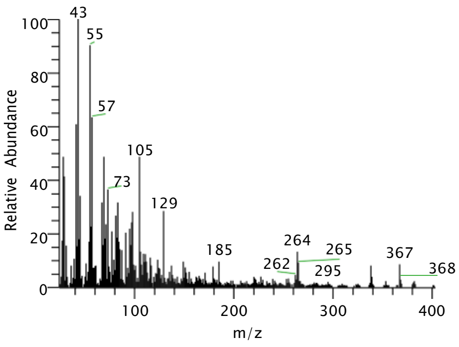

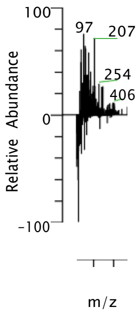

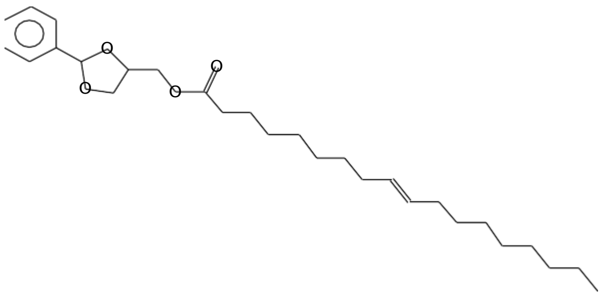

My Qual X-Report

Hit Spectrum | Delta | Compound Structure

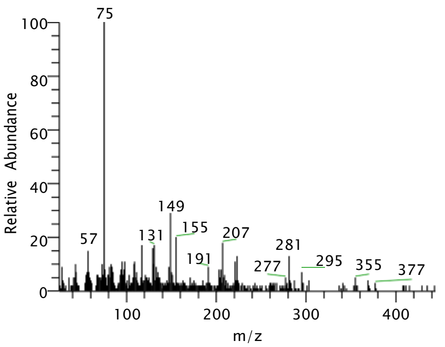

Raw data - Library entry

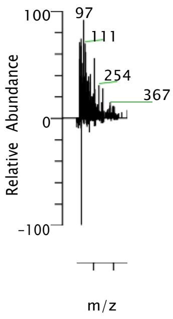

9,12,15-Octadecatrienoic acid, 2,3-bis[(trimethylsilyl)oxy]propyl ester, (Z,Z,Z)-
Formula C27H52O4Si2, MW 496, CAS# 55521-22-7, Entry# 9235
2,3-Bis[(trimethylsilyl)oxy]propyl (9E,12E,15E)-9,12,15-octadecatrienoate #

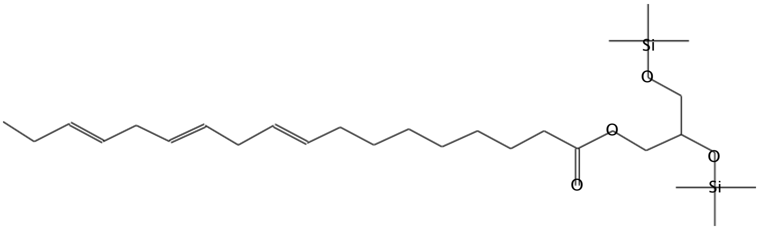

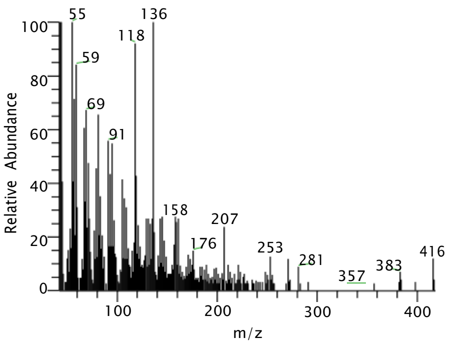

Raw data - Library entry

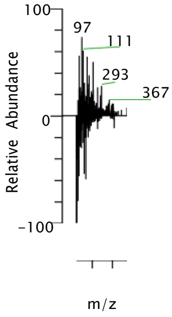

9,10-Secocholesta-5,7,10(19)-triene-3,24,25-triol, (3á,5Z,7E)-
Formula C27H44O3, MW 416, CAS# 40013-87-4, Entry# 6060
24,25-Dihydroxycholecalciferol

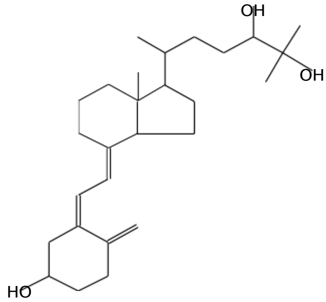

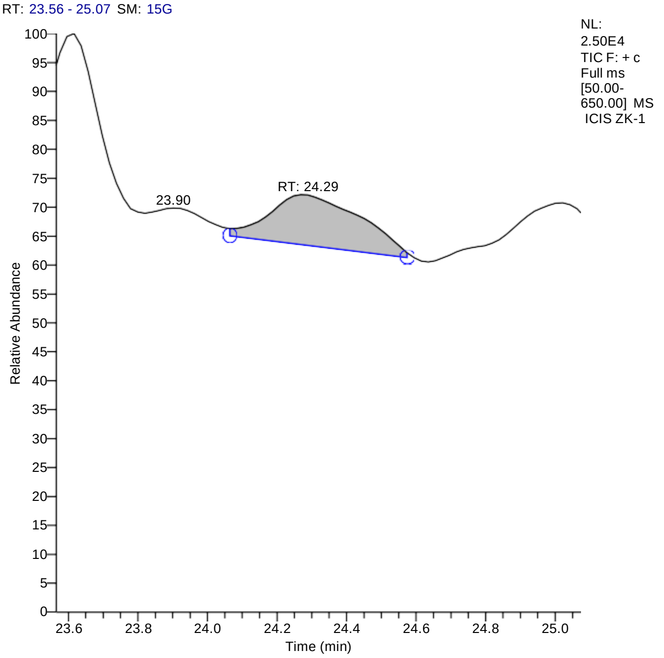

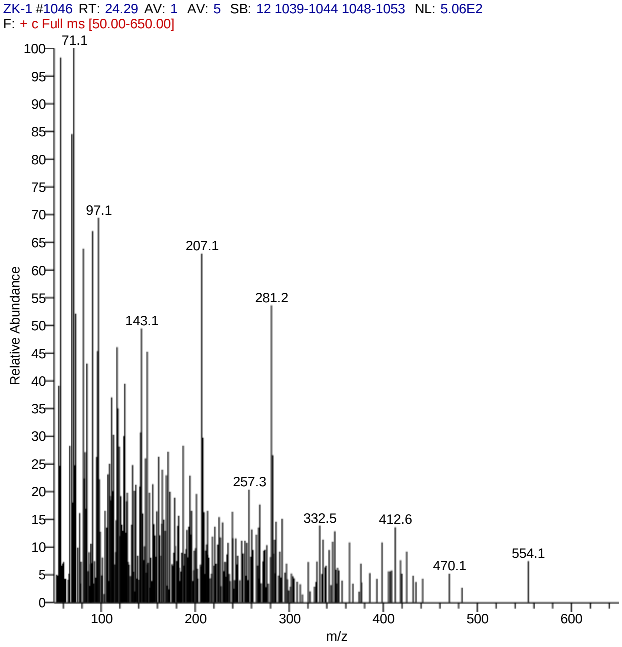

SI	RSI	Compound Name	Library	Probability	Area %	Area	RT
588	667	Corynan-17-ol, 18,19-didehydro-10-methoxy-, acetate (ester)	MAINLIB	29.58	0.76	41095.49	24.29
580	672	Dasycarpidan-1-methanol, acetate (ester)	MAINLIB	22.06	0.76	41095.49	24.29
559	581	17-Pentatriacontene	MAINLIB	9.38	0.76	41095.49	24.29

My Qual X-Report

Hit Spectrum | Delta | Compound Structure

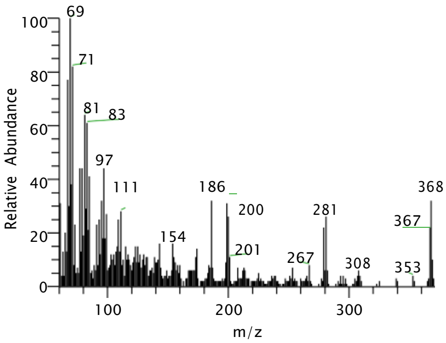

Raw data - Library entry

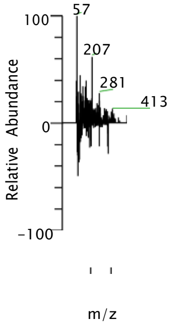

Corynan-17-ol, 18,19-didehydro-10-methoxy-, acetate (ester)
Formula C22H28N2O3, MW 368, CAS# 56053-13-5, Entry# 28918
10-Methoxycoryn-18-en-17-yl acetate #

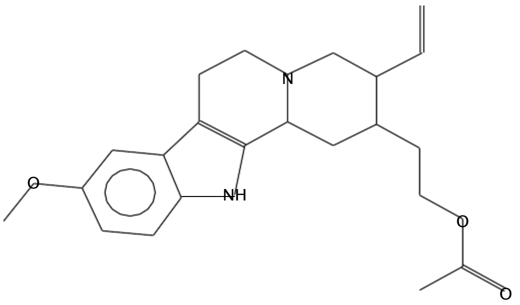

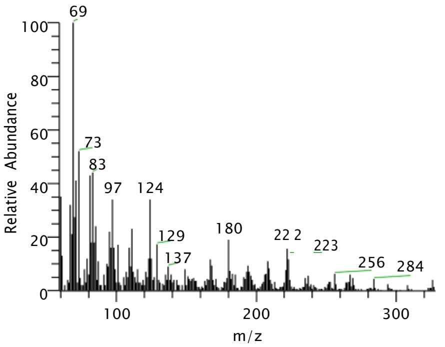

Raw data - Library entry

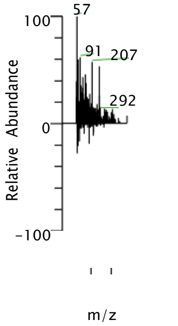

Dasycarpidan-1-methanol, acetate (ester)
Formula C20H26N2O2, MW 326, CAS# 55724-48-6, Entry# 28935

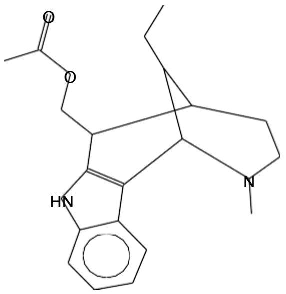

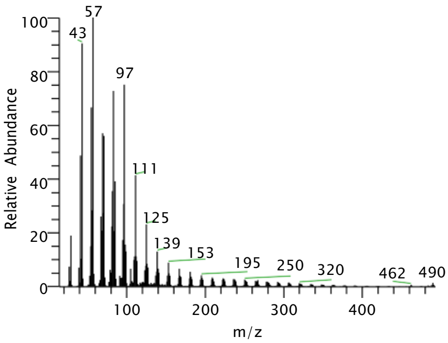

Raw data - Library entry

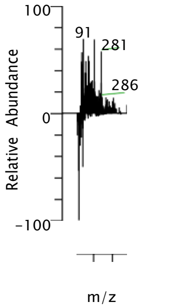

17-Pentatriacontene
Formula C35H70, MW 490, CAS# 6971-40-0, Entry# 21018
(17E)-17-Pentatriacontene #

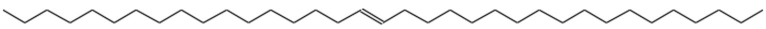

RT: 24.24 - 25.64 SM: 15G

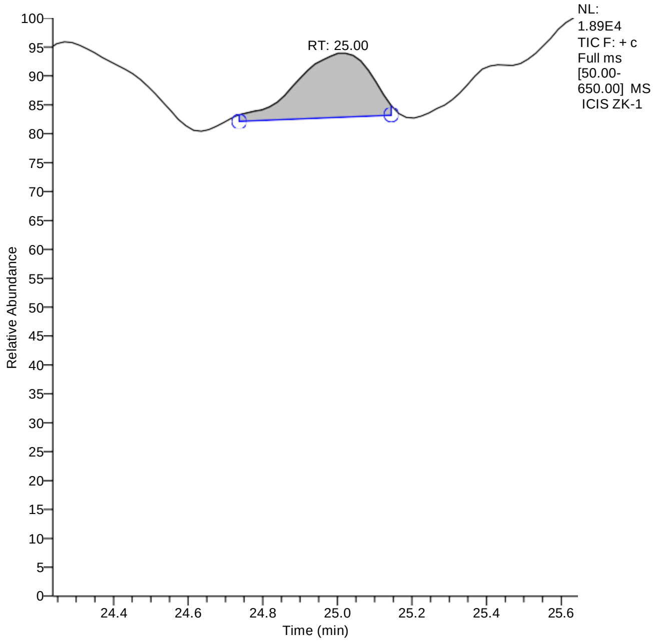

ZK-1 #1081 RT: 25.00 AV: 1 AV: 5 SB: 12 1074-1079 1083-1088 NL: 6.57E2
F: + c Full ms [50.00-650.00]

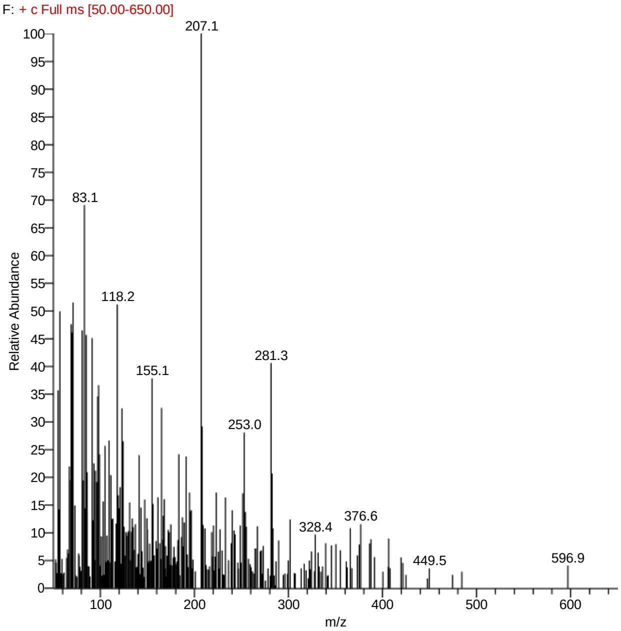

My Qual X-Report

SI	RSI	Compound Name	Library	Probability	Area %	Area	RT
567	648	9,12,15-Octadecatrienoic acid, 2,3-bis[(trimethylsilyl)oxy]propyl ester, (Z,Z,Z)-	replib	12.77	0.54	29263.31	25.00
558	625	Oleic acid, 3-(octadecyloxy)propyl ester	replib	9.27	0.54	29263.31	25.00
558	577	Docosanoic acid, 1,2,3-propanetriyl ester	MAINLIB	9.27	0.54	29263.31	25.00

Hit Spectrum | Delta | Compound Structure

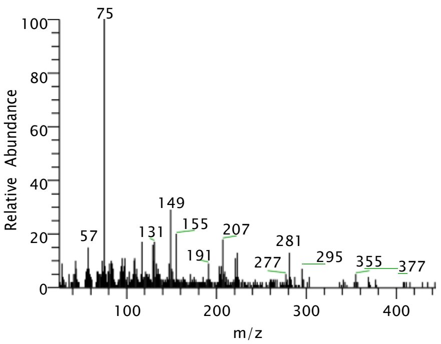

Raw data - Library entry

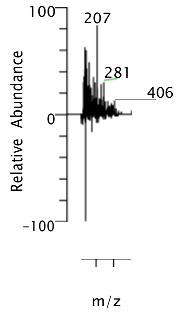

9,12,15-Octadecatrienoic acid, 2,3-bis[(trimethylsilyl)oxy]propyl ester, (Z,Z,Z)-
Formula C27H52O4Si2, MW 496, CAS# 55521-22-7, Entry# 9235
2,3-Bis[(trimethylsilyl)oxy]propyl (9E,12E,15E)-9,12,15-octadecatrienoate #

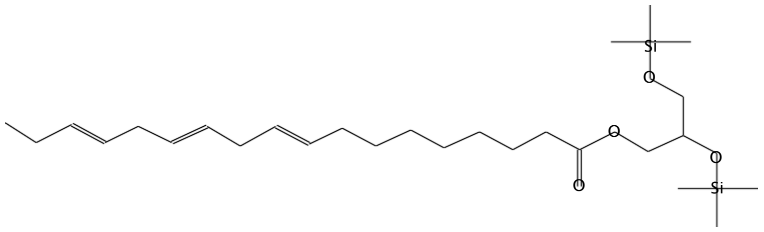

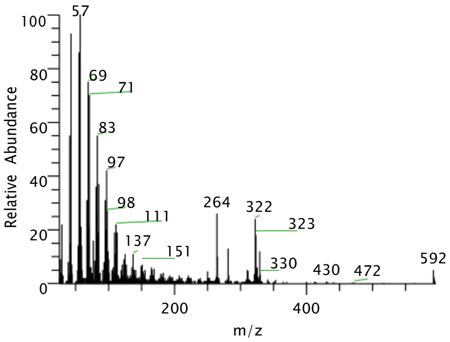

Raw data - Library entry

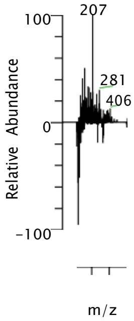

Oleic acid, 3-(octadecyloxy)propyl ester
Formula C39H76O3, MW 592, CAS# 17367-41-8, Entry# 5395
3-(Octadecyloxy)propyl (9E)-9-octadecenoate #

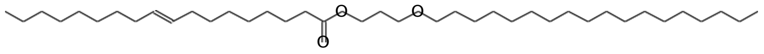

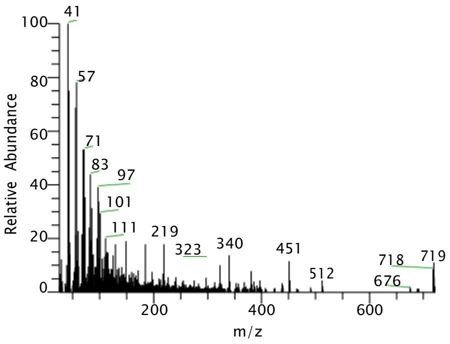

Raw data - Library entry

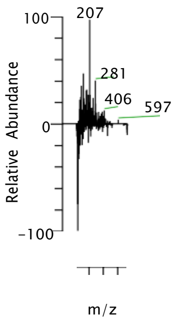

Docosanoic acid, 1,2,3-propanetriyl ester
Formula C69H134O6, MW 1058, CAS# 18641-57-1, Entry# 2725
Docosanoin, tri-

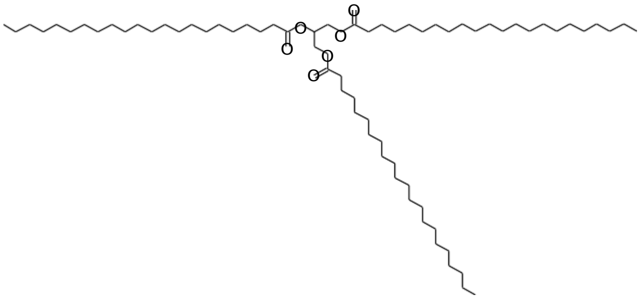

My Qual X-Report

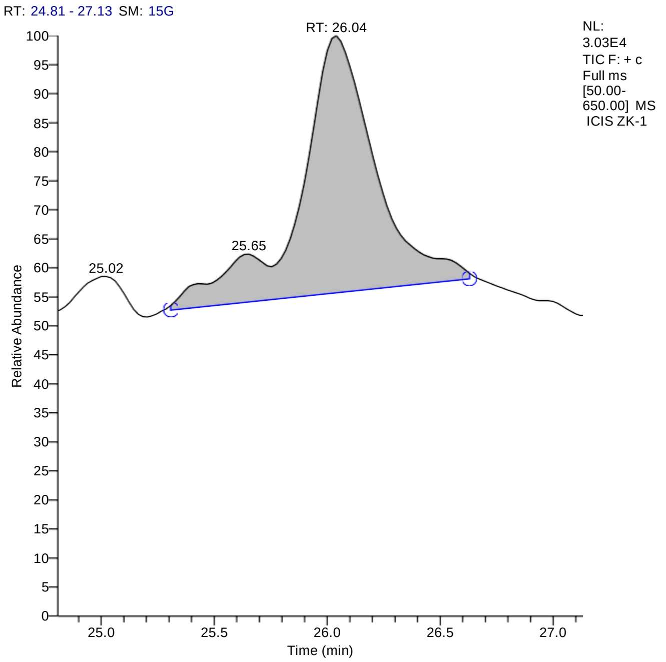

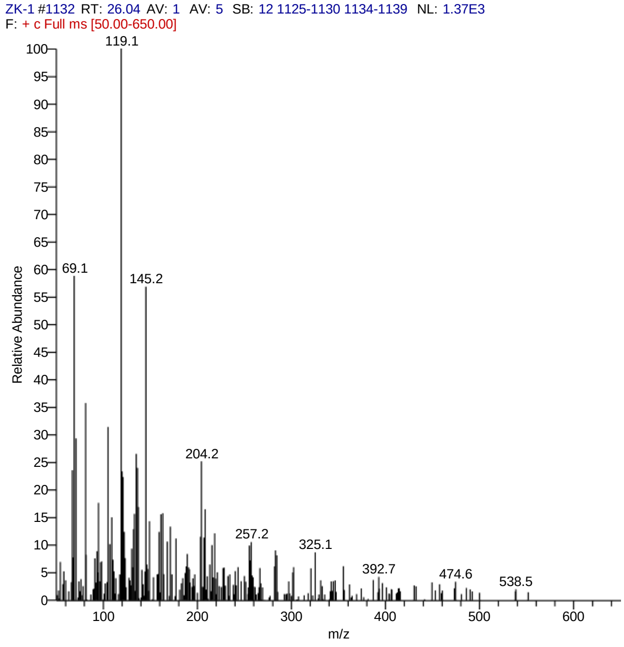

SI	RSI	Compound Name	Library	Probability	Area %	Area	RT
522	523	Rhodopin	MAINLIB	8.19	5.91	319482.82	26.04
522	543	Azafrin	MAINLIB	8.19	5.91	319482.82	26.04
511	512	.psi.,.psi.-Carotene, 1,1',2,2'-tetrahydro-1,1'-dimethoxy-	MAINLIB	5.62	5.91	319482.82	26.04

Hit Spectrum | Delta | Compound Structure

Raw data - Library entry

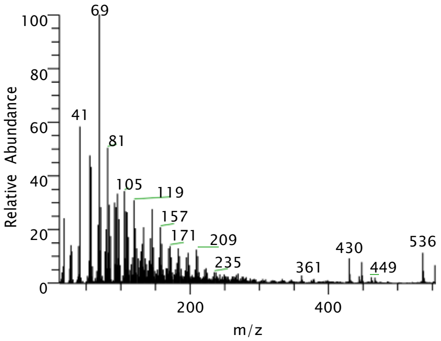

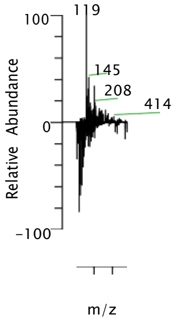

Rhodopin
Formula C40H58O, MW 554, CAS# 105-92-0, Entry# 28224
.psi.,.psi.-Carotene, 1,2-dihydro-1-hydroxy-

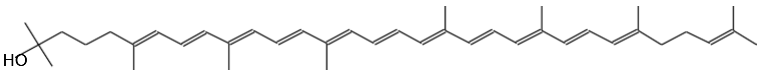

SI 522, RSI 543, MAINLIB, Entry# 65273, CAS# 507-61-9, Azafrin

Raw data - Library entry

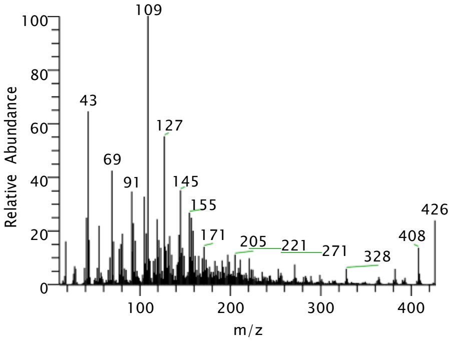

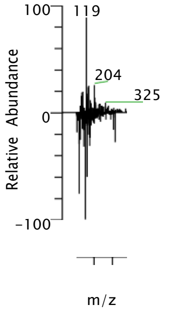

Azafrin
Formula C27H38O4, MW 426, CAS# 507-61-9, Entry# 65273
10'-Apo-á,.psi.-carotenoic acid, 5,6-dihydro-5,6-dihydroxy-, (5R,6R)-

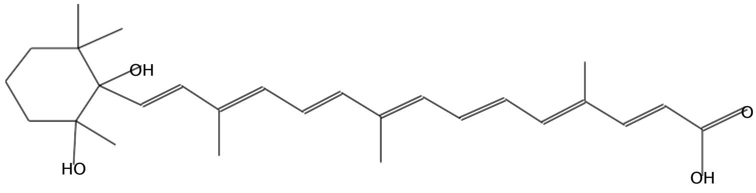

My Qual X-Report

Hit Spectrum | Delta | Compound Structure

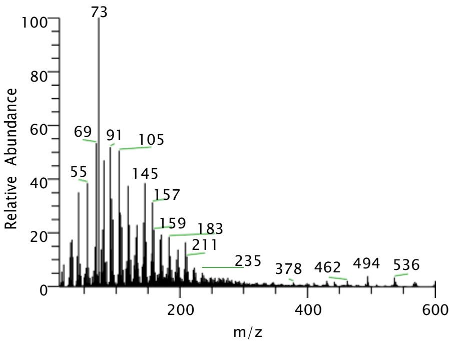

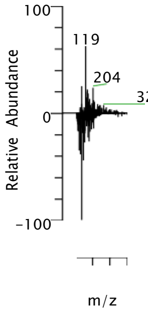

.psi.,.psi.-Carotene, 1,1',2,2'-tetrahydro-1,1'-dimethoxy-
Formula C42H64O2, MW 600, CAS# 13833-01-7, Entry# 32753
Lycopene, 1,1',2,2'-tetrahydro-1,1'-dimethoxy-, all-trans-

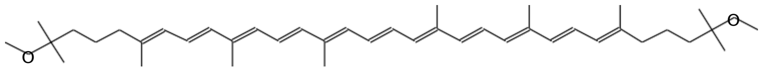

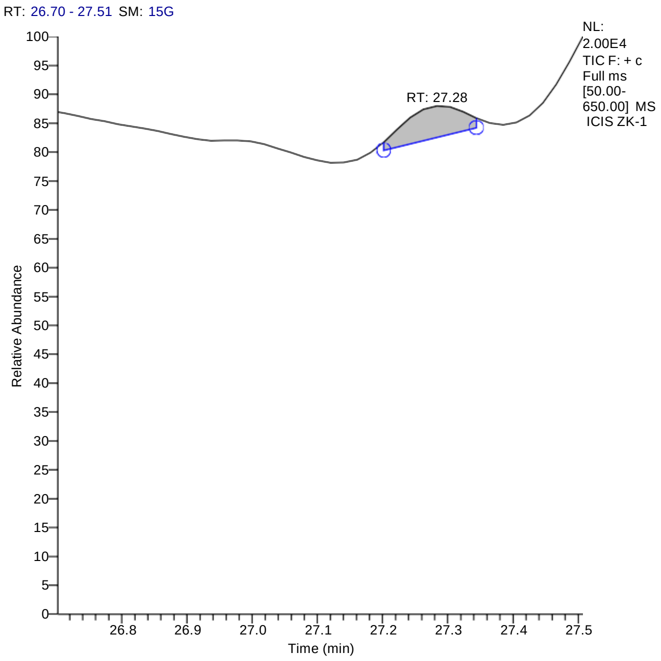

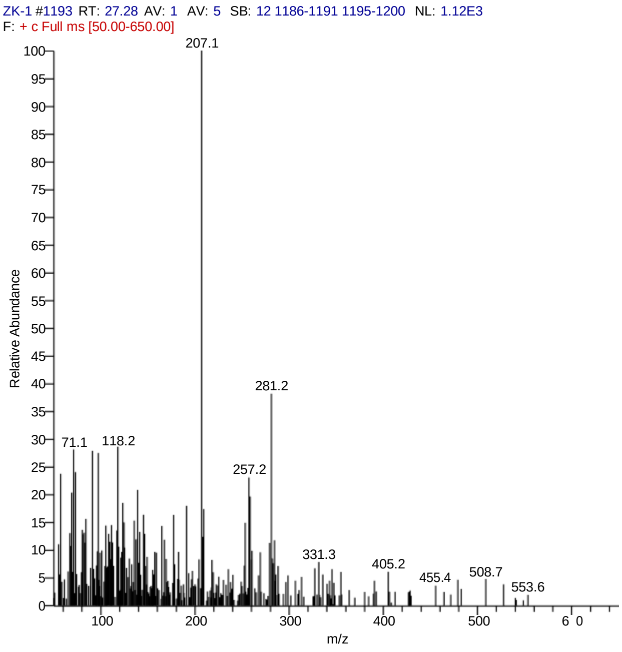

SI	RSI	Compound Name	Library	Probability	Area %	Area	RT
572	613	Butanoic acid, 1a,2,5,5a,6,9,10,10a-octahydro-5,5a-dihydroxy-4-(hydroxymethyl)-1,1,7,9-tetramethyl-11-oxo-1H-2,8a-methanocyclopenta[a]cyclopropa[e]cyclodecen-6-yl ester, [1aR-(1aà,2à,5á,5aá,6á,8aà,9à,10aà)]-	MAINLIB	26.41	0.13	6904.09	27.28
571	596	4H-Cyclopropa[5',6']benz[1',2':7,8]azuleno[5,6-b]oxiren-4-one, 8-(acetyloxy)-1,1a,1b,1c,2a,3,3a,6a,6b,7,8,8a-dodecahydro-3a,6b,8a-trihydroxy-2a-(hydroxymethyl)-1,1,5,7-tetramethyl-, (1aà,1bá,1cá,2aá,3aá,6aà,6bà,7à,8á,8aà)-	MAINLIB	25.38	0.13	6904.09	27.28
539	629	9,12,15-Octadecatrienoic acid, 2,3-bis[(trimethylsilyl)oxy]propyl ester, (Z,Z,Z)-	replib	6.78	0.13	6904.09	27.28

My Qual X-Report

Hit Spectrum | Delta | Compound Structure

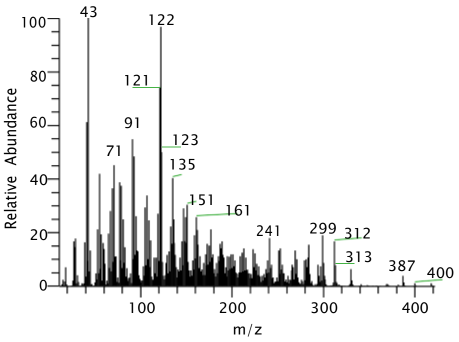

Raw data - Library entry

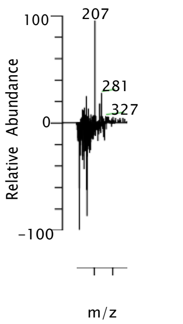

Formula C24H34O6, MW 418, CAS# 77508-67-9, Entry# 10305

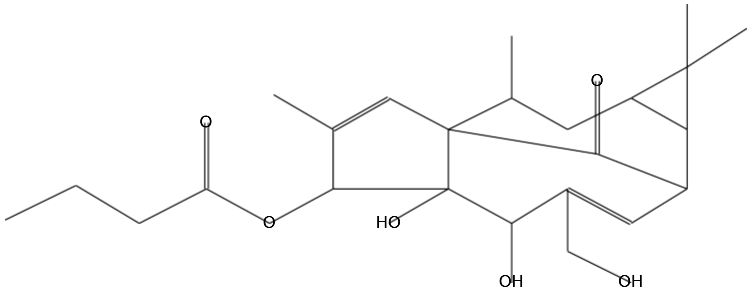

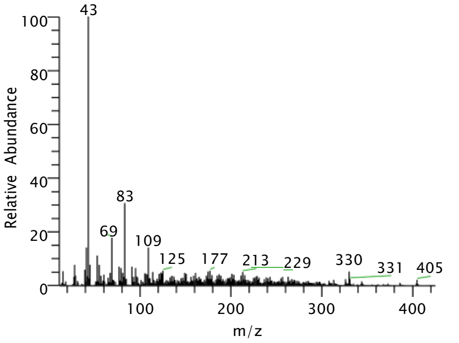

Raw data - Library entry

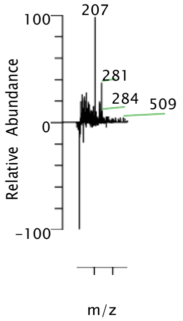

Formula C22H30O8, MW 422, CAS# 77646-23-2, Entry# 8594

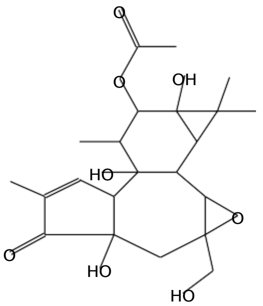

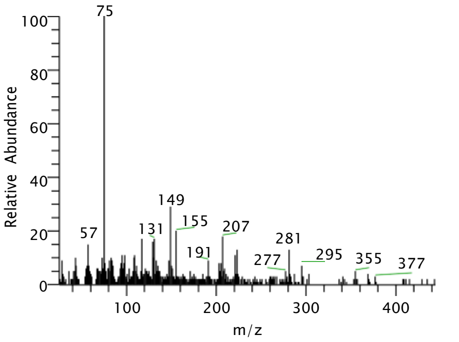

Raw data - Library entry

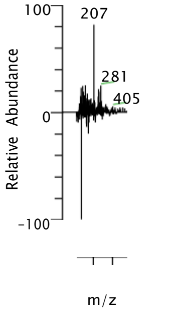

9,12,15-Octadecatrienoic acid, 2,3-bis[(trimethylsilyl)oxy]propyl ester, (Z,Z,Z)-
Formula C27H52O4Si2, MW 496, CAS# 55521-22-7, Entry# 9235
2,3-Bis[(trimethylsilyl)oxy]propyl (9E,12E,15E)-9,12,15-octadecatrienoate #

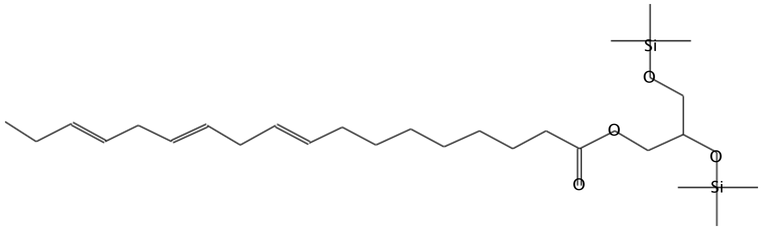

GCMS analysis of n-hexane fraction of *B. indica*

```
Information from Data File:
File        : C:\MSDCHEM\1\DATA\20201230.B\S.D
Operator    : Dr. Barkat Ali
Acquired    : 19 Jan 2022  15:19     using AcqMethod PLANTS 2
Sample Name: S
Misc Info   :
Vial Number: 1

Search Libraries:   C:\NIST11.L                         Minimum Quality:   0

Unknown Spectrum:   Apex
Integration Params: AutoIntegrate
```

Pk#	RT	Area%	Library/ID	Ref#	CAS#	Qual
1	4.25	0.54	C:\NIST11.L			
			2-Furancarboxaldehyde, 5-methyl-	5629	000620-02-0	81
			2-Furancarboxaldehyde, 5-methyl-	5630	000620-02-0	72
			2-Furancarboxaldehyde, 5-methyl-	5631	000620-02-0	64
2	4.37	0.24	C:\NIST11.L			
			2-Furancarboxaldehyde, 5-methyl-	5629	000620-02-0	45
			2-Furancarboxaldehyde, 5-methyl-	5631	000620-02-0	43
			2-Furancarboxaldehyde, 5-methyl-	5630	000620-02-0	43
3	5.53	0.27	C:\NIST11.L			
			2-Cyclopenten-1-one, 2,3,4-trim...	10233	028790-86-5	38
			Phenol, 2-methoxy-	10078	000090-05-1	38
			Phenol, 2-methoxy-	10079	000090-05-1	38
4	5.91	7.72	C:\NIST11.L			
			Propanenitrile, 3-(dimethylamino)-	3011	001738-25-6	58
			Propanenitrile, 3-(dimethylamino)-	3008	001738-25-6	58
			2-Methoxydiethylamine	4496	034322-82-2	50
5	6.20	3.54	C:\NIST11.L			
			Silacyclopentane	1611	000288-06-2	58
			2-Propanone, 1-(dimethylamino)-	4034	015364-56-4	53
			2-Propanone, 1-(dimethylamino)-	4039	015364-56-4	53
6	6.37	0.84	C:\NIST11.L			
			N,N-Dimethylglycine	4441	001118-68-9	53
			2-Methoxydiethylamine	4496	034322-82-2	53
			Silacyclopentane	1611	000288-06-2	52
7	6.48	0.73	C:\NIST11.L			
			N,N-Dimethylglycine	4441	001118-68-9	64
			2-Methoxydiethylamine	4496	034322-82-2	59
			N,N-Dimethyl-2-methoxyethylamine	4510	003030-44-2	59
8	7.51	0.36	C:\NIST11.L			
			N,N-Dimethylglycine	4441	001118-68-9	53
			Benzeneethanamine, N,.alpha.-di...	22371	033817-09-3	53

			Benzeneethanamine, N,.alpha.-di...	22366	007632-10-2	53
9	7.75	0.47	C:\NIST11.L			
			Cyclopentanemethylamine, 2-isop...	43398	017943-85-0	59
			Cyclopentanemethylamine, 2-isop...	43397	017943-83-8	59
			Benzeneethanamine, N,.alpha.-di...	22371	033817-09-3	53
10	8.13	16.94	C:\NIST11.L			
			2-Methoxy-4-vinylphenol	23329	007786-61-0	87
			Ethanone, 1-(3-methoxyphenyl)-	23390	000586-37-8	64
			Ethanone, 1-(3-methoxyphenyl)-	23391	000586-37-8	64
11	8.36	1.87	C:\NIST11.L			
			Methamphetamine	22301	000537-46-2	53
			N,N-Dimethylglycine	4441	001118-68-9	50
			O-(2-(Dimethylamino)ethyl)-2-ch...	135379	126551-78-8	45
12	8.57	4.88	C:\NIST11.L			
			9-Azabicyclo[6.1.0]non-4-en-9-a...	16830	066387-78-8	43
			1-Ethyl-3-(3-dimethylaminopropy...	26834	019785-93-4	38
			26-Deoxy-26-ethylaminodihydrone...	163842	1000256-67-5	35
13	8.64	2.32	C:\NIST11.L			
			Cyclopentaneethanamine, N,.alph...	18409	000102-45-4	52
			Benzedrex	26564	000101-40-6	47
			N-Acetyl-3,4-methylenedioxymeth...	79092	181765-92-4	47
14	9.02	1.21	C:\NIST11.L			
			1-Cyclopropyl-1-methyl-ethylamine	3491	172947-13-6	64
			2-Methoxydiethylamine	4496	034322-82-2	38
			Dimethylamine, N-(diisopropylph...	39583	062474-28-6	35
15	9.08	0.59	C:\NIST11.L			
			2-Nonanone, 9-methoxy	37205	1000130-73-3	43
			Cyclopentaneethanamine, N,.alph...	18409	000102-45-4	43
			1-Octadecanamine, N,N-dimethyl-	115994	000124-28-7	38
16	9.12	1.19	C:\NIST11.L			
			9-Azabicyclo[6.1.0]non-4-en-9-a...	16830	066387-78-8	38
			Cyclopentaneethanamine, N,.alph...	18409	000102-45-4	35
			2-Methoxydiethylamine	4496	034322-82-2	35
17	9.27	1.60	C:\NIST11.L			
			Benzene, 2-(1,3-butadienyl)-1,3...	37591	005732-00-3	46
			Quinoline, 3-ethyl-	27839	001873-54-7	43
			Naphthalene, 1,2-dihydro-2,5,8-...	37590	030316-23-5	38
18	9.55	0.95	C:\NIST11.L			
			6,8-Nonadien-2-one, 6-methyl-5-...	50509	060714-16-1	55
			2-Butanone, 4-(2,6,6-trimethyl-...	50523	020483-36-7	51
			Benzene, 1,4-dimethyl-2-(2-meth...	30715	055669-88-0	30
19	9.65	0.72	C:\NIST11.L			
			Benzene, (1,1-dimethylpropyl)-	21829	002049-95-8	35

			Benzene, (chloromethyl)ethyl-	26341	026968-58-1	35
			Carbamic acid, (1,3-dithian-2-y...	60984	139540-22-0	35
20	9.71	0.70	C:\NIST11.L			
			Benzenemethanamine, N,N-dimethyl-	15101	000103-83-3	49
			N-Acetyl-D-phenylalanine	60702	010172-89-1	35
			2-Methoxydiethylamine	4496	034322-82-2	30
21	9.93	0.45	C:\NIST11.L			
			N,N-Dimethylglycine	4442	001118-68-9	35
			Formamide, N-[3-(dimethylamino)...	12911	005922-69-0	35
			2-Methoxy-4-methyl-3,4-dihydro-...	11927	038113-08-5	35
22	9.98	3.12	C:\NIST11.L			
			2-Hydroxy-5-methylbenzaldehyde	15760	000613-84-3	25
			7H-1-Benzopyran-7-one, 5-methox...	97997	000643-56-1	22
			1,3,2-Benzodioxaborole, 2-hydroxy-	15551	045770-13-6	11
23	10.16	2.59	C:\NIST11.L			
			4-(2,6,6-Trimethylcyclohexa-1,3...	49263	001203-08-3	89
			Benzene, 1-ethyl-3,5-diisopropyl-	49300	015181-13-2	64
			Benzene, 1,4-dimethyl-2,5-bis(1...	49323	010375-96-9	52
24	10.55	0.43	C:\NIST11.L			
			1H-Imidazole-4-ethanamine, N,N,...	25310	045967-45-1	35
			Cyclopentanemethylamine, 2-isop...	43398	017943-85-0	35
			Benzeneethanamine, 4-chloro-.al...	44695	000461-78-9	27
25	10.76	0.40	C:\NIST11.L			
			Benzeneacetic acid, 4-hydroxy-3...	44579	000306-08-1	50
			2-Propanone, 1-(4-hydroxy-3-met...	42289	002503-46-0	45
			Homovanillyl alcohol	35221	002380-78-1	43
26	10.83	0.51	C:\NIST11.L			
			1H-Imidazole-4-ethanamine, N,N,...	25310	045967-45-1	46
			2-Methoxydiethylamine	4496	034322-82-2	46
			Benzeneethanamine, 2-methoxy-N,...	41943	000093-30-1	38
27	11.11	1.61	C:\NIST11.L			
			Benzaldehyde, m-nitro-, O-methy...	43117	033499-33-1	43
			4-Methyl-2,5-dimethoxybenzaldehyde	42242	004925-88-6	40
			2,4,7(1H,3H,8H)-Pteridinetrione	42954	002577-38-0	38
28	11.16	0.51	C:\NIST11.L			
			Dimethylamine, N-(diisopropylph...	39583	062474-28-6	30
			Dimethylamine, N-(diisopropylph...	39584	062474-28-6	30
			Benzeneethanamine, 2-methoxy-N,...	41943	000093-30-1	27
29	11.31	1.16	C:\NIST11.L			
			Megastigmatrienone	49213	038818-55-2	99
			Megastigmatrienone	49214	038818-55-2	97
			Megastigmatrienone	49215	038818-55-2	46
30	11.52	0.38	C:\NIST11.L			

```
                N-[2-(3,5-Dimethoxy-phenyl)-2-m... 105646 1000189-96-8 22
                N,N'-Diethyl-1,6-hexanediamine      37379 013093-05-5 18
                5-Hepten-2-amine, N,6-dimethyl-     18388 000503-01-5 18

31  11.60  1.03 C:\NIST11.L
                Bicyclo[3.3.0]octan-2-one, 6-me...  22854 1000154-23-0 55
                Bicyclo[3.3.0]octan-2-one, 7-me...  22829 1000154-23-1 50
                Bicyclo[3.1.1]hept-3-en-2-one, ...  22830 001196-01-6 46

32  11.72  1.15 C:\NIST11.L
                Thiourea, dimethylaminomethyl       14083 109858-55-1 30
                O-Ethyl phosphonodiamide, N,N'-...  61910 137796-04-4 22
                1,2-Oxathiolane, 2,2-dioxide         9362 001120-71-4 22

33  11.86  2.99 C:\NIST11.L
                Megastigmatrienone                  49213 038818-55-2 99
                Megastigmatrienone                  49214 038818-55-2 97
                Megastigmatrienone                  49215 038818-55-2 70

34  12.08  0.27 C:\NIST11.L
                2-(3-Hydroxypropylamino)pyrimidine  25127 1000255-82-5 42
                2-Cyclohexen-1-one, 4-(3-hydrox...  61523 034318-21-3 38
                2-Cyclohexen-1-one, 4-(3-hydrox...  61547 052210-15-8 38

35  12.19  1.21 C:\NIST11.L
                Silane, dimethylphenyl-             15697 000766-77-8 43
                Silane, dimethylphenyl-             15698 000766-77-8 35
                3-Methyl-trans-3a,4,7,7a-tetrah...  15284 1000145-84-3 30

36  12.23  0.65 C:\NIST11.L
                Ar-tumerone                         66965 1000292-71-0 91
                Cinnamyl tiglate                    66906 072934-01-1 30
                Cyclobutanecarboxylic acid, 3-p...  66933 1000282-61-2 25

37  12.33  0.89 C:\NIST11.L
                Piperidine, 2,2,6,6-tetramethyl-    18393 000768-66-1 30
                1,3-Propanediamine, N,N,N',N'-t...  13167 000110-95-2 27
                Imidazole-5-carboxylic amide, 4...  35008 091026-74-3 25

38  12.44  0.34 C:\NIST11.L
                Phenol, p-[3-(methylamino)propyl]-  32524 032180-92-0 52
                Benzeneacetonitrile, .alpha.-[2... 105282 000125-79-1 49
                Cyclopentanemethylamine, 2-isop...  43398 017943-85-0 47

39  12.55  0.59 C:\NIST11.L
                O-Ethyl phosphonodiamide, N,N'-...  61910 137796-04-4 53
                2,3-Benzo-11,12-cyclohexano-1,4... 146918 026030-69-3 35
                2-Propanamine, 1-(2,6-dimethylp...  41935 031828-71-4 35

40  12.64  0.45 C:\NIST11.L
                Benzenebutanoic acid, 4-hydroxy...  51664 022320-10-1 38
                1,8-Dimethyl-3,6-diazahomoadama...  51718 123366-49-4 30
                8-Hydroxy-2-octanone                19994 025368-54-1 22
```

41	12.75	2.33	C:\NIST11.L			
			L-Phenylalanine, N-acetyl-, met...	69971	003618-96-0	64
			l-Phenylalanine, N-(2-hydroxy-1...	105593	112681-36-4	50
			L-Phenylalanine, N-acetyl-, met...	69973	003618-96-0	43
42	12.90	0.47	C:\NIST11.L			
			1,2-Benzenediol, 4-[2-(dimethyl...	54021	000554-99-4	47
			N,N-Dimethyl-1,7-octadien-3-amine	25016	069196-06-1	38
			2-Cyclohexen-1-one, 3-(3-hydrox...	62847	027185-79-1	38
43	13.46	0.53	C:\NIST11.L			
			3,5-Dimethoxy-4-hydroxyphenylac...	63887	004385-56-2	46
			2-Pentanone, 1-(2,4,6-trihydrox...	62574	1000116-22-3	41
			1-Butanone, 1-(2,4,6-trihydroxy...	62582	001509-06-4	38
44	13.51	0.77	C:\NIST11.L			
			Methanamine, (diethylphosphonat...	84387	162900-51-8	38
			6-Methyl-2-tridecanone	64499	073105-73-4	38
			2-Propanamine, 1-(2,6-dimethylp...	41933	031828-71-4	27
45	13.72	0.92	C:\NIST11.L			
			5,5,8a-Trimethyl-3,5,6,7,8,8a-h...	42654	054344-82-0	43
			Guaifenesin	54397	000093-14-1	43
			4,6-Dimethyl-2-pyrimidone	10005	000108-79-2	43
46	14.03	0.87	C:\NIST11.L			
			Bicyclo[3.1.1]heptane, 2,6,6-tr...	16409	006876-13-7	50
			cis-Bicyclo[2.2.1]heptane, 2,3-...	10387	1000262-02-5	45
			Bicyclo[3.1.1]heptane, 2,6,6-tr...	16412	004795-86-2	45
47	14.12	0.47	C:\NIST11.L			
			Benzenemethanol, 4-hydroxy-.alp...	43323	000365-26-4	59
			2-Heptanone, 6-methyl-	12094	000928-68-7	59
			2-(6-Nitro-2-oxo-2H-chromen-4-y...	96408	1000261-28-1	58
48	14.32	0.45	C:\NIST11.L			
			8-Nonen-2-one	17950	005009-32-5	50
			3,7,11,15-Tetramethyl-2-hexadec...	115551	102608-53-7	38
			1,2-Dihexylcyclopropene	61706	035365-52-7	30
49	14.54	0.53	C:\NIST11.L			
			Cyclohexanol, 2-(1-methylethyl)-	19242	000096-07-1	38
			Bicyclo[4.1.0]heptane, 3-methyl-	5868	041977-47-3	38
			Bicyclo[2.2.1]heptane, 1,3,3-tr...	16366	006248-88-0	25
50	15.06	1.58	C:\NIST11.L			
			Hexadecanoic acid, methyl ester	100708	000112-39-0	99
			Pentadecanoic acid, 14-methyl-,...	100727	005129-60-2	97
			Hexadecanoic acid, methyl ester	100707	000112-39-0	97
51	15.55	4.41	C:\NIST11.L			
			n-Hexadecanoic acid	92227	000057-10-3	95
			n-Hexadecanoic acid	92228	000057-10-3	92
			n-Hexadecanoic acid	92226	000057-10-3	86

52	15.91	0.28	C:\NIST11.L			
			Hexadecanoic acid, ethyl ester	108865	000628-97-7	98
			Hexadecanoic acid, ethyl ester	108868	000628-97-7	94
			Hexadecanoic acid, ethyl ester	108867	000628-97-7	83
53	17.26	1.09	C:\NIST11.L			
			9,12-Octadecadienoic acid (Z,Z)...	114386	000112-63-0	99
			9,12-Octadecadienoic acid (Z,Z)...	114388	000112-63-0	99
			9,12-Octadecadienoic acid, meth...	114374	002462-85-3	99
54	17.35	2.91	C:\NIST11.L			
			9,12,15-Octadecatrienoic acid, ...	113305	000301-00-8	99
			9,12,15-Octadecatrienoic acid, ...	113306	000301-00-8	98
			9,12,15-Octadecatrienoic acid, ...	113307	000301-00-8	97
55	17.51	1.54	C:\NIST11.L			
			Phytol	115542	000150-86-7	58
			Phytol	115540	000150-86-7	52
			7-Oxabicyclo[4.1.0]heptane, 1,5...	11124	162239-52-3	49
56	17.96	8.58	C:\NIST11.L			
			9,12,15-Octadecatrien-1-ol, (Z,...	96963	000506-44-5	94
			2-Methyl-Z,Z-3,13-octadecadienol	106377	1000130-90-5	90
			9,12,15-Octadecatrienoic acid, ...	113306	000301-00-8	87
57	18.18	0.59	C:\NIST11.L			
			Linoleic acid ethyl ester	121902	000544-35-4	94
			Linoleic acid ethyl ester	121900	000544-35-4	60
			9,12-Octadecadienoyl chloride, ...	116562	007459-33-8	45
58	18.26	0.89	C:\NIST11.L			
			Ethyl Oleate	122993	000111-62-6	95
			(E)-9-Octadecenoic acid ethyl e...	123033	006114-18-7	64
			Oleic Acid	107517	000112-80-1	56
59	23.69	0.49	C:\NIST11.L			
			1,2-Benzenedicarboxylic acid, m...	105069	004376-20-9	81
			1,2-Benzenedicarboxylic acid, d...	154183	027554-26-3	55
			4-Methoxyanthranilic acid	34134	004294-95-5	50
60	30.44	0.56	C:\NIST11.L			
			N-Methyl-1-adamantaneacetamide	60896	031897-93-5	41
			Methyltris(trimethylsiloxy)silane	122473	017928-28-8	38
			Silane, 1,4-phenylenebis[trimet...	70586	013183-70-5	35
61	31.34	0.62	C:\NIST11.L			
			Stigmastan-3,5-diene	155644	1000214-16-4	50
			Ethane, 1-(4,4,4-trifluoro-1,3-...	122004	1000226-87-3	35
			Acetic acid, [4-(1,1-dimethylet...	70681	088530-52-3	35
62	35.87	0.69	C:\NIST11.L			
			1,3-Bis(trimethylsilyl)benzene	70584	002060-89-1	47
			Tetrasiloxane, decamethyl-	122472	000141-62-8	38

5-Methyl-2-phenylindolizine 60950 036944-99-7 38

Fri Jan 21 17:54:49 2022

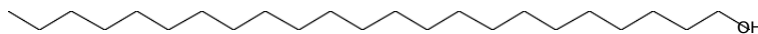

Supplement: Supplementary file 1 [file plants-13-01753-s001.zip › plants-3048557-supplementary/GCMS analysis of ethylacetate, nbutanol and nhexane fraction of B.indica.pdf]
