# Supplementary material for: Pharmacognostic Evaluation, Chemical Characterization, and Antibacterial Activity of Bassia indica (Wight) A.J. Scott
Source: Plants (Basel). 2024 Jun 25;13(13):1753. doi: 10.3390/plants13131753 (PMC11244141; doi:10.3390/plants13131753)
Supplement: Supplementary file 1 [file plants-13-01753-s001.zip › plants-3048557-supplementary/Supplementary data.pdf]

Table S1. Molecular docking scores of co-crystal lingands with targeted proteins

Co-crystal ligand			
**Compound**	**PDB ID**	**Organism**	**Docking score kcal/mol**
2-Amino-2-hydroxymethyl-propane-1,3-diol	4DDQ	*B. subtilis*	-4.4
Chlorobiocin	1KZN	*E. coli*	-8.5
Novobiocin	7PTF	*Pseudomonas*	-9.1
{N}-[3-(4-isoquinolin-1-ylpiperazin-1-yl)propyl]benzamide	6FM4	*S. aureus*	-8.4
Blind docking	5ZTJ	*S. typhi*	-
**Positive control**			
Ceftriaxone	4DDQ	*B. subtilis*	-7.1
	1KZN	*E. coli*	-6.5
	7PTF	*Pseudomonas*	-7.0
	6FM4	*S. aureus*	-8.1
	5ZTJ	*S. typhi*	-8.8

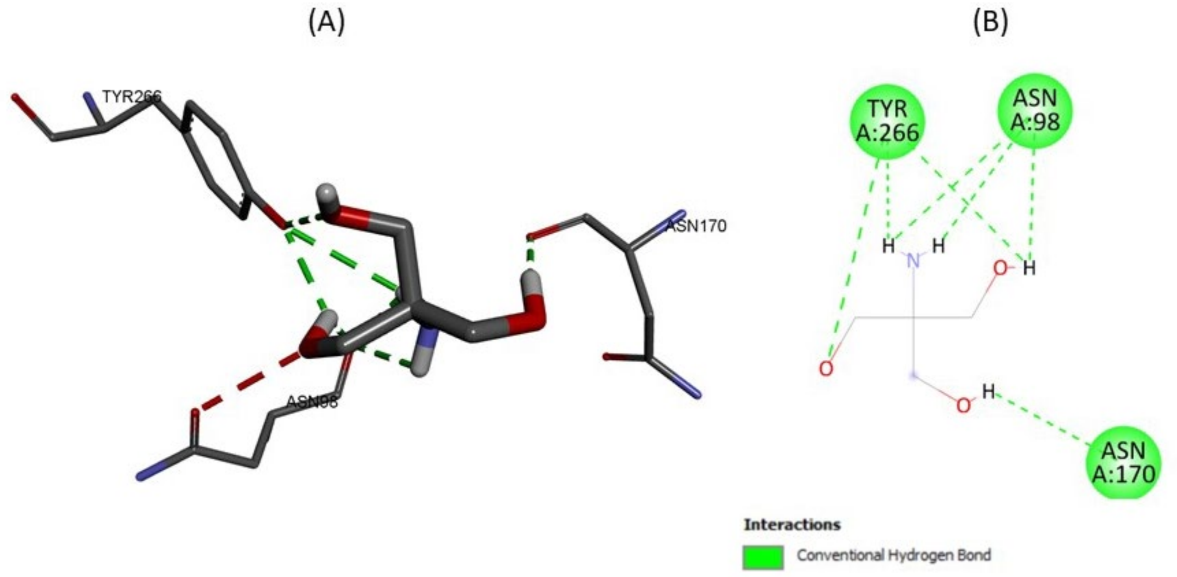

Figure S1. The predicted 3D(A) and 2D(B) binding mode of co-crystal ligand against DNA gyrase subunit B of *B. subtilis*

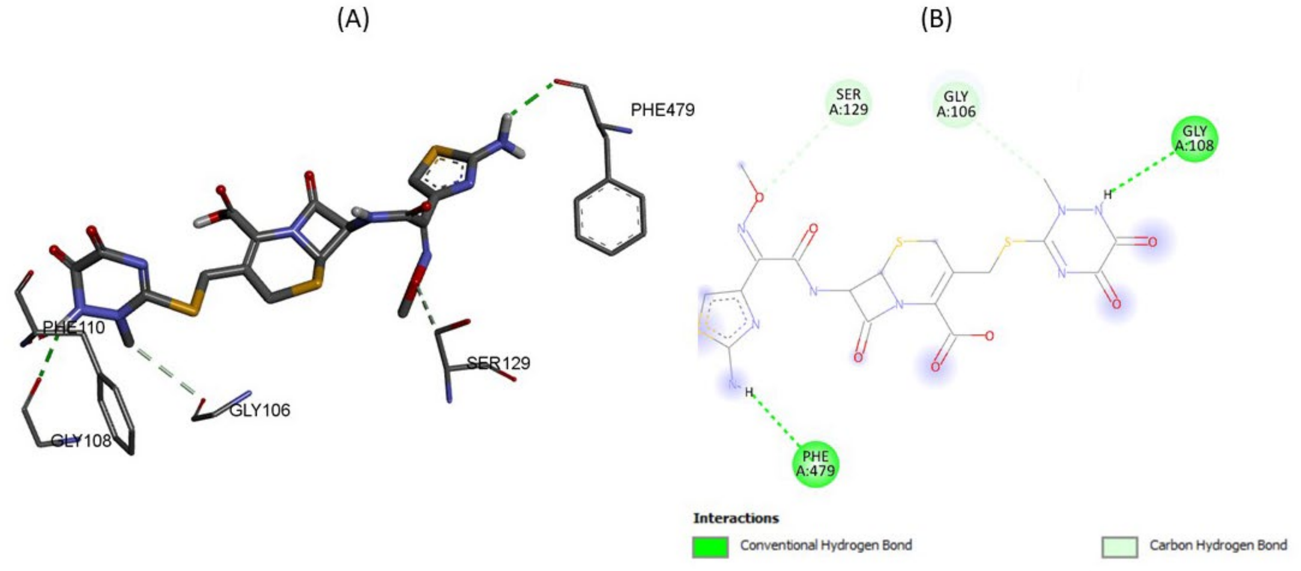

Figure S2. The predicted 3D(A) and 2D(B) binding mode of Ceftriaxone against DNA gyrase subunit B of *B. subtilis*

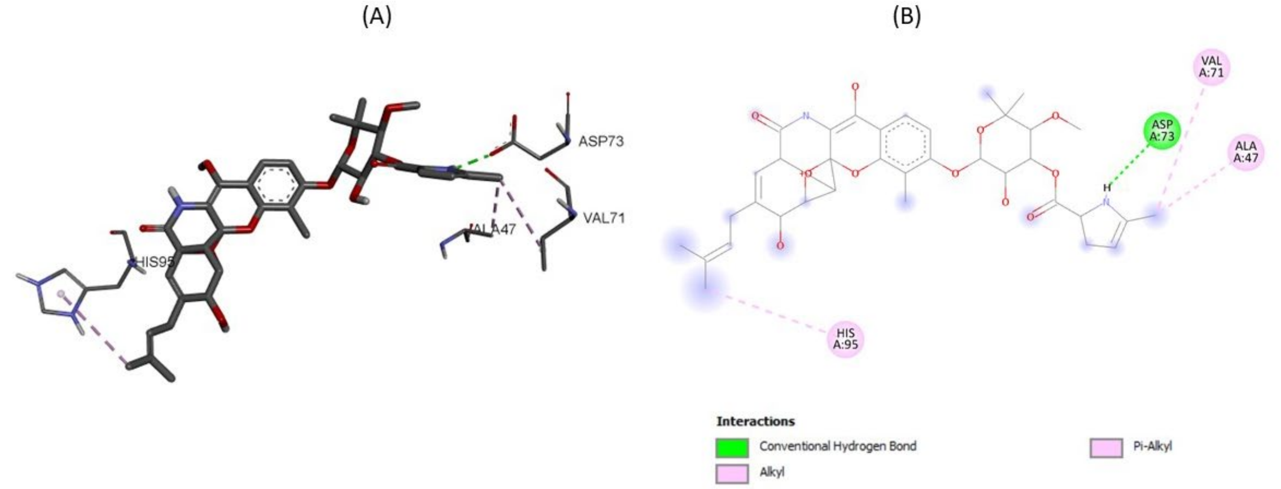

Figure S3.The predicted 3D(A) and 2D(B) binding mode of co-crystal ligand against DNA gyrase subunit B of *E. coli*

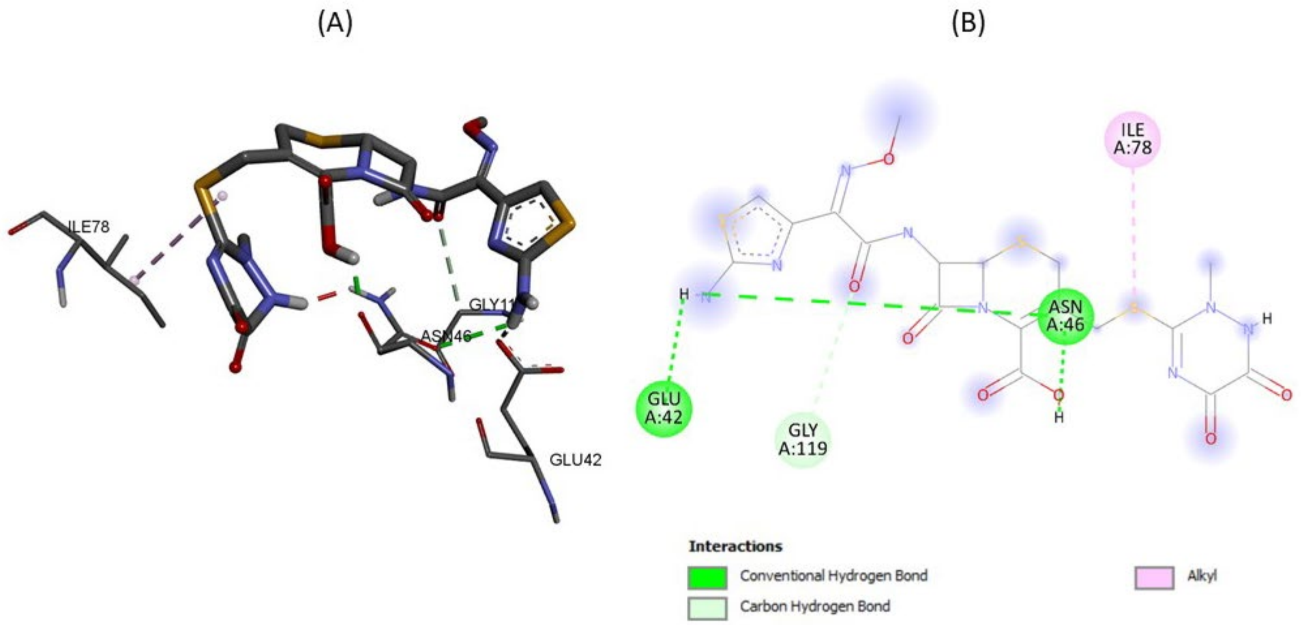

Figure S4. The predicted 3D(A) and 2D(B) binding mode of ceftriaxone against DNA gyrase subunit B of *E. coli*

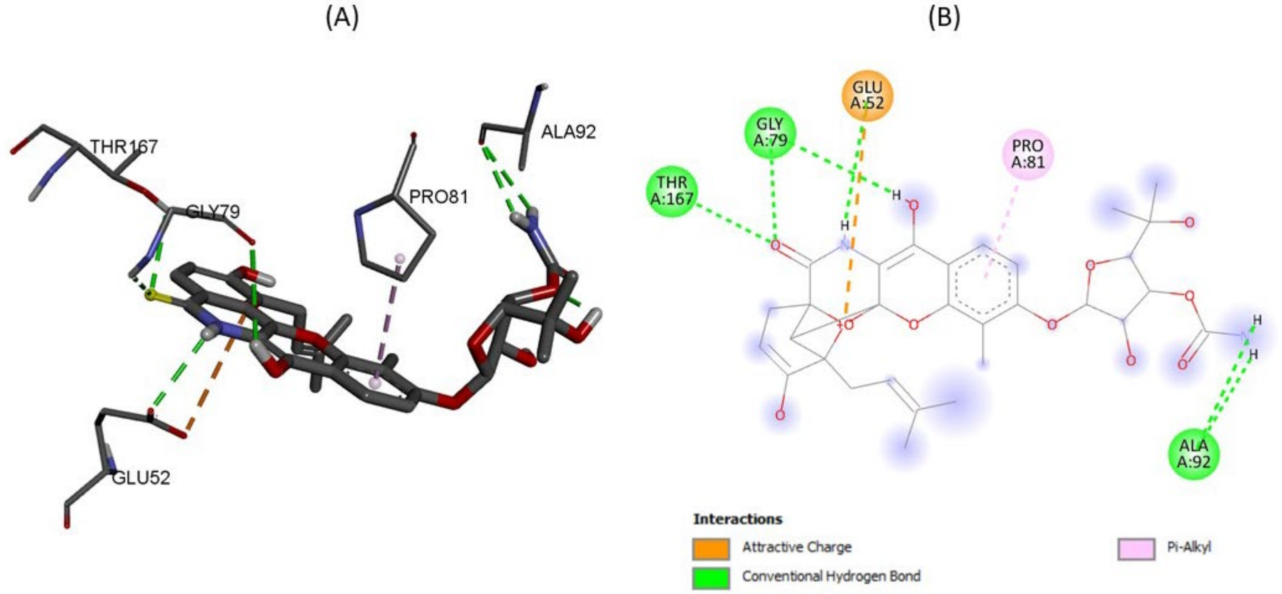

Figure S5. The predicted 3D(A) and 2D(B) binding mode of co-crystal ligand against DNA gyrase subunit B of *Pseudomonas*

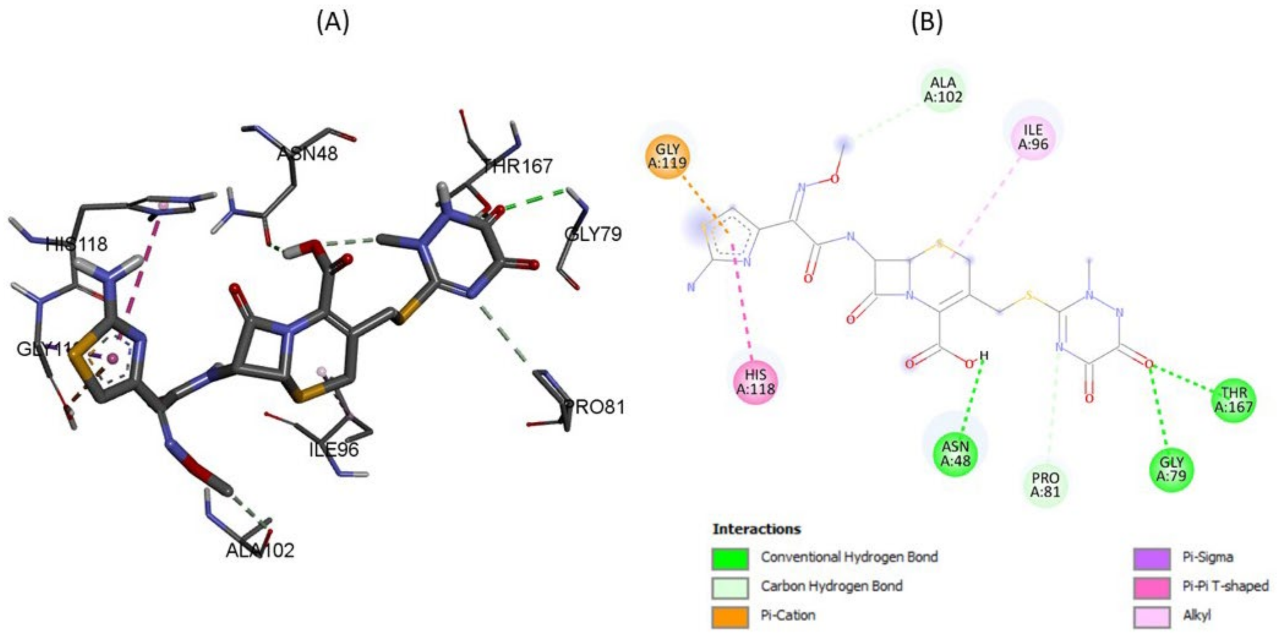

Figure S6. The predicted 3D(A) and 2D(B) binding mode of ceftriaxone against DNA gyrase subunit B of *Pseudomonas*

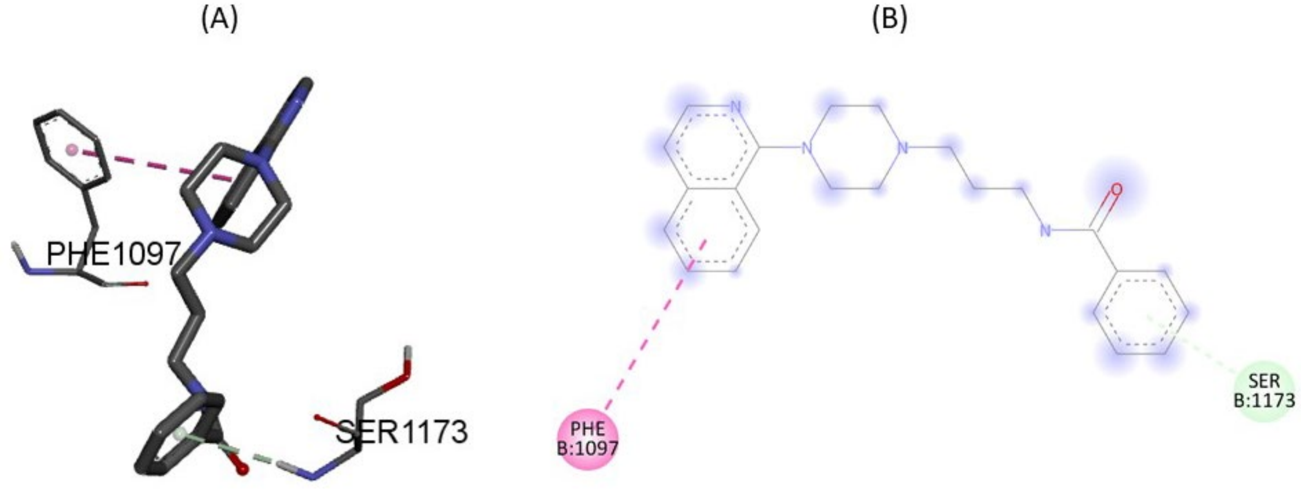

Figure S7. The predicted 3D(A) and 2D(B) binding mode of co-crystal ligand against DNA gyrase subunit B of *S. aureus*

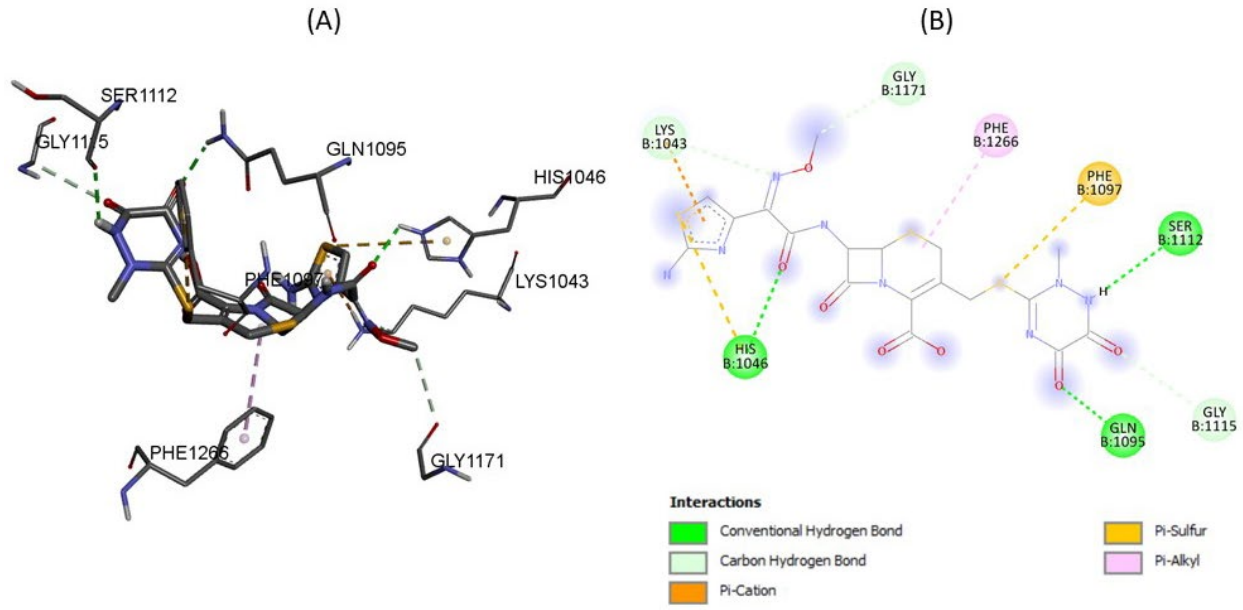

Figure S8. The predicted 3D(A) and 2D(B) binding mode of ceftriaxone against DNA gyrase subunit B of *S. aureus*

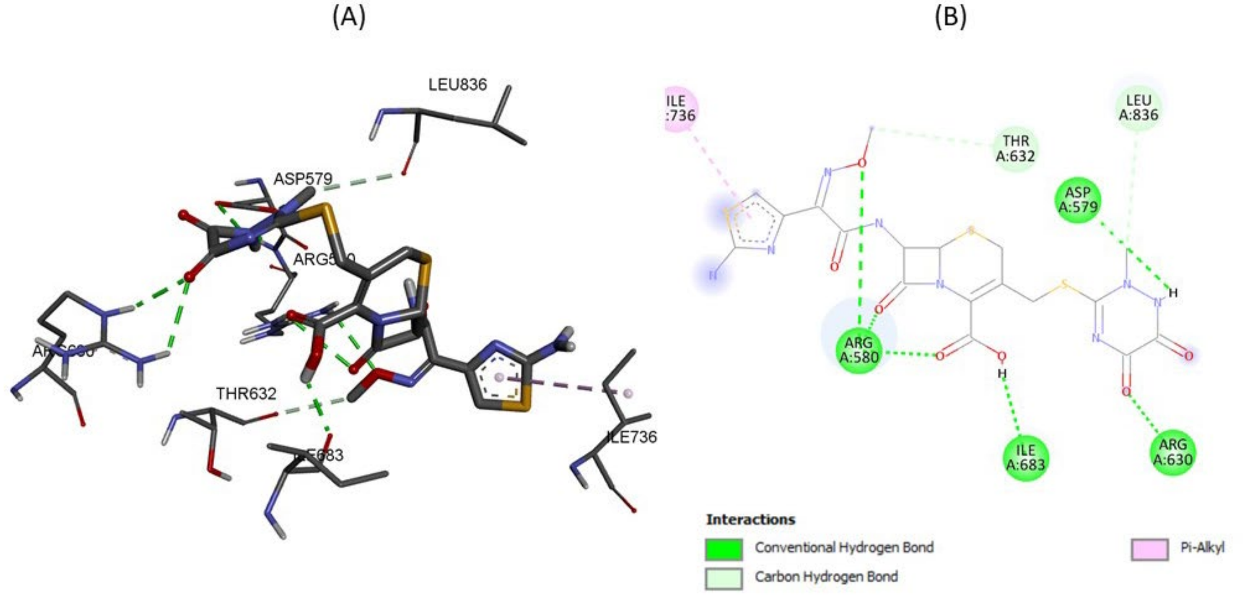

Figure S9. The predicted 3D(A) and 2D(B) binding mode of ceftriaxone against DNA gyrase subunit B of *S. Typhi*
